# Genome-Wide Identification of Neuropeptides and Their Receptors in an Aphid Endoparasitoid Wasp, *Aphidius gifuensi*

**DOI:** 10.3390/insects12080745

**Published:** 2021-08-18

**Authors:** Xue Kong, Zhen-Xiang Li, Yu-Qing Gao, Fang-Hua Liu, Zhen-Zhen Chen, Hong-Gang Tian, Tong-Xian Liu, Yong-Yu Xu, Zhi-Wei Kang

**Affiliations:** 1 College of Plant Protection, Shandong Agricultural University, Tai’an 271018, China; 13145384661@163.com (X.K.); lzx18854807257@163.com (Z.-X.L.); 15550855190@163.com (Y.-Q.G.); liufanghua5@163.com (F.-H.L.); chenzz0327@163.com (Z.-Z.C.); 2 State Key Laboratory of Crop Stress Biology for the Arid Areas, Key Laboratory of Northwest Loess Plateau Crop Pest Management of Ministry of Agriculture, Northwest A&F University, Yangling 712100, China; tianhg@nwsuaf.edu.cn; 3 Key Laboratory of Integrated Crop Pest Management of Shandong Province, College of Plant Health and Medicine, Qingdao Agricultural University, Qingdao 266109, China; txliu@qau.edu.cn

**Keywords:** *Aphidius gifuensis*, neuropeptide precursors, neuropeptide receptors, expression profile

## Abstract

**Simple Summary:**

Neuropeptides and their receptors play important roles in insect physiology and behavior. Due to their specificity and importance, they have been considered as the targets for new environmentally friendly insecticides. Hence, more information on genomics and transcriptomics of neuropeptide and their receptors in more insects, especially in beneficial insects, are needed to avoid the side effects of these environmentally friendly insecticides. *Aphidius gifuensis* is one of the most well-known aphid parasitoids and has been successfully used to control aphids such as *Myzus persicae* and *Sitobion avenae*. In this study, we systematically identified neuropeptides and their receptors from the genome and head transcriptome of *A. gifuensis*. Meanwhile, we also analyzed their expression patterns in response to imidacloprid exposure and in different tissues. Our results not only laid a preliminary foundation for functional studies on neuropeptide precursors and their receptors but also provided useful information for future pesticide development.

**Abstract:**

In insects, neuropeptides and their receptors not only play a critical role in insect physiology and behavior but also are the potential targets for novel pesticide discoveries. *Aphidius gifuensis* is one of the most important and widespread aphid parasitoids, and has been successfully used to control aphid. In the present work, we systematically identified neuropeptides and their receptors from the genome and head transcriptome of *A. gifuensis*. A total of 35 neuropeptide precursors and 49 corresponding receptors were identified. The phylogenetic analyses demonstrated that 35 of these receptors belong to family-A, four belong to family-B, two belong to leucine-rich repeat-containing GPCRs, four belong to receptor guanylyl cyclases, and four belong to receptor tyrosine kinases. Oral ingestion of imidacloprid significantly up-regulated five neuropeptide precursors and four receptors whereas three neuropeptide precursors and eight receptors were significantly down-regulated, which indicated that these neuropeptides and their receptors are potential targets of some commercial insecticides. The RT-qPCR results showed that dopamine receptor 1, dopamine receptor 2, octopamine receptor, allatostatin-A receptor, neuropeptides capa receptor, SIFamide receptor, FMRFamide receptor, tyramine receptor and short neuropeptide F predominantly were expressed in the head whilst the expression of ion transport peptide showed widespread distribution in various tissues. The high expression levels of these genes suggest their important roles in the central nervous system. Taken together, our study provides fundamental information that may further our understanding of neuropeptidergic signaling systems in the regulation of the physiology and behavior of solitary wasps. Furthermore, this information could also aid in the design and discovery of specific and environment-friendly insecticides.

## 1. Introduction

Neuropeptides are 3 to 100 amino acid polypeptides and have been identified as involved in various physiological processes in insects, such as behavior regulation, reproduction, and circadian rhythm [1,2,3,4,5]. For example, in *Drosophila melanogaster*, neuropeptide F (NPF) regulates their feeding behaviors thereby influences their reproductive capacity [2]. In *Schistocerca gregaria*, NPF plays a critical role in reproduction and ecdysteroidogenesis [6]. Silencing insulin-like peptide 2 leads to smaller male mandible size in *Gnatocerus cornutus* [7]. A neuropeptide is encoded by a larger precursor gene (called pre-propeptide) that produces one or more mature peptides by a series of cleavages and modifications [1,8]. Mature peptides exert their biological functions by binding to their corresponding receptors in the extracellular environment [1,9].

Neuropeptide corresponding receptors belong to G protein-coupled receptors (GPCRs), receptor guanylyl cyclases (RGCs), and receptor tyrosine kinases (RTKs) [9,10,11]. Neuropeptide corresponding GPCRs belong to the A-family (or rhodopsin-like), the B-family (or secretin-like), or leucine-rich repeat-containing GPCRs (LGRs) [11]. Insect family A GPCRs include opsins, biogenic amine receptors and neuropeptide and protein hormone receptors [12]. Generally, the B family GPCRs have a long N-terminal domain and contain four types of receptor: corticotrophin-releasing-factor-related diuretic hormone receptor, calcitonin-like diuretic hormone receptor, pigment-dispersing factor receptor and parathyroid hormone receptor-like [13]. According to the numbers of leucine-rich repeat motifs, receptors in LGRs have three types: type A, B and C [14]. Receptors of prothoracicotropic hormone (PTTH), insulin-like peptides and neuroparsin are not GPCRs, but belong to RTKs [15,16]. In *Tribolium castaneum*, knockdown of dopamine-2 receptor results in over 90% mortality during larval–pupal metamorphosis [17]. Interestingly, injected dopamine and its agonists in *Locusta migratoria* cause gregarious behavior in the migratory locust [18]. Injection of dsDop1 (dsRNA of dopamine-1 receptor) and Dop1 antagonist impair the olfactory preference of gregarious locusts to their odors [19]. Notably, the injection of Dop1 agonist in solitarious locusts initiates preference to gregarious odors [19]. In *Helicoverpa armigera*, dopamine receptor is also serviced as a membrane receptor for 20-hydroxyecdysone [20]. Knockdown of CCHamide2 receptors in *A. pisum* influenced its feeding behaviors and thus reproductions [21]. Silencing PTTH receptor torso in the prothoracic gland prolongs the third-instar larval stage of *D. melanogaster* [16]. Topical application of 1895 and CAPA-PVK analogue 2315 (ASG-[*β*^3^L]-VAFPRVamide) cause 72% mortality in *Myzus persicae*, while having no influence on beneficial insects including *Bombus terrestris*, *Chrysoperla carnea*, *Nasonia vitripennis* and *Adalia bipunctata* [22]. Due to the critical role of neuropeptides and their receptors in insect survival, they have been considered as attractive targets for developing new environmentally friendly pesticides to control the economically important pests [5,23,24,25]. Meanwhile, the conservation of these pesticides target genes between target pests and beneficial insects should also be considered to avoid adverse side effects on these beneficial insects [1,24]. Thus, genetic information about neuropeptides and their corresponding receptors in beneficial insects are needed [26,27,28].

*Aphidius gifuensis* has been selected as a potential biological agent of the green peach aphid *Myzus persicae* Sulzer, one of the main pests of various crops in China and Japan, and it has been successfully used to control *M. persicae* on tobacco in Yunnan and many other regions of China [29,30,31,32,33]. *A. gifuensis* is non-host feeding, and a female parasitizes about 60 aphids per day [34]. *A. gifuensis* female is able to discriminate the plant volatiles that released from healthy, mechanically damaged or aphid infested plant [35]. Furthermore, when provided with various aphid species, the *A. gifuensis* female can adjust its parasitic strategy by laying fewer eggs in maternal aphid species and more eggs in other aphid species [30,36]. Interestingly, both virgin and mated *A. gifuensis* females show high preference of aphid alarm pheromone, E-(*β*)-farnesene [37]. All of these results suggested that *A. gifuensis* has evolved a comprehensive chemosensory and nervous system to help it find a suitable host and conduct the right parasitic strategy. The identification and expression of chemosensory genes have been well documented at genomic and transcriptomic levels, whereas neuropeptides and their corresponding receptors were ignored [37,38,39]. In our recent study, we found that exposure of imidacloprid (IMD) significantly disrupted the olfactory and parasitism behavior by influencing the expression of related genes such as detoxification genes, chemosensory genes, and acetylcholine receptors [40]. As for neuropeptide and their receptors, we only analyzed the expression of DopR1, OAR, TAR and NFR [40]. In *D. melanogaster*, neonicotinoids disturbed learning memory, circadian behavior and sleep by preventing the accumulation of pigment dispersing factor neuropeptides [41]. Thus, a comprehensive analysis of the influence of IMD on the expression of neuropeptides and their receptors in *A. gifuensis* is needed. In this study, we predicted the neuropeptide precursors and their receptors using the publicly accessible genome data and our newly sequenced head transcriptome data [38]. Furthermore, we also analyzed the expression of these genes in different tissues and their responses after the exposure of IMD based on our previous study [40]. We thought that the identification and expression of neuropeptides and their receptors provides fundamental information that may further our understanding of neuropeptidergic signaling systems in the regulation of physiology and behavior of solitary wasps. Furthermore, this information could also aid in design and discovery of specific and environment friendly insecticides.

## 2. Materials and Methods

### 2.1. Insect Rearing

A colony of *A. gifuensis* and its host pea aphid, *Acyrthosiphon pisum* Harris kept at 21 ± 1 °C and 16 L: 8D photoperiod. *A. pisum* was reared on broad bean (*Vicia faba* L., var. ‘Jingxuancandou’, Jinnong, Taigu, Shanxi, China). After the emergence of adults, 10% of honey water was provided as supplementary nutrients.

### 2.2. RNA Sequencing and Gene Identification

The heads of *A. gifuensis* were cut from newly emerged adult male and female wasps (1–2 days old), and then frozen in liquid nitrogen. Total RNA of the heads was extracted from 40 heads using RNAiso reagent (Takara Bio, Dalian, China) following manufacturer’s instructions. The RNA integrity and quality were verified by 1% agarose gel electrophoresis and Nanodrop ND-2000 spectrophotometer. A high-quality RNA sample was sent to Novogene Bioinformatics Technology Co., Ltd. for cDNA and Illumina library generation. Transcriptome de novo assembly was carried out as our previously published paper [39,40].

The identification of neuropeptide precursors and their receptors in *A. gifuensis* were predicted by local BLAST searching in the genome and transcriptome of *A. gifuensis* with the amino acid sequences of neuropeptide precursors and their receptors from *Pteromalus puparum*, *Habrobracon hebetor*, *D. melanogaster* and *Nasonia vitripennis* [26,27,28,38,42].

### 2.3. Phylogenetic Analysis

Phylogenetic analyses of these candidate neuropeptide receptors were constructed by PhyML3.1 [43]. Firstly, MAFFT was used to align the amino acid sequences of these receptors. Then, these alignments were manually edited using Bioedit Sequence Alignment Editor 7.1.3.0 (Ibis Pharmaceuticals, Inc., Carlsbad, CA, USA) [44]. Phylogenetic trees were subsequently constructed by PhyML3.1 using the Maximum likelihood (ML) method with 1000 bootstraps [43]. Further editions of these phylogenetic trees were conducted with the ITOL tool [45]. All amino acid sequences for these receptors used in this study are shown in Table S1. For phylogenetic tree of family-A neuropeptide GPCRs, sequences from *A. gifuensis*, *H. hebetor*, *B. mori*, *D. melanogaster*, *P. puparum*, *N. vitripennis*, *Rhodnius prolixus*, *Triatoma dimidiate*, *Triatoma infestans*, *Triatoma pallidipennis*, and *T. castaneum* were used (Table S1). For the phylogenetic tree of the family B neuropeptide GPCRs, sequences from *A. gifuensis*, *H. hebetor*, *B. mori*, *D. melanogaster*, *P. puparum*, *N. vitripennis*, *Rhodnius prolixus*, *Nilaparvata lugens*, and *T. castaneum* were used (Table S1). For **the** phylogenetic tree of the LGRs, sequences from *A. gifuensis*, *H. hebetor*, *A. mellifera*, *A. pisum*, *B. mori*, *D. melanogaster*, *Lymnaea stagnalis*, *Pediculus humanus corporis*, *P. puparum*, *N. vitripennis*, *N. lugens*, *R. prolixus*, *Triatoma dimidiate*, *Tetranychus urticae*, *T. pallidipennis*, and *T. castaneum* were used (Table S1). For the phylogenetic tree of the RGCs, sequences from *A. gifuensis*, *H. hebetor*, *H. hebetor*, *Aedes aegypti*, *Anopheles gambiae*, *D. melanogaster*, *P. puparum*, and *N. vitripennis* were used (Table S1). For the phylogenetic tree of the RTKs, sequences from *A. gifuensis*, *H. hebetor*, *A. aegypti*, *A. gambiae*, *D. melanogaster*, *P. puparum*, and *N. vitripennis* were used (Table S1).

### 2.4. Expression Analysis

Heat maps of neuropeptide precursors and their receptors were performed by pheatmap in R 4.0.4 (https://www.r-project.org/, accessed date (15 February 2021)). Tissue-specific expressions of several target genes were determined by RT-qPCR. Total RNA of heads and bodies (without heads) were extracted using RNAiso reagent (Takara Bio, Dalian) following manufacturer’s protocol. To remove the genomic DNA, the total RNA was treated by Dnase I (42 °C 2 min). These total RNAs were subsequently used to produce cDNA using PrimeScript™ RT reagent Kit with gDNA Eraser (Perfect Real Time, Takara Bio, Tokyo, Japan). Specific primers were designed by Primer Premier 5 (PREMIER Biosoft International, Palo Alto, CA, USA), and are provided in Table S2. qPCR was performed in iQ5 (Bio-rad, Hercules, CA, USA) in 20 μL reactions. The qPCR reaction condition and normalization of these genes were conducted as in our previously published paper [39,46]. Relative expressions of these genes were normalized by 18S RNA, and the normalization of each gene was compared with the expression level in female heads. Each sample had three technical replicates and three biological replicates (50 heads or 20 bodies per replicate). Differences among these samples were subjected to one-way analysis of variance (ANOVA) with Bonferroni’s test at *p* < 0.05.

## 3. Results

### 3.1. Transcriptome Sequencing, Assembly, and Annotation

We sequenced the head of *A. gifuensis* and obtained 48,430,060 clean reads (Table S1). GC content was 28.32% (Table S1). N50 of the head transcriptome was from 546 (transcripts) and 370 (unigenes) (Table S1). Mean length was from 351 (transcripts) and 293 (unigenes) (Table S1). Gene ontology (GO) analysis showed that genes involved in cellular process, metabolic process, single-organism process, binding and catalytic activity were predominantly expressed in head (Figure S1). Notably, signal transduction, translation and endocrine system were the major enrichment pathways (Figure S2).

### 3.2. Identification of Neuropeptide Precursors

**Figure 1:**
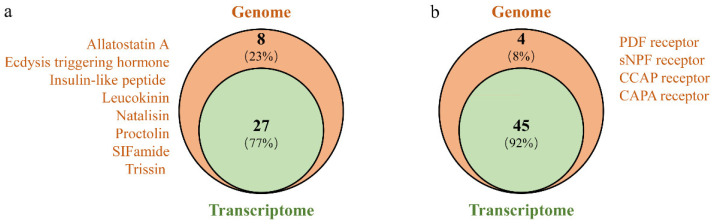
Overlap of neuropeptides (**a**) and their corresponding receptors (**b**) identification results between genomes and transcriptomes databases.

**Figure 2:**
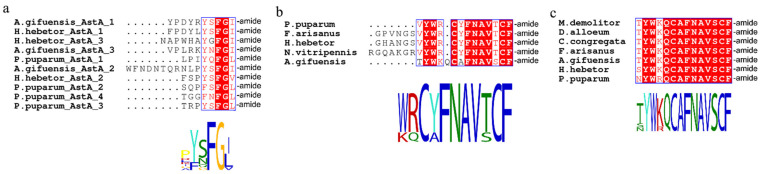
Multiple alignment of allatostatin neuropeptides of *A. gifuensis*. (**a**) allatostatin A; (**b**) allatostatin CC; (**c**) allatostatin CCC.

Based on the *A. gifuensis* genome and transcriptome data, we predicted and annotated 35 neuropeptide precursors from *A. gifuensis* genome and 27 neuropeptide precursors from *A. gifuensis* transcriptome (Figure 1; Table 1 and Table S2). Adipokinetic hormone, allatostatin B, allatostatin C, RYamide, sulfakinin and tachykinin were missing in *A. gifuensis* (Table 1). Allatostatin A, ecdysis triggering hormone, insulin-like peptide (ILP), leucokinin, natalisin, proctolin, SIFamide and trissin were only observed in the genome of *A. gifuensis*, while they were not found in the head transcriptome (Figure 1a). For the allatostatin neuropeptide family, allatostatin CCC was most conserved whereas allatostatin A was the most variable of the three allatostatin neuropeptides (Figure 2).

### 3.3. Neuropeptide Receptors in A. gifuensis

Based on the genomics and transcriptomics analyses, we also predicted 49 neuropeptide receptor genes (Table S1), including 35 A-families GPCRs, four B-families GPCRs, two LGRs, four RGCs, and four RTKs. Receptors for PDF, sNPF, CAPA and CCAP were only found in the genome of *A. gifuensis* (Figure 1b).

**Figure 3:**
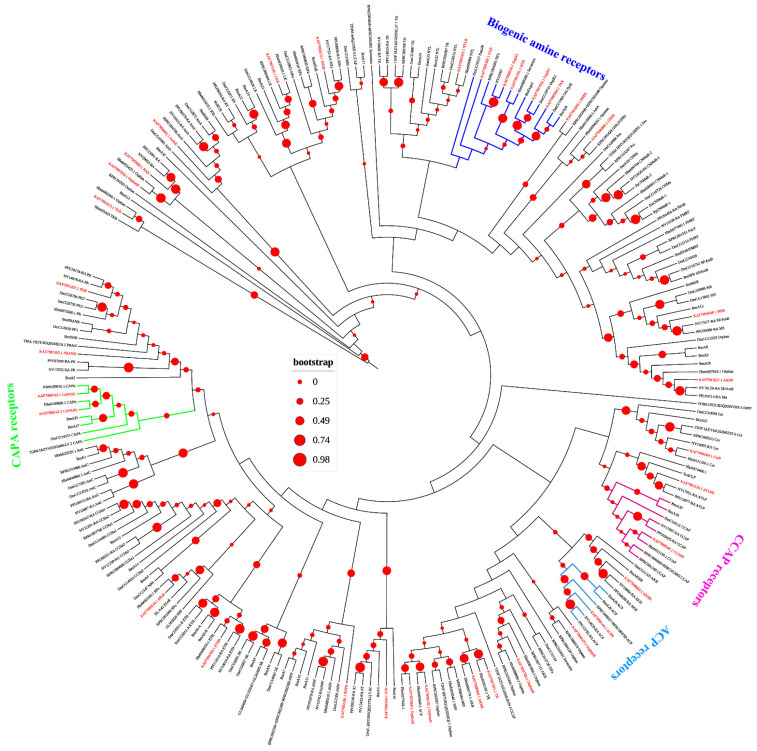
Phylogenetic tree analysis of the family-A neuropeptide G protein-coupled receptors (GPCRs). *A. gifuensis* (KAF), *H. hebetor* (Hh), *B. mori* (Bm), *D. melanogaster* (Dm), *P. puparum* (Pp), *N. vitripennis* (Nv), *Rhodnius prolixus* (RPR), *Triatoma dimidiate* (TDIM), *Triatoma infestans* (TINF), *Triatoma pallidipennis* (TPAL), and *T. castaneum* (Tc). The GPCRs from *A. gifuensis* are highlighted in red.

In the present study, our 35 identified A-family GPCRs include opsins and biogenic amine receptors, and neuropeptide and protein hormone receptors (Figure 3). There are two CAPA splicing variant a (CAPA) receptors (KAF7988142.1 and KAF7988143.1), one crustacean cardioactive peptide (CCAP) receptor (KAF7998540.1), and one AKH/corazonin-related peptide (ACP) receptor (KAF7998144.1; Figure 3). We also found one AKH receptor (KAF7998064.1) while AKH were not found in *A. gifuensis* (Table 1 and Figure 3). For biogenic amine receptors, we identified two dopamine like receptors (KAF7998503.1: DopR1 and KAF7997032.1: DopR2), one serotonin receptor (KAF7991191.1: THR), one octopamine-tyramine receptor (KAF7998304.1: TAR) and one octopamine receptor (KAF7995300.1: OAR; Figure 3).

**Figure 4:**
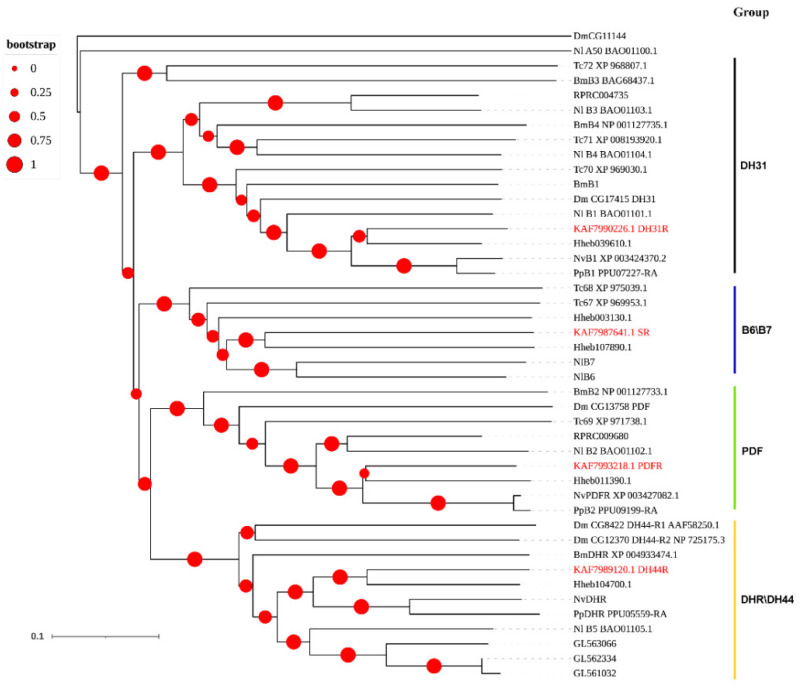
Phylogenetic tree of the family B neuropeptide GPCRs. *A. gifuensis* (KAF), *H. hebetor* (Hh), *B. mori* (Bm), *D. melanogaster* (Dm), *P. puparum* (Pp), *N. vitripennis* (Nv), *Rhodnius prolixus* (RPR), *Nilaparvata lugens* (Nl), and *T. castaneum* (Tc). The GPCRs from *A. gifuensis* are highlighted in red.

A total of four Family B GPCRs were identified from the genome and transcriptome of *A. gifuensis*. Interestingly, these four receptors were divided into four different groups, respectively (Figure 4).

**Figure 5:**
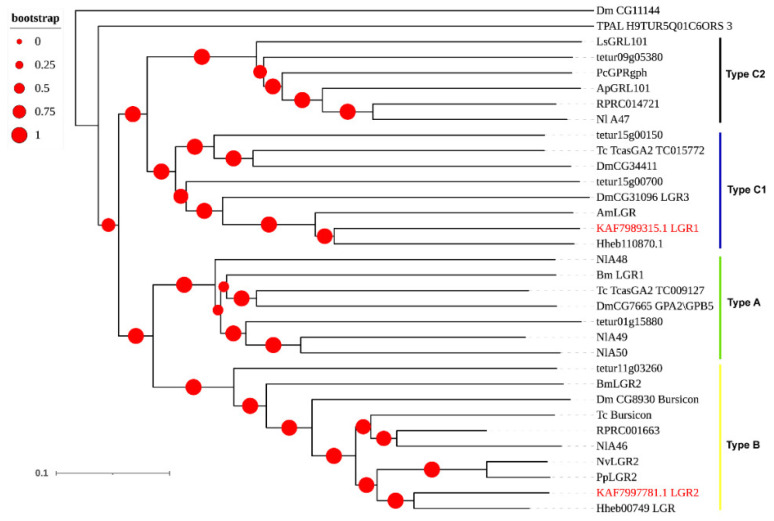
Phylogenetic tree of the leucine-rich repeat-containing GPCRs (LGRs). *A. gifuensis* (KAF), *H. hebetor* (Hh), *A. mellifera* (Am), *A. pisum* (Ap), *B. mori* (Bm), *D. melanogaster* (Dm), *Lymnaea stagnalis* (Ls), *Pediculus humanus corporis* (Pc), *P. puparum* (Pp), *N. vitripennis* (Nv), *Nilaparvata lugens* (Nl), *R. prolixus* (RPR), *T. dimidiate* (TDIM), *Tetranychus urticae* (tetur), *T. pallidipennis* (TPAL), and *T. castaneum* (Tc). The GPCRs from *A. gifuensis* are highlighted in red.

For LGRs, a type C1 leucine-rich repeat (KAF7989315.1: LGR1) and a type B LRR (KAF7997781.1: LGR2) were predicted (Figure 5), and the ligand of LGR1 and LGR2 were orphan and bursicon, respectively (Table 1). Furthermore, the most similar genes of these two *A. gifuensis* LGRs were both from ectoparasitoid *H. hebetor*.

**Figure 6:**
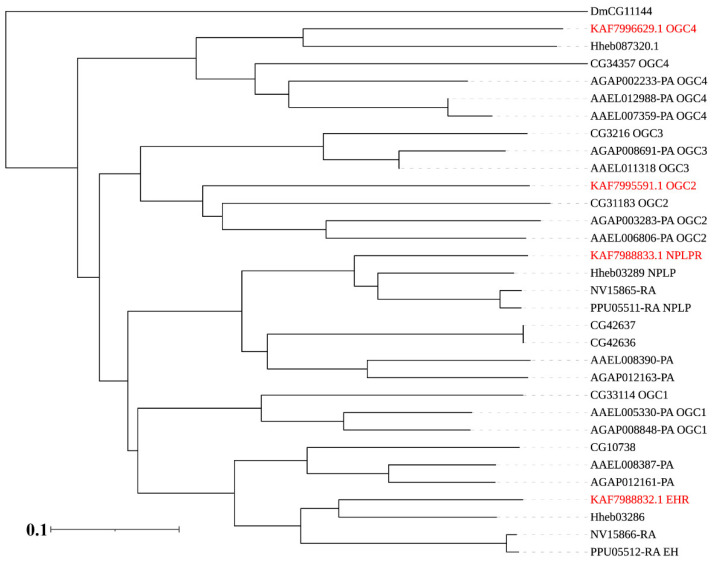
Phylogenetic tree of the receptor guanylyl cyclases (RGCs). *A. gifuensis* (KAF), *H. hebetor* (Hh), *H. hebetor* (Hh), *Aedes aegypti* (AAEL), *Anopheles gambiae* (AGAP), *D. melanogaster* (Dm), *P. puparum* (Pp), and *N. vitripennis* (Nv). The GPCRs from *A. gifuensis* are highlighted in red.

In addition to RGCs, we found one receptor for the eclosion hormone (KAF7988832.1: EHR) and neuropeptide-like precursor (KAF7988833.1: NPLPR) resepectively, and two orphan receptor guanylyl cyclases (KAF7995591.1: OGC2 and KAF7996629.1: OGC4) in *A. gifuensis* (Figure 6).

**Figure 7:**
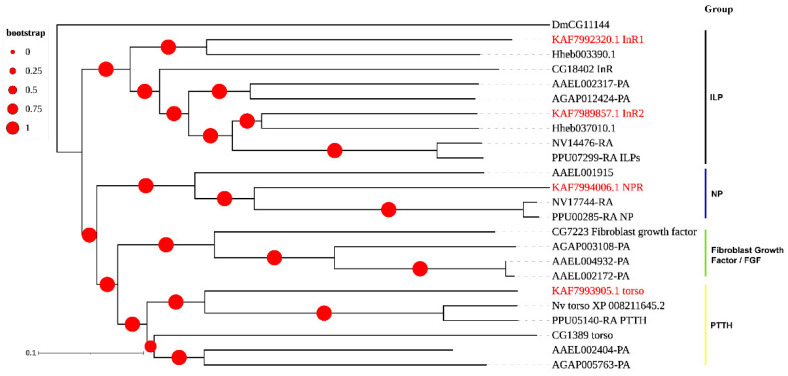
Phylogenetic tree of the receptor tyrosine kinases (RTKs). *A. gifuensis* (KAF), *H. hebetor* (Hh), *A. aegypti* (AAEL), *A. gambiae* (AGAP), *D. melanogaster* (Dm), *P. puparum* (Pp), and *N. vitripennis* (Nv). The GPCRs from *A. gifuensis* are highlighted in red.

As for RTKs, we found one PTTH receptor torso (KAF7993905.1), two insulin-like peptide (ILP) receptors (KAF7992320.1: InR1 and KAF7989857.1: InR2) and one neuropeptide (NP) receptor (KAF7994006.1), while no fibroblast growth factor (FGF) receptor was found in *A. gifuensis* (Figure 7).

### 3.4. Expression Profiles of Neuropeptides and Their Receptors

**Figure 8:**
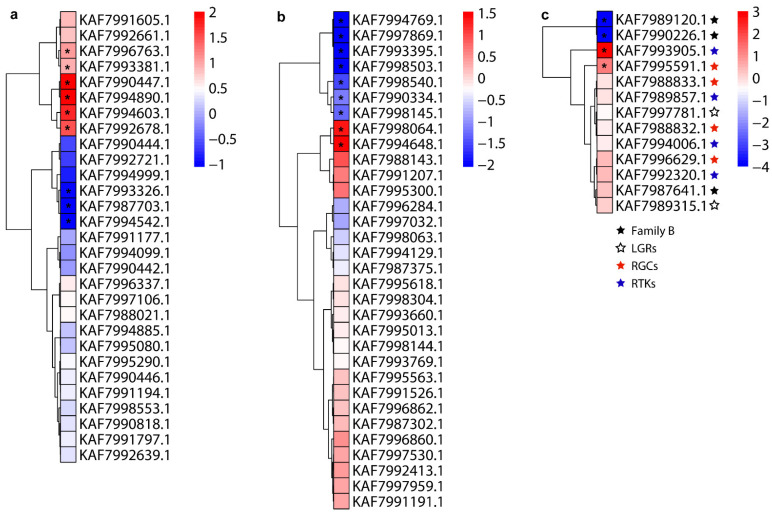
Expression profiles of *A. gifuensis* neuropeptide precursors and their receptors in response to imidacloprid (IMD). (**a**) neuropeptide precursors; (**b**) the family-A neuropeptide GPCRs; (**c**) receptors of the family-B GPCRs, LGRs, RGCs and RTKs. Asterisk (*) indicated that an absolute value of log2Ratio ≥1/≤−1 and FDR < 0.05.

Based on previously published and our newly sequenced transcriptome data, we analyzed the expression profiles of neuropeptides and their receptors in response to IMD exposure (Figure 8). We found that allatostatin CC (KAF7990447.1; AstCC), diuretic hormone 44 (KAF7994603.1; DH44), elevenin (KAF7994890.1; Ele), ion transport peptide (KAF7996763.1; ITP), NVP-like putative neuropeptide (KAF7992678.1; NVP) and SIFamide (KAF7993381.1) were significantly higher in IMD-treated *A. gifuensis*, whereas the expressions of FMRFamide (KAF7993326.1), Natalisin (KAF7987703.1) and pigment dispersing factor (KAF7994542.1; PDF) were significantly lower in IMD-treated *A. gifuensis* (Figure 8a. As to neuropeptide receptors, IMD significantly induced the expressions of AKHR (KAF7998064.1), myosuppressin receptor (KAF7994648.1; MSR), OGC2 (KAF7995591.1) and torso (KAF7993905.1), while the expressions of ecdysis triggering hormone receptor (KAF7994769.1: ETHR), neuropeptide receptor A5 (KAF7990334.1: ATR), CCAPR (KAF7998540.1), KAF7998145.1, NPFR (KAF7993395.1), DopR1 (KAF7998503.1), adhesion G protein-coupled receptor A3 (KAF7997869.1: OrphanR), diuretic hormone 44 receptor (KAF7989120.1: DH44R) and diuretic hormone 31 receptor (KAF7990226.1: DH31R) were down-regulated by IMD treatment (Figure 8b,c).

**Figure 9:**
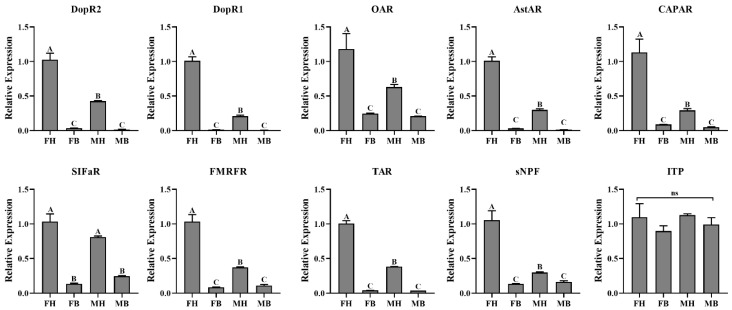
The tissue-specific transcript abundances of *A. gifuensis* neuropeptide precursors and their receptors. FH: female head, MH: male head, FB: female body, MB: male body. AstAR: allatostatin-A receptor; CAPAR: neuropeptides capa receptor; SIFaR: SIFamide receptor; FMRFR: FMRFamide receptor; sNPF: short neuropeptide F; ITP: ion transport peptide. The normalization of each gene was compared with the expression level in female heads. The error bars represent standard errors and the letters above each bar indicate significant differences in transcript abundances (*p* < 0.05).

Furthermore, we also analyzed their expressions in different tissues by RT-qPCR. We found that sNPF was highly expressed in the head, while the expression of sNPF in the female head was significantly higher than that in the male head (Figure 9). The expression trends of DopR1, DopR2, AstAR, FMRFR (FMRFamid receptor), TAR, CAPAR and OAR were similar with sNPF (Figure 9). SIFaR (SIFamide receptor) was predominantly expressed in the head, and there was no significant difference between female and male (Figure 9). There was no tissue-specific expression of ITP (ion transport peptide; Figure 9).

## 4. Discussion

In the present study, we systematically identified neuropeptide precursors and their receptors of *A. gifuensis* from the previously released genome and our sequenced head transcriptome databases. The numbers of our identified neuropeptide precursors and their receptors in *A. gifuensis* genome are similar to those found in other parasitic wasps such as *P. puparum*, *H. hebetor* and *N. vitripennis* [26,27,28]. However, the numbers are less than those in *D. melanogaster*, *Bombyx mori*, *N. lugens*, *A. aegypti*, *L. migratoria*, *Rhynchophorus ferrugineus*, *Aphis mellifera* and *T. castaneum*, which might be due to the parasitic life of parasitic wasps [8,42,47,48,49,50,51]. Notably, there were three very conservative neuropeptide precursors (adipokinetic hormone, RYamide and tachykinin) not found in the *A. gifuensis* genome and transcriptome. RYamide was firstly found in *N. vitripennis* with the C-terminal consensus sequence FFXGSRY-amide [28]. In *D. melanogaster* and *B. mori*, RYamide or its receptor was found to be expressed in the brain, terminal abdominal ganglion and gut (enteroendocrine cells: EEs) [52,53]. Injection of RYamide significantly depressed the proboscis extension reflex of *Phormia regina* to sucrose [54]. Tachykinin is a multifunctional neuropeptide with a characteristic conserved C-terminal sequence FXGXR-amide [55,56]. In *Bactrocera dorsalis*, tachykinin modulates the olfactory sensitivity to ethyl acetate (avoid aversive odor) [57]. Knockdown of tachykinin significantly decreased the electrophysiological responses to ethyl acetate and suppressed the avoid behavior for ethyl acetate [57]. Furthermore, tachykinin is one of the most abundant secreted peptides expressed in midgut EEs [55]. In *D. melanogaster* and *Plutella xylostella*, tachykinin has been found to play a critical role in lipid metabolism [55,58]. Therefore, we speculate that the absence of some neuropeptide precursors might be due to a method as some specific neuropeptide genes that might not be identified by homolog searching. In the future, peptidomics is an alternative method to be used for identifying the neuropeptide genes and their products. Furthermore, it also can be used for confirming the predicted neuropeptide genes from genome and transcriptome.

Generally, expressions of genes in different tissues reflect their functions. In this study, sNPF, DopR1, DopR2, AstAR, FMRFR, TAR, CAPAR and OAR predominately expressed the head, while ITP was widely expressed among these samples. Consistent with our results, three dopamine receptors in *Chilo suppressalis* were also found to be significantly highly expressed in the central nervous tissues [59]. In *Agrotis ipsilon*, brains and antennal lobes predominately expressed DopR1 that is involved in sex pheromone responsiveness [60]. Similarly, TAR1 is highly expressed in the antennae, and silencing TAR1 reduced the sensitivity of *Halyomorpha halys* to its alarm pheromone (E)-2-decenal [61]. In *L. migratoria*, dopamine and octopamine signaling mediated the olfactory behavior and ultimately influenced the phase change [18,43,62]. Silencing DopR1/2 and OARα1 disrupted the attractive and repulsive behavior of *L. migratoria* to their odors [18,19,62,63]. For FMRFR, RNAi silencing of FMRFR decreased the locomotor and flight activities in *D. melanogaster* [64,65]. In previous studies, *A. gifuensis* showed higher parasitoid preference and suitability on its maternal host aphids than other aphid species [30]. Natal rearing experience affects the host discrimination and oviposition decision of *A. gifuensis* [36]. Due to the importance of neuropeptides and their receptors in the modulation of insect behavior, all of these results indicated that neuropeptides and their receptors in *A. gifuensis* may play a critical role in the odor perception and successful parasitism.

Based on previously published work, we analyzed the expression of neuropeptide precursors and their receptors in response to IMD [40]. The expressions of AstCC, DH44, Ele, NVP and SIFamide were up-regulated by IMD whereas FMRFamide, Natalisin and PDF were down-regulated. In *D. melanogaster*, PDF mutants showed a decreased male sex pheromones and increased frequency of remating behaviors [66]. Exposure of IMD disrupted the learning, behavioral rhythmicity and sleep of *D. melanogaster* [41]. Further investigation revealed that IMD prevented the accumulation of PDF in the dorsal terminals of clock neurons [41]. SIFamides are highly conserved neuropeptide precursors and have been identified to be involved in the regulation of sexual behavior, feeding and sleep [1,67,68]. Knockdown SIFamide in *Rhodnius prolixus* caused smaller blood meal consumption, while the injection of Rhopr-SIFa resulted in a larger blood meal [67]. Surprisingly, mutations and silencing (RNAi) of SIFamide in *D. melanogaster* led to male-male courtship behavior whilst the activation of SIFamidergic neuron enhances appetitive behavior [68]. Our previous work reported that some of the IMD-exposed females *A. gifuensis* bent their abdomens forward as if they were mating or attacking aphids, while no male *A. gifuensis* and aphids were present [40]. All of these results indicated that this abnormal behavior might be caused by the impact of IMD on these neuropeptide precursors that eventually leads to nervous disorder.

In the pharmaceutical industry, GPCRs are one of the therapeutic targets for the development of new drugs [23,24]. About 40–50% of all medications exert their effects via this family of proteins such as losartan, olanzapine and cinacalcet [9,23]. Due to the important roles of GPCRs in modulating biology, physiology and behavior in insects, GPCRs are also considered as targets for next generation insecticides. In contrast to the pharmaceutical industry (knowledge-driven drug design), research on insect GPCRs especially for new pesticide discovery is lacking [18,24]. Amitraz is the only pesticide that targets the octopamine receptor and tyramine receptor [69,70]. Recently, a research group provided a novel approach “proof-of-concept” for insecticide discovery using genome sequence data for functional characterization and chemical molecules screening of GPCRs [71]. They successfully found two lethal molecules (amitriptyline: 93% mortality and doxepin: 72% mortality) for *A. aegypti* [71]. In this work, we found that exposure of IMD significantly influenced the expression of GPCRs in *A. gifuensis* including dopamine receptor and FMRFamide receptor, which means random ligand screening for novel insecticide discovery might impair beneficial insects including natural enemies of insect pests and pollinators. Thus, genetic information of these beneficial insects will contribute to our current and future efforts in rational insecticide discovery and design.

Taken together, in this study, we systematically identified the neuropeptide precursors and their receptors in *A. gifuensis*. Meanwhile, we also analyzed their expression patterns in response to IMD exposure and different tissues. Our results not only laid a preliminary foundation for the functional studies on neuropeptide precursors and their receptors but also provided useful information for future pesticide development.

## Figures and Tables

**Table 1:** Description of neuropeptide genes in the genome of *A. gifuensis*.

Peptide Name	Gene ID	Acronym	Protein (AA)	Assigned Receptor ID
Allatostatin A	KAF7998325.1	AstA	181	KAF7996862.1
Allatostatin CC	KAF7990447.1	AstCC	136	nd ^1^
Allatostatin CCC	KAF7990446.1	AstCCC	90	nd
Bursicon alpha	KAF7994885.1	Burα	147	KAF7997781.1
Bursicon beta	KAF7994885.1	Burβ	123	KAF7997781.1
CAPA splicing variant a	KAF7994999.1	CAPA	103	KAF7988142.1 KAF7988143.1
Crustacean cardioactive peptide	KAF7995080.1	CCAP	130	KAF7998540.1
CNMamide	KAF7990444.1	CNMa	152	nd
Corazonin	KAF7991797.1	Crz	122	KAF7996284.1
Diuretic hormone 31	KAF7996337.1	DH31	110	KAF7990226.1
Diuretic hormone 44	KAF7994603.1	DH44	198	KAF7989120.1
Eclosion hormone	KAF7992721.1	EH	85	KAF7988832.1
Ecdysis triggering hormone	KAF7993382.1	ETH	168	KAF7994769.1
FMRFamide	KAF7993326.1	FMRF	305	nd
IDLSRF-like peptide	KAF7991605.1	IDLSRF	201	nd
Insulin-like peptide 1	KAF7990199.1	ILP1	645	KAF7992320.1 KAF7989857.1
Elevenin	KAF7994890.1	Ele	151	nd
Inotocin	KAF7991194.1	Inotocin	143	nd
ITG-like peptide	KAF7998553.1	ITG	237	nd
Ion transport peptide	KAF7996763.1	ITP	225	nd
Leucokinin	KAF7987704.1	LK	218	KAF7997530.1
Myosupressin	KAF7994099.1	MS	99	KAF7989857.1
Natalisin	KAF7987703.1	NTL	296	KAF7995563.1
Neuroparsin	KAF7991177.1	NP	130	KAF7994006.1
Neuropeptide F 2	KAF7997106.1	NPF2	123	KAF7993395.1
NVP-like putative neuropeptide	KAF7992678.1	NVP	735	nd
Neuropeptide-like precursor	KAF7992639.1	NPLP	526	KAF7988833.1
Orcokinin-A	KAF7990442.1	OKA	154	nd
Pheromone biosynthesis activating Neuropeptide/hugin-pyrokinin	KAF7995290.1	PBAN	158	KAF7987302.1 KAF7991207.1
Pigment dispersing factor	KAF7994542.1	PDF	88	KAF7993218.1
Proctolin	KAF7992386.1	Pro	220	nd
Prothoracicotropic hormone	KAF7990443.1	PTTH	113	KAF7993905.1
Short neuropeptide F	KAF7990818.1	sNPF	95	KAF7992286.1
SIFamide	KAF7993381.1	SIFa	123	KAF7995013.1
Trissin	KAF7988021.1	Tris	76	nd

^1^ nd means not found.

## Data Availability

All the individual data are gathered in the Supplemental material section. Transcriptome data is available in NCBI bioproject PRJNA733581.

## Supplementary Materials

The following are available online at https://www.mdpi.com/article/10.3390/insects12080745/s1, Figure S1: Functional annotation of *A. gifuensis* transcripts based on gene ontology (GO) categorization, Figure S2: Top 20 enriched Kyoto Encyclopedia of Genes and Genomics (KEGG) pathways of *A. gifuensis*, Table S1: The amino acid sequences of neuropeptide receptors, Table S2: Primers used in this study, Table S3: Summary of head transcriptome, Table S4: The amino acid sequences of neuropeptide precursors. Predicted signal peptides (highlighted in yellow), cleavage signals (red), putative bioactive mature peptides (light blue), amidation signals (pink), and cysteine residues (deep yellow) are indicated.

## References

[1] Schoofs L., De Loof A., Van Hiel M.B. (2017). Neuropeptides as regulators of behavior in insects. Annu. Rev. Ѐntomol..

[2] Van Wielendaele P., Wynant N., Dillen S., Zels S., Badisco L., Broeck J.V. (2013). Neuropeptide F regulates male reproductive processes in the desert locust, *Schistocerca gregaria*. Insect Biochem. Mol. Biol..

[3] Ragionieri L., Predel R. (2019). The neuropeptidome of *Carabus* (Coleoptera, Adephaga: Carabidae). Insect Biochem. Mol. Biol..

[4] Hou L., Yang P., Jiang F., Liu Q., Wang X., Kang L. (2017). The neuropeptide F/nitric oxide pathway is essential for shaping locomotor plasticity underlying locust phase transition. eLife.

[5] Ons S. (2017). Neuropeptides in the regulation of *Rhodnius prolixus* physiology. J. Insect Physiol..

[6] Van Wielendaele P., Dillen S., Zels S., Badisco L., Broeck J.V. (2013). Regulation of feeding by Neuropeptide F in the desert locust, *Schistocerca gregaria*. Insect Biochem. Mol. Biol..

[7] Okada Y., Katsuki M., Okamoto N., Fujioka H., Okada K. (2019). A specific type of insulin-like peptide regulates the conditional growth of a beetle weapon. PLoS Biol..

[8] Hou L., Jiang F., Yang P., Wang X., Kang L. (2015). Molecular characterization and expression profiles of neuropeptide precursors in the migratory locust. Insect Biochem. Mol. Biol..

[9] Audsley N., Down R.E. (2015). G protein coupled receptors as targets for next generation pesticides. Insect Biochem. Mol. Biol..

[10] Ons S., Lavore A., Sterkel M., Wulff J.P., Sierra I., Martínez-Barnetche J., Rodriguez M.H., Rivera-Pomar R. (2016). Identification of G protein coupled receptors for opsines and neurohormones in *Rhodnius prolixus*. Genomic and transcriptomic analysis. Insect Biochem. Mol. Biol..

[11] Jaeger W.C., Armstrong S.P., Hill S.J., Pfleger K.D.G. (2014). Biophysical detection of diversity and bias in GPCR function. Front. Endocrinol..

[12] Li C., Song X., Chen X., Liu X., Sang M., Wu W., Yun X., Hu X., Li B. (2014). Identification and comparative analysis of G protein-coupled receptors in *Pediculus humanus humanus*. Genomics.

[13] Harmar A.J. (2001). Family-B G-protein-coupled receptors. Genome Biol..

[14] Van Hiel B., Vandersmissen H.P., Van Loy T., Broeck J.V. (2012). An evolutionary comparison of leucine-rich repeat containing G protein-coupled receptors reveals a novel LGR subtype. Peptides.

[15] Vogel K., Brown M.R., Strand M.R. (2015). Ovary ecdysteroidogenic hormone requires a receptor tyrosine kinase to activate egg formation in the mosquito *Aedes aegypti*. Proc. Natl. Acad. Sci. USA.

[16] Rewitz K.F., Yamanaka N., Gilbert L.I., O’Connor M.B. (2009). The insect neuropeptide ptth activates receptor tyrosine kinase torso to initiate metamorphosis. Science.

[17] Bai H., Zhu F., Shah K., Palli S.R. (2011). Large-scale RNAi screen of G protein-coupled receptors involved in larval growth, molting and metamorphosis in the red flour beetle. BMC Genom..

[18] Guo X., Ma Z., Kang L. (2015). Two dopamine receptors play different roles in phase change of the migratory locust. Front. Behav. Neurosci..

[19] Guo X., Ma Z., Du B., Li T., Li W., Xu L., He J., Kang L. (2018). Dop1 enhances conspecific olfactory attraction by inhibiting miR-9a maturation in locusts. Nat. Commun..

[20] Kang X.L., Zhang J.Y., Wang D., Zhao Y.M., Han X.L., Wang J.X., Zhao X.F. (2019). The steroid hormone 20-hydroxyecdysone binds to dopamine receptor to repress lepidopteran insect feeding and promote pupation. PLoS Genet..

[21] Shahid S., Shi Y., Yang C., Li J.J., Ali M.Y., Smagghe G., Liu T.X. (2021). CCHamide2-receptor regulates feeding behavior in the pea aphid, *Acyrthosiphon pisum*. Peptide.

[22] Shi Y., Nachman R.J., Gui S., Piot N., Kaczmarek K., Zabrocki J., Dow J.A.T., Davies S., Smagghe G. (2021). Efficacy and biosafety assessment of neuropeptide CAPA analogues against the peach-potato aphid (*Myzus persicae*). Insect Sci..

[23] Garland S.L. (2013). Are GPCRs still a source of new targets?. J. Biomol. Screen.

[24] Yang D., Zhou Q., Labroska V., Qin S., Darbalaei S., Wu Y., Yuliantie E., Xie L., Tao H., Cheng J. (2021). G protein-coupled receptors: Structure- and function-based drug discovery. Signal Transduct. Target. Ther..

[25] Liu N., Li T., Wang Y., Liu S. (2021). G-protein coupled receptors (GPCRs) in insects—A potential target for new insecticide development. Molecules.

[26] Xu G., Teng Z.W., Gu G.X., Qi Y.X., Guo L., Xiao S., Wang F., Fang Q., Wang F., Song Q.S. (2020). Genome-wide characterization and transcriptomic analyses of neuropeptides and their receptors in an endoparasitoid wasp, *Pteromalus puparum*. Arch. Insect Biochem. Physiol..

[27] Yu K., Xiong S., Xu G., Ye X., Yao H., Wang F., Fang Q., Song Q., Ye G. (2020). Identification of Neuropeptides and Their Receptors in the Ectoparasitoid, *Habrobracon hebetor*. Front. Physiol..

[28] Hauser F., Neupert S., Williamson M., Predel R., Tanaka Y., Grimmelikhuijzen C.J. (2010). Genomics and peptidomics of neuropeptides and protein hormones present in the parasitic wasp *Nasonia vitripennis*. J. Proteome Res..

[29] Kang Z.W., Liu F.H., Liu X., Yu W.B., Tan X.L., Zhang S.Z., Tian H.G., Liu T.X. (2017). The potential coordination of the heat-shock proteins and antioxidant enzyme genes of *Aphidius gifuensis* in response to thermal stress. Front. Physiol..

[30] Pan M.Z., Liu T.X. (2014). Suitability of three aphid species for *Aphidius gifuensis* (Hymenoptera: Braconidae): Parasitoid performance varies with hosts of origin. Biol. Control.

[31] Yang S., Wei J.N., Yang S.Y., Kuang R.P. (2011). Current status and future trends of augmentative release of *Aphidius gifuensis* for control of Myzus persicae in China’s Yunnan province. J. Entomol. Res. Soc..

[32] Kang Z.W., Liu F.H., Tan X.L., Zhang Z.F., Zhu J.Y., Tian H.G., Liu T.X. (2018). Infection of powdery mildew reduces the fitness of grain aphids (*Sitobion avenae*) through restricted nutrition and induced defense response in wheat. Front. Plant Sci..

[33] Kang Z.W., Liu F.H., Zhang Z.F., Tian H.G., Liu T.X. (2018). Volatile *β*-Ocimene can regulate developmental performance of peach aphid *Myzus persicae* through activation of defense responses in Chinese cabbage *Brassica pekinensis*. Front. Plant Sci..

[34] Pan M.Z., Wei Y.Y., Wang F.R., Liu T.X. (2020). Influence of plant species on biological control effectiveness of *Myzus persicae* by *Aphidius gifuensis*. Crop. Prot..

[35] Pan M.Z., Cao H.H., Liu T.X. (2014). Effects of winter wheat cultivars on the life history traits and olfactory response of *Aphidius gifuensis*. BioControl.

[36] Pan M.Z., Liu T.X., Nansen C. (2018). Avoidance of parasitized host by female wasps of *Aphidius gifuensis* (Hymenoptera: Braconidae): The role of natal rearing effects and host availability?. Insect Sci..

[37] Fan J., Zhang Q., Xu Q., Xue W., Han Z., Sun J., Chen J. (2018). Differential expression analysis of olfactory genes based on a combination of sequencing platforms and behavioral investigations in *Aphidius gifuensis*. Front. Physiol..

[38] Li B., Du Z., Tian L., Zhang L., Huang Z., Wei S., Song F., Cai W., Yu Y., Yang H. (2020). Chromosome-level genome assembly of the aphid parasitoid *Aphidius gifuensis* using Oxford Nanopore sequencing and Hi-C technology. Mol. Ecol. Resour..

[39] Kang Z.W., Tian H.G., Liu F.H., Liu X., Jing X.F., Liu T.X. (2017). Identification and expression analysis of chemosensory receptor genes in an aphid endoparasitoid *Aphidius gifuensis*. Sci. Rep..

[40] Kang Z.W., Liu F.H., Pang R.P., Tian H.G., Liu T.X. (2018). Effect of sublethal doses of imidacloprid on the biological performance of aphid endoparasitoid *Aphidius gifuensis* (Hymenoptera: Aphidiidae) and influence on its related gene expression. Front. Physiol..

[41] Tasman K., Hidalgo S., Zhu B., Rands S.A., Hodge J.J.L. (2021). Neonicotinoids disrupt memory, circadian behaviour and sleep. Sci. Rep..

[42] Broeck J.V. (2001). Neuropeptides and their precursors in the fruitfly, *Drosophila melanogaster*. Peptides.

[43] Guindon S., Dufayard J.-F., Lefort V., Anisimova M., Hordijk W., Gascuel O. (2010). New algorithms and methods to estimate maximum-likelihood phylogenies: Assessing the performance of PhyML 3.0. Syst. Biol..

[44] Rozewicki J., Li S., Amada K.M., Standley D.M., Katoh K. (2019). MAFFT-DASH: Integrated protein sequence and structural alignment. Nucleic Acids Res..

[45] Letunic I., Bork P. (2019). Interactive Tree of Life (iTOL) v4: Recent updates and new developments. Nucleic Acids Res..

[46] Kang Z.W., Liu F.H., Xu Y.Y., Cheng J.H., Lin X.L., Jing X.F., Tian H.G., Liu T.X. (2020). Identification of candidate odorant-degrading enzyme genes in the antennal transcriptome of *Aphidius gifuensis*. Ѐntomol. Res..

[47] Hummon A.B., Richmond T.A., Verleyen P., Baggerman G., Huybrechts J., Ewing M.A., Vierstraete E., Rodriguez-Zas S.L., Schoofs L., Robinson G.E. (2006). From the genome to the proteome: Uncovering peptides in the *Apis* Brain. Science.

[48] Roller L., Yamanaka N., Watanabe K., Daubnerová I., Žitňan D., Kataoka H., Tanaka Y. (2008). The unique evolution of neuropeptide genes in the silkworm *Bombyx mori*. Insect Biochem. Mol. Biol..

[49] Li B., Predel R., Neupert S., Hauser F., Tanaka Y., Cazzamali G., Williamson M., Arakane Y., Verleyen P., Schoofs L. (2007). Genomics, transcriptomics, and peptidomics of neuropeptides and protein hormones in the red flour beetle *Tribolium castaneum*. Genome Res..

[50] Tanaka Y., Suetsugu Y., Yamamoto K., Noda H., Shinoda T. (2014). Transcriptome analysis of neuropeptides and G-protein coupled receptors (GPCRs) for neuropeptides in the brown planthopper *Nilaparvata lugens*. Peptides.

[51] Zhang H., Bai J., Huang S., Liu H., Lin J., Hou Y. (2020). Neuropeptides and G-Protein coupled receptors (GPCRs) in the red palm weevil *Rhynchophorus ferrugineus* Olivier (Coleoptera: Dryophthoridae). Front. Physiol..

[52] Roller L., Čižmár D., Bednár B., Žitňan D. (2016). Expression of RYamide in the nervous and endocrine system of *Bombyx mori*. Peptides.

[53] Collin C., Hauser F., Krogh-Meyer P., Hansen K.K., de Valdivia E.G., Williamson M., Grimmelikhuijzen C.J. (2011). Identification of the *Drosophila* and *Tribolium* receptors for the recently discovered insect RYamide neuropeptides. Biochem. Biophys. Res. Commun..

[54] Ida T., Takahashi T., Tominaga H., Sato T., Kume K., Ozaki M., Hiraguchi T., Maeda T., Shiotani H., Terajima S. (2011). Identification of the novel bioactive peptides dRYamide-1 and dRYamide-2, ligands for a neuropeptide Y-like receptor in *Drosophila*. Biochem. Biophys. Res. Commun..

[55] Song W., Veenstra J.A., Perrimon N. (2014). Control of lipid metabolism by tachykinin in *Drosophila*. Cell Rep..

[56] Van Loy T., Vandersmissen H.P., Poels J., Van Hiel B., Verlinden H., Broeck J.V. (2010). Tachykinin-related peptides and their receptors in invertebrates: A current view. Peptides.

[57] Gui S.H., Jiang H.B., Xu L., Pei Y.X., Liu X.Q., Smagghe G., Wang J.J. (2016). Role of a tachykinin-related peptide and its receptor in modulating the olfactory sensitivity in the oriental fruit fly, *Bactrocera dorsalis* (Hendel). Insect Biochem. Mol. Biol..

[58] Wang Y., Wu X., Wang Z., Chen T., Zhou S., Chen J., Pang L., Ye X., Shi M., Huang J. (2021). Symbiotic bracovirus of a parasite manipulates host lipid metabolism via tachykinin signaling. PLos Pathog..

[59] Qi Y.X., Jin M., Ni X.Y., Ye G.Y., Lee Y., Huang J. (2017). Characterization of three serotonin receptors from the small white butterfly, *Pieris rapae*. Insect Biochem. Mol. Biol..

[60] Gassias E., Durand N., Demondion E., Bourgeois T., Aguilar P., Bozzolan F., Debernard S. (2019). A critical role for Dop1-mediated dopaminergic signaling in the plasticity of behavioral and neuronal responses to sex pheromone in a moth. J. Exp. Biol..

[61] Finetti L., Pezzi M., Civolani S., Calò G., Scapoli C., Bernacchia G. (2021). Characterization of *Halyomorpha halys* TAR1 reveals its involvement in (E)-2-decenal pheromone perception. J. Exp. Biol..

[62] Ma Z., Guo X., Lei H., Li T., Hao S., Kang L. (2015). Octopamine and tyramine respectively regulate attractive and repulsive behavior in locust phase changes. Sci. Rep..

[63] Xu L., Li L., Yang P., Ma Z. (2016). Calmodulin as a downstream gene of octopamine-OAR α1 signalling mediates olfactory attraction in gregarious locusts. Insect Mol. Biol..

[64] Ravi P., Trivedi D., Hasan G. (2018). FMRFa receptor stimulated Ca2+ signals alter the activity of flight modulating central dopaminergic neurons in *Drosophila melanogaster*. PLoS Genet..

[65] Kiss B., Szlanka T., Zvara A., Zurovec M., Sery M., Kakaš S., Ramasz B., Hegedüs Z., Lukacsovich T., Puskás L. (2013). Selective elimination/RNAi silencing of FMRF-related peptides and their receptors decreases the locomotor activity in *Drosophila melanogaster*. Gen. Comp. Endocrinol..

[66] Krupp J.J., Billeter J.-C., Wong A., Choi H.C., Nitabach M.N., Levine J.D. (2013). Pigment-dispersing factor modulates pheromone production in clock cells that influence mating in *Drosophila*. Neuron.

[67] Ayub M., Hermiz M., Lange A.B., Orchard I. (2020). SIFamide influences feeding in the Chagas disease vector, *Rhodnius prolixus*. Front. Neurosci..

[68] Sellami A., Veenstra J.A. (2015). SIFamide acts on fruitless neurons to modulate sexual behavior in *Drosophila melanogaster*. Peptides.

[69] Corta E., Bakkali A., A Berrueta L., Gallo B., Vicente F. (1999). Kinetics and mechanism of amitraz hydrolysis in aqueous media by HPLC and GC-MS. Talanta.

[70] Finetti L., Roeder T., Calò G., Bernacchia G. (2021). The insect type 1 tyramine receptors: From structure to behavior. Insects.

[71] Meyer J.M., Ejendal K.F.K., Avramova L.V., Garland-Kuntz E.E., Giraldo-Calderón G.I., Brust T.F., Watts V.J., Hill C.A. (2012). A “genome-to-lead” approach for insecticide discovery: Pharmacological characterization and screening of *Aedes aegypti* D1-like dopamine receptors. PLos Negl. Trop. Dis..

