# Supplementary material for: Genome-Wide Identification of Neuropeptides and Their Receptors in an Aphid Endoparasitoid Wasp, Aphidius gifuensi
Source: Insects. 2021 Aug 18;12(8):745. doi: 10.3390/insects12080745 (PMC8397052; doi:10.3390/insects12080745)
Supplement: Supplementary file 1 [file insects-12-00745-s001.zip › Supplementary Files/Supplementary information.pdf]

Supplementary Information

Figure Legends

Figure S1. Functional annotation of *A. gifuensis* transcripts based on gene ontology (GO) categorization.

Figure S2. | Top 20 enriched Kyoto Encyclopedia of Genes and Genomics (KEGG) pathways of *A. gifuensis*.

Table Legends

Table S1. The amino acid sequences of neuropeptide receptors.

Table S2. Primers used in this study.

Table S3. Summary of head transcriptome.

Table S4. The amino acid sequences of neuropeptide precursors. Predicted signal peptides (highlighted in yellow), cleavage signals (red), putative bioactive mature peptides (light blue), amidation signals (pink), and cysteine residues (deep yellow) are indicated.
