# Supplementary material for: Genome-Wide Identification of Neuropeptides and Their Receptors in an Aphid Endoparasitoid Wasp, Aphidius gifuensi
Source: Insects. 2021 Aug 18;12(8):745. doi: 10.3390/insects12080745 (PMC8397052; doi:10.3390/insects12080745)
Supplement: Supplementary file 1 [file insects-12-00745-s001.zip › Supplementary Files/Table S1.pdf]

Table S1. The amino acid sequences of neuropeptide receptors.

The family A

>KAF7996862.1 hypothetical protein HCN44_002508 [*Aphidius gifuensis*]
MDNLLNKTTITRLIDTADLNITNCTIIDDNCNDNLIENIVAITVTIFFGSIGILGLAGNSLVVIV
VALNPGMRSTTNILIINLAVADLLFVIFCIPFTATDFVLNYWPFGNVWCKIVQYIIIVTACAS
VYTLVLMSLDRFLAVVHPIASMTVRTENHAFLATCIAWLVILILSIPVIIVHGEVPHHLQEDK
RVCRILPEVNWPLFQITFFLFSFLIPLTLISVLYICMLVRLWRSSRGSAESRRGRRRVTRLVLV
VVGVFAVCWCPLQVILVAKSIGYYPDNPMNIMLQITSHVLAYMNSCVNPILYAFLSDNFRK
AFRKIIYCKPRYDPKALGPSTKTTRAASSGDLL

>KAF7996860.1 hypothetical protein HCN44_002506 [*Aphidius gifuensis*]
MFFTYLLTVLLISFYNVDSSVIKKRADTSDYYDTDLCDSPIYCHGELLETIQLSQIFNDSKSF
VDLYQKHDPSVTLANFEKLMVENDRKPSRDQLRTFINDNFERSKEIMPWTPPDMYMYPPI
LERIADRDFRDWLTDLNYIWKTLARQMSPKVLDNPTRHSFIPVEHGFIVPGGRFEESYYW
DSYWTIEGLLLCGMHETAKGILLNFFSMIQRYGFIPNGGRVYYLMRSQPPLLIAMVEKYIE
ATNDVEFLVDNLHLLEKEFAFFQREKTVDVHKNGKIYRMARYFVKSLGPRPESYREDYM
SAQYFKTDEEKQTFYDEMKAGAESGWDFSSRWFINNHNDAAGNLTDISTRNIIPVDLNAF
LQRNARKLSKFNEYLENTFKAKQYKEIAVRYQEAINEVLWNEEAGIWFDYDLKNQKPRK
SFFPTNLTPLYTKSFDKSKSSIYASRAVEYLKTQGIGGFLGGVPTSLNPSGQQWDAPNAWPP
LQSIIVQGLYNTQCEPALTAAEELATRWTRSNYIGYKHYNQMFEKYDAAKPGEFGGGGEY
PVQEGFGWTNGVVFEFLDMYPNMTVSD

>KAF7994769.1 hypothetical protein HCN44_004241 [*Aphidius gifuensis*]
MLTTISTALELNSSYYTTVIGGSSTLEGFSVSSADPENATSTPYMLPAYIRTPSMVVCIAVMV
LGIVGNLMVPLVVLRGKDMRNSTNIFLVNLSVADLCVLLICAPTLLVEVNSGPQVWPLGE
HMCKAVPFVELTVAHASVLTILAISFERYYAICEPLRAGYICTKARATFLCFLAWILAAICTS
PILPLTSYTIDPDEQGIKYPNCITDLSQTWAITFVIITIIIFFIIPLFILIILYTVIAQHLMANPAISR
GPANNLLKYRKQVVMMLGTVVLCFFICILPFRAFTLWIIISPESFVINIGVEWYFCLLYISRN
MFYLNSAINPILYNLMSTKFRGGFLRLCGFSTIRERDTPTSGRTKTTTSNNSNNSSDWRRQS
SNKSCSVKVSKVSMKQFKFTSIKKNNINRKNNIESYV

>KAF7987302.1 hypothetical protein HCN44_003064 [*Aphidius gifuensis*]
MESTTEYWNTTKNNNFSSSSIIEEINVERLIGPRRDSLYIVLPITIIYLSIFLTGIIGNVSTCIVIS
RNKSMHTATNYYLFSLAISDLLLLIAGLPVEIYSVWSKYPYIFGEYFCILRGLFAECSTYASV
LTITAFTVERYFAICHPFLSHTMSKLSRAIKLILIIWFIALTFSIPQALQFGIVQHKKIHPDFIIC
TLKTIFIKHSFELSTLLFFIIPMTVITVLYIMIGLKLRKSNLMKNNNNVRSQRGGSNNKFHKE
NKHRGRSSRRVLKMLVCICIAFFICWCPFHIQRLIAIHGTRNDHVTSKNSWIEYLYIVFTYIS
GIFYYVSTTINPILYNIMSNKFREAFMETLASCGNNKSSEAPHEQRSYSSLSRSHQKTIGMF
GSRTIGGNGVAQDNSDCSGNSNNDDGSRQSTSAACQSKIETVKNNKSKNIKYNKIKKSKN
NIIENIDTNFDDKNDNKSSIISVKKSQIINDNNDNKNNVVKFLTNGKNNSNNCNKKWWRYI
KLLPTKKSFKLTTRSTNDNNNVDIQSNREEYSMTSCYVDGDHRLV

>KAF7991207.1 hypothetical protein HCN44_002769 [*Aphidius gifuensis*]
MNFELMKNVSIETNDLSSHINVSIDKLLINNNQVSDKHDALIIIVPITIIYIAIFITGLIGNISTC
VVIAKNRSMHTATNYYLFSLAISDLLLLISGLPPEMYYMWSHYPYIFGETFCIISSFAAETSA
NATVLTITAFTVERYVAICHPFLSHTMSKLSRAIKYVIFIWFFALCLAIPQAIQFGIIYEFDKN
GAKLPDSATCSVKWKILDHAFEISTIIFFVLPMTIITVLYILIGMNLRDSRILISVKRNNIPGSL
NNTESGRSRVASQRNIIRMLVFVTVAFGVCWAPFHAQRLLAVYAKSLGSQIETDLSTVYKI

VTYISGILYFLSTTVNPLLYNIMSNKFREAFKSMLAEHCGGRSLQTTSPRRLTYSSLSRYNN
KSIIKKQEKNSGSYSGSDETQRLTTIKRIDKIDKLNIENNDLPIIIKDNKNKNININKLSSSKIN
LVEPKVICHIVNDDNCEWNKNHLQTNDNCQNQITIVTSLAKSFNEGCHNNGGLSHAPVNL
CLNKSQRSHKTVSLGVLAERFRSGTKGLFTNQNRQQKSDNNNLQQQNVQTKIQHTLSIGS
ANTISNSSLQDLDETEFTGSELARYMGELNYDIIT

>KAF7990334.1 hypothetical protein HCN44_000139 [*Aphidius gifuensis*]

MNLYKQFDKINFTSDYDDGDDNSTTNISNIIKNCTNDYCISDEDYIDLIADYLKPTIYGWFL
ITMHTIVFFGGIIGNSLVCIAVYRNHSMRTVTNYFITNLAFADILVIFFCLPPTVLWDVTETW
FFGEKLCKIILYIQTVSVSVSILTLTFISIDRWYAICFPLEFKSTTSRAKKAILFIWIVSLIIDAPD
LFVLHVEPASHLKVDTIFYTQCETSWSRNSEKIFIYTKLIFFYFGPLTFMSFAYAQIIRVLWKS
EIPGYNCNVPPVTEITSSTMIHENLEGQLNSRRKAAKMLVAVVLVFGICYFPVHLLSILRLSS
IGLPSNDFTVAFSLIAHWLCYANSAINPIIYNFMSGKFRKEFKHSFYCAHGGSSYDRKFDGR
VVYQFPGTSQITGGRQRTLKFIFKNNNNNIEGHKSSEIIPLSEISHSRQSKSI

>KAF7992286.1 hypothetical protein HCN44_001611 [*Aphidius gifuensis*]

MNNSLLLINTTTIKTYEADVSDVTTNIIVQIIFYIFYGIIFVIGIFGNGLVCYVVIKNPHMQTV
TNSFITNLAISDILLCILAVPFTPLYTFIGRWIFGKLMCSLVPYIQGVSIYISTFTLTSIAIDRFFV
ICHPFRPRIKIKNCLLIIGSIWIASIILTFPYGIYMNIQELYYCEENWPSQKLRKIFGSFTSILQF
VLPFFSIAICYICVFVKLNNRSKYKPGFKSSKKEEIDKERTKKTNRMLIAMVTIFGCCWLPI
NTINIIDDFFADVSYIWKHYRLCFFISHCLAMSSTCYNPLLYAWLNDNFRMEFKKVLPCFD
SVTFNLTTKTTTTTCESKKNYNKNDSKHGNIIFSHIAEKSMIQESIQLDLRTTSTTPFQLEFK
NINQDILEKRKVTNCIVSKTFKNSFK

>KAF7993660.1 hypothetical protein HCN44_010255 [*Aphidius gifuensis*]

MIDSIKPFLFGSRISGSTCFEIVGNNLTTRKKEIIYSALIWIYMSVTIGIEMYINGFDLRSKELL
KKFRCSLGYITFTADIIITCYWKNDLTTALDHLRLYDLSSQFYDNTRSIYCRIVVIFIFCGWIS
VGYVTYYVEAASPIIVVIVYIHFYTRISMQIFTFFGFMLSLYQRFDHLIRIVLSNGLLKPGISV
ISGTWRHVKLQDVWCMHCSLSAAAAEINSTYSAQLFVWIVTISLNMLSRVYTLIITMSNE
DSTFRKIREIMWTIACLINLFLIVGSCHITSRQANLTPEAVFSPQSNIQRKPKLNKKNLNIAM
YFGIRKLQFTTVADFISINLPFLLSVKFEKMSELLNNFYNDMGHKADYVLIVLYVSIMLLAL
IANIMVILVVIKYQYMRSVTNYFVVNLSIADLLVSLICMPMAISQAVSIKWIYGETMCKLFY
YLQGVAVAASVFTITAMSIDRYLAIRSPIALRRVFNRKSTFMIIIALWLIALLIFSPVLSAVTLQ
SPDIHDLIDADNIQIFKPPVFKICSEDFKSLGIHAHHFGILCFIVVYTIPGFIVLTLYSLMGRTL
CARKPPFDCDSVAGSASSQQGFRLVRERKRVAWILLTLAIGFALCWLPYNALRLLLDLGIIY
EGGIIPTLLSYFLFLGHANSALNPVVYCFMTRNFRRSVAELICHGTSKFNHRKSHRNNVGG
VGVGSSVKNHGWNNRKNQKPYAVAALQHSTIKNKNKSFTSSSGYENSYDRHSPHRRCYM
LKTIKNNNKKNDTNRIDELADNINKKSTPHRIRSSYSDCGKTDKSVVFDGKRSQSLCVLSS
AVNYQVAYDCANQTTVVTNISQTGI

>KAF7994129.1 hypothetical protein HCN44_011398 [*Aphidius gifuensis*]

MADNDDLLDYEDEEQTEAVVDGSGDAPAKKEVKGTYVSIHSSGFRDFLLKPEILRAIVDC
GFEHPSEVQHECIPQAVLGMDILCQAKSGMGKTAVFVLATLQQLELTENQVYYVLVMCHTR
ELAFQISKEYERFSKYMPAVKVGVFFGGLPIQKDEEVLKNTCPHIVVGTPGRILALVRNKK
LNLKHLKHFILDECDKMLELLDMRRDVQEIFRSTPHGKQVMMFSATLSKDIRPVCKKFM
QDPMEVYVDDEAKLTLHGLQQHYVKLKENEKNKKLFELLDVLEFNQVVIFVKSVQRCM
ALAQLLTEQNFPAIGIHRGMTQEERLSRYQQFKDFQKRILVATNLFGRGMDIERVNIVFNY
DMPEDSDTYLHRVARAGRFGTKGLAITLVSDESDAKILNDVQERFDVNITELPEEIDLASYI

EGR
>KAF7995013.1 hypothetical protein HCN44_004485 [*Aphidius gifuensis*]
MDVLLVDLTSKILPMLQPQSPLLEMASLRLPDSEEFKVDRNHRTSINGQLITTRNPSQSSIEL
STTLSPLYQSNDVFIKTKDELHEYLKNSITDLVTSYNNNDDDTSAGLFNASTSDNIPIEHMP
NRFYRHSISMTAVYCIAYLFVFIVGLIGNSFVIAVVYRSPRMRTVTNFFIVNLAVADVLVIVF
CLPATLIGNILVPWVLGRFMCKTVSYIQGVSVAASVYSLVAVSLDRFLAIWWPLKCQITKR
RARMIIVVIWFIAMTTSSPWLMFFDLIKYGDDPDMRFCVERWPRPQDGTLFFIILALSKMN
NLTDIIFDGHSNITDSSIKLLKNIKKICLPRSNKITDASLTKLFENSPKIDYLCLRDTSVTAEF
VKKVADITMNRKRRTKLYSSAVDIWSLGCIFSEMATKRALFPGDSEIDQLFRIFRTLGTPDE
TTWPGVSQLPDYKSMFPQWDRMNLKEVVPNFDDDARDLFSKLLTYDPSTRITAMGALNH
PYFKDVKTILPILPKKD
>KAF7995563.1 hypothetical protein HCN44_006670 [*Aphidius gifuensis*]
MMAIKEYCFSIAAHRRMRTVTNYLLVNLSITDLMMTLLNCVFNFIFMLNSNWPFGVFYCT
INNFVSHVTVASSTFTLVVIAFDRYMAIMRPLQHHMSRRRTIVALILIWFTSSMLAMPCLLY
STTKTRRYGNGKKRIVCYMLWPDGDYLDSKSEYIYNLVILGVTYLIPMTVMAVCYTLMG
RKLWGSKSIGEHTQHQKDSMKSKRKVVKMFIIVFVLFAVCWLPYHGFFIFLHHYRSVMG
NSYVQHVYLSFYWLAMSNAMVNPIIYYWMNTRFRLYFQQIICRFCCMIRRQDTVSRQMH
ELTGFHRSEIGRSNSGRMKSTSIRWRHSTPDNHIQSFQMNTRSICEKLHHKRQDVAII
>KAF7998540.1 hypothetical protein HCN44_010948 [*Aphidius gifuensis*]
MVENSASKYLTTENMNNIFITKSNTSLMDTDATAELDQFYFYETEQFTVLWILFIVIVVGN
TAVLTGLQLGKRRKSRMDFFIKQLALADLFVGFISVLTDIVWRTTVAWVAGNIACKFVKFS
QVLVTYSSTYVLVALSIDRYDAITKPMNFSGSWKRARVLVVSAWGLSGILSIPILFLYEEKIV
QGKKQCWIDLDSPLKWRIYMTLVSFTLFLVPTIIISGCYIIIVATIWSQNNALRQGPMRDRR
ASSRGLIPRAKIKTVKMTFVIVFVFILCWSPYFTFDLLQVYGYIPKTQTNIALATFIQSLAPL
NSAANPIIYCLFSKSFFKTVR
>KAF7988142.1 hypothetical protein HCN44_007636 [*Aphidius gifuensis*]
MYEMISADKNVTIEEMLNFLYENMTNYANLSEEDYLLRQFGPKYLSLKLVIPITIVYVTIFV
MGIFGNVVTCWVIFRNPIMQTATNYYLFSLAVSDLMLLVLGLPFELRGFWQQYPWELGLG
LCKLRSYVSETSSYVSVLTIVAFSMERYLAICHPLHLYAMSGLKRPLRFIIVAWLVALLAALP
FAVYTKINYVEYPPGSGYYSEESAFCAMLLPNMPDFPLYELSCVIFFLIPLVLLIILYLRMTIV
IKSTTLGRSIEGTVHGETRQTQSRKIVIRMLSAVVFTFFLSWFPFHAQRLLYVYDRSSSLAD
VNEWLYVFSGFLYYISTAINPILYNVMSAKYRSAFVQTLCCKPTINHFNRDEQSSMKETTL
YRCGSCKSSQIARGRSKSVRYHLENSKEITIKYPDDESYNFQCERRCSNFESTSNHNQNTN
ESRKLSMIIRSNNRSNSRKNETAKLPGETHI
>KAF7988143.1 hypothetical protein HCN44_007637 [*Aphidius gifuensis*]
MDITSSEEVEFENFWDSLKNENLTEADYLTRILGPKQLPLRIIIPLTLAYVTIFLSGVIGNIITC
TVIIKNSIMHTATNYYLFSLAISDLILLILGLPNELSCVWQQYPWELGVALCKIRAYVSEMSS
YVSVLTIVAFSMERYLAICHPFRAHAMSGLKRPIIIISAAWIIAIISAIPFAIYTNVNYIEYPPDS
GNNSADSAICAMLLPDMPNFPLYELSCIVFFLIPMFVMLAVYIRMGLKIRESAKNKLNCDG
EYSAHLDSQQVQSRTSIIRMLSAVVFSFFLAWCPFHAQRLLYVYALEADYYPDLNEWLYIL
GGFLYYFSTTVNPILYNLMSLKYRKAFKDTVLCKKQFRRGTFSNVDDRKIICKCLTDVKSH
PKNSRCSVRCKINNDTFKKHESKIIMQNKIDHIDCTNDCDENFNAKLLPNQHLFVNQQSSN
ESTSANKISESSASSFL
>KAF7998145.1 hypothetical protein HCN44_009543 [*Aphidius gifuensis*]

MIRRTKMDKFLEAENTVHLKDEILLSPITKYLLPTLVLGMILISSVYVTMLVVNSSDDNWIS
SCEIQSTYYVNCLPGKKSNYEMCISIGCCWSDVLSFCYHSNPSSHGYTQLSSDEIYTLKPR
KKISPLGSKNRQQVFFNTSRINENHFKFSFNTNATIEEKKFINMNYKLNENLKNTEIGSSSN
LSIVINNPEFSITVYENNNSINIPIFTTSKGPLILTDNYWEMTLLLGEIVSIYGLNRLELNGTTS
WNYDTGSGNYIPAFVGINSKGFGIGCVIDYLGPLESEVLDKSNLIILRGMSLPDVINLHVFIE
ITPLDVVKQMSKLTKQLNPSKVTKQESKFELGLHVCPDNNKNIQNDIETTILNMNNYNVP
WNSHCVNSKFYQTLDETMNKQEAEILKNTSDKLIKINKIVIPHLSPLVSSSGKLLQQLSNM
SLLLLENSMYLKKFYVGMYNNDNIIYPNWFDDKTLVVYENYLLNYLNIFKQPNFIYVENS
WPIDETNYTFDFKNFDYMPKNLAKQMTNGTIPPGLLSSTSKYHYQHHNEYSMKFINFINK
YTIGRKIMKQNNIIGWTGLRETIIHGVGCGFVGQLPQPIYICGIFQTPAELCTRWYAAAVVF
PYIIAIPENLPGGNWLNKGTSKYASKLLQFRHSFLTYKNTLIHEYNSYGQPVLAPTFIHYPL
EEFTSHALDQFMWGESILVGLVITPGIMQVKMHIPGNLSWRHLQDGLEVQPTKENSISIVA
LESELITLVRPGYIVPLEKELSGTSTVKNSLILKANLACITDGNNFANGKIGYAVDTFLYINF
KNNNFTISNIINTKKQACIDSSTLPTVIHSFEILGDNIFEVDIDICQIIHDNYSVLLNK
>KAF7998144.1 hypothetical protein HCN44_009542 [*Aphidius gifuensis*]
MNMDINAMSTMTTNINNIDVAPANATLPPSLTFTNHSLTVVIIYCMCFIIAAVGNLTVFVTLS
RGRYRKSRISLMICHLSAADLVVTFIMIPVEVSWRLTVQWVAGNIACKVFSFLRAFGLYLS
SNILVCVSLDRYFAVLHPLRVTDAKRRGKIMLTIAWISSVIYATPQYFIFHVSTHPEFPNFHQ
CVTFESLDKKSENLYAIFCVLAMYFIPLIIICWAYTKILCEITSKSRGLDQVIPKSNSSNTCDS
TGTSTTGSRMRLRRSDMSCIERARSRTLKMTITIVAVFILCWTPYVGMLLWYTIDRPSAEK
VDSRIQEFLFLMAVSNSCANPLVYGSYAIDFRKECCRCFLPYPTASSSRRMEGAYELANRN
ANGGMIRVKKNPGVSSVICAVIPDILKGKDSKSTQYSGTALLPMNSDRQRISESLSSKKSLT
VV
>KAF7987375.1 hypothetical protein HCN44_003137 [*Aphidius gifuensis*]
MSINLRSVLISDPVDESCGALLASYGVPVTTKYKLPKDELIQEIQKHDGLIVRSETKVTAEI
LAAASNLRVVGRAGTGVDNIDLDAATRKGIVVLNTPGGNSISACELTCALISSLARNVSQA
AASMKEGRWDRKLYSGFELSDKTLAVLGFGRIGREVAHRMQSFGMKIVCFDPVLSQEAA
ASVGALKLNLDEIWPIADYITVHTPLIPQTRNLINATTLSKCKKGVKIINVARGGIVDEEAL
LNSIKSGHCGGAALDVFMEEPPKNKITLELIQHPSVIATPHLGASTAEAQQRVAVEIAEQFL
AISGKTDKYSVTGIVNAPILSAAMSGDNGPWIELSKKLGKLAARFVKKNINAKIESHIVGD
ELKNKKFIHTAVLVGVLSGQTKNGLNLVNASTLAKDIGIDIKELYVNGDNNYVVVKIGQY
EIRGTVRNNESYLLSIDDNVFNNGIVLRDFISIYHGNGPQDLATIVNEFTAKNININSFNASG
NWLAIETNQDVDIIIPGITSY
>KAF7995618.1 hypothetical protein HCN44_006725 [*Aphidius gifuensis*]
MSNDTNVTLDPFLDYDINESLNNYEWSELAPALVVYSITFCLGLTGNLVIIASTLCPKLRPL
PSTPTNIFLGGLASADLILITFCIPVKIAKLFSYTWTMGLFLCKSVHYMQSVSAICSVLTLTA
MSVERYYAIVHPMRAQYTCTISQARRIVMVTWAASFLLAIPMLFTQRHKAVGEKVPAFWC
VRDSDSVTIWRLHELYMLLLVLVIPLVVMAFCYTAICWEIWLVMKRRYHMTSRHALSPTV
NNNAVNGESIAMTDRRRDGDRSRRSRSRRGDARTEIESRTMKQVVKMLVAVVVLFAICWS
PMLIDNVLTSYGILPRLKHDTSKHLNTIFQLMAYFNSCINPIIYGFMSKHFRESFLAAACGG
WWCCCRRRVYTPPVKRHPSVSQTRTTSVRWA
>KAF7997530.1 hypothetical protein HCN44_006101 [*Aphidius gifuensis*]
MQFIFYYVELDELYDVPTGIIILLSICYGSISFLAVVGNSLVMWIVATSKRMQSVTNCFIANL
ALADIVIGLFSIPFQFQAALLQRWNLPHFMCPFCPFVQVVCVNVSVFTLTAIALDRHRAVL

KPLSARPSKLRAKIVIGGIWFISGTLAAPMAIASRVVFVPENPNVHYKPFCQNVNVSEGTM
LTYRALLGFLQYLTPLTIISCVYARMGFKLWGSKAPGNAQDSRDANLMKNKKKVIKMLVI
VVLLFAGCWLPLQMYNVLQYTFPSINEYRYINIIWFCCDWLAMSNSCYNPFIYGIYNEKFK
KEFQQRCPLRSRKWSASVPNDSIDLDKTLTTRTSFRYILRNDWRRQKSSTYLPSGSYYQGI
PMRDSIKVSCNGDDT

>KAF7998064.1 hypothetical protein HCN44_009462 [*Aphidius gifuensis*]
MEPTIVLMKNIENTTLTPELPIDMRFNEGHVVSIVTYSILMIISITGNTTVLYLILQRRRRNRS
RINTMLMHLAVADLLVALLMMPMEITWAATVSWPFGDAMCRIMAFFRVFGIYLSSFVLVC
ISVDRYYAVLKPLQLMDVDRRGKMMLLFAWIGSTLCSAPQMVVFHVEVHPNYTWYEQCI
TYNAFASDGHELMYSIFGMVMMYFFPLIIIIYTYSSILLEIYRKSRETLGPDRIRRSSLGFLG
RAKIRTLKMTIIIVFVFFVCWAPYYIMSVWYWIDRESAKKVDQRIQKGLFLFACTNSCMNP
IVYGLFNIRKTTQGSTQNTGTAGKKTSIENVVVFNSRRRDDKNLQKNETIQNQEYNLRRN
DVNNDIVNHNLHIYNKRNSKNRKILNNNNNTVSFCLIDTKLSLELIKKSFKKTENKNEIIII

>KAF7998063.1 hypothetical protein HCN44_009461 [*Aphidius gifuensis*]
MDHRVVALTVLFATFSIFSTFAIIHDDALTRNLEDALHDDQFAIIVSHLKLKKINYGTNTETL
FAVKDTEPKTKFLLMFNHQQNRVILETVENKINGREDLQIESLNITEPMKNIIFIIHQNEFNS
KIDVYVDCLNIGQLPLKKTLKDLMGQKIEDSIIQVFREKKIRTKIYRESRIRDAWEREGCEL
SLLDDFAYHMETTPANSIRVRRRGDIPSNVHTLDDLNCVGDQQLVKTLNELIEFTKRIWAE
LERNTQETRHVKKLIEDCSACKPEFFIATTPAPPRPSCNFQSPCYPGARCDDSDGTPRCLSC
PRGYDGDGITCNRIRTCSDNPCYPGVRCQDLGNGYRCSRCPAGFTGNGEHCEHLRGCDTN
PCHANVACHPQPDAPYYRCGQCPHGYNGNGISCQPVDECDLRNPCDPRLQCTNTPGISPG
YRCDPCPAGYIGNKTRCVDINECEDGQNAACVANSECINTEGSFRCGPCNYGFIGNQTFGC
QSINICQDRITICDKRADCICIGPNQYVCQCHVGWAGDGFTCGIDSDNDGHADLVDNCPSV
SNSGQEDADNDGTGDACDNDSDKDGVLNIPDNCPLTYNPQQQDTDNGGPDGVGDVCDN
CPKIPNPHQEDSDGDGVGDACDNDKDNDGILNHEDNCPSKKNEEQEDTDGDGIGDACDN
CIDVPNTNQNDEDGDGVGDDCDTGRDRDKDGVQDDIDNCPDIANAQQIDTDNDGYGNE
CDNDIDDDGIPNNIDNCILTYNPKQECTNGGIYGDACTIDYDQDKVPDRYDNCPNNSQIWS
TDFREYQTIALDPEGVAQTDPKWQIHHNGSEIVQTANSDPGIAIGKAKFTGVEFEGTFYVN
TDIDDDYVGFVFSYQSNRKFYAVMWKKTAQTYWRASPFRAVAEPGVQLKLVDSETGPGK
IMRNSLWHTGDRDNQVKLLWKDPKNIGWKEKTPYRWRLIHTPKIGLIRLWIHQGRNLIVD
SGNVYDSVLKGGQLGVFCFSQEMIIWSNLYYKCSDTVPAMVYDELSDENKRLVHKDSSG
YYNQSSRS

>KAF7996284.1 hypothetical protein HCN44_001916 [*Aphidius gifuensis*]
MDSMSSNVFFPPCDNFTNEINSWYNKSHGSIINDSMINKTCLEHAPILTKQAYFKAIVLAF
MAGLSLIANIATIWSIRKNKRKHHGCSAVYTLILHLSVADLFVTIFCIAGESFWSYTVAWPW
GNITCKLFKFLQMFSLYLSTFVLVLIGVDRFIAVRYPMKSLNTAQRCNRLVIITWIISFILSIPQ
MVVFHVAKGPFIEEFSQCVTHGTYTEPWQEQLYAILSLIFMFILPLIFLIVTYVSTVITIAQSG
KAFKPEVANAHNANHLTGDINRRRLIHRAKSKSLRISIVIVTAFVLWWTPYYTMMIICMFL
DPDEHLSKDMQSGIFFFGMSNSLVNPLIYGAFHLWPKKRRPGFRYRDGSTMQHRSATTNA
SFMLATRGGSTRLISNSTKKSDTNIQGNPHPIDETVLMKLIEKPKKINNEKTESKTKIILRFN
NTTILTN

>KAF7993395.1 hypothetical protein HCN44_007898 [*Aphidius gifuensis*]
MTIGISNLMYKQIAFVLGAVGLLGLIFNLLVIIVIIKDSSNSWTPTNVVLVNLAISDFLVAFFG
NPLAFISALNRGWYWSTSTCKWYACLMSTFGLASIGNLTVMALERWMLITNPTRPLSTRS

AMYFASFVWVYALVQSLPPLLGWGSYGPEGGNVSCSVSWEIHDSTTHNDSYIAFIFIFGLII
PLIIILSSYFGIIIKLKRVRQRIGSRSKRELKVTKMIGIMITAFLIAWMPYAIFAIAAQYFHFQP
SASIAILPSLLAKSSICYNPIIYAFLSSQFTQSFKHFFGIQDNAISRQQQDLSHGTWITLTTRIE
KV

>KAF7991526.1 hypothetical protein HCN44_008897 [*Aphidius gifuensis*]

MVNTTTTTTTIIPIIDERDEILAYWEICVLSIILVVTLIGNLLVLFSLWLRRYRGKRRKLTRMY
YFMMHLSIADLITGVFNVLPQLVWDITYRFQGGYGLCKIVKFMQPLGNYLSSYVLTATAID
RYHAICYPMSYCLTTSRQSRIMVHSAWCFSLFLCIPQVFVFSYREISTGIWDCWAEFYLPYS
QQIYVTWFSIAVFLLPFMVLVFTYVSICIGIWGNSEISKFKKRDELTRMDSNNNNNNNNNR
EPLITRAKIKTVKQMITVVCLYVITSSPFIGCQLWATWDPNAPQTPFFTGPIFAILVLLSSLTS
SVNPWIYLGFNKELRIIIKKFIFRNSENQNENEFLSSTRSSTIIGSTTRYNTTTKYQANKSSSA
AAHRNNHSAVVVIQTKS

>KAF7998503.1 hypothetical protein HCN44_010911 [*Aphidius gifuensis*]

MSTDTVFGILPVLNTNNSLEYEEDISDDALSLLSELFVGFLLFVLIFFSVAGNILVCVAIYTD
RGLRRIGNLFLASLAIADLFVGCLVMTFAGFNDLLGYWMFGPRFCDTWIAFDVMCSTASI
LNLCAISLDRYIHIKDPLRYGRWVTRRVAVASIASVWLLAATVSFVPISLGLHRSEDSKGPA
PTYNDGSQEYPTCALDLSPTYAVVSSCISFYVPCIVMISIYCRLYCYAQKHVKSIRAVTKIPQ
PSSIGKSFRARTTRKLQPSKIKSQTKTKPTSPYHVSDHKAAITVGVIMGVFLICWVPFFCVN
IVAAFCKTCIPDRAFQVLTWLGYSNSAFNPIIYSIFNTEFREAFKRILTKGARARGNQPSTSE
CGEFRSVIVQKRNGSIVECNISPRSSADSCQVNQVITGRQHRDTIVSSI

>KAF7993827.1 hypothetical protein HCN44_011096 [*Aphidius gifuensis*]

MKMMHHFMKNYSYILGTFNITADDLDYMNNYSAVIINTNNDPSVCFCGGYVRELALYYR
VYHGYVALIVCLFGVVANILNIVVLTRKDMAIAPINRILTNLATVDMLVMIEYIPFALYEYF
VLPEKQKFPYGWAVFVLFHMHFAQLLHTISIALTLLLAIWRYIAIRFPQYSHTWCTASRCRC
SIWCCFFFPIIACAPSYLVFGIHEKYIYENGKKDILYHVDAWSYSSNSSVLYQLNFWTLGVV
VKLLPCLILTGISCSLIKALYKAKGRKQVLRGYNPCSMTNDDKVSRRPSKSERRADRTTRM
LVAILLLFLITEIPQGVLGLMSGIYGDCFFRNCYHNFGEFMDILALLNGAINFILYCSMSRQF
RTTFSQLFKPRIMNKWHGTTQQTEIHSTYV

>KAF7992413.1 hypothetical protein HCN44_001738 [*Aphidius gifuensis*]

MTSQFDFNPMDLASVFRRNINETDTESEMSRFSPPLRMFATIVAIIILLTGLFGNLLTIIALSR
YPKIRNVAAAFIISLCVADFIFCLLVLPFDSIRFVNASWTNYRYLCMGVPFLRYGNIGVSLLS
VGAITINRFIMITHHSLYAKVYKKHWIALMIAMCYICSYGMQLPTLTGSWGKFDYDEKLG
TCSIIKDENGRSSKTFLFITAFIIPCIVIVCCYTKIFWVVHSSESRMRKHASPTIKSPHTPGRDT
REIKQRRSEWRITKMVLAIFLSFVACYLPITIVKVSDPDVNYPKFHVIGYLLLYFSSCVNPLI
YVIMNKQYRQAYANIISCSKIRATLTPHGSSAPGHNNIGQDYSKDYSKTMVSTVSIALNPVI
RKSDLQDD

>KAF7993769.1 hypothetical protein HCN44_010364 [*Aphidius gifuensis*]

MVSNDRVIKLSEKQRRKCAKQNSKTKSQKKKRIIESFKILQTTQKPIEDQNFTSCNESETK
VCDESTASMSAKQRRKKNTKREKKSKQQNEQYFSDEVYKSTDIKSSKNSTNFGTEKYVL
SESKTFEYLSGYPPIKKKKLEILTKYFNERLSEYFIDKLQTSSQKKITTYERPIQHPIHEIKNL
TGEKTKPIEYPVYEVQNLSKENLSDYKRSVEYPIFDVQNSSAEKTSKYSTCNNNITVEKNK
NTSGYQKSLEYPIYDVQVSSNGRIVKYERSVEYPIFDVQNSGTGKTSKYSTCDDNKTDEK
NKNTSGYQNSLEYPIYDVQVSSKEKILKYAGSVEYPMFDVQNSGAEKTSKYSTCNDNQT
DEKNQNTSGYQNSLEYPIYDVQVSRSVEYPMFDVQNSGAGKTSKYSTCNDNKTDEKNK

NTSGYQNLLEYPIYDVQVSSKEKILKIKWEIRHHDRRNCKPKIVLFKAKQKLLYCLKSAIG
TAMRKNFKGDNLRVKDVKGEENRQRLCDNNAAYQFLRNIRTSPGYAEKRGKHALAMVR
QLGLPHLFVTLGPNEFFSPELLLQLAINEARTTNKPPIVETLVDALNLSQAEKSRLIKNDPV
LCAEFHNRARAFYSYIFINVLIHVIKGGINRNANLIFQIDAEGIYFQNLEIIVQRFGAYNSFTP
EELEEKKEQYDIDREEYELEQVDVPLVLQKSNIKATLSLNGGEDESNFNIVPARCRAPSLGI
DIDMVSEFDPSSSDSGVVSRCAVVKPLKLRLCRPIFGRKAVEKIKRDPSNNVGIVGKCDIST
PIEIKPVKPRDPEREKRRIARKKEKRATLILGLIMGSFIACWLPFFFLYIIKPLIPSLTIPAQAFV
IAFWLGYINSALNPVIYTVFNKDFRRAFRRILYK
>KAF7995300.1 hypothetical protein HCN44_005960 [*Aphidius gifuensis*]
MATTQDPTTLDTTSKLSDLLGTSNHHFNFTSTSFVTPDWPDTSVAVGKGCFLGVIIIGAVFG
NLLVIVSVMRHRKLRIITNYFVVSLALADMLVAMFAMTFNLSVQVTGRWLFGYFMCDVW
NSLDVYFSTSSILHLMCISVDRYVAIVKPLKYPIYMTKKVVFYMLLACWFAPGIISFVPIFN
GWYTTIDNNNYRHMHPDICEFKVNEVYAILSSSISFWIPCTIMTLTYLAIFKEANRQEKQM
HSRMGNVMLLSHRPSRDLNNINGELNNSGSSKTLTLNEINTDHLHTPTKDKNLIKMKREH
KAARTLGIIMGTFILCWLPFFLWYVTTSLCGSHCSCPDIVVHIVFWIGYMNSALNPLIYAYF
NRDFREAFKNTLQCAFCSLCRREPFDLEALDIRRPSLRYESKFLLLYFHDYKILFLYIIALLT
LTKKL
>KAF7997959.1 hypothetical protein HCN44_009357 [*Aphidius gifuensis*]
MEEPSLEALMQAGLIFVVGVAIILSNLLIIATYLNFRGPSEVINCYLLSLASADLLCGLLIVPL
SVYPALVRRWVYGDIVCRLVGYLEVTLWAVSVYTFMWISVDRYLAIRKPLRYETVQTKTR
CQCWMVFTWISVAMMCCPPLLGFNKPIFDHEAFVCMLDWGNMAAYTITLSILVLGPSVIT
IVYTYGYIFSMMRKLKSGVPIHDKEYATALAENLSNPSHLMSFVLVMAFWLSWAPYAGLR
IYTSVNGGPPQIPFLQFAVVWIGITNGFWKAVILGTLSPQFRLAARVLCLTLCCRHRRLPPEL
LGLDDDD
>KAF7997869.1 hypothetical protein HCN44_009267 [*Aphidius gifuensis*]
MKKLLILLILIQVLGPSFVNACPAPCSCKNTGQQSDKIRVKCNKDIQDIKQIGFNIIAPDIYG
LDLSKNVIDVVEPYVFQNLTNLRRLNLAYNKISKLEKDCFFGLQNLERLNISNNQISSIDSM
VFSQLLNLKKLSLSFNKISTVETDLFHNLLAIDQLELNGNALKTLSERSFHVLRELRLVDLS
DNPWECDCRLYWLSNWNSSLYKLNPFPKCHSPQNFQGHYITELGLSADYSCQFTKPVVEI
KPDQNQVVFEGDTITLKCSVPSIPNDPSANLNWLWDPTMNIDNNINNEKNLINPLEKFSNI
KIENRRLSDNAIIDISLKIDPVKEEHNGKWNCHLLSNFGNISTAISIIVISDKTRYCPLTITTNN
KGVYAWPKTVIGWKVELPCEGFALVPLRASYECDENGNWKNLNTELCPFTSSITKSFEQFS
KVDLTLTKSTFLETAKRFKNHTSDVNKITDPIEIHFIVKTIENYLKYLTYEKELDILLINIISII
MKVPRLILKTAEKNYKACTRLIKSIENINEHKNSNILNNKNIALETIRIKQEYTYSSLSCTW
YSYNQLIEKNDSRILNCLENNKTINNFYIDNKIIEATIQLKFNFTKIETFNLTTTPQLMISMFS
DNNLFPFYTDNKTYEKYDITSGIIGSNIIGLNNNITKNLTEPIIVILKMLNPYDSNKRPILAIW
NINNNGSGEWSTKGCELKEALATLVLYHCDKLGYYGLLEDLSYLDEKPMIIGAKFKYSNP
AIYIGTLIIIICLTCTSVTYIICNTSIAMPKRSKHSIINTWIAIILLCFFYTTGIQQTEDIEICQNV
GLALHYLSLCCLLWMTVSAHIMYHRLSKSNIKTIPDDEIPEQTMPKPILGLYLCGWGISMII
CGISSAINHHEYYGYNYCFLSTGPAIVQLFIPSIILIIYMMILYLLIRCTIRNINNNNGQLSEGT
QATENVDLELLEPNENRVDDNSVNSTPTVSSEIEDIEHSQITQLKGHIIILILFIIIWLSGCITTI
QPFTNYLPNEELIFASIYALSSCGLGLFVLLFYGIARSDVRSQWTIMRCWLKRKKNNCCRT
RNVCDAHQTIPAQPLVQNLTLPILNTQATQVTSDTNSIASSRCYIDTSMRVSNIQLKTSDIGS
DTTLSINKNQNINLVVLHRQQYRSNNSVTTYTESAVAGGHSTCAEMFYNPHQSGVARKFF

KKQRRHIKHNNLGPRKQGDGGATSDNDSCISIPRPAQRNHDNQHLNNSKVNNTNIHVEIN
PVSDVKNINILSDSCGSISSIGDNNIPVRYVIGQEKIHINGKKLNNNNNLSNKNNIQQNIPLS
PIESDGDTKTEEEKLLKNASQQCSLEYSSEIDSVTQITSEKSDNNLPEIDETPETSDKKIDIDF
KCTSLNQINHNNNNNNNKNSKILLKRCEQSNSLSCLTNINHYNIENIQKNYRNSYNDLLSI
DTSSKNCEADSRASIDDLISEENSDINSRHYIESQQSETDENSFTSETLFNRDAPLFTTSLNNI
KRISSNSENINFDILHELKLPNLNQQVNNIINHDNNNHDYLVKEFNSLTDLTAINVSLGEIRH
LDINASIGVDYEDTNYTDTQIYNEDFVVDDDDDDNNVILLDENSDKKETSV
>KAF7991191.1 hypothetical protein HCN44_002753 [*Aphidius gifuensis*]
MRELNASTCNGLYEKVEWSGTSIIGTLVILALVDVMVILGNLLVILAVYHTTKLRNVTNMF
IVSLAVADLLVGVAVLPFSATLEVFEVWIFGDIWCSVWLAVDVWMCTASILNLCAISLDRY
LAVTRPVSYPQVKFISLNFTIYSIALGSFYIPMLVMMFFYWRIYNAAVSTTKAINRGFRTTK
SSKMFGSRFDEQRLTLRIHRGRGSVHNGGHNSTSPKSPESSNNPSGRRDKIKISVSYPSTET
LNTKCNTLERTPSKCSQVSVHYSNGQTQNQLSTRNTHLKVGGINRIGSTKKQNRRSSCES
QMTGDEVSLRELTPCNEGKPRVMKMGKRNIKAQVKRFRMETKAAKTLGIIVGGFIVCWL
PFFTMYLIRAFCAKCIHPTVFSVLFWLGYCNSAINPCIYALFSKDFRFAFKRIICNCLCNKNK
NSLRRGSDGSQLATRNDRSPSYTIRVPQHGVSIEDSDPDPCSDQNTHSQSDSR
>KAF7994648.1 hypothetical protein HCN44_004120 [*Aphidius gifuensis*]
MNMTCGPGLDSFHQEYAKVHGYASILVCIFGSIANTLNIAVLSRREMSSPTNAILTGLAVAD
LLVMLEYMPYASHMYLYHRSRRDTYTYGWSVFVLVHSLFTQVCHTISICLTVTLAIWRYI
AIAHPQKNREWCSFNRTIFAIIGAYVICPLICLPVYFTTAVTSKTELLNSHGNSINLTIYNDTI
YNETLWFVDLSETMKDNNLLEQLNIWMYSVVIKLIPCFALTILSLRLILALLEAKKRRKLLT
ASTVIKLSNNKTIDNGCDLKKNLNKKKTTRLLDKERQTDRTTRMLLAVLLLFLLTEFPQG
VLGLLSVILGPSFFRTCYVKLGEVMDILALINSAINFILYCAMSRQFRTTFQQLFCNWMPIS
HHDNNGHTMTATHTMTQVTQV
>KAF7998304.1 hypothetical protein HCN44_009702 [*Aphidius gifuensis*]
MNASGESAGTMTTVVDYDTGGCPSPENENGSTMEAWEAAAASLILGFLVLATVLGNVLV
ILSVFTYRPLRIVQNFFIVSLAVADLAVAILVEPFNVAYLVLGKWMFGIHLCKLWLTCDVLC
CTASILNLCAIALDRYWAITDPINYAQKRTLKRVLGTIAGVWILSGIISSPPLAGWNDWPEEI
DPGTPCQLTRRRGYVIYSSLGSFFIPLLLMSLVYLEIFLATRRRLRERARQSRLGAVTSTRHK
NDNDDAEESVSSETNHNERSTPRIHAKTSLVVIDDDAATEVTVSCHDTLKKSSSHPRRHGV
SGAGGGNGATTSTTVYQFIEERQRISLSKERRAARTLGVIMGVFVVCWLPFFLMYVIVPFC
TVCCPSDQMVYFITWLGYVNSALNPLIYTIFNLDYRRAFRRLLHIH
>KAF7997032.1 hypothetical protein HCN44_005309 [*Aphidius gifuensis*]
MNESEIFLLGWDESSILARDLNRTFYNSTYNKNVTIDDLWDLASDRVGLAIILILCSVATVF
GNSLVILAVFRERYLHTATNYFVTSLAFADCLVGLVVMPFSAIYEVLENRWLFSTDWCDIW
RSLDVLFSTASILNLCVISLDRYWAITDPFTYPTRMSRKRAALLIAIVWICSSAISFPAIAWW
RAVRTEAVPKDKCPFTEHLGYLIFSSTISFYLPLFVMVFTYYRIYRAAVIQTRSLKLGTKQV
MMASGELELTLRIHRGGATHTDPRNLFRTASSTPEDLQELDESITLHNNNNNNNGLTRIPSN
RINHKHHLGKNFSLSRKLAKFAKEKKAAKTLGIVMGVFIICWLPFFVVNLWSGFCSKCIW
QEEIVSAAVTWLGWINSGMNPVIYACWSRDFRREKKIVNQK
>KAF7998304.1 hypothetical protein HCN44_009702 [*Aphidius gifuensis*]
MNASGESAGTMTTVVDYDTGGCPSPENENGSTMEAWEAAAASLILGFLVLATVLGNVLV
ILSVFTYRPLRIVQNFFIVSLAVADLAVAILVEPFNVAYLVLGKWMFGIHLCKLWLTCDVLC
CTASILNLCAIALDRYWAITDPINYAQKRTLKRVLGTIAGVWILSGIISSPPLAGWNDWPEEI

DPGTPCQLTRRRGYVIYSSLGSFFIPLLLMSLVYLEIFLATRRRLRERARQSRLGAVTSTRHK
NDNDDAEESVSSETNHNERSTPRIHAKTSLVVIDDDAATEVTVSCHDTLKKSSSHPRRHGV
SGAGGGNGATTSTTVYQFIEERQRISLSKERRAARTLGVIMGVFVVCWLPFFLMYVIVPFC
TVCCPSDQMVYFITWLGYVNSALNPLIYTIFNLDYRRAFRRLLHIH

>KAF7997032.1 hypothetical protein HCN44_005309 [*Aphidius gifuensis*]

MNESEIFLLGWDESSILARDLNRTFYNSTYNKNVTIDDLWDLASDRVGLAIILILCSVATVF
GNSLVILAVFRERYLHTATNYFVTSLAFADCLVGLVVMPFSAIYEVLENRWLFSTDWCDIW
RSLDVLFSTASILNLCVISLDRYWAITDPFTYPTRMSRKRAALLIAIVWICSSAISFPAIAWW
RAVRTEAVPKDKCPFTEHLGYLIFSSTISFYLPLFVMVFTYYRIYRAAVIQTRSLKLGTKQV
MMASGELELTLRIHRGGATHTDPRNLFRTASSTPEDLQELDESITLHNNNNNNNGLTRIPSN
RINHKHHLGKNFSLSRKLAKFAKEKKAAKTLGIVMGVFIICWLPFFVVNLWSGFCSKCIW
QEEIVSAAVTWLGWINSGMNPVIYACWSRDFRREKKIVNQK

Other insects:

>Hheb026340.1 NPF

MDETRRQLEALPDKYFEKIAHILMDLRNDTVDLSKPHLRISFANNYAFFIFLYVVMITMGV
AMNIGMIYHIVRHKLYHDPTYAYLINLAISDVVKCIFVLPITLAVLMIHNWIFGKFLCFFLP
MLQDIPLHVSMMTYLLIASDRYRLVSDPGKPRIPAFVVALGAWFFAVCIVLPYAIYTSYLDL
TMYKKPTLHGFGICMVNLYDDIQQYMRCLFLFTYIAPLTITAYLYVKASRELQNQEEPMAV
AMFEARRKNSYSRHGSNTSNDITSFRDGKRESGSVMTGGTVGLSGLSANYDLYDAELDV
RKEQRTQKYLIFMVSVFAILLCPLMVLRLAKPALLETYENTGHFDITFIMFVWMAFASTVT
TPLFYASWQMSRPAKERLKGYFQFSTKRLPPVLEKGLRHHGNRHQSGLANVAYTPQARN
GSLSGSNGGEDYSRGNSTFHSPDLGNNVHRMNLVQ

>Hheb109890.1 CAPA

MKSKDLLSLNNTTTMEPVDTGGTEIFNFSFFFNSSEEEYLRLLLGPKHLPMRLVIPITVVYV
FIFVMGIFGNVVTCWVILRNPVMQTATNYYLFSLAVSDLMLLILGLPFELRVFWQQYPWE
LGWGLCKIRAYVSETSSYVSVLTIVAFSMERYLAICHPLHLYAMSGLKRPLRFIFAAWLLA
MIAALPFAAYTTVNYVEYPPGSGRNSEESAFCAMLLHNMPGFPLYELSCLIFFLVPLILIMV
LYIRMGLRIQNTTLGGSIEGTVHGETRQAQSRKIIIRMLSAVVVTFFICWAPFHAQRLLYVY
DRTSSFGDVNEWLYFLGGCLYYISTAINPILYNVMSVKYRSAFMETLCCTHGGNSLNRDD
QSSMKETTIYRCGSCKSSQIARGRSKSVRYHSENFRDLLHTKPPDNTNDNFRPERHLMNN
SNSLINNHETCLLESRKLSTVVHTSNGRAKCRTTDINGSPDETHI

>Hheb109910.1 CAPA

MDRMNLELLTSEEEREFEEFWDNLNNQNLTEEEYLTRVLGPKHLPAKLVIPLTLAYVTIFVS
GVVGNVATCFVIIKNSTMHSATNYYLFSLAISDLILLCLGLPNELSSIWQQYPWPLGLGLCK
IRAYVSEMSSYVSVLTIVAFSMERYLAICHPLRAYAINGPRRPIIIILAAWMIAIVSAIPFAIYM
KINYVEYPPGSDKNSADSAICAMLLPDMPHFPLYELSCVVFFFIPMLIILVVYTRMGLKIRA
STRNNAATRPGESSAHWDSRQVQSRKSIIRMLSEYLLMH

>Hheb023220.1 AstC

MNSTDFLDNPFNLTNTSCGVNVPIVAAVNQVLYSIVCIVGLLGNTLVIYVVLRFSNMKTVT
NIYIVNLAIADECFLIGIPFLVTTISLGYWPFGQTTFTLYSFIFGFAIPLTLILIFYVLVLRKLRT
VGPKNKSKERRRSHKKVTKLVLTVIAVYIACWLPYWVTQVTLIFTPPMQCQSMLTITIFLL
AGCLSYSNSAMNPILYAFLSENFKKSFLKACTCAAGNDINAALQIENSVFPKKNKQRGER
GQFNKMTTSVTSKPEMDDEEGERGLLICKSSTTNITMTSRSSIPMACDKDRNGGVENGIQ
ETLLSSGGERGLIICKRPNTDIIITSKSGITIAGDKESEIKNDSQEQLMSSTDKQNFIICQSSTM

TNKSGMPIEEREGGVENDNQEILLTEVURERERAYQS
>Hheb002560.1 Orphan
MTDSMSMAARIAEAANRTVIEGEMSRFPKPLRTFAAVVAILIMIVGLAGNLLTIVALCKYPK
VRNVAAAFIIRFIDSGWTDVRFLCVLVPFLRYGNVGVSLLCVAAITVNRYIMITHHNLYGR
VYKKHWIAAMILFCYVFSYGMQVPTLIGAWGKFDYDPNLETCSIIKDSNNRSSKTFLFVM
GFIVPCIVIVGCYAKIFWVVHSSESRMRKHASPTVKSPHTPGRDTREIKQRRSEWRITKMV
LAIFLSFVVCYLPITIVKVADPKVQYPAAHVMGYLLLYFASCVNPIIYVIMNKQYRQAYAG
VISCSRIRATLTPHGSSVPGQNNYGQGNHIVTVTKL
>Hheb005840.1 ACP
MKPKLAASANDPLDPKNEARLKDRAVLTPIIKYVIPTFLIATSVLAGVYYVTMLFLSFNENEP
GITTTCDIQLAFRIDCLPGLEAIYGDCMDAGCCWDESDKFCYHTLPSVHTYQAVKHENYW
ALHTKNKLSPLKSYNKPSLQATINKIDNGYVEIKLTTTPSIQSMRHEIEKGAPAASPSNGNS
TGHDSILDDLINVMIHEPTFGVTLTRVDNLTKAFPILTTSRGPLIITDHYWELSLYLGNSNTT
LYGLNSGELNSSINWIYNNKGGRVMSNILGITSKSWIVGCYIDSQGPMEIEVLPSNLIIVRGL
ALPKDLSLHVFIGKEPEEITKRFVALHNNQLEREPPIPESFGLHICPDDTPKEMLSDLDKVIK
TMDDYRAPWDTHCIYKKFRSTLDQKMSISDAEDLEDIRKKLEGIGRKIIHHISSMCSYGKS
SLPFELANATMLLENTYGPYVGSIDSESFVYPEWRDERIEEVYNSVIEEYLHEAPVSKSLYV
RDSWPRDDSNYTTAAFEKFDYLPKELRALMSSGTIPIDVHSNITSFHYKHHNEYAEKFDAF
VGKYAYPMNVEEPNGESIGGWAALKNTLKRGIASGPIGQLPPAIYVCNVTTMDEGNLCTR
WYGLAVAFPHILARPQNIPGGELLNPGTSKYVAQLLRLRTSFTLYQQSNIMAYFHNGASILS
PTHYHYPEDTATRYTPDQFMWGPSVLVGLVTSPNIYQLQMAIPGEEPWRHILGGLIVHPSK
VSISVLEGEIIALLRPGHIIPFHEETALTSMATSQRPLKLICNLACFDDKCNAQGKIFYHPNLY
IQVNVSKTEIYLKTYNDGLVDCNIQENITIDTVRFLGIVISKSSSGTYVSNGASISGSRLRLR
RSDMSSIERARSRTLKMTITIVAVFILCWTPYVAMLMWYTFDRQSAENVDPRLQDAFFIMA
VGNSCANPLVYGSYAIDFRKECCRCFLPYPTAPKIDALELTQRNTGKKVQVTKIPSPGVSSL
LVRSIRHAVPTYLRVGSLRTRSTGVSCNEVIPSSSPKLISESFSSKSLPLHEMKHEKSHNFLS
VPGDDVLKMSTSSGIVSVGQSA
>Hheb011130.1 CCAP
MTRILDNLYSNKTDGNFTQLVGDFNLSDFIIRNELPNGSLADVNATDIDPFYFYETEQFTVL
WLLFAVIVAGNVAVLAGLLLGKRRKSRMDFFIKQLALADLLVGLISVLTDIVWRSTVTWY
AGNIACKLIRFSQVVVTYSSTYVLVALSIDRYDAITRPMNFSGSWWRARVLIAAAWGLSVL
FSIPIIFLYEETVVEGKNQCWIELSSPVKWRVYMTLVSFTLFIAPTIIIGGCYTVIVATIWSQSS
VLRQGPLRDRRASSRGLIPRAKIKTVKMTFVIVFARSENFPSIASLPSNDALHDKDFVTVLQ
DQIKVMKARTTALYHENVHLKTQIHQERMTSKNLEERLDQMKEYKNIGECKNEWTNTE
MRPEEMTIKVPFTCHACSKTIVNDNDDDDDGDPMVFITKTELNNLEKDIKELRESLQLRE
NSWDGMVEREQNYSRQLTRLAQEIMTVNQLVENQSNDIENLSILLQAREGELKSAQKDII
GLQKLVVRLEKRNKALKENGGEKTMTEMNERDRKWIETIVRQVSTPRGRQKSKESYYST
PRSVKNSGRDETTTIIDT
>Hheb035150.1 TR
MRGTTNFFLANLAAADLCVGVFCVYQTLTNYLMNSWQLGDFLCKTYMFVHALSYTASI
MILVVVCIERYLAIVHPIRCRSMLTRGRLRAAVVIVWILAAVYASPRFIYVETINHKLNSGS
VDIICIANIRKHNKNVLDAVNLILLYLVPLFLMCCLYTRIALGLWKSGEAFGGPGLVARTRN
GRVHHIHASSKNVLRARRGVIRMLIAVVLMFAVCNLPQQARILWLHVDPNYDRGSDFSTIF
TVSTFLISYTNSCLNPLLYAFLSRNFRRAMRELFTCHNHNPSRAFGMGYVPGDAARLENG

HTANMPHSSVIRLSSVHDSPCTTHTIARQGTLVKTSNENKMSINLRSVLISDPVDESCGALL
ASHGVPVTTKYKLSKEELIREIQHHDGLIVRSETKVTGEIIAAATNLRVVGRAGTGVDNID
LLAATRSGIVVLNTPGGNSISACELTCALISALARNVAQAAQSMKEGRWDRKLYSGFELSG
KTLAVLGFGRIGREVALRMQSFGMKIVCFDPMLDPEVAASLGATKLTLDEIWPIADYITVH
TPLIPQTRNLINATSLGKCKRGVRIINVARGGIVDELALLDALKSGQCGGAGLDVFAEEPPK
NPTTLELIQHPKVIATPHLGASTAEAQQRVAVEIAEQFLAISGITDKYTVTGIVNAPILSAAM
TVENGPWIELSKKLGQLAARFLKKNMNAPIESHTVGAGLQNKKFIHTAVLVGILSGQTKN
GLNLINAATLAKDIGINVKEAHVDGEVDAVIIKIGNHQIKGTVRNNEALLLSVDDAMFNN
GIVLRDFISLYHANGPQDLVTIVNAFSSKGITINSLNANGNWLVIETDQNVTIPIQGIEAF
>Hheb039010.1 LK
GAQPRMNTSWTAGNGESDLIWESDSNYSDVYNDSSIFEDSDELYNVPTGIIFLLSLLYGSISI
LAVAGNSLVMWIVATSRRMQSVTNFFIANLALADIVIGILAIPFQFQAALLQRWNLPYFMC
AFCPFIQVLSVNVSVFTLTAIAVDRHRAILKPLSARPSKFCAKIIIACIWFLSGALAAPMAIAL
RVVLVPESSTGGRMHLKPFCQNVNLSEGSMITYRGLLGFFQYLTPLAIISCVYARMAFRLW
GSQAPGNAQYSRDANLMRNKKKVIKMLVIVVTLFAVCWLPLQTYNVLQSTCHRINEYKY
INIIWFCCDWLAMSNSCYNPFIYGFYNEKFKREFQQRYPFKSRKWSTSPPPGSLDIEKTMS
TRTSLSVANIITTQNPNNTFKYSVNIAEGIERSSFNN
>Hheb111350.1 Crz
MELEHAPTLTQHAIIKAIVLCVLVADLFVSVFCIMGDAMWSYTVNWPWGNVACKFFKFS
QMFSLYLSTFVLVLIGVDRFVAVRYPLTSFNYPGRCQQFVAIAWILAFILSIPQAKYRMQILG
EQYY
>Hheb063160.1 RFa
MADEKYFDNFTNDTNTTYDPYADYDIEDSFNHFDWEELAPVVVIYSITFCLGLVGNLVIIT
STLCPKLRPLPSTPTNIFLGGLASADLILILFCIPVKVAKLFSYSWTMGWFLCKGVHYMQS
VSAICSVLTLTAMSVERYYAIVHPMRAQYTCTISQARRIVVITWISSFFLAIPIIFVQRHKPVG
WRYPAFYCVRDDSQPTYWRAHELYMLLLVLVVPLVVMAFCYTAICWEIWLVMKRRYHM
TSRHALNPSMNNNNVTNGECIPMTDRRRSTERSRRARSRREDTTTDGESRTMKQVVKML
VAVVVLFAICWSPMLIDNVITSYGILSQSKQGTVKHLNTAFQLMAYFNSCINPIIYGFMSKH
FRESFLAAACGGWWCCFRRRVYTPPVKRHPSLSQTRTTSVSHFYLDNLLFSSQVRLIAPSF
SENSKNFIYHCLTTIQIKGGLDAALPVNIDHPFPLSLIVSNSLQKIKEQQQQKISSPNLNN
>Hheb073560.1 PK
MRQGLREDRSAIDTFPIGINKDLGGDSLNEISQSIHENYTSQLAQLMTDDEFNQTTPKRDA
LYIVVPITIIYFAIFLSGLIDLLLLVSGLPPEMYYIWSHFPYIFGEIFCIIQSFAAETSANATVLTI
TAFTVERYVAICHPFRSRTIPQLSRVVKYIIVIWVLALCLAIPQAIQFGITFSKTLNGTIIPDTA
TCSVKWVIIKHAFEISTILFFVVPMTIITVLYGLIGIKLISSRMPGADKRKRPEQSNSQDSSRS
GVPNEKNVLRMLVAVVVAFFICWAPFHAQRLLAVYAKSLGDGGSSLVTVYTTLTYISGIFY
YLSTTINPLLYNIMSNRFREAFKRMLAEHCGGRRSIEPASPRKRTYSDLSHGRGPVGKRPE
QNSGSFSASDETQHLTPLVRNDEIEQASSHQESSVLIAKDSIGRKQLSSDVTPKSRSNDSSD
SSQIIIVTSLAKGLDEACHNKGGLSHSTVNKCLKVQRPIKAVTLGLLAERLRSGTKGLFAH
QQRQTRSSKMRVDSTIQRVQQKMQSHPSIESANTISNSSLQDLDETEFTGSELAKYMGEIN
FELVT
>Hheb044860.1 AstC
MNSTDFLDNPFNLTNTSCGVNVPIVAAVNQVLYSIVCIVGLLGNTLVIYVVLRFSNMKTVT
NIYIVNLAIADECFLIGIPFLVTTISLGYWPFADRYIAVCHPITSPKMRTQCISLLVSVTAWFTS

ALFMVPIVLYAETKDFPNGGNCNIFWPNNYGGQTTFTLYSFIFGFAIPLTLILIFYVLVLRKL
RTVGPKNKSKERRRSHKKVTKLVLTVIAVYIACWLPYWVTQVTLIFTPPMQCQSMLTITIF
LLAGCLSYSNSAMNPILYAFLSENFKKSFLKACTCAAGNDINAALQIENSVFPKKNKQRGE
RGQFNKMTTSVTSKPEMDDEEGERGLLICKSSTTNITMTSRSSIPMACDKDRNGGVENGI
QETLLSSGGERGLIICKRPNTDIIITSKSGITIAGDKESEIKNDSQEQLMSSTDKQNFIICQSST
MTNKSGMPIEEREGGVENDKQEILLTEVURERERAYQS
>Hheb116660.1 AstA
MDEKISFNATRWIDWMDQIGQNSTNCTINESNCPDYDELLIKRVVKVVVPLFFSPIGILGLV
GNSLVVIVIALNPGMRSTTNILIINLAVADLLFVIFCIPFTAADFVLPYWPFGDLWCRMVQY
LIIVTACASVYTLVLMSLDRYLAVVHPVASMTVRTEHHAFLAICIVWFVILTASIPVLLIHGE
LTGAPEEEEGEKQKVVNAPKSPLLCSCDKGVGTWAIFDNDIADPFNVKILYSGLIQFVHEF
GRGEKSAWNCTPNENDKKKSKYMAIMFLKYIIYLPDRSVESIDVNSINMLRLHLFLFTTLL
AGFADDAHAASIFTHYNDEEHKGTDLCHSLVFCNSELLKTVQLANIFNDSKTFVDHYQLN
DPSVTLANFERLMSETNRKPSKDQIAKFVAENFANTNEVLPWNPPDWQPNPPILERIEDPNI
RDWVKQLNGIWKNLSRQMSPDVLKHPERHSFIPVEHGYIVPGGRFQELYYWDSYWGVE
GLLLSGMTQTARGIILNLLSMVERFGFVPNGGRIYYLMRSQPPLLIPMIEKYVEATGDIEFL
ADNLLTMEKEFAYFQREKTVDIVKDGKTYRMARYIVSSQGPRPESYREDYKLAQFFPEAQ
RNTLYEDLKAGAESGWDFSSRWFVTDGDKVGNLSNTSTRSIIAVDLNAFLQRNARLLAEF
NKLLGNRVKAREWMDVANAYQEAIDEVLWNEQFGIWLDYNIKNGQQRHHFYATNLTPLY
TKSFNASRAAYYAKRTVEYLKSQGIDDFMGGTPSSLSETGEQWDAPNAWAPLQSIIIQGLY
NTNAEPALSASKELATRWLRSNYLGFERYNQMFEKYDSGNPGHYGGGGEYIVQPGFEFT
NGVVFEFLDINAKKLSSTIPIFSE
>Hheb090620.1 Opsines
MSNFLSGGGARKRELKVTKMVALMVTAFLIAWTPYAAVAIATQYFHWQPPNSLGVLPSIL
AKSSICYNPIIYAGLNSQFPQSLRKLLGMKQSRTRSQGPASDVTMGINREKVFE
>PPU05513-RA AstC
MMSNVSRGLFDFADNASQQNITMPLDCDANMPIISLLHQILYSIICIVGLLGNTLVIYVVLR
FSKMQTVTNTYIVNLAIADECFLVGIPFLVTTMSLGVWTFGKFMCKAYMTSTSINQFTSSIF
LFIMSADRYIAVCHPISSPKLRTPFISKMVSLTAWVTSAIFMIPVFLYANTMETKDGQMSCNI
YWPDDHGGQTTFTLYSFVLGFAIPLTLIFIFYFLVIRKLQTVGPKNKSKEKKRSHRKVTRLV
LTVITVYIICWFPYWLTQMALIYTEPNQCQSRITITTFLLAGFLSYSNSAMNPILYAFLSDNF
KKSFLKACTCAAGKDVNATLHIENSVFPRRNKANAERIQSNKMVTSGNSKNDGEDEENE
RGLLISKTSTTTVTMTSRSNITVTSEVRDPTSQQQREKDALKNGTQLTLLTQV
>NV15867-RA AstC
MMSNISSGLFVFADNASQQNITMPLDCDANMPIISLLHQILYSIICIVGLLGNTLVIYVVLRF
SKMQTVTNTYIVNLAIADECFLVGIPFLVTTMSLGVWTFGKFMCKAYMTSTSINQFTSSIFL
FIMSADRYIAVCHPISSPKLRTPFISKMVSLTAWVTSAIFMIPVFLYANTMETKDGQMSCNIY
WPDDHGGQTTFTLYSFVLGFAIPLTLIFIFYFLVIRKLQTVGPKNKSKEKKRSHRKVTRLVL
TVITVYIICWFPYWLTQMALIYTEPNQCQSRITITTFLLAGFLSYSNSAMNPILYAFLSDNFK
KSFLKACTCAAGKDVNATLHIENSVFPRRNKANAERIQSNKMLTSGNSKNDGEDEENERG
LLISKTSTTTVTMTSRSNITVTSEVRDPTSQQQREKDALKNGTQLTLLTQV
>BmA1
MEIEEEIELYRQMNYSYDFNGTFNGTMGTCPIVNLPYVSIVTQVLYALVCIVGLLGNTLVIYV
VLRYSKMQTVTNMYIVNLAIADECFLIGIPFLITTMSLNKWPFGDYMCKTYMISTGINQFT

SSIFLCIMSADRYIAVCHPIAAPRLRTPCVSRIVSAAAWTASAAIMTPIFMYAKLVRIGNKLS
CNIVWPEQDFSQGQITFTLYSFALGFAAPLTLIFIFYCLVIRKLKTVGPKNKSKEKKRSHRK
VTKLVLTVIAVYVLCWLPYWAFQMALIYSPPSQCVNHITITVFLVAACFSYSNSAMNPILYA
FLSDNFKKSFLKACTCAAGKDVNATLHVENSVIPRRRARAQARAAEARGGFAAAVGGSR
SEASTAMTSRSMAASEVLPLEARPPTLTPLIAHNGLSHSRL
>DmCG7285 AstC
MTLTSLITPTEQLAVAPNGTTLHQLESVESESYPSINGTQNETMVTSVRPHLDHRNRPTQQ
NGSHYLEYDDDGPDCSYSYNFILKLITMILYALVCIIGLFGNTLVIYVVMRFSKMQTVTNIY
ILNLAIADECFLIGIPFLLYTMQVGNWPFGNYMCKAYMVSTSITSFTSSIFLLIMSADRYIAV
CHPISSPRYRTPFVSKLVSAFAWMTSVLLMLPVILFASTVQSSNGNVSCNIEWPDTQNSHTD
STFILYSLVLGFATPLTFILVFYCLVIRKLHTVGPKHKSKEKKRSHRKVTKLVLTVISAYIFCW
LPHWISQVALISSAPQRCASRLELAVFLACGCLSYSNSAMNPILYAFLSDNFKKSFMKACTC
AARKDVNAQLQLENSFFPKFGKGRQSERLLGGNGKGGAQRGALTKKKCLATRNNNAPM
ATTTTTTTTTTGTDAVTCLQPPVHQVPAEIQVGNPATVLVVNAETNNCKPPVLHTDL
>DmCG13702 AstC
MEGGWWRGGGGGGRLGGKAIMEGHSTPNGAAASHRNNSTRTNIATNGCAHSGILLFVLT
AMTLTSLITPTEQLAVAPNGTTLHQLESVESESYPSINGTQNETMVTSVRPHLDHRNRPTQ
QNGSHYLEYDDDGPDCSYSYNFILKLITMILYALVCIIGLFGNTLVIYVVMRFSKMQTVTNI
YILNLAIADECFLIGIPFLLYTMQVGNWPFGNYMCKAYMVSTSITSFTSSIFLLIMSADRYIA
VCHPISSPRYRTPFVSKLVSAFAWMTSVLLMLPVILFASTVQSSNGNVSCNIEWPDTQNSHT
DSTFILYSLVLGFATPLTFILVFYCLVIRKLHTVGPKHKSKEKKRSHRKVTKLVLTVISAYIFC
WLPHWISQVALISSAPQRCASRLELAVFLACGCLSYSNSAMNPILYAFLSDNFKKSFMKAC
TCAARKDVNAQLQLENSFFPKFGKGRQSERLLGGNGKGGAQRGALTKKKCLATRNNNA
PMATTTTTTTTTTGTDAVTCLQPPVHQVPAEIQVGNPATVLVVNAETNNCKPPVLHTDLX
DRAPSMPLETVVFIARR
>PPU06379-RA AstA
MLLTANLTSTALQQQPPIGCYEDELMEEELDFDQVLVQSIVQVVVPIFFGLIGILGLLGNSL
VVIVVAANPGMRSTTNILIINLAVADLLFVLFCIPFTATDFVLPYWPFGNAWCKVVQYLIIV
TACASVYTLVLMSLDRYLAVVHPIASMSVRTESHAFIAIWIVWIVILTSSIPVLIIHGEWDEN
QMLNRSEACALHLGIVEQHRQQLQQHESKDEEPPLELTPQIACRILPDANWPLFQVSFFLA
SYVVPLTLICGLYVCMLLRLWKGARVSAESRRGRKRVTRLVLVVVGVFAVSWCPIQVILVI
KSLDMYPLSSATIALQIASHVLAYTNSCVNPILYAFLSDNFRKAFRKIIYCRSRQDQHNRLG
PLTKTTRAGSSGDIFCRTTTLVVDDESDDEVVPADNTSSQGQSARIIES
>NV10415-RA AstA
MTELGWSANSSVSQAILLTANLTSTALQQQPPIGCYEDELMEEELDFDQVLVQSIVQVVVP
IFFGLIGILGLLGNSLVVIVVAANPGMRSTTNILIINLAVADLLFVLFCIPFTATDFVLPYWPF
GNAWCKVVQYLIIVTACASVYTLVLMSLDRYLAVVHPIASMSVRTESHAFIAIWIVWIVILT
SSIPVLIIHGEWDENQMLNRSEACALHLGIVEQHRQQLQQHESKNEEPPLELTPQIACRILP
DANWPLFQVSFFLASYVVPLTLICGLYVCMLLRLWKGARVSAESRRGRKRVTRLVLVVVG
VFAVSWCPIQVILVIKSLDMYPLSSATIALQIASHVLAYTNSCVNPILYAFLSDNFRKAFRKII
YCRSRQDQHNRLGPLTKTTRAGSSGDIFCRTTTLVVDESDEEVPSDNKSSQGQSARIIES
>BmBAR
MESTEDEFYTICLNLTAEDPSFGNCNYTTDFENGELLEKVVSRVVPIFFGFIGIVGLVGNAL
VVLVVAANPGMRSTTNLLIINLAVADLLFVIFCVPFTATDYVMPRWPFGDWWCKVVQYFI

VVTAHASVYTLVLMSLDRFMAVVHPIASMSIRTEKNALLAIACIWVVILTTAIPVGICHGER
EYSYFNRNHSSCVFLEERGYSKLGFQMSFFLSSYVIPLALISVLYMCMLTRLWKSAPGGRV
SAESRRGRKKVTRMVVVVVVVFAVCWCPIQIILLVKALNKYHITYFTVTAQIVSHVLAYM
NSCVNPVLYAFLSENFRVAFRKVMYCPPPYNDGFSGRPQATKTTRTGNGNSCHDIV
>DmCG2872 AstA
MAGHQSLALLLATLISSWPKASWGATGNGSIISVSNSSGNNYAFTSEHTDHSDHNANDSM
EYDAESVALERIVSTIVPVFFGIIGFAGLLGNGLVILVVVANQQMRSTTNLLIINLAVSDILFV
IFCVPFTATDYVLPEWPFGNVWCKFVQYMIVVTCHCSVYTLVLMSFDRFLAVVHPVTSMS
LRTERNATLAIMCAWITIVTTAIPVALSHSVRIYQYHGNAGTACVFSTEEEIWSLVGFQVSFF
LSSYVAPLTLICFLYMGMLARLWKSAPGCKPSAESRKGKRRVTRMVVVVVLAFAICWLPI
HVILVLKALNLYGGSHLSVIIQIISHVVAYTNSCINPILYAFLSDNFRKAFRKVVWCGSPPPL
MTNQQVTKTTRTATGNGTSNIEML
>DmCG10001 AstA
MENTTMLANISLNATRNEENITSFFTDEEWLAINGTLPWIVGFFFGVIAITGFFGNLLVILVV
VFNNNMRSTTNLMIVNLAAADLMFVILCIPFTATDYMVYYWPYGRFWCRSVQYLIVVTA
FASIYTLVLMSIDRFLAVVHPIRSRMMRTENITLIAIVTLWIVVLVVSVPVAFTHDVVVDYD
AKKNITYGMCTFTTNDFLGPRTYQVTFFISSYLLPLMIISGLYMRMIMRLWRQGTGVRMS
KESQRGRKRVTRLVVVVVIAFASLWLPVQLILLLKSLDVIETNTLTKLVIQVTAQTLAYSSS
CINPLLYAFLSENFRKAFYKGLQSNRLGMWTTTHQDVSSEKTTY
>PPU10178-RA PK
MLNESFVASEYRRELDWLNLTATLLQEPLVDSNEDDDSIARFLERDPLYIVLPISILYTLIFIT
GLVGNVSTCVVIARNKCMHTATNYYLFSLAISDLLLLISGLPPEIYYIWSNIYIFGETFCIIQS
FAAETSANATVLTITAFTVERYVAICHPFISHTMSKLSRAVKYVIVIWLLALCLAIPQAIQFGI
VYSKLANGTLLKDSAMCSVKWPFIHHAFEISTILFFVVPMTLITALYILIGVKLRTSRLLSTV
KRIPSGQGFGQSDSRSKNCSQRNVIRMLVAVVVAFFICWAPFHSQRLLAVYAENNKKDKH
KLAIVMPVYTALTYISGIFYYLSTTINPLLYNIMSNKFREAFKAMLSKHCGSSLQKSNPGRP
TYSSLSRYPRSTIHRVDPHQVSASLSVSEETQKLSPTAAECPVEIISCARLGYCSPADKNESN
EIVTENILCREFLNNEPVQSKCIKPECWLIT
>PPU07065-RA PK
MEEPFGNSSNSSNSSYLGEEYLVDPLKATFGPVRDQLYVVIPISIIYASIFVTGTVGNISTCIVI
ARNKSMHTATNYYLFSLAVSDLLLLIFGLPSEIYQVWYKYPYVFGEAFCILRGLAAETSTN
ASVLTITAFTAERYVAICHPFLSQTMSKLSRAVKLILFIWLIALVCAVPQALQFGIVSYGTPD
ALMCQYKRQILQFSFELSTFLFFIIPMTLIMVLYILIGLKLKKSTLMKRNCRQHHRRSESTR
VKVGSSRMDRHCRHSRSTRRVLKMLVAVVIAFFICWAPFHMQRLIAIYGKKNVYTLDRHY
WMEQIYLILTYVSGVLYYVSTTINPILYNIMSNKFREAFMETLARSCRMSRFVMPRERRSY
SSLSRSQQRNPATYPSRTTAVSGGTALAQDSTDCSGNSFREEHQDLRAGANIAEYSSSETPP
PLPPLIYGNSKQSSRKSVITIEIGASDTSLSIMSVGNAPGQPRGSGSAGMGSHFVELNRQTG
RNHSIGRELPSPGYDRCIRMTASPAQPLQQSTVVNCRNKPNQTTSSKKKWWRVLDWLPG
LKSIRTSRALTRPTQDNIIAELPKQPDDYFMQLHTFKAKDESCRPV
>NV14978-RA PK
MLNESFVASEYRRELGWWNVTATSLQEPLVDRREDDDDDSIARLLRRDPLYIVLPISILYTL
IFITGLVGNVSTCVVIARNKCMHTATNYYLFSLAISDLLLLISGLPPEIYYIWSNIYVFGETFC
VVQSFAAETSANATVLTITAFTVERYVAICHPFISHTMSKLSRAVKYVIAIWLLALCLAIPQA
IQFGIVYNKLPNGTLLKDSAMCSVKWRFIHHAFEISTILFFVVPMTLITALYILIGVKLRTSR

LLSTVKRIPSGQGLGQSDSRSKSCSQRNVIRMLVAVVVAFFICWAPFHSQRLLAVYAENNK
KDEDKLAIVKPVYTALTYISGIFYYLSTTINPLLYNIMSNKFREAFKAMLSKHCGSRLQKSN
PGRPTYSSLSRYPRSTIHRVDPQVSASLSVSEETQKLSPTAAECPVEIISCSRLGYCGSPAGK
NEPNEIVTENILCREFLNNEPVQSKCIKPECWLIT
>NV12532-RA PK
MEEPFGNSSNSSNFVGEEYLVDPLKATFGPVRDQLYVVIPISIIYASIFVTGTVGNISTCIVIA
RNKSMHTATNYYLFSLAVSDLLLLIFGLPSEIYQVWYKYPYVFGEAFCILRGLAAETSTNA
SVLTITAFTAERYVAICHPFLSQTMSKLSRAVKLILFIWLVALVCAVPQALQFGIVSYGTPEA
LMCQYKRQILQFSFELSTFLFFIIPMTLIMVLYILIGLKLKKSTLMKRNCRQHHRRSESTRV
KVGSSRMDRHCRHSRSTRRVLKMLVAVVIAFFICWAPFHMQRLIAIYGKKNVYTLDRHY
WMEQIYLILTYVSGVLYYVSTTINPILYNIMSNKFREAFMETLARSCRMSRFVMPRERRSY
SSLSRSQQRNPATCPSRTTAVSGGTALAQDSTDCSGNSFREEHQDLRAGANIAEYSSSETPP
PLPPLIYGSSKQSSRKSVITIEIGASDTSLSIMSVGNAPGQPRGSGNAGMGSHFVELNRQTG
RNHSIGKELPSPGYDRCIRMTGSPARPLQQSSVVNCRSKPSQTANSKKKWWRLLDWLPGL
KSIRTSRALTRPNQDNIIAELDQRLPKQPDDYFMQLHTFKAKDESCRPV
>BmA2
MMEPNNSEYENNSYITFFGPVGMAFYDNGQVLEDSLTVKIILSIILAVIMILSLIGNGCTCAV
IARNRSMRTPTNCYLFNLAITDLFMALFVPIDIYIIWIPEFYPLGEVGCRLHFVLWDCLSNCS
LLIITAFTVERYLVITRPFLRQKLSLNSRVFKLVGVIWFVSCSFCIPDLLYIDMIEEKKYVFCY
VAMSHIVSVFVAAEIFVFYVIPMTIIIILYILITIELKFKKKLRSSPASNGQQNRDKAVIMLAAV
ALSFFLFWSPYCYLRIMLIWPGVYEKHYNAWKIVNYLCYNSYASSALNPILYSLMSRKFRR
AFKDFFTRRKPDSTNRNDIAKASLSKNETKLELKM
>DmCG8784 PK2
MLQGVAITIANDSNDDGINQSFMAHVSPSPNQSPSIGVGIGIASSTMANPSESPEMLLLKND
KFLTHVAHLLNITTENLSNLLGSTNGTNASTMAADSPVDESLTLRTALTVCYALIFVAGVLG
NLITCIVISRNNFMHTATNFYLFNLAVSDLILLVSGIPQELYNLWYPDMYPFTDAMCIMGSV
LSEMAANATVLTITAFTVERYIAICHPFRQHTMSKLSRAIKFIFAIWLAAFLLALPQAMQFS
VVYQNEGYSCTMENDFYAHVFAVSGFIFFGGPMTAICVLYVLIGVKLKRSRLLQSLPRRTF
DANRGLNAQGRVIRMLVAVAVAFFLCWAPFHAQRLMAVYGLNLINIGISRDAFNDYFRILD
YTSGVLYFLSTCINPLLYNIMSHKFREAFKITLTRQFGLARNHHHQQSQHHQHNYSALLRQ
NGSMRLQPASCSVNNNALEPYGSYRVVQFRCRDANHQLSLQDSIRTTTTTTTINSNSMAA
GNGVGGGAGGGGGGRRLRKQELYGPGPGTAVPHRMLQAQVSQLSSLGDANSLLEAEVV
DRHYASGRAKRALLATKSGALLVTPPQSGDPSEVSQPATRLKLTRVISRRDEVANTSTPPFC
GSHSLPDPETCQSASVAGRSSRKFPWRKRRQKTEDPSSEGLTYGSPKSQ
>BmPBANR
MMADETVNMEMLENNLLNVTNVTDQSSAYSESYPLHLLVPLSVTYAVIFIVGILGNTSTC
VVIARNRSMHTATNFYLFSLAISDIILLVCGLPLELYRLWNPFTYPLGEAQCITIGLASETSA
NATVLTITAFTMERYIAICRPFMSHTMSKLSRAVRFIIAIWVFALCTAVPQAMQFGIVSYVEN
GQSMSACTVKGPGVHQVFVISSFVFFVVPMSVISVLYALIGLKLRTSRILHPVKKLSLDSNE
RPGAHTPYRNGSSQRRVIRMLVAVALSFFICWAPFHVQRLLAIYGKSLEHPSDTFYLVYIVL
TFLSGVLYFLSTAINPFLYNIMSNKFRNAFKMTLAAWCGRRGGPRMGRSYSALLASQRQR
AANGLTDPVRGPRRLRRLSTATTHLCDAPPRAQVSATKIAISP
>DmCG8795 PK2
MAVKMLPTNSSGVLATDLQLFHNEKFLLNLTQVLNISADNLTSLLQGLEPEELLPTVTPMT

PLSLLATLSVGYALIFIAGVLGNLITCIVISRNNFMHTATNFYLFNLAISDMILLCSGMPQDL
YNLWHPDNYPFSDSICILESVLSETAANATVLTITAFTVERYIAICHPFRQHTMSKLSRAVKF
IFAIWIAALLLALPQAIQFSVVMQGMGTSCTMKNDFFAHVFAVSGFLFFGGPMTAICVLYV
LIGVKLKRSRLLQALPRRCYDVNRGISAQTRVIRMLVAVAVAFFICWAPFHAQRLMAVYGS
TSGIESQWFNDVFSILDYTSGVLYFLSTCINPLLYNIMSHKFREAFKVTLARHFGLGGKNQ
GRGLPHTYSALRRNQTGSLRLHTTDSVRTTMTSMATTTTGLNGSANGSGNGTTTGQSVR
LNRVSLDSVQMQGQNRSRQDLFDNPRRMLQTQISQLSSVGDAHSLLEEDLQFPGEPLQRQ
PTMCSIDELTDDLAISRSRLKLTRITRPPGGVTGGVAGGSTTGAAGSGGVSGDESSGKVRK
AKVKVLKSSSPFKGLRTKFNWRARRKGSHKPHEKGATVNGGDTEERAAF
>BmDHR
MNSETINDTANASRPVDSTRVFGPQRDTLYIVLPITIIYTFIFVSGLLGNIFTCIVIVRNKNLH
TATNYYLFSLAISDLLLLVSGMPQEMYSIWSKWPYVFGHTFCVIRGLAAETSTNASVLTITL
FTIERYLAICHPFVSHKMSKLSRAVKHVVLLWVAALALALPQALQFGIRQYQGVIMCLQT
RVIIEHSFEISTFLFFLAPMVLITVLYSFIGLKLREKSNVKEQNQNDFESSIRYSHKMCRKPS
QSTRRVIKMLVAVVVAFFICWAPFHAQRLVAIYGTNENHLAKSPILFSVYLFLTYISGIFYYM
STCINPILYHIMSNKFRDAFKMTLCCCGTRNDTAVKRSSYTAMAFVRHPTSSGTSNSGNSIR
NETNLQSKTRRTNGRDKILNDAHVCRNGTLSSAAVGKPDSRTNGDRPLDRNLINETYFNT
NC
>DmCG9918 PK1
MSAGNMSHDLGPPRDPLAIVIPVTVVYSLIFITGVVGNISTCIVIKKNRSMHTATNYYLFSL
AISDFLLLLSGVPQEVSYIWSKYPYVFGEYICIGRGLLAETSANATVLTITAFTVERYIAICHP
FLGQAMSKLSRAIRIIVLVWIMAIVTAIPQAAQFGIEHYSGVEQCGIVRVIVKHSFQLSTFIFF
LAPMSIILVLYLLIGVHLYRSTLVEGPASVARRQQLKSVPSDTILYRYGGSGTAMSFNGGGS
GAGTAGLMGGSGAQLSSVRGRLNHYGTRRVLRMLVAVVVCFFLCWAPFHAQRLIAIYAPA
RGAKLRDQHEFVYTVMTYVSGVLYYLSTCINPLLYNIMSHKFREAFKAVLFGKKVSKGSL
NSRNNIESRRLRRALTNSSQTQRFSIESAEQPKPSIMQNPTNKPPVAAQYAMIGVQVN
>BmA25
MDTGSFLRGNETYDEFFERCNNLSRFDCTEEEMLWWLMGSRRLPLREIIPISIVLVVIFLTG
VIGNVCVCVVIVKHPGLHTATNYYLFSLAISDLLLLMFGLPNDLSVYWHQYPYSLGLVFC
KLRALISEAATYVSVLTISAFSLERYLAICHPLHVYAMAGLTRASRIILILWIISIVCASPFAVY
TDITYRDYPPNSGNISVDSAFCALMASSPLLELSSIFFFFIPAVLILCLYVRMGLHIRSTRLTE
KTKLGLLNGHVHGETRQAKSRKAIIRMLAAVVIAFFVCWAPFHVQRVFYVYGYSLPHYH
VINEHLFNVAGALYYVSATVNPILYNVMSGR
>BmA27
MVEFTRENETVSELISRCTNISHFDCTEDAMLWVMMGPRRLPLQKIVPISVLLLVIFVTGV
VGNLTVCVVIVRHPTMHTATNYYLFSLALSDLLLLLFGLPNDLSVSWHQYPYSLGIVFCKL
RALISEAASYGSVLTIVAFSLERYLAICHPLHLYAMAGLRRALRVVAALWLLSFVAAAPFAS
YTTVSYHDYPPGSGNSSLESAFCAMLEVPSWYLYELSSLLFFILPGLIILCLYVRMGLRIRST
HTSKPGSPGTLNGVNGSVHGEARQAQSKKTIIRMLAAVVIAFFVCWAPFHFQRLFYIYGTG
ASHYHIINEYLFYVAGAFYYVSATVNPILYNVMSHRYRIAFKETLFCKKATRIRSKYIEQSS
TRETVVHNGRRTRSKYRNERKNCSYYVTETSLCSEWKKDFYQQKKMHVLYKERSGSEL
CSENEASQLMFGYLPGEENDDT
>DmCG14575 CAPA
MNSSTDPTFSELNASFTNTPDTLFATSVSSDPSHGFGEEDYACGTFNCSPKEFVAFVLGPQT

LPLYKAVLITIIFGGIFITGVVGNLLVCIVIIRHSAMHTATNYYLFSLAVSDLLYLLFGLPTEVF
LYWHQYPDLFGMPFCKIRAFISEACTYVSVFTIVAFSMERFLAICHPLHLYAMVGFKRAIRII
TALWIVSFISAIPFGLLSDIQYLNYPLDHSRIEESAFCSMSPKIVNEIPVFEVSFCIFFVIPMILII
LLYGRMGAKIRSRTNQKLGVQQGTNNRETRNSQMRKKTVIRMLAAVVITFFVCWFPFHL
QRLIFLYAKNMDNYLDINEALFSIAGFAYYVSCTVNPIVYSVMSRRYRVAFRELLCGKAVG
AYYNSGFARDHSSFRESSAYDRVHSVHVRASQHPNKFETDSSSANRVLIKKTYSLPLPKNA
DSTVLSTTDIVIVLENSHTVCEEPKVENDIWIENEETCI
>PPU09239-RA AT
MESLVVTALTFLATSASGETGEAEDDSANDALDPLSNCTNNLCISEDEYLDEMHAYIYPKS
YEWVLIVLHCIVFIVGLVGNALVCLAVYRNHTMRTVTNYFIVNLAVADLLVIIICLPPTILW
DITETWFLGLMPCKIVLYLQTVSVTVSVLTLTFISIDRWYAICFPLRFKSTTSRAKTAIIIIWV
MALLFDIPDLLVFYTHQNRKLHGKTILFTQCLPSWSRENQIAFNIIKLILLYTGPLMFMSFA
YCQIVRVLWRNDIPGHNLSTRIINANDLSSQSNVGNPEGQLKSRRKAAKMLVAVVLMFAV
CCFPVHLLNILRSSIEIRSSDLVNITSCLVHWLYYANSAINPLIYNFMSGKFRREFKRTFCCPR
GGGSHNRAVYRMAARKSSHSAPSARGLSSRVIIIRSSDKAI
>NV13423-RA AT
MESLVVTALTFLATSASGETSEAEDDSANDSLDPASNCTNNLCISEDEYLDEMHAYIYPKS
YEWVLIVLHCIVFIVGLVGNALVCLAVYRNHTMRTVTNYFIVNLAVADLLVIIICLPPTILW
DITETWFLGLMPCKIVLYLQTVSVSVSVLTLTFISIDRWYAICFPLRFKSTTSRAKTAIIIIWV
MALLFDIPDLLVFYTHQDRKLHGKTILFTQCLPSWSRENQIAFNIIKLILLYTGPLMFMSFA
YCQIVRVLWRNDIPGHNLSTRIINANDLSSQSNVGNPEGQLKSRRKAAKMLVAVVLMFAV
CCFPVHLLNILRSSIVIRSSDLVNITSCLVHWLYYANSAINPLIYNFMSGKFRREFKRTFCCP
RGGGSHNRAVYRMAARKSSHSAPSARGLSSRVIIIRSSDKAI
>BmA5
MALRKESLAIITMLIICNYVLSSNFDSIPESIRVRKSVDNTTSRSSLKNLNETMKQSNNETEF
GRLLDATEMTTEYDNFTEEPCVGDRAFCNLTREEYMEMLNDYVFPQPYEWVLIATHAIVF
VIGLIGNALVCIAVYRNHSMRTVTNYFIVNLAVADFMVILICLPPTVLWDVTETWFFGTAM
CRIVLYFQSVSVTVSVLTLTFISVDRWYAICFPLKFKSTTGRAKTAILIIWLLSLLFNIPEFVV
LQVQTKMQLRFNVQYFMQCASTWSDESDLTWHIIKALFLYTFPLLLMTIAYCQIVRVLWR
SDNIPGHTESHKLCSTQTGQSNWLAASRRTTPSIHTNASTEGQLRSRRKAAKMLVAVVAM
FAVCYFPVHLLSVLRVAFDVQQTDVMTCIALISHVMCYANSAVNPLIYNFMSGKFRREFHR
SYFKCFCCCHTTPAPEQNGASFEPIGSSRARTIRTTVRRHDSCVSYRLAHLSPSNHNIHRDY
IQNTNTSFIEPMNGNRRSKIRDESISDTATRFTVTTDIPCKD
>BmA16
MTTVEDDLNVPKKMKANKIISEHDDRFKTDTNSSEFEEAENETCVGDPQYCNMTKEEYV
KMIQEYIYPNPYEWILIATHTFVFITGLFGNALVCVAVYRNHSMRTVTNYFIVNLAVADFM
VILFCLPATVLWDVTETWFLGEGLCKVLPYFQSVSVTVSVLTLTFISVDRWYAICFPLKFKS
TTGRAKTAILIIWLVSLCFNIPELVVLKLVRFVPLRFELPYLLQCYGTWSPSSELVWHILKVL
LIYTLPLVLMAVAYHQIARVLWSSNGIPGQADTKKLATAELTQLRSRRKAAKMLVSVVIMF
AVCYFPVHLLSVMRYTIDMGQTEFITIWALVSHVMCYANSAINPLIYNLMSDKFRREFRRA
FCCSTSPGQQDFTSMSRVTTKKDSSIMASFKPGHTSTTFVHNNKNGHMT
>PPU12514-RA ETH
MTIFNDTMYYEGDDVTIFASALVTNTPKPVNDFYQLPIYMQVLSVLICVIVMVIGIIGNLM
VLIVILGAKDMRNSTNIFLVNLSIADLCLLLVCTPAILVEVNAGPEVWVLGEHMCKAIPFIE

STIAHASVLTILAISFERYYAICKPLQANYVCTKSRATMICILNWIIAGFCTSPFLLMVTYKL
EVDARGTLVPICGTEALTQWSIVYIATTIGAFFVVPVIVLMMLYSVIVYRLIKRSAIKHEMN
RHALHNRNQVIRMLCTVISAFFICLLPFRAMMIWVIDHVLSELGAESDFLRAHVNQV
>NV14019-RA ETH
MTIFNDTTYYEGDEVTIFASTLVTNTPKPVNDFYQLPIYMQVLSVLICVIVMVIGIIGNLMV
LIVILGAKDMRNSTNIFLVNLSIADLCLLLVCTPAILVEVNAGPEVWVLGEHMCKAIPFIEST
IAHASVLTILAISFERYYAICKPLQANYVCTKSRATMICILDWIIAGFCTSPFLLMVTYKLEV
DARGTLVPICATEALAQWSIVYIATTIGAFFVVPVIVLAMLYSVIVYRLVKRSAIKHEMNRH
ALHNRNQVIRMLCTVISAFFICLLPFRAMMIWVIVSPLEELANFGAEGYYCLLYFSRIMFY
LNSALNPIFYALMSTKFKNGFLKILKAAFS
>BmA6-A
MISTINYTQSQTNVNILHVAYSSYGNDIENVTEYRTKSEAAVDLDDAFRNGSLTNTTIGYTN
NNFTEYAEIPHYIKITSMTFCIAIMCLGVIGNVMVPIVILKTKDMRNSTNIFLVNLSIADLMV
LLVCTPTVLVEVNSKPETWVLGKELCLAVPFVELTVTHASVLTILAISFERYYAICEPLRAG
YVCTKTRATLICGLVWFFAALFTSPILAIADHKATSVNGTVVNQCLTQAGTVWEITFFVTIII
LLYLLPLIILIVLYSIIAKNLITAASKVVMNKTVDPYNARARKQVILMLGTVVLCFFLCLMP
YRALTLWIIITPSGFDGISSEKWYNILYFSRVMLYINSAINPILYNLMSSKFRIGFCKVCICYK
KENDLNRRTQRTITNGSTTSSSLTRTTNSLKKFFGHRTSVDRSEAETNSKDEERSLFDRIFPN
RAFLRQQSAPVCSNLNPNRINRMRSEGCMDINRPDNIHSNLNPKVIRSEIDADLPRANSLR
RNVLINTAKAKSVDSERNVMNYSKKTKVDSVVAFQKSKSVDYEFPESFV
>BmA6-B
MISTINYTQSQTNVNILHVAYSSYGNDIENVTEYRTKSEAAVDLDDAFRNGSLTNTTIGYTN
NNFTEYAEIPHYIKITSMTFCIAIMCLGVIGNVMVPIVILKTKDMRNSTNIFLVNLSIADLMV
LLVCTPTVLVEVNSKPETWVLGKELCLAVPFVELTVTHASVLTILAISFERYYAICEPLRAG
YVCTKTRATLICGLVWFFAALFTSPILAVATFTYEQDEDGTEVPVCLTQADTFWSALFFILTI
AIFFIVPLGVLLVLYSVIAKNLMENPVIIAQSSKNTSGTGNVIRYRKQVILMLGTVVLSFFIC
LLPFKALTLWIIVFPPETIMSLGIDGYYILLYFCRVMLYLNSAINPILYNLMSSKFRDGFVKL
LKINKLMRCSRNLRETMQRRDTFNTTTSTGFSSSQNTSDSFWRRYSNRVSSQKNILNNSKK
IKEEKVNPIKIGEIINVENTRRNSMKFIAALNEDAQIDNEVEIADNENNKQIQILNLDVKTNS
VYSITLDVSKEGKNRFVCVPAQDRDNKNIFIYDYNTKESFV
>DmCG5911-B ETH
MLPQIPSYIRTTAMFFCIVIMLLGVVGNVMVPIVIVKTKDMRNSTNIFLTNLSIADLLVLLVC
TPTVLVEVNTRPETWVLGHEMCKAVPFVELTVAHASVLTILAISFERYYAICEPLKAGYVC
TKGRAILICVLAWGIAALFTSPIIAISTYSVEPYGDGTDAPVCTTAADGFWSIFYFVGCITVF
FFLPFGILVLLYAAIAYKLLRPNNAFHRPTSPQPQQPSGGATSGSSQVPSTKGNSHQQSNGM
RKHRKQVIFMLVAVVSSFFVCLLPFRAFTLWVILASAEDVEGLGIAGYYNLLYFSRFMLYL
NSAMNPILYNLMSSKFRSGFWRLLLTCLGQRPHHHHRHHYHQRQHPTAGGSGRNASTRQ
EQDAEEGAALAGTTSARHPRRTLRREATFLINSISTSSGTDRTTSSSAWRSNSLSISGLSERE
RGILGAAIIGTTAATVTTACLQERRASKI
>DmCG5911-A ETH
MLPQIPSYIRTTAMFFCIVIMLLGVVGNVMVPIVIVKTKDMRNSTNIFLTNLSIADLLVLLVC
TPTVLVEVNTRPETWVLGHEMCKAVPFVELTVAHASVLTILAISFERYYAICEPLKAGYVC
TKGRAILICVLAWGIAALFTSPILWVAEYKLAEYIDGSSVAVCLTQAISDWTLAFFLMTISVF
FVVPFVTLVVLYGIIARNLVSNRAAMLRARPTKPELSLKARKQVVLMLGAVVLSFFVCLLP

FRVLTLWIILSTDQTLHDLGLVRYYSLLYFCRIMLYLNSAMNPILYNLMSTKFRRGFKRLCQ
DAGRLLLELVTLGRRKEDSSRGRRGTLSLGMGTNTNTNTNSSNATGATSSSILSRSSNRRC
SEDISRTRLKIEMQMPCGSDLEAMAMLQHSTLGKGIARRVSDSRLMPLRNHQPRRHKPQI
SFDEESLEENKRSEAKIPTKCREKLPGIAREIVNLTENTL
>PPU01679-RA sNPF
MSSPQPLNYSQNASEAAKDFITSNLAVRLVFTTFYASIFLLGLFGNALVCFVVARNRQMQT
VTNLFITNLALSDILLCALGVPFTPSYTFLQKWIFGYYMCHLVPYAQGVSIYISTLTLTGIAV
DRFLVILYPFRPRMKIGICLSIIAIIWVVALLLTLPYGYYVQFQSFGDIPYCEENWPDEQFRRT
FSLLTSVLQFVLPFIVIAFCYICVSVRLNDRAKMKPGSKTSRREEADRERKKRTNRMLIAM
VAIFGVSWLPLNIVNMVNDFYEPAQNWIYYKVLFFMAHCLAMSSTCYNPFLYAWLNENF
RKEFKQMHLSCRNRLGYLVNLLVASPLRRLLPRASSSRGEHEVIGGNVCCTEHRTTDRLTP
RGALL
>NV15762-RA sNPF
MSSSQALNHSQNASVLEPANDFITSNLAVRVVIITFYVSIFLLGLFGNALVCFVVARNRQMQ
TVTNLFITNLALSDILLCALGVPFTPSYTFLERWVFGDSLCHLVPYAQGVSIYISTLTLTGIAV
DRFLVILYPFRPRMKIGVCLSIIVTIWIVALLLTLPYGLYMQFQSAGKIRYCEENWPDEQFRR
TFSLLTSVLQFVVPFIVIAFCYICVSIRLNDRAKMKPGSKTSRREEADRERKKRTNRMLIAM
VAIFGVSWLPLNIVNMVEDFYQPAQDWSYYKVLFFMAHCLAMSSTCYNPFLYAWLNENF
RKEFKQMHSSFRNRLRFLVNLLVASPLRRLLPRASSSRGEHEVVGGNVCCTEH
>BmA7
MNETIVNYTDLNISNISEKITNATANGLPPFQANSHENIIDNKWVQAMFCVIYTIIFVLGLLG
NILVCFVVIRNKAMQTVTNLFISNLALSDILLCIFAIPFTPLYTFRGTWSWGSLLCHIMPFAQ
GCSVYISTLTLMSIAIDRFFVIIYPFRPRMKIETCITVIIMIWTFSITVTTPYAIFMTYYDFKFG
KFCEETWPSERLRRIFGSVTSVMQFVLPFIVIAVCYICVSFKLNDRAKAKAASKNSKKEEL
DKNRKRRTNQMLIAMVTIFGLSWLPLNIINLCNDYYMYAIHLKYYFLIFFVAHVIAMSSTC
YNPFIYAWMNENFRKEFKQLIPCIDSSAQTRGNIQMEQLGAGPEKTFNGNTTTDSYLGSSS
QRATSFRHKRKPSAAADVEKSGVELNEDLLTVDVKHCHISTSYNLRRESVKLRLINEESFD
GTPSQSQF
>BmA10
MPTETWTSNDTTAYNGTSITVNHSNDNVNASFSDLIEYKGVQAAFCVAYTIIFAVGIFGNA
LVCYAVIRNRAMQTVTNLFITNLALSDILLCVFAVPFTPLYTFLARWVFGSLLCHIMPYAQG
CSVYISTLTLTSIAIDRFFVIIYPFKPRMKIKTCLGLIIFIWFFALLVTFPYGYYMSLTDIYCEE
KWPSDHIRKAFGAITTIMQFVIPFIVMAFCYTCVSIKLNDRLKSRPGSKNSKKEDAERERK
RRTNRMLIAMVAIFGLSWLPLNLINMSTDFYSLTEIWKYYMLVFFLAHFIAMSSTCYNPFL
YAWLNENFRKEFKQILPCLGAFVTKKSKRKFNQSDRTGMYRSEKTCNGNDTVQESLLTST
INKIPSVRYKIEFNDKLKGYDEEAVDNISPDEKPESNPSPNEDCLNMYMFADKSVISSDKEP
IVSAL
>BmA11
MFGNDTLQDVMASTIAAKYEPAMSLNGTTYVGGGVLILTRTLTGESVEMIDEPKTNKTIDI
IDVKLVQVAFCILYTIIFVLGVFGNVLVCYVVFRNKAMQTVTNLFITNLALSDILLCVFAVP
LTPMYTFLGRWVFGRLLCHLMPYAQGTSVYISTLTLTSIAIDRFFVIIYPFHPRMKLNTCIFI
VIFIWVFSLVVTCPYGLFMGIQTTNNETYYCEESWPSDRSRKIFGVFTTVLQFLIPFLVIAVC
YTCVSIRLNDRARSKPGAKNSKREEADRDRKRCTNRMLISMVAIFGISWLPLNLINIFNDF
YAQMTEWNYYFVSFFLAHSMAMASTCYNPFLYAWLNENFRKEFKQVLPFFESNGGVRNS

YHPGRVPPHKTNKNVCNGNETIQETLLASSFNRGPSIKQRFEGNGKKDNGIEVENILLEDK
TISATFHTKTENVNLQLIDEESHFSDHRDTKSPI
>DmCG7395 sNPF
MANLSWLSTITTTSSSISTSQLPLVSTTNWSLTSPGTTSAILADVAASDEDRSGGIIHNQFVQ
IFFYVLYATVFVLGVFGNVLVCYVVLRNRAMQTVTNIFITNLALSDILLCVLAVPFTPLYTF
MGRWAFGRSLCHLVSFAQGCSIYISTLTLTSIAIDRYFVIIYPFHPRMKLSTCIGIIVSIWVIAL
LATVPYGMYMKMTNELVNGTQTGNETLVEATLMLNGSFVAQGSGFIEAPDSTSATQAYM
QVMTAGSTGPEMPYVRVYCEENWPSEQYRKVFGAITTTLQFVLPFFIISICYVWISVKLNQ
RARAKPGSKSSRREEADRDRKKRTNRMLIAMVAVFGLSWLPINVVNIFDDFDDKSNEWRF
YILFFFVAHSIAMSSTCYNPFLYAWLNENFRKEFKHVLPCFNPSNNNIINITRGYNRSDRNT
CGPRLHHGKGDGGMGGGSLDADDQDENGITQETCLPKEKLLIIPREPTYGNGTGAVSPILS
GRGINAALVHGGDHQMHQLQPSHHQQVELTRRIRRRTDETDGDYLDSGDEQTVEVRFSE
TPFVSTDNTTGISILETSTSHCQDSDVMVELGEAIGAGGGAELGRRIN
>BmA4
MPFYDDMGLDPSVNLTLNASAKQLIEASKGIQDPNILLEKFSQNRKVDDPTRSLLIAFYIIL
VVIGAVGNALVILSVVRKPVMRTARNMFIVNLAVSDALVCVVGTPLTLMELLTKHWPLPD
WPSLCKACGAIQAISIFVSTISITAIALDRYQLIVYPTKPGVQTIGALVTMFFIWVTAFILASP
LYIFRSLKTHKLGIAGISSLSFCIEDWPITDGRAIYSLLSLIFQYLLPVLVVVMAHIQIHRRLR
GRRRTTRKTPAILIAIAVTYVISWLPLNVFNLVADFSSAPFKDEKTMTVTYAVCHMFGMSS
AVSNPLLYGWLNDNFRKEFEEILTKCCCRKKPLVNGTRTTNRRMETELTALAQLEHTVTG
NTKTSQCSQVF
>DmCG1147 NPF
MIISMNQTEPAQLADGEHLSGYASSSNSVRYLDDRHPLDYLDLGTVHALNTTAINTSDLNE
TGSRPLDPVLIDRFLSNRAVDSPWYHMLISMYGVLIVFGALGNTLVVIAVIRKPIMRTARNL
FILNLAISDLLLCLVTMPLTLMEILSKYWPYGSCSILCKTIAMLQALCIFVSTISITAIAFDRY
QVIVYPTRDSLQFVGAVTILAGIWALALLLASPLFVYKELINTDTPALLQQIGLQDTIPYCIE
DWPSRNGRFYYSIFSLCVQYLVPILIVSVAYFGIYNKLKSRITVVAVQASSAQRKVERGRRM
KRTNCLLISIAIIFGVSWLPLNFFNLYADMERSPVTQSMLVRYAICHMIGMSSACSNPLLYG
WLNDNFRKEFQELLCRCSDTNVALNGHTTGCNVQAAARRRRKLGAELSKGELKLLGPG
GAQSGTAGGEGGLAATDFMTGHHEGGLRSAITESVALTDHNPVPSEVTKLMPRLEQY
>BmA9
MNDPDISYNSSLLRMREITSTLAPFTTIRNASVAKPNNAYEWRFILPPYFVIFLLSICGNCLVI
ATLASNRRMRTVTNVYLLNLAISDFLLGVFCLPFTLVGQIYRRFLFGAALCKLIPFLQAVSV
SVDVWTLVAISLERYFAICRPLKSRKWQTQCHAYKMIAMVWILSLILNSPIMLVSTLQPMR
GNAHQCREVWSSLELERAFNLGLDAGLLLLPFFVMSFAYCLIVTKLWRGMRHEIQHNFN
WQRHQTHQASYKNNQLLPATTIKKSNSADVCCNKTNQTNQKKPNKDTSEQVEPDARSTQ
PYCMHAVDHEFRHFVRSTHIDKSIEAKRKVIRMLFVIILEFFVCWTPLHVINTIYLFYPDQL
YEHIGSKGIICSQLLAYCSSCCNPITYCFMNRKFRQAFISLSKSCGIFGLCCREKSESGKQAA
PPPVSSSQEVTACVIRGSQTGRTELDGLEGKDCV
>DmCG6881 SK
MFNYEEGDADQAAMAAAAAYRALLDYYANAPSAAGHIVSLNVAPYNGTGNGGTVSLA
GNATSSYGDDDRDGYMDTEPSDLVTELAFSLGTSSSPSPSSTPASSSSTSTGMPVWLIPSYS
MILLFAVLGNLLVISTLVQNRRMRTITNVFLLNLAISDMLLGVLCMPVTLVGTLLRNFIFGE
FLCKLFQFSQAASVAVSSWTLVAISCERYYAICHPLRSRSWQTISHAYKIIGFIWLGGILCMT

PIAVFSQLIPTSRPGYCKCREFWPDQGYELFYNILLDFLLLVLPLLVLCVAYILITRTLYVGM
AKDSGRILQQSLPVSATTAGGSAPNPGTSSSSNCILVLTATAVYNENSNNNNGNSEGSAGG
GSTNMATTTLTTRPTAPTVITTTTTTTVTLAKTSSPSIRVHDAALRRSNEAKTLESKKRVVK
MLFVLVLEFFICWTPLYVINTMVMLIGPVVYEYVDYTAISFLQLLAYSSSCCNPITYCFMN
ASFRRAFVDTFKGLPWRRGAGASGGVGGAAGGGLSASQAGAGPGAYASANTNISLNPGL
AMGMGTWRSRSRHEFLNAVVTTNSAAAAVNSPQL
>DmCG6857 SK
MLPRLCADACRQCFAKIARRDTHRGTRTPYGCADTQSRPKPNFLLREVDEVCCTAASASP
RLLVLFRDHKRASFFGLTIDAFYHYLRQALPLAKEAAIHLNASNEISAVGDGVTITGTPGDL
LNYSGLELDLGLDLDLNLDMDLATTPSSSTLAPAVTVRTPGNRSVVRVSADVPIWVVPCY
SAILLCAVVGNLLVVLTLVQNRRMRTITNVFLLNLAISDILLGVFCMPVTLVGTLLRHFIFG
ELLCKLIQFAQAASVAVSSWTLVAISCERYYAICHPLRSRTWQTINHANKIIAIIWLGSLVCM
TPIAAFSQLMPTSRPGLRKCREQWPADSLNYERAYNLFLDLALLVLPLLALSFTYLFITRTL
YVSMRNERAMNFGSSGPEVTTSSSAAVAEAGSQRRANGSHCQSLDTIVPHQHNPHQQHH
HHSQYYYDYGHCGSKRRLISGGGPCEGRRHLYCMRSASVKSLRHQQINGGGGTLSGTGA
GNGECCSRVHRMRQQMQLQQQGYVSDNESRRKSLSQPSLRITEAGLRRSNETKSLESKK
RVVKMLFVLVLEFFICWTPLYVINTMTMLLGPTVYEYVGYTSISFLQLLAYSSSCCNPITYC
FMNASFRRAFVDTFKGMRVCERLCAPCCFWRRRSKNETNLSVAGNSIALANSVMSSHTIL
ESPRL
>PPU13001-RA
MAATALAATSAVMPPKLDNADDNSSLDMEQMSEILRGFYDDFNSETNYLLIGLYVPVIILA
MTANILVIVVVFKYQYMRSVTNYFVVNLSVADLLVTMICMPMAVSQAVSIIWVYGELMC
KLFFYLQGVAVAASVFTITAMSIDRYLAIRNPIAFRRVFNRKSTIIVIAALWVVALSIFAPVLR
AVTLQSPITDLYNITLSGQWASDEPGVQMPKPPTFYVCSEDFKPLGIHAHIFGAACFVLVYA
VPGFIVIIAYSMMGRTLCARKPPFDCDSIEGSASSQQGFRLVRERRRVAWILLLLAVLFALC
WLPYNVLRLLVDLGVVHEGRLISDVLSYCLFLGHANSALNPIVYCFMTRNFRRSVAEILCR
GNYGLARRKPHRKTLGSAVGVCAGCNNNSAGRAYYPKAHPGDSVGRGIFAPSGLVGQQH
QQSMPGTAGPTSSLTLLQPGITGTTMIAGGVPTTALHQISSQGGQTTSSAPAHAVLALRTAS
NNCSPSNTGSSSGYDSFYSRHSPHRRCYMLRSLPQDQERHKAISPAKECNGDDCNVLVTS
KKSLHRHSHSSRSSSSSSHSRIHRDSCDAGKQSSLISSDEQRFM
>NV20652-RA
MAATALAATSAVMPPKLDKAENSSLDMAEMSEILRGFYEDFNSETNYLLIGLYVPVIILAM
TANILVIIVVFKYQYMRSVTNYFVVNLSVADLLVTIICMPMAVSQAVSIIWVYGELMCKLFF
YLQGVAVAASVFTITAMSIDRYLAIRSPIAFRRVFNRKSTIIVIAGLWIVALSIFAPVLRAVTLQ
SPITDLDNITLAGQWASDEPGLQMPKPPTFYVCSEDFKPLGIHAHIFGTACFVLVYAVPGFI
VIMAYSMMGRTLCARKPPFDCDSIEGSASSQQGFRLVRERRRVAWILLLLAVLFALCWLPY
NVLRLLVDLGVVREGRLISDVLSYCLFLGHANSALNPVVYCFMTRNFRRSVAEILCRGNY
GLARRKPHRKTLGSAVGVCAGCNNNSAGRAYYPKAHPGDSVGRGIFAPSGQAGQQQSTP
GTAGPTSSLTLLQPGTTGTTMAGGAPTTAVHQASSQGNQSPSSTPAHAVLALRAASNNSAS
NTGSSSGYDSFYSRHSPHRRCYMLRSLPQDQERHKAISPAKECNDEDCNVLVTSKKSHRH
SHSSRSSTSSSHSRIHRGSYDAGKQSSLISSDEQRFM
>BmA12
MTTMDTPNFSHVNGLNLSGNASGGLHLETTVDIFFQPEHVLITLYVPVILLSFIANILLIVVA
IKCNYTKNVTDIFLVNLSAADLLVTGICMPIQLSKAITLVWFYGETVCKIVNYIQGVAVAAS

VFTISAMSVDRWLSITPEPRLRPPGRKQATLLLMLLWIAALLIFIPTSLVAGVRKETIPIISKG
DKNISIETRDIHFCIEEWPSPETRKQYGMFSFTLVYAIPGSITIMSYACMGRTLCSVRPPFDID
EGNVSMQQGLRLMKERKRVAWILLLLAVLFALCWMPYNIMQLLLDVSVVNAKDLSAYLP
YALFLGHANSAINPIVYCFMTRKFQRSVKKLLCGRPLCQQAKFTWRCNQKPEGSSSDYEL
YHEPHKRCYLQMNTLRYNGHYSAQIPRGIETRAQTTQLSHVTRSSRRTAPAHALSAEMLQ
RHGHIDYRR
>BmA35
MDRSEISFENMTFLEWNGSMGDELNMPHLDLEVFYRPDIVIIALYVGVLTASLSANTLLIF
VVIKFQYMRNVTNIFLVNLSVADLLVTLFCMPVQIAKSVTLLWYFGEVMCKTVNFLQGVA
VASSVFTITAMSVDRYLAITQSLRQPWMPSRRGACGLLVCLWLIALAIFAPLLAVAAVERER
VPPLSRTKNGSQILVERTIEFCTEKWPDSIKKELYGAFSFILVYAVPGCIVVVSYSLMGRRLC
SVLPPFDQTEGSANSQQRLRLVRERKRVALILLLLAVLFALCWLPYNILQLLVDVNAVQVD
SVSLVLPYTLLLGHANSAINPIVYCLMTRNFRRSLRKLICHDPGNINSNTHFHVSLHTFSRL
SLTSYWNSPLTLIVLTCFAANVSKAREG
>BmA17
MSYGNASDDAQTDEATHAHALATLLAMYVLVSLIGIIGNVSLMAALASGGAARLRTPQM
LSACAADFLVCAASAPLAATRAATVETLPCHVTYYIETFPVAASTLSLVAIAADRCGAVHR
GRGTTLCSRPFLAVAAVWSMALLLGAAAFTTTCITCPPLAAVHAVVAFCFPVIAVARCHWS
VRVKLTALSLTARAAHGELPLPVPLMRRPTHVIIVAGVGPRERRDAVDVGDARSKKKLQPS
LLGPQPQTSTLRSRRRLGNVLMGIAGIFAMCWCPHAAIVICGSFGIHVPEMVGHYALLLGY
AHSALNAVAYWVLNRHALTSACTAWHLPQLRVREERPSSTNEAALGAFHPRLARPAPSPR
PPPSSFLY
>DmCG13995
MNRDNLQQWWENSYRRQHPEPTDDLGLDSAELHLALQEPNQLPADYDYGNFSLGNPYD
VDSEHSISPLTLLLLAVSYGLVVFGGVVGNSTLVLTLCSASSVRLRNPLLLAVCIADLLVTGI
SAPVTLLNLAMNRRTRSLPLVLCKVIHYVQVMPVSASTISFFMLSLDRYATVKHPRLAQLR
QRRYLHVSLALLSWLASAAISTPFLFAYKIIAKSMVVKGGGAANTTPNPVSISCTSDLGAN
AMFMSFIIFHTIAVFVLPGIGVLLNHYGVRRKLCALSLTARAAHGELPLPIPILRRQTHMVIV
TGCPNAQQAACGGGTTADDTSNGNGTGTGGGPMAVSPGDIQLHTLQPRQPGSAGSALEP
GSYRSSNPISPRAMREIRAHSQRQRINRAGRGPATPGIPLPQTSTLRSRRHLANMLIASAVIFI
ACWAPHVFCIFYKNFGNNQQCSQTSVYFSLLLGYFYSAISPVIYWALNHNSLRQSPCAPIIR
LRSMQNFLRSRFRTHTAPPPPSSTNEAALGAFNPKLIKLTPKQYRAQASSHYLY
>PPU08251-RA CCHa2
MYNITRHFNGSTSTIFSLEEEDEEDQYIPYEDRPETYIVPILFFLILVVGVAGNGVLVLTLLR
HANMRNIPNTYVLSLALGDLLVIVTCVPFTSILYTIESWPWGLAVCKLSECAKDISIGVSVF
TLTALSAERYYAIVNPIRRHVAGLSAKPLTILTVTLIWLLASILALPAALFSHIPTEPLKGNHSI
TICSPFPKEFGESYEKGMVLFKFLAYYAIPLCIISGFYLGMARHLELSTRNMPGEHPGAPHH
CEQIRARKKVGKMVIAFVIIFFVCFLPYHVFMLWFHFYPASREVFDEFWHAFRIIGFCLSFI
NSCVNPIALYFVSGTFRKRFNEYLCCCIPSVRRAANAQGRFPRTRGEASTFYETSFNSTYRR
HTQELNSSTLLNYSAGESKPA
>PPU08247-RA CCHa1
MAYGTTSLPEEFLLQHHEITGNESFTECLNSTSGCAEENYVPYGDRPETYIVPIVFACILVV
GVMGNGLLMLTICRHSNMRNVPNTYVLSLAIGDLLVILTCVPFTFTVYYVLDSWPFGLMLC
KVSECAKDISIGVSVFTLTALSADRFFAIVDPMRKLHATGGGKRATRFTMLVATLIWLLAIF

CAIPASFSYIRVFRVNKNVTFLTCYPFPEEFGPNYPRTVLICRFCIFYVFPLSIIAVFYMLMAR
HLVQSTRNIPGEMQGQVKQIKARKKVAKMVMAFVAVFAICFFPQHVFMLWFYLHPSAQE
DYNAFWHYFRILGFCLAFMNSCINPIALYCVSGTFRKYFNRYLMCYTRALDVVSKVSFICL
PGRRVTSPMHEDDAIPRNRRLPSRSFRMGLNNGYEIKVFGASIGGSRDQTITIE
>NV12199-RA CCHa2
MSEGTNAKVLIWLFYLNTPLPKNVYLHLINGAASYPAKFFNYNVISTQPIKPYIFQRMYNI
TWHLNGSTSTIFSLEEEDEEDQYIPYEERPETYIVPILFFLILVVGVAGNGVLVLTLLRHANM
RNIPNTYVLSLALGDLLVIVTCVPFTSILYTIESWPWGLAVCKLSECAKDISIGVSVFTLTAL
SAERYYAIVNPIRRHVAGLSAKPLTILTVTLIWLLASILALPAALFSHIPTEPLKGNHSITICSP
FPKEFGESYEKGMVLFKFLAYYAIPLCIISGFYLGMARHLELSTRNMPGDHPGAPHHCEQI
RARKKVGKMVIAFVIIFFVCFLPYHVFMLWFHFYPASREVFDEFWHAFRIIGFCLSFINSCV
NPIALYFVSGTFRKRFNEYLCCCIPSVRRTANAQGRFPRTRGEASTFYETSFNSTYRRHTQE
LNSSTLLNYSAGESKPT
>NV12201-RA CCHa2
MAYGTTILPEELLLQHQNTGNDSFAECLNATSGCSAEENYVPYGDRPETYIVPIVFACILVV
GVMGNGLLMLTICRHSNMRNVPNTYVLSLAIGDLLVILTCVPFTFTVYYVLDSWPFGLMLC
KVSECAKDISIGVSVFTLTALSADRFFAIVDPMRKLHATGGGKRATRFTMVVATLIWLLAIF
CAIPASFSYIRVFRVNKNVTFLTCYPFPEEFGPNYPRTVLICRFCIFYVFPLSIIAVFYMLMAR
HLVQSTRNIPGEMQGQVKQIKARKKVAKMVMAFVAVFAICFFPQHVFMLWFYLHPSAQD
DYNAFWHYFRILGFCLAFMNSCINPIALYCVSGTFRKYFNRIQEVYVDDEAKLTLHGLQQ
HYLKLKEKEKNKKLFELLDELEFNQVVIFVKSVQRCNSLTQLLTEENFPTIGICKGMTQEE
RLTKYQSFKDFQQRTLVATNLFGRCLDIERVNIVFNYDMPENSDTYLHRVARAGRCGTKG
LAITFDCDEADAKILNDVKERFDVNIAALPDEIDLASYIEGR
>BmA14
MTAEANYTMSAENDTDEYMPYDERLETYLVPILFAVIFVVGVLGNGTLVIVYARHRGMRN
APNTYIFSLALADLLVILICVPFVSIIYTLESWPWGELICRISESAKDVSIGVSVFTLTALSAE
RYCAIVNPFRKLQLRKLPLVCATFIWGAAFIFAIPAAIFSSTVTVELKDNVTIVYCTPYPKD
WTNYSKGMTIAKALVYYGLPLIVITVFYSLMARRLLASTREMPGALQGGQGEAQAKARK
SVACMVLIFVIVFFICFLPYHAFELWYHLSPTSLLDYNDWTHALRIIGFCLSFLNSCVNPVA
LYCVSGVFRQHFNRYLCCRRSALHPTCSSRLSRTAICETSFRSTHRHRCNRNPTTESVVISN
YDYGSTNKKSNIITRNNADGVTILTIRDSNVFISGEIDDKRINR
>BmA15
MMQETNETYDNSTEIYQPYYERPETYIVPILFALIFVIGVVGNGTLVAVFVRHKAMRNVPN
TYILSLALADLLVIITCVPFTSIVYTVESWPWGRTVCQVSEAAKDVSIGVSVFTLTALSADR
YFAIVDPLRKLHATGSSKRATRLTIATAIGIWILAGLLATPAFIGSYLRPFVVNPTTQFLVCYP
YPQEWGEHYAQIVVMVRFLLYYSLPLAVIALFYVLMAWHLVLSTQNMPGEMQGTQRQM
RARRKVAVTVLAFVLVFAACFLPSHVFMMWFYFCPTAENDYNGWWHGLRIVGFCLSFLN
SCVNPIALYCTSGIFRKHFNR
>DmCG14593 CCHa2
MYASLMDVGQTLAARLADSDGNGANDSGLLATGQGLEQEQEGLALDMGHNASADGGI
VPYVPVLDRPETYIVTVLYTLIFIVGVLGNGTLVIIFFRHRSMRNIPNTYILSLALADLLVILV
CVPVATIVYTQESWPFERNMCRISEFFKDISIGVSVFTLTALSGERYCAIVNPLRKLQTKPLT
VFTAVMIWILAILLGMPSVLFSDIKSYPVFTATGNMTIEVCSPFRDPEYAKFMVAGKALVYY
LLPLSIIGALYIMMAKRLHMSARNMPGEQQSMQSRTQARARLHVARMVVAFVVVFFICFF

PYHVFELWYHFYPTAEEDFDEFWNVLRIVGFCTSFLNSCVNPVALYCVSGVFRQHFNRYL
CCICVKRQPHLRQHSTATGMMDNTSVMSMRRSTYVGGTAGNLRASLHRNSNHGVGGAG
GGVGGGVGSGRVGSFHRQDSMPLQHGNAHGGGAGGGSSGLGAGGRTAAVSEKR
>DmCG14484 CCHa1
MIANLVSMETDLAMNIGLDTSGEAPTALPPMPNVTETLWDLAMVVSQSTQWPLLDTGSS
ENFSELVTTETPYVPYGRRPETYIVPILFALIFVVGVLGNGTLIVVFLSVRQMRNVPNTYILS
LALADLLVIITTVPLASTVYTVEYWPYGSFLCSLSEFMKDVSIGVSVFTLTALSGDRYFAIV
DPLRKFHAHGGGRRATRMTLATAVSIWLLAILCGLPALIGSNLKHLGINEKSIVICYPYPEE
WGINYAKSMVLLHFLVYYAIPLVVIAVFYVLIALHLMYSASVPGEIQGAVRQVRARRKVAV
TVLAFVVIFGICFLPYHVFFLWFYFWPTAQDDYNAFWHVLRIVAYCMSFANSCANPVALYF
VSGAFRKHFNRYLFCRGASGRRKKRGQHDTFCMHRDTSLTSTASKRFQSRHSCYQSTIRS
CRLQETTITTLPNGGNQNGANISAVELALPVLQAPGHNEAHAPPSYGFLPLNEIVQQTRSSP
AKFQESLLN
>PPU08836-RA SIFa
MSVCYSGLPPVASNRPRLQWLHGGEPGVSLRRSAVGLLYERTVLRPYPLRQYPLRPYPPAP
VPPCASTRLRQYPPAPVPACARTAMGQNRHGLESAWARIAMGQNRHSSPTRMYQHTNRM
SKSSICRSTPIQKTRDNLQAKTQVSCWSYISSYTAALLHELTLTLLIHRIVVSHSAMTSGQM
AIICAIKMFYYSDTNKHQQQHHLHADLQYDGLRLSAPAAAASMLQPPSPLLEMASLRLPD
SDEFDLERRRSGGQQSATGLPSGEQLGDLRGLVAARAEIFAEYVNGSVQDLLVLASTRRPS
PFAGGGGSAGTMDAAGPEDDDEMQRLAGFFNCTNCNISVELVPDRWYRHSVAMSVVYF
VAYCLVFVVGLIGNSFVIAVVYRSPRMRTVTNFFIVNLAVADVLVIVFCLPATLMSNIFVPW
VLGWFMCKIVPYIQGVSVAASVYSLVAVSLDRFLAIWWPLKCQITKRRARMMIVIIWFIAL
TSTMPWLLFFDLVPIYSDDPNLKLCLERWPNPEDDSLFFLIGNLMLCYVLPMILISLCYVLI
WIKVWRRHIPSDTKDDQMERLQQKSKVKVVKMLIVVVILFVLSWLPLYVIFARMKFGGKI
ADWEEELLPIATPIAQWLGASNSCINPILYAFFNKKYRRGFMAILKSGQCCGKLRYYETVA
MMSSSTSMRKSSYYVNNNNSSTRRAFHGPPVHQDSNVSYIFNHTGV
>NV17731-RA SIFa
MVDQSSVAARHQHYHQHAAQHQQHHLHSDLQYDGLRLSAPAAAASMLQPSSPLLEMAS
LRLPDSDEFDLERRRSSGQQSATGLPSGEHLGDLRGLVAARAEIFAEYVNGSVQDLLVLAS
TRRPSPFAGGGGPAGTMDAASPENDDEMQQLAGFFNCTNCNISVELVPDRWYRHSVAMS
VVYFVAYCLVFVVGLIGNSFVIAVVYRSPRMRTVTNFFIVNLAVADVLVIVFCLPATLMSNI
FVPWVLGWFMCKIVPYIQGVSVAASVYSLVAVSLDRFLAIWWPLKCQITKRRARMMIVVI
WFIALTSTMPWLLFFDLVPIYSDDPNLKLCLERWPNPEDDSLFFLIGNLMLCYVLPMILISL
CYVLIWIKVWRRHIPSDTKDDQMERLQQKSKVKVVKMLIVVVILFVLSWLPLYVIFARMK
FGGKIADWEEELLPIATPIAQWLGASNSCINPILYAFFNKKYRRGFMAILKSGQCCGKLRY
YETVAMMSSSTSMRKSSYYVNNNNSSTRRAFHGPPVHQDSNVSYIFNHTGV
>BmSIFaR
MKMAPLRLPVDYYTDDFLNFSTQNPNNERHHTRHNHSHLRESHKNHVADMLSNSIIDAF
NTRFVENSVLPDMEPLSSHMDLEERHYPSRMNGTLNRSDFGGDEFMYRHSGAMTAVYC
AAYLLVFLVGLVGNCFVIAVVYRSPRMRTVTNFFIVNLAFADILVIVFCLPATLMSNIFVPW
VLGWLMCKTVPYVQGLSVAASVYSLVAVSLDRFLAIWWPLKCQITKRRSRMMIVFIWIFA
ILVTTPWVFFFDLVVVFEENPNVHLCIDVWPNPLSEVLYFVVGNLIFCYILPMVMITMCYIL
IWIKVWRRSIPTDTQDAQMERMQQKSKVKVVKMLVAVVILFVLSWFPLYLIFARIKLGGPI
KKWEEEMLPIVTPLAQWLGASNSCINPILYAFFNKKYRKGFVAIIKSRKCCGRLRYYETIAL

QSSSTSTRKSWHYNNNNPSITRRSPPVDKNAVSFIFSHTGV
>DmCG10823 SIFa
MMAASGRIRKRKHKSHTSGDVPSTTTSVPMPIPTMAPGKMVAETMEEAAALAGDYNNFT
HNFVDLQNLLSFNELNGTSGSGGTAVSSLGSSSAIKLNNSAITDTLLGTVLTTATATVAPAAS
SLLATLAATTTASARGSLAGKSLAIADATSSTYYSNLLNLSPATTSLISAAAATKSYNDSAL
RWEQLDGSVDFGFDPLYRHSLAMSMVYCVAYIVVFLVGLIGNSFVIAVVLRAPRMRTVTN
YFIVNLAIADILVIVFCLPATLIGNIFVPWMLGWLMCKFVPYIQGVSVAASVYSLIAVSLDRF
IAIWWPLKQMTKRRARIMIIGIWVIALVTTIPWLLFFDLVPAEEVFSDALVSAYSQPQFLCQ
EVWPPGTDGNLYFLLANLVACYLLPMSLITLCYVLIWIKVSTRSIPGESKDAQMDRMQQK
SKVKVIKMLVAVVILFVLSWLPLYVIFARIKFGSDISQEEFEILKKVMPVAQWLGSSNSCINP
ILYSVNKKYRRGFAAIIKSRSCCGRLRYYDNVAIASSTTSTRKSSHYHQNSSRKSPSSKGNA
VSYIYEHNSLRRHNMMLKQDSNLSQQMLLKQDSHGSRQFLIKQESSCSDASGIRRPLCQQ
DSNGSKVSLSKQDSIVSYMEARRSAGHGLNDTLVDRDSVSMDVGRRQGATPSSLLDKRQ
KFVKQDSVISFVDQRPEQRRHQLVKQDSVISFADQRRGLLHKQDSLMANRTGDAPTHHV
SILKKTDSQLSYGSSTSPRRNADLYE
>PPU05054-RA RY
MEICPENRPRKHYAGICTSSSSMTEEVENVTSVVGWLNESLSNETIIYDCDNFAGGPLSVQ
WFQAILYVLYSSVFVIALLGNVMVCYVVYKTGQQNRNKRSNIQNRQTNYYLVNLAIGDIL
IDLFCVPTSFISTLVLQYWPFRAELCPVVNYSQAVSVLVSAYTLVAISMDRYVAIKYVLKPR
GSTKSAKFSIFIVWLLAIIVAFPILIVSGVDQPQRKYEVCERYVCIEIWSDQKQRYYYTFSLLI
LQFVVPLLILTFSYTSIAIMVWGKRPPGEAENNRDQRMTRSKKKMTKMMVAVVFVFSICW
LPFNLLNLVMDYNESLRHWHGLPYVWASLHWLSMSHACYNPVIYCWMCTTFRRGFYSV
LKRVPLVGRYIPDKSRAYNVGGIPLAGSDGQNNSSLRRINTYTTYVSVRRKTNHNYGAPIR
SASFRCNNSLRNSGPMHRHFIHLEAQPEESL
>NvRYR
MTSTQDEICSGGDINCSMTEEPAIVTGTADWRNLSVLENGSLIFDCDDFNGNIPFTSTLAQV
ILYILYGSMFVIALGGNSLVCAAVIRTRNNLRPNNLTNYFIVNLAIGDILINIFCVPTSVLSTL
VFHYWLLPSQLCAIFNFFQAVAVLVSAYTLVVISMERYLAIMYLFRPRRGTKYAKISILIVW
LLAMAISLPILVVSDVEQPDIRYEMCDFYTCTEKWSDKKQKYSYTIALLILQYLVPLLILLF
SYISIAIVVWGKKAPGEAEDNRDQRMIRSKTKTIKMLMAVVIVYTICWLPFNVLNLVMDI
NEDINRWYGLPYIWAMLHWLAMSHACYNPFIYCWMSTPFRQGIFNMLKCVPIIRRFVPDR
SHALNTSAVGIPLTGFDGQHNSSLRRKNNCTTYVSVRKKMNHNHGAPIRSASFRCNNSLR
SSGPMHRHFVHLEVQQEESL
>BmA19
MLDMYESESIDFDSSTLSDFLIRNVTSSEYYINATASSNLTKLDYDAICNPSTSSESFFTSATF
QTCVYFMYCIVFVVALVGNGLVCFVVQTSPRMKTVTNYFIVNLAVGDILMTLFCVPFSFV
SMLVLRYWPFGGIMCKVVNFSQAVSVLVSAYTLLAISIDRYMAIMRPLKPRMGKTAAKM
VVAGVWGGAIATATPIYVVSKLERPAEWHKYCQLDICHEEWDHVEQSERYTCALLVLQFV
LPLSALVCTYARIAHVVWGGRPPGEAESTRDSRMQRSKRKMIKMMVIVVAVFTVCWLPL
NIFIVLWTLHEGDEEWAAWPGMPYVWFASHWLAMSHSCYNPIIYCYMNTRYRRGFKQAL
GWFFRVRFETTSPRCHHSSICEGMQMSEMVGVNGIVRRGTSSSCVSRLQRAPTCSSCASV
RRGVAGGFTTPVPPIRALSVRTHFN
>BmA22
MSMDQSEELSNDMNYKATYAANVHHRNDTIFNQSAYNDTWPSHELFCIYESSSEDFLSSP

VFQICVYFMYSAVFLVALLGNGLVCFIVHTSPRMKTVTNYFIVNLAVGDILMTLFCVPFSFV
SMLVLRYWPFGAVMCKVVNFSQAVSVLVSAYTLLAISIDRYIVIMRPLKPRLGKGAAKMV
VAAVWGGAIITAAPIPIVSQLQRPSPWHEACKVDICSEQWADGKQSEHYTFALLTLQFTLPL
TALVYTYGRIAHFVWGGRPPGEAESGRDSRLQLSKRKMIKMMVTVVAVFVVCWLPLNIFI
VLWTLHEGDEDWAVWPGMPYVWFASHWLAMSHSCYNPLIYCYMNAKYRHGFKQVLSG
LFCLKLNESKRSCQRSSLCERIPLSGELIIFHS
>DmCG5811 RY
MEHHNSHLLPGGSEKMYYIAHQQPMLRNEDDNYQEGYFIRPDPASLIYNTTALPADDEGS
NYGYGSTTTLSGLQFETYNITVMMNFSCDDYDLLSEDMWSSAYFKIIVYMLYIPIFIFALIG
NGTVCYIVYSTPRMRTVTNYFIASLAIGDILMSFFCVPSSFISLFILNYWPFGLALCHFVNYS
QAVSVLVSAYTLVAISIDRYIAIMWPLKPRITKRYATFIIAGVWFIALATALPIPIVSGLDIPMS
PWHTKCEKYICREMWPSRTQEYYYTLSLFALQFVVPLGVLIFTYARITIRVWAKRPPGEAE
TNRDQRMARSKRKMVKMMLTVVIVFTCCWLPFNILQLLLNDEEFAHWDPLPYVWFAFH
WLAMSHCCYNPIIYCYMNARFRSGFVQLMHRMPGLRRWCCLRSVGDRMNATSGEMTTK
YHRHVGDALFRKPKICIRCKTLHLVSVSVFLFVLLRFWFI
>PPU04628-RA AKH
MSTALVNTTTAASLGYDDLPIDMRFNAGHVVSIVTYSILMIISAVGNITVLALLLRRRGNA
ARTRINTMLIHLAIADLLVTFLMMPLEIGWAATVSWKAGDAMCRIMSFFRMFGLYLSSFILI
CISVDRYHAVLRPLQMIDIDRRGRFMIAGSWICSALCSAPQMVVFHVEAHPTFTWYEQCIT
FNTFPSFTHELTYSLFGMVMMYWFPLIVIIYTYTSILAEMYRRSKDTTSDRIRRSSLGFLGR
ARVRTLKMTIIIVLVFFICWTPYYVMSLWYWIDSVTATKVDLRIQKALFLFACTNSCMNPIV
YGAFNIRKGNKVTRNWDIHTLK
>NV16060-RA AKH
MTTAPVNATTVASLDYDDLPIDMRFNAGHVVSIVTYSILMIISAVGNITVLALLLRRRGNA
ARTRINTMLIHLAIADLLVTFLMMPLEIGWAATVSWKAGDAMCRIMSFFRMFGLYLSSFILI
CISVDRYHAVLRPLQMIDIDRRGRFMIAGSWICSALCSAPQMVVFHVEAHPTFTWYEQCIT
FNTFPSFTHELTYSLFGMVMMYWFPLIVIIYTYTSILAEMYRRSKDTTSDRIRRSSLGFLGR
ARVRTLKMTIIIVLVFFICWTPYYVMSLWYWIDSVTATKVDLRIQKALFLFACTNSCMNPIV
YGAFNIRKGNKVTRNWDIHTLK
>BmAKHR
MDIDEKVSGPGGASQKNWSHLLHVNNTYDELPLEMRFNYSHMVSMTVYSVLMVISATG
NLTVLYQLVRRRRAKRASRLDILLMHLAVADLMVTFLMMPLEIAWAGTVQWFAGDLMCR
VMMFTRTFGLYLSSFVLICIAVDRYYAILKPLNVTWEATVRRAIIVAWVCAGLASLPQSFIF
HVEEHPEVKGYNQCVSYGSLPTEKHEFAYFLVNMILMYVIPLVSTLYCSCAALFEIIRRANT
ANDKMRRSGIGLLGRARARTLKMTVTIVLVFFTCWSPYYCYCLWYWIDKESIKNLDPALQ
KAMWLFSCTNSCANPIVYGVFNRNRWNWRAGKFQNGRCRSGSGRKGSRLPHGESTEISA
ATLSRARHSNGSDHNGRRDSSYANQNGPQKHWNTINNNHVTNGMV
>DmCG11325 AKH
MAKVAEENDHRDLSNWSNVNDTNGTIHLTKDMVFNDGHRLSITVYSILFVISTIGNSTVLY
LLTKRRLRGPLRIDIMLMHLAIADLMVTLLLMPMEIVWAWTVQWLSTDLMCRLMSFFRV
FGLYLSSYVMVCISLDRYFAILKPLKRSYNRGRIMLACAWLGSVVCSIPQAFLFHLEEHPAV
TGYFQCVIFNSFRSDFDEKLYQAASMCSMYAFPLIMFIYCYGAIYLEIYRKSQRVLKDVIAE
RFRRSNDDVLSRAKKRTLKMTITIVIVFIICWTPYYTISMWYWLDKHSAGKINPLLRKALFI
FASTNSCMNPLVYGLYNIRGRMNNNNPSVNNRHTSLSNRLDSSNQLMQKQLTNNSLLNG

RGQVMAAAVSATTKLANVVSLKGTANGNGSAAAAGTVPITPPLTVTIAPLATDDEANDDS
CLSAVTIRCQDQSPIRQKCGESIELTSVVK
>PPU13782-RA ACP
MDQLQGTRMQLLQDFNNDFRNDSFYSYDFRDNMSMAMPTMPPSMTFTRRTLTIIIVYCIC
FLVAAIGNLTVFLTLWRGRYRKSRISLMICHLSIADLLVAFFTIPIEIGWRLTVQWIAGNYAC
KLFLFLRAFGLYLSNNILICVSLDRYFAVLYPLRVNDARRRGKLMLSVAWFFSVLYAIPQSIV
FHVENHPNHKNFTQCVTFGAFPSDLVENTYNVFCVLTMYFIPLAIICWVYLKILCEISSKSR
DNKPVVIKTGSNGTLESSNSNQGSRMRLRRSDMSSIERARSRTLKMTIIIVVAFIFCWTPYIT
MNLWYVIDKKSAKEVNEMVQESLFIMAVGNSCANPLVYGSYAIDLKKECCRCFLPCTTTK
TNADVNLIQRSLGSKFQKPEMKSPGVSKQIVHSVCQAVHGFFKVGSGQTKSTAVCGKVLA
PVSPRLSVSTTSKGAVVEKLPLHTIVHDLFGYSIPVFDISLNN
>NV14629-RA ACP
MDQLQGSRMQLLQDFNNHDFRDNMSMAVPTMPPSMTFTRRTLTIIIVYCICFLVAAIGNLT
VFLTLWRGRYRKSRISLMICHLSIADLLVAFFTIPIEIGWRLTVQWIAGNYACKLFLFLRAFG
LYLSNNILICVSLDRYFAVLYPLRVNDARRRGKFMLSVAWFFSVLYAIPQSIVFHVENHPHH
KNFTQCVTFGAFPSDLVENTYNVFCVLTMYFIPLAIICWVYLKILCEISSKSRDNKPAGSNG
TLESSNSNQGSRMRLRRSDMSSIERARSRTLKMTIIIVVAFIFCWTPYITMNLWYVIDKKSA
KEVNEMVQESLFIMAVGNSCANPLVYGSYAIDLKKECFRCFLPCTTTKSNADVNLIQRSLG
SKFQKPEMKSPGVSKQIVHSVCQAVHGFFKAGSGQTKSTNVCGKVLVPISPRLSVSTTSKG
VVVEKLPLHTIVS
>BmA28 ACP
MDESTQMDVTACNDTTCSDTTSTPEQNFVIGVYSILLVIGAVGNVAVLISLLRNRRRKSRVS
LLMTHLVIADMIVIFYFIPLEIGWRKTNAWLAGNVACKFLQVFRGFGLYLSSNVLVCISVD
RFFAIIYPLRLAIARKRSKMMLYVAWAFALLLSLPQSAVFRVMEHPQIPDFKQCVSFEAFSN
HQQELAYNVICLSAMYFVPLLVITICYLCIFYKISRNSKQNSEKEPPSNSRRVILRRSDQRPL
VRARRRTLRMTVTIVTVFACCWFPYATMTLWYMLDWESAMRVPKRLQDFFFIMAVSNSC
MDPLVYGSYTVDLRALILALRKIFCIRKEPTVLPGIKRPETITLVDQLRISQSRKRVRLSNPR
DDLTTPRTSSEPFAYRAHHSFSERAIIMKPTHSCDDFTLSSPKKWYSA
>BmA29 ACP
MKMVNLNFDYEDTKEVPTSEIDKKFWEYFDATSTEISNYSSADLVPLDQGPVLATYAILLA
IGGVCNIAVLVKLAKPRRRKSRVDMLMTHLALADVCVTCGVIPLEIGWKYTNAWLGGNF
LCKLLLVLRAFGLYLSSNVLVCISIDRFFAVIYPLRLPEAKRRSRQMLYCAWVGALACSLPQ
SMVFRVKHHPRVIGFEQCVSFDAFNSYEQEVAYNVFCMCAMYFLPLIVITVCYVCIFCEIR
KSSKELGDKYHSGLKPVRLRRSDRSLLERARRRTLRMTVTIVSVFALCWLPYAIMAMWY
MVDRESASKVSRRIQDLLFAMAVSNSCMNPLVYGSYTLDIRGALRRFLKKCCSSTTPEVK
GQAGSSSNKNANFDTPHITEPKNIRTRLGVRFAETSLTAVPERLEVPRAPRGPA
>NV10005-RA Crz
MYVLEEEPCLNIRNASGLISTNILRNSSCLGHAPQLTYGAYLRAVVLLSMTLLSFLANLATI
WSIKSNKRKSQNCSAIYSLILHLSVADLFVTVFCMGGEALWSYNVAWIWGNTACKAFKFL
QMFSLYLSTFVLVLIGIDRFVAVKYPMKTLNTAKKCNQLISFIWFISFILSTPQVVIFHVAQG
PFIEDFSQCVTHGFYTEVWQEQLYTTLSLIFMFIMPLTILITTYMSTVITIARSERLFKSELAN
SSSAHKTGDVNRRRLIHRAKTKSLRISVVIVVAFVLWWTPYYIMMIIFMFLNPDKHVSAD
MQKGIFFFGMSNSLVNPLIYGAFHLWPQKKNRKHR
>BmA21

MDNEGNSTILYDANIMYPSELTLRTEFNTDGNNMNVCAIWPIEKCIEILKLNDTKTDDILG
RSFIYNDTQLTCLEHAPVLTKTTVIRASVLSAMAFLSFIGNVATIISIRRSKRCRGRARPSWT
AIYSLIFQLSIADLLVTIFCIAGEAAWSFAVQWYAGNIGCKLFKFLQMLALYLSTFVLVLIGV
DRWLAVKYPMKSMATATRSGRLVIIAWVLSVILSIPQAVVFRVAKGPFFEEFHQCVTHGFY
TERWQEQAYTTLSLVFMFILPLIILVSTYVSTVRTIAQSEKVFKPEVRRQEKYFTPDMNRRR
LIDRAKMKSLRMSVVIVAAFLIWWTPYYVMMIIFTFLNPDKNQSEELLNGIFFFGMSNSLV
NPIIYGAFHLWPRKKRSYQHSDRESGGHHASILRRGDNNTSSVRLTTIRSLRSSAKYSNGQ
NISLL
>DmCG10698 Crz
MEDEWGSFDRLPSVPSASMDLETENEVVSNWSTLANFTRLVAGAAPEIVNYTLNMIDVG
VGMATDISNLSVSTTPLPAYAISNSSSLAHTNSRHEAPPMAEQVPEHVMDHAPQLSRSGLL
KVYVLAVMALFSLLGNLLTIWNIYKTRISRRNSRHTWSAIYSLMFHLSIADVLVTWFCIIGE
AAWCYTVQWLANELTCKLVKLFQMFSLYLSTYVLVLIGVDRWIAVKYPMKSLNMAKRC
HRLLGGTYILSLVLSLPQFFIFHVARGPFVEEFYQCVTHGFYTADWQEQMYATFTLVFTFLL
PLCILFGTYMSTFRTISSSEKMFQGSKLANYSTAKLPTQTNRQRLIHKAKMKSLRISVVIIIA
FLICWTPYYVMMIMFMFLNPDKRLGDDLQDAIFFFGMSNSLVNPLIYGAFHLCPGKGGKS
SGGGGNNNAYSLNRGDSQRTPSILTAVTQVDGTGGSSRQMRAFRQQSYYRSSSNGTAGPG
AAPFKEQVGLLHVGPGNGTPGGSVSSGATPQLIRKGSALLARQPSCLREQEHQQRLLLHE
KPSTLVVSYDSQRGGVGVGVASGLLDNNERVSSV
>BmA23
MDGSANTSQDDEADWPGNSTLDEYIAQNSTSDVYDTLYDVPTGVIVLLSFLYGSISVLAV
VGNFLVMWVVATSRRMQSVTNCYIANLALADIVIGLFAIPFQFQAALLQRWLLPHFMCAF
CPFVQALSVNVSVFTLTAIAVDRHRAIITPLSAHTSKRVAKVIIVFIWLLAFTLAAPMEMSW
EVVMEDEIDPGTKLVYKKPFCTASEFGSNSLAIYRLLLYIFQYVIPLCVITFAYVHMAMKL
WGARAPGNAQETRDANHMKNKKKVIKMLVLVVALFALCWLPLQSYLLLQSFFPSINEYR
YINVIFFCFDWLAMSNSCYNPFIYAIYNEKFKKEFKQRFTFGKKPNRFANDSYEDGQSYRT
RILSFRSTNDRCLYSTRKSINITPDDSLRLSTHSSVQYTNNQSRENGCECTKTEEAQARITAR
RYANMRMGCRHPNARKCFSKTNETDEMPIGDERVSELYIFPNSNIVEFRDISYDDKV
>DmCG10626 LK
MAMDLIEQESRLEFLPGAEEEAEFERLYAAPAEIVALLSIFYGGISIVAVIGNTLVIWVVATTR
QMRTVTNMYIANLAFADVIIGLFCIPFQFQAALLQSWNLPWFMCSFCPFVQALSVNVSVF
TLTAIAIDRHRAIINPLRARPTKFVSKFIIGGIWMLALLFAVPFAIAFRVEELTERFRENNETY
NVTRPFCMNKNLSDDQLQSFRYTLVFVQYLVPFCVISFVYIQMAVRLWGTRAPGNAQDSR
DITLLKNKKKVIKMLIIVVIIFGLCWLPLQLYNILYVTIPEINDYHFISIVWFCCDWLAMSNS
CYNPFIYGIYNEKFKREFNKRFAACFCKFKTSMDAHERTFSMHTRASSIRSTYANSSMRIRS
NLFGPARGGVNNGKPGLHMPRVHGSGANSGIYNGSSGQNNNVNGQHHQHQSVVTFAAT
PGVSAPGVGVAMPPWRRNNFKPLHPNVIECEDDVALMELPSTTPPSEELASGAGVQLALL
SRESSSCICEQEFGSQTECDGTCILSEVSRVHLPGSQAKDKDAGKSLWQPL
>PPU14025-RA TK
MTLEETLLQPVLQLHSGISSATAILNSNASNLLLDQLSPSSIGSTTYPGAYYEFDGFINGTGN
ASLFDDSDAQNSNKFILPWWRQIIWTVLFAGMIVVATGGNLIVIWIVLAHKRMRTVTNYF
LVNLSIADAMVSTLNVIFNYIYMLNSHWPFGNLYCKISQFIAVITICASVFTLMAISIDRYVAI
VNPLKPRMGKRTTLCIAVAIWAVGAVLSLPMLLFYTTFTHNFPNGEVRVICYPSWPDQNNS
GQSYNEYLYNVIFMILTYFLPIGAMTFTYARIGVELWGSQSIGEATQRQLDNIRNKRRVVK

MMMVVVIIFAVCWLPFHVYFIVTSYLPELTNEPYIQELYLAIYWLAMSNSMYNPIIYCWM
NSRYVIYVSTYTSEQV
>NV10406-RA TK
MTLEETLLQSVQPILNSSTSGLLLDQLSPSIIGSTTWPGAFYELDGFINGTGNASLFDDSDA
QNSNKFILPWWRQIIWTVLFAGMIVVATGGNLIVIWIVLAHKRMRTVTNYFLVNLSIADA
MVSTLNVIFNYTYMLNSHWPFGNLYCKISQFIAVITICASVFTLMAISIDRYVAIVNPLKPR
MGKRTTLCIALAIWAVGAVLSLPMLLFYTTFTHNFPNGEVRVICYPSWPDQDNSGQSYNE
YLYNVIFMILTYFLPIGAMTFTYARIGVELWGSQSIGEATQRQLDNIRNKRRVVKMMMVV
VMIFAVCWLPFHVYFIVTSYLPHLTNEPYIQELYLAIYWLAMSNSMYNPIIYCWMNSRFRR
GFAQFFSWCPLVRIGHEPALSRSEAVTSRYSCAGSPEIRARISRNGKSFSTVIES
>BmA24
MMLDELGPTVASNQSTSLADLDSFYVTFYDVENERYVNDSQNATEPFQSFILPWWRQILW
TVLFAGMVVVATVGNLVVIWIVLTNKRMRSVTNYFLVNLSVADAMVSTLNVTFNFTYML
NSNWPFGHFYCKFCQFIAVLSISASVFTLLAISVDRYVAIMSPLQPRLGKRATLGITAAIWA
WSSFISSPNLIYFTTENVSLPDGTIRCVCYSHWPDGMTTRSRLEYAYNVLFMVLTYFMPIIA
MTYAYSRVGVELWGSQSIGECTQRQLDNVKSKRRVVKMMIVVVVIFAVCWLPFHVYFVV
TSYYPDVVSYPHIQEIYLGIYWLAMSNSMYNPIIYCWMNSKFRRGFKQFFWCCGAFGGG
GLARHRALGPDRTDRSMRSLSPSRKNGTSM
>DmCG7887 TK
MENRSDFEADDYGDISWSNWSNWSTPAGVLFSAMSSVLSASNHTPLPDFGQELALSTSSF
NHSQTLSTDLPAVGDVEDAAEDAAASMETGSFAFVVPWWRQVLWSILFGGMVIVATGGN
LIVVWIVMTTKRMRTVTNYFIVNLSIADAMVSSLNVTFNYYYMLDSDWPFGEFYCKLSQ
FIAMLSICASVFTLMAISIDRYVAIIRPLQPRMSKRCNLAIAAVIWLASTLISCPMMIIYRTEE
VPVRGLSNRTVCYPEWPDGPTNHSTMESLYNILIIILTYFLPIVSMTVTYSRVGIELWGSKTI
GECTPRQVENVRSKRRVVKMMIVVVLIFAICWLPFHSYFIITSCYPAITEAPFIQELYLAIYW
LAMSNSMYNPIIYCWMNSRFRYGFKMVFRWCLFVRVGTEPFSRRENLTSRYSCSGSPDHN
RIKRNDTQKSILYTCPSSPKSHRISHSGTGRSATLRNSLPAESLSSGGSGGGGHRKRLSYQQ
EMQQRWSGPNSATAVTNSSSTANTTQLLS
>BmA32 NTL
MPPWAHNSWICVFSIMLIIAVGGNAIVIWIVIAHKRMRTVTNYFLVNLSLADLMMSALNCL
FNFIYMLHSDWVFGLQYCKISNFIANVTVAASVFTLTGISFDRFQAIVRPMRPRMSKTCSLI
AIGGIWLGGMVLATPYLLYSTTKEYKSRVGVKTACLLVWPDGMPDVSKMDFVYQIAFFIV
TYAVPMVGMSFFYTAMGRELWGSRTIGELTQRQLDSIKSKRKVVKMFILVIVIFGICWFPY
HGYFIYTHLDSSILYSRYVQHVYLGFYWLAMSNAMVNPIIYYWMNAKFRSYFRMAIMCR
WLEVMWRRRHPLDSPPECPSQSNTRSRSGFYSLTYRGMQRIKRKYSSRMGAPHASDPQR
PALAETVFAC
>BmA33 NTL
MDSDLDIQSFINCTQQIFGHEQKWDDLNVSEILDLLPKQILEDINLKITLGNCMGLGERPYS
PPWWGQLAWFIVFAVMLLLAVIGNTMVIWIVLAHRRMRTVTNCFLVNLAVADLLMATLN
GAPNFVFLVTANWPFGAVTCTASNFTASLTVSAGVFTLVAITVDRYVAIVKPLQHRLSRRV
VRAALFTVWIASAMLALPSLLYSDTYKKQYVNGEREICFIKWPDGSYPTSLSDYCYNLVF
LSVTYVLPMAVMVWAYAQMSAALTGRAIGECTLHQMQVVRAKRKVVRMFVLVVMVFA
LCWLPYHAYFVLVYHHQSLATAPFAQHIYLGFYWLAMANSMFNPLIYYWMSNKFRLYFR
LVLCWCWKSESATPNDLKKLEVKSYSVSQRHFRDVSSFSRA

>DmCG6515 NTL
MSEIVDTELLVNCTILAVRRFELNSIVNTTLLGSLNRTEVVSLLSSIIDNRDNLESINEAKDFL
TECLFPSPTRPYELPWEQKTIWAIIFGLMMFVAIAGNGIVLWIVTGHRSMRTVTNYFLLNLS
IADLLMSSLNCVFNFIFMLNSDWPFGSIYCTINNFVANVTVSTSVFTLVAISFDRYIAIVHPL
KRRTSRRKVRIILVLIWALSCVLSAPCLLYSSIMTKHYYNGKSRTVCFMMWPDGRYPTSM
ADYAYNLIILVLTYGIPMIVMLICYSLMGRVLWGSRSIGENTDRQMESMKSKRKVVRMFIA
IVSIFAICWLPYHLFFIYAYHNNQVASTKYVQHMYLGFYWLAMSNAMVNPLIYYWMNKR
FRMYFQRIICCCCVGLTRHRFDSPKSRLTNKNSSNRHTRGGYTVAHSLPNSSPPTTQTLLAV
LAQTLTQPKPQTQLLLSHHSPHPTQPSAAETKSQWKRSTMETQIQQAPVTSSCREQRSAQ
QQQPPGSGTNRAAVECIMERPADGSSSPLCLSINNSIGERQRVKIKYISCDEDNNPVELSPK
QM
>PPU03425-RA CCAP
MNFFIKQLAFADLMVGLISVLTDIVWRSTVAWYAGNVACKIIRFMQVVVTYSSTYVLVALS
IDRYDAITRPMNFSRSWCRARALVTAAWSISVLFSVPIIFLYEEKIVEGKNQCWIELGSPAN
WRIYMTVVCLTLFIIPAIIIGGCYMVIVWTIWSQSSALRHDPTRDTRRASSRGLIPRAKIKTV
KMTFVIVFVFILCWSPYIVFDLLQVYGHVPRSQTNIAVATFIQSLAPLNSAANPIIYCLFSTPF
CKTVRNMQAVSWFSGLCPSNRHHCFGTNTHGNSTRTTVTTSLTAHSSRRSGHISMLHPSSR
KRVMVSLV
>NV17097-RA CCAP
MDFQLPSETWVSASTWPIETALGGILDASIVGYLSTEVSTLNNISRIIERNITDEIDPFYFYQT
EQFTVLWLLFSVIVVGNTSVLVGLIFGKRRKSRMNFFIKQLAFADLMVGLISVLTDIVWRS
TVAWYAGNVACKIIRFMQVVVTYSSTYVLVALSIDRYDAITRPMNFSRSWCRARALVTAA
WSISVLFSVPIIFLYEERIVEGKNQCWIELGSPANWRIYMTVVCLTLFIIPAIIIGGCYMVIVW
TIWSQSSALRHDPTRDTRRASSRGLIPRAKIKTVKMTFVIVFVFILCWSPYIVFDLLQVYGH
VPRSQTNIAVATFIQSLAPLNSAANPIIYCLFSTPFCKTVSNMQAVSWFSGLCPSNPHLCFGT
NTHGNSTRTTVTTSLTAHSSRRSGHISMLHPSSRKRVMVSLV
>BmA26
MDADIMMEGFETDMTTPNYAVNPIMTPMTAADHNHTWENISNATTPGSTINVYYFYDAA
QFTVMWILFVSIVVLNSSVIAALLCTNARKSRMNFFIMQLAIADLFVGLTYVFPDILQKIIIA
WYAGEFMCKTVKFLQAVVMYASTYVLVALSIDRCDAITNPMNFSGSWNRARVLVVSAWL
ISVIFSIPLFILYEVKEVQGELQCWIDLGNPKRWRIWVTLVSMMIFILPALTIAACYAVIVLTI
WTKSKAVVMSPPISSRRTKTMRNGQIESDPDSRRASSRGLIPRAKIKSVKMTFVIVFVFVLC
WSPYIVFDLLQVYGHIPSTQHYSAIATLIQSLAPLNSAANPLICCMFSPYIYTSLRRVPPYK
WIWWLGRHKRAGRSTLRSRSDSTAHSDLLSSTHARRSHSVATILNRTRSSSVSRPQSEARK
TQLLVLASARG
>BmA30
MEEPCMNCSFFAAFNDTQNEGNYTGNVTFINKFYFYQSAQLAILWILLVTIVAGNATVVLA
LLLTKSRKSRMNFFIMQLAIADLLVGLISVLPDLIQRVTITWLAGSITCKMMKYLQGVVTY
SSTYVLVALSVDRCDAITHPMNFTGSWRRARALILCAWLLSFFFCIPMLLLFNEADIEGILQ
CWSSISKIQWRIWMTSVFVSLFVAPALIISACYGVIVVTIRQKSHRVLGRRATTTRQYSDDL
DSRRASSRGIIPKAKIKTVKMTFVIVFVFVLCWSPYMIFDLLQVYGYVPDTQVNVAIASLIQ
SLAPLNSAANPVIYFIFSNRIFVSLKNIPPYKWLTCLMKGNDSPAGNESRAHTELLTSSHRR
TRHDLTIRVKDDSVKFPKPRVQHRSSTNSRKVRLHLPDGQHQVNNNCHTVRRRDDTFL
>DmCG6111 CCAP

MLHLRLFDSSLYYTLASASESSGLASSTSTERSFNGTQGAGGVAVGGESLTPTDVAAVNLT
YFTPAISHVMLAPTTIATTTASATMVQIQTTAAPSHDLETGGNSTSSDPGEFDNLNSFYFYE
TEQFAVLWILFTVIVLGNSAVLFVMFINKNRKSRMNYFIKQLALADLCVGLLNVLTDIIWRI
TISWRAGNLACKAIRFSQVCVTYSSTYVLVAMSIDRYDAITHPMNFSKSWKRARHLVAGA
WLISALFSLPILVLYEEKLIQGHPQCWIELGSPIAWQVYMSLVSATLFAIPALIISACYAIIVKT
IWAKGSIFVPTERAGFGAAPARRASSRGIIPRAKVKTVKMTLTIVFVFIICWSPYIIFDLLQVF
GQIPHSQTNIAIATFIQSLAPLNSAANPLIYCLFSSQVFRTLSRFPPFKWFTCCCKSYRNNSQ
QNRCHTVGRRLHNSCDSMRTLTTSLTVSRRSTNKTNARVVICERPTKVVTVPAMSEV
>PPU12877-RA AVLP
MDVERLHTAACQRTLQKRIASPGIIMEESTTSMIVLPAEDWRDESLAVWEIIVLALILTTTL
MGNVLVLFAIYLKKCRGRRQRLTRMHFFVMHLSVADLITGLLNVLPQLAWDVTFRFQGG
PILCKLVKFCQPLGSYLSSYVLIATAVDRYHAICYPLSYCRTTSRRSRITVYYVAWLLALLLCV
PQVFIFSYQEISAGVWDCWATFTVPYGERAYVTWYTVTVFLLPFCVLTFTYAEICCSIWRN
REVMVLASHERQQALTKEGRSQTSLISKAKINTVKQTLAVVTLYAASSIPFVGCQLWATWD
PVASTSAFFDGPIFTILSLLSSLTSCVNPWIYLTFSYELRAALTKFLRSLIKRDRSSRFERASSN
ANSNETRSSKRSSFISRMSRYTSFIIYRSNANDRNSK
>NV17951-RA AVLP
MEESATSMIAAPAEDWRDESLAVWEVIVLALILTTTFMGNVLVLFAIYLKRCRGKRQRLTR
MHFFVMHLSVADLITGLLNVLPQLAWDVTFRFQGGPILCKLVKFCQPLGSYLSSYVLIATA
VDRYHAICYPLSYCRTTSRRSRITVYYVAWLLALLFCLPQVFIFSYQEISAGVWDCWATFTVP
YGERAYVTWYSVTVFLLPFCVLTFTYAEICCSIWRNREVMVLASHERQQALTKEGRSQTT
LISKAKINTVKQTLAVVTLYAASSIPFVGCQLWATWDPFASSSAFFDGPIFTILSLLSSLTSCV
NPWIYLTFSYELRAALTKFLRSLIKRDRTSRFERASSNANSNETRSSKRSSFISRMSRYTSFII
YGPMRTIEIVNKLYI
>TcAVLP
MYTPKLSQMDISENSTYLFDKHEDRNNTDRDENLARVEVATLAIIFLVTVIGNSTVLLALW
TRRRYAGRKKLSRMYFFILHLSIADLITAFLSVLPQLAWDITYRFYGGFLLCKVVKYGQTL
GPYLSSYVLMATAIDRHQAICYPLTYCSWTSRRSKVMVYLAWVASLAFCIPQLTIFTYTSV
GEDEYDCWATFQEPWGKRAYVTWYSISVFMVPLVVLIFTYTSICIEIWQSSESSLRPRSSQK
SAPGKRTPLISRAKINTVKQTIAVIVMYIACSTPFILAQLWATWDPQSPFIDGPVFVILTLLYS
LNSCVNPWIYLAFNRELPRLLLRHYTASSKNYRSATGGNSASNSSGDAQSTSLRPFSRWSL
CNSARSNKYPTRVPHRPYVAQYNARRWIVTTTT
>BmA31
MIDGTSSNMHHVRLLIGVEEKAMWSSLNKTTSGPEDVNVSRINETGPLYNEDEYIFDRTD
VRAIFITLYTIVFCCCFFGNLLVILVVTLSRRLRSITNFFLANLAVADLCVGVFCVFQNLTIYL
IPSWIFGDFLCKMYQFVHSLSYTASIFILVVICTERYFAIIHPITCKQILTSTRLRLVILGVWITS
AAYSAPKFIWVETITNDLGNGQMETICIPHRRKYNSEIFDMVNFGLLYVTPLCVMTVLYTR
IAVGLWQSSHTLQSLGRVQCCATECPRPEKPRTANYEPQRTFVGATENKNGVSTKVKGPPA
KNVRTYEPRDCHHLSHLSRNVLRARRGVVRMLIVVVLTFAICNLPFHARKMWQYWSSGY
EGSSDFSTLLTPLTFLITYFNSGINPLLYAFLSKNFRKGMKELLFCNYRAKRKNDQIIVLNPV
GGGVLRRSSTRSTRANCSTITMAANGDS
>DmCG14003 TR
MIMTMMQTVRAWQQESDVEHRKQHKQRWRPDGAHISAAYDLNSDNDDGHHRVVHNQ
NNGSPNSSPNQSTSAFRQRQPHHPPTGQQPPRLPCTVTHFSAHWKTLLLILLTLLSASTLTAS

ANVTSTISPPINGSSTDYILLYGESTTSLVPALTTGLSGDGSGAVIEDEEDAEKASEYIFDRTD
VRIIFITLYTLVFCCCFFGNLLVILVVTLSRRLRSITNFFLANLAFADFCVGLFCVMQNLSIYL
IESWVFGEFLCRMYQFVHSLSYTASIFILVVICMERYFAIVHPITCKQILTAARLRMVIVTVW
ITSAVYSTPKFVFSKTIKNIHTQDGQEEEICVLDREMFNSKLLDMINFVLLYVMPLLVMTVL
YSKIAIALWRSSRGLTPHVVQHQHQQPQQPSCQDIGMGMHNSMYHHHPHHHHHHHQHH
QLQSAASSAGVVGVGLGGGGGGGPGPSLASGGSSTTSLSRKQSSKYEKRGVSITESQVSL
EADRPIVSACRKTSFYHHGHAHHQRAGNASVGGGSGGAGAGATHMSHSSSNVLRARRG
VVRMLIIFVLTFALCNLPYHARKMWQYWSRSYRGDSNFNALLTPLTFLVTYFNSGVNPLL
YAFLSRNFRKGMKELLLCSWKKGKGKSSSNSSMHHKRKALQTHSLPTDTTHIGNEQL
>Hheb007010.1 Orphan
MAVAPINKILTALATADMFVMIEYIPFSIYYYIIFPNRATFPYFGAVFVLFHMHFAQLLHTISI
ALTLSLAVWRYLAIRFPQHNHAWCSDARCKMALWCSLAVSAFACSPSYFVFEIHEQPVQE
NGITEILYYVNADSYSGSGIAYQINFWVLAVVVKLLPCLILTVISCWLIKELYSYALFLLALF
RAIYSKEPGEENKGEIME
>Hheb077040.1 FMRF
MKSSINYLLIGLARCDTVLIITSVLIHGLPAIYAYTGLLFDYKFGVFPQIVRYLYPLSCMAQM
VTVYLTLTVTMERYVAVCHPLRARAFCTYGRARLAVLSIVIVSIIYNMPKFWEVDLEKEIH
WKYNVTVYCVVPAILRSSDLYITIYVNWMYFFVYYAFPFVALVVFNVAIYRR
>PPU04456-RA RFaR
MTLEPVFNWTTSEDNNSSSLVGNLSIVATSLGPPKECELPVNDGFLEFFVYGILLNVVSFLG
ILGNAISIIVLSRPQMKSSINYLLIGLATCDTILILLSVLVYGLPGIYAYTGYLFHYKFFVYPKI
VRYLYPLSTTAQMATVYLTLTVTMERYVAVCHPLKARSLCTYGRARTALLLIALISILYNLP
KLWEVRLGEEIHWRYNITVYCIGATQLRENSLYKTIYVHWLYFFVYYAFPFVALVIFNVAIY
KRVRKANRDLRRLSRHQRREIGLATMLLCVVIVFLVCNVLPLVSNVYENMYVNPPGWMI
QMGNLLVTINSGVNFVIYVIFGRKFKRIFLKIFCRVSGETGLDCMGRPSRADSPDFQTNEDS
IATNLSNVELRNSIRRLHGSHYHHHHGSHHGSNSLRPGGGSTHSGGSNGFSRSSVYYPTSP
KPGFHNNGSNHSQHNNHQHHHRDRSTNLEDTTFC
>PPU05580-RA MS
MSNASAGYNFTCGSGVDSFHTSYVAIHGWASLLVCIFGSIANGLNIAVLTRREMSSPTNAIL
TGLAVADMLVMIEYIPYAVHSYLYHRPKRETYTYAWTAFVLFHSNFAQVFHTISIWLTVTL
AVWRYIAVAHPQKNREWCSYNRTILAIVAAYVICPLICFPLYVTTEVTSKNVTLDANDRQV
NLTGNGSSLRYLQSGAEANNATLYFVALTESAQNGLKEMNFWMYSVVIKLIPCVALTILSL
RLIMALVEAKKRRKKLTSTTMLKMEESINLAESKKRKRKASRMMDKERQTDRTTKMLLA
VLLLFLLTEFPQGTLGLLSVVLGPDFFNTCYVKLGEAMDILALINSAINFILYCAMSRQFRT
TFKQLFCRWKLLGRWLPVPQHAENNNGNMTTNHTVTQVTQV
>PPU04714-RA MS
MSETAQPGQPAGQHPQEQPSAHQLSNYTELLRRLNITEEDLDYVNNFGSSAGIGGDTGGG
CSCGPCHCGGLVRRIAASYRSYHGYVALLVCGFGTLANLLNVAVLTRKELRRAPINRILTG
LAAADVLVMLEYVPFAIYEYIVLPERRHFPYGWAVFVLFHMHFSQLLHTISIALTLTLAVW
RYIAVRFPQCSRSWCTPARCRLALLCSLLVAGLACAPSYLVFGIREQRLMEENGPVVLYHV
DASRGPSSDDDADDRGLLYRLNFWLLGVLVKLLPCFVLTLISCRLIQALYKAKARRRLLRP
LDGQLTDAPATGGRSERRSDRTTRMLVAVLLLFLITEIPQGVLGLLSALLGDCFFRSCYHSL
GEIMDILALFNGAVNFILYCSMSRQFRTTFGRLFKPSIVVGKWQPACTHQTDIQSTYV
>PpCNMaR-2

MNALVSLMAQRGPARCGWLLAQGRVRRAIAFQNETDAVLEQRLEDQPVVPHHQLPARN
HSYEHGFETGFETGFETGIEAYEEEPGDECHSLYFVLDFSHQYYIPFIILLGLVGNLLSCIVFL
NTHLKMRSSSYYLAALATADFSFLFSLLLVWLNNSIGWRVFNKDGWCETVVYVSSVCSSL
SVWLIVAFTVERFIAVQYPLHRPHMCTIARAKTIVLALVVLAMASHSYSFVTAGVVKTQD
GNEMCDMKYEYLETMRIVSIVDSIASLMAPLVLIIVMNTMITRNLIRFSRRFGESSSGGLST
ETRCPSRERSDINLNPIPRITCRFSDVRSSSRNLVSTRNQQSITKMLLLISTVFILLNLPSYVIR
LCVFFFTLARKDTPSLLWCLQQFFMLLYYTNFSINFLLYAMCGITFRRCLEQLLRKALKSLT
RYHCNPQRYI
>PpCNMaR-1
MSRSNNSTTTSTMEGENGYSFSYPFAWSLINGLEMYYLPALVVFGSLGNCLSVYVVFATK
MRRTSSSFYLAALALSDTGFLMSVLIAWLTLINVHIMNQQGFCQFFVYLTNICSFLSAWCF
VVAFTVERFVAVCYPLRRQWMCTVNRAKILISCVTAAGLILCSPVLLYSRPRLLENSTKPV
CYIAEGWDKVASALNFLDTILTFALPFSLIVVLNSLIVRAVWHVDSVRTSLKARASTKDSPP
AVHQARVTKMLLVVSSVFFCFNLPAYAMRVYAYLQADGAATEVEFLAQKACNLLFNTNF
GISFGLYCASGQNFRAAVAQLFHCQRQLKLRPGMSKRSSTNQINHHGGTETIRISGSVSGD
HTIVFEKPWRDSFQLRPYPRSLQRPVADCESAEDCNTPRKDYSAQM
>NV22020-PA CNMaR-2
MNALVSLLAQRGSARCGWLMDESRVRRAISFQDETEASLGQPADGRPATSHHQVPARNRS
FEAGIEAYEEEAGDECHSLYFVLDFSHQYYIPFIILLGLVGNLLSCVVFLNTHLKMRSSSYY
LAALATADFSFLFSLLLVWLNNSIGWRVFNKDGWCETVVYVSSVCSSLSVWLIVAFTVER
FIAVQYPLHRPHMCTIARAKSIVLALVVLAMVSHSYSFVTAGVVKTQDGNEMCDMKNEY
LETMRIVSIIDSIASLMAPLVLIVVMNTMIMRNLLRFSRRFGQNSSGGLSTETRCPSRERSDI
NLNQIPSGSSSNNGANGIALSSGIGARRQPSQQSFQSSKNNHSNHSRTQQQQQQQQQQQQ
TTTTSPPQAQQAPSTIVPPSTPRNAYVLPEITSRCIHVRSSSRNLVSTRNQQSITKMLLLISTV
FILLNLPSYVIRLCVFFFTLARKDTPALLWCLQQFFMLLYYTNFSINFLLYAMCGITFRRCLG
QLLRKILKSLTRYHCNPQRYI
>NvCNMaR-1
MSRSNDSTTAAAMEGENGYSFGYPFAWTLINGLEMYYLPALVVFGSLGNCLSVYVVFATK
MRRTSSSFYLAALALSDTGFLMSVLIAWLTLINVHIMNQQGFCQFFVYLTNVCSFLSAWFV
VAFTVERFVAVCYPLRRQWMCTVNRAKILISCVTAAGLVLCSPVLLYSRPRLLENSAKPVC
YIAEGWDKVASALNFLDTILTFALPFSLIVVLNSLIVRAVWHVESVRTSLKARASTKDSPPA
VHQARVTKMLLVVSSVFFCFNLPAYAMRVYAYLQADGAATEVEFLAQKACNLLFNTNFGI
SFGLYCASGQNFRAAVAQLFHCQRQPKLRPGISKRSSTNQINHHGTETIRISGSVSGDHTIV
FEKPWRDSFQLRPYPRPLQRPVAVCESIEDCYTPRKDYTAQM
>NV11638-RA FMRF
MTLEPVFNWTTSEDNNSNGSSVGNLSIVATSLGPPKECDLPVNDGFLEFFVYGILLNVVSF
LGILGNAISIIVLSRPQMKSSINYLLIGLATCDTILILLSSVLVYGMPGIYAYTGYLFHYKFFVY
PKIVRYLYPLSTTAQMATVYLTLTVTMERYVAVCHPLKARSLCTYGRARTALLLIALISIFY
NLPKLWEVQLSEEIHWRYNITVYCIGATQLRENPLYKTIYVHWLYFFVYYAFPFVALVIFNV
AIYKRVRKANRDLRRLSRHQRREIGLATMLLCVVIVFLVCNVLPLVSNVYENMYVNPPG
WMIQMGNLLVTFNSGVNFVIYVIFGRKFKRIFLKIFCRVSGETGLDCMGRPSRADSPDFQT
NEDSIATNLSNVELRNSIRRFHGSHHHHHHGSHHGSHHGSNSLRPGGGSTHSGASNGFSRS
SVYYPTSPKPGFHNNGSNHSQHNNHQHHHRDRSTNLEDTTFC
>NV17617-RA SP/AstB

MSNASAGYNFTCGSGVDSFHTSYVAIHGWASLLVCIFGSIANGLNIAVLTRREMSSPTNAIL
TGLAVADMLVMIEYIPYAVHSYLYHRPKRETYTYAWTVFVLFHSNFAQVFHTISIWLTVTL
AVWRYIAVAHPQKNREWCSYNRTIMAIVAAYVICPLICFPLYVTTEVTSKNVTLDANDRQI
NLTGNGSSHYYSGRSGADANNATLYFVALTESAQNGLKEMNFWMYSVVIKLIPCLALTILS
LRLIMALVEAKKRRKKLTSTTMLKMEESINLAESKKRKRKASRMMDKEKQTDRTTKMLL
AVLLLFLLTEFPQGTLGLLSVVLGPDFFNTCYVKLGEAMDILALINSAINFILYCAMSRQFR
TTFNQLFCRWKLLGRWLPVPQHAENNNGNMATNHTVTQVTQV
>NV16129-RA SP/AstB
MNEAALQLGQHQQPLAHQLSNYTELLRRLNITEEDLDYVNNFGSSAGIGGTGGTGGTGG
GCSCGPCHCGSLVRRFAASYRAYHGYVALLVCGFGTLANLLNVAVLTRKELRRAPINRILT
GLAAADVLVMLEYVPFAIYEYIVLPERRHFPYGWAVFVLFHMHFSQLLHTISIALTLSLAV
WRYIAVRFPQCSRSWCTPARCRLALLCSLLAAGLACAPSYFVFGIREQKLMEENGPVVLY
HVDASRGPSSDDDRGLLYRLNFWLLGVLVKLLPCFVLTVISCRLIQALYKAKTRRRLLRPL
DGQLTDTPATGGRSERRADRTTRMLVAVLLLFLITEIPQGVLGLLSALLGDCFFRSCYHSLG
EIMDILALFNGAVNFILYCSMSRQFRTTFGRLFKPSIVVGKWQPACTHQTDIQSTYV
>BmMSR
MSEDESYGFCSESATAFQRVYIQAHGYIALIICLLGSAANSVNIAVLSRKEMTSCTNSILTGL
AVADLLVMIDYIPLALHLYTKIGSELNQNSYGWAVFIYFHSIFSQTFHTISIWLTIMLAVWRY
IAIKFPQKNQTLCNKRNTTLAIILAYAVCPVLCLPIYFAMNIKERLPSDTSNGTNVTLNYTSA
ANVSDPKQFIIEMTNNNDLLTAIFWIYSVFIKLIPCVVLSILSVLLIMKMKSSDRRRQKLLKK
SAITTTEGEKARLNDDGKKGGGRTDRTTRMLVALLGLFLATELPQALFGLLTAIAPHLFLIC
YYAFGEVMDLMALVGSAVNFVLYCSMSRQFRTTFTRLARKVLPLPQRQREREPLTTVTVS
>BmA3
MTIEAAPNITQTLTSLFEEMLLETEASNHNESNLDLSHFLSALRVQSNSSNHEPLDFGTLIRL
VDGFRNKLNLSRPIEPACTYCDGNIRDVILAYNSIHGYISLIVCFFGSLANTLNVAVLTRRDL
AAAPINRLLKWLAVADVFVMLEYVPFAIYRYLILPGQREMPYKWAAYLLFHMHFTQIFHT
ASICLTLSLAVWRYVAIKYSDKNHILCTERRCSTAILSSFIIPPVLCIPTYMVFDIHTAVVLEPT
GPMILYHVDSDEEGGLYQINFWVHAVLIKLLPCCLLTVISLWLIREVYSANQHQKKIRVYN
ACPSNNKAIKRQYKADRRTNRTTKMLVAVLLLFLVTELPQGILGLLSGILGRCFFKRCYDL
FGELMDALALLNGAINFVLYCSMSRQFRMTFGQMMWRAHLHRWSPPQASHSDGQTTAK
SSVP
>BmA8
MSNKPQSLLDIFQEIFAASGNSSDSYNETQILSMLREQTNRGGAGEEFESLLVKMMKDAK
SKLNLTLKPDTCGYCEGDFRDVITVYNSIHGYVSLLVCTIGVLANSMNIAVLTRRDMAAAP
INRLLKWLAVADVFVMIEYMPFVIYRHLVLPEKLDFPYSWAMYLLFHMHFAQILHTASICL
TLSLAIWRYIAIKYSDRSHILCTERRCSIAILTSFILPPILCTPTFMVFDIHTKNVTNADGNPDI
AYHVDSDYQGTLYQANFWVHGVVIKLLPCSILTVISIWLIKALYKANQHQKNLRNYSACP
AAEKMVKRQHKADKRTDRTTKMLLAVLLLFLVTELPQGILGLMSGLLGWCFFKRCYDLF
GELMDFLALLNGAINFILYCTMSRQFRQTFRQMLLQPPLARFLPPTASHSESHNQNTCTEKI
KSMKTSIP
>BmA13
MANDTDAYCVPGATNFNKAYSRLHGYIALVICIVGSATNSINIAVLSRREMSSSTNSILTGL
AVADLLVMLEYIPYALHMNIKIGPQVNKNTYAWTVFVYFHSIFSQTFHTISIWLAVTLAVW
RYVAIAFPQRNRTWCNRKNTITAIVSAYVICPFLCLPIYFAMTIVPSEVTSKNNSEFAEDLAL

PQNRTVYLLEMSKNVELVTAIMWIYSVILKLVPSIALSILSTCLISKLTTTERRRQNLLKRST
VGPNEPEKQCLADDSNTRRSSRTNRTTRMLLAVLGLFLSTEVPQGLLGLASAVAPDFFKNC
YSMFGDLMDMLALFTSSVNFVLYCSMSRQFRCTFARLARRMLYAAEEPAKFAAKLEPTTQ
VTGPL
>BmA20
MLSENETRSSYDTQTNLSHISGNLFEDASINSLLNHGGNYTTNIGQVLKVLEDWRNRFNIT
VVKECDGTEYCAGEFRDLIIAYNSIHGYVSLLVCLFGSLANAFNVAVLTRRDLAVAPINRLL
KWLAVADVFVMIEYVPFAIYRYLLLPGQEDRPYSWAAYMLFHMHFTQIFHTASILLTLSLA
VWRYLAIKYPAHSPILCTDRRCTVAIMLSFVLPSILCIPSYFVFTIHKDFSYDRNANVFSKVY
FVDSDFDGYLYHINFWVHAVLIKLLPCVILTIISAWLIRALYRANYRKKILKGYNACPAETI
VNGKGNIFTRRSTKRSKIERRTDRTTKMLVAVLLLFLLTEFPQGILGLLSGILGRCFFKHCYN
LFGELMDALALLNGAINFVLYCSMSRQFRTTFGQMLRTRCAKTYRAGSQTELQTTYV
>Dm_CG13802 MS
MVTNMSQPHYCGTGIDDFHTNYKYFHGYFSLIVCILGTIANTLNIIVLTRREMRSPTNAILT
GLAVADLAVMLEYIPYTVHDYILSVRLPREEQLSYSWACFIKFHSVFPQVLHTISIWLTVTL
AVWRYIAVSYPQRNRIWCGMRTTLITIATAYVVCVLVVSPWLYLVTAIAKFLETLDANGKTI
ASVPLSQYILDYNRQDEVTMQVMSSTTPDVSWAIPSDSANGTAVSLLSLTTVIPLTTLSTGV
TTSSSLGERNVTVYKLYHSALALRDRQFRNATFLIYSVLIKLIPCFALTILSVRLIGALLEAK
RRRKILACHAANDMQPIVNGKVVIPTQPKSCKLLEKEKQTDRTTRMLLAVLLLFLVTEFPQ
GIMGLLNVLLGDAFFLQCYLKLSDLMDILALINSSINFILYCSMSRQFRSTFALLFRPRWLD
KWLPLSQHDGEGRVGGSGGLGGYGGYGRQRLLHTDAVSKSMAIDLGLTTQVTNVXQESS
GRAAMSAAAGGAAASVALALAATDVDGCPPATDAAVSTNDISLVEKLHLQPSPRGTAISS
GQHRRRRSGSGTKCIWPTTDWLRKLRNQKARETEQSSEQDIELGKSSINRRSSVLLMVLL
SSSDEVKAKAVLVSEQPPSPADEDVEDAIDALWL
>Dm_CG8985 MS
MASGNNETEPLYCGSGMDNFHTSYKNMHGYVSLVVCILGTIANTLNIIVLTRREMRSPTN
AILTGLAVADLAVMLEYIPYTIHDYILTDSLPREEKLSYSWACFIKFHSIFAQVLHTISIWLTV
TLAVWRYIAVGYPQKNRVWCGMRTTIITITTAYVVCVLVVSPSLYLITAITEYVDQLDMNG
KVINSIPMTQYVIDYRNELLSARTAALNATPTSAPLNETVWLNASTLLTSTTTAAPPTPSPV
VRNVTVYRLYHSDLALHNASLQNATFLIYSVVIKLIPCIALTILSVRLILALLEAKRRRKKLT
SKPATPGASNGTKSPANGKAADRPRKNSKTLEKEKQTDRTTRMLLAVLLLFLITEFPQGIM
GLLNAVLGDVFYLQCYLRLSDLMDILALINSSINFILYCSMSKQFRTTFTLLFRPKFLDKWL
PVAQDEMAAARAERSAVAPVLEKGRQQPQVVMASTTTNITQVTNLXHRRSRGRRTLLSR
LLSVLKRGRRRSSGEGGGVGGGGAPLAGNDAVEPAFQAIVVVVDKVSGATENQLYTAEQ
ARIVT
>BmA18 CNMa
MCPLPANETDYYLESSEYFNATSSYISAVNETFFDIATENIATLLNVYYTPLLVALGCIGNLL
SVFVFYRTKLRLQSTSQYLTALALSDTVFLFQLIPPWLNAVEVTGLFYKHGFCQIFVYVTY
VSCCMSSWLVVAFTVERFVAVLYPLRRNALCTVTRARHIIFTVLLASSLINLPVLRFAVPSK
NDCNIDFEYLDQAARFNLVDTALSFSVPLFLITILNTWIMIGVWKLERQRRQIMKEEMERA
RARPYRTTGCPRSQHRVTRMLLIVSSVFVVLNLPAYTMRILAYAYNMNEEEYSGRWAAVQ
QLSLLFFNTNFGINFMLYCLSGQNFRRAVQQTLPCLRKRADHRAARRATQTRPGSGSSK
>DmCG16726 CNMa
MDMEYITSSSGNITATTEADFSSSLGESNVTEYNTTEMDANESAGEDEEMLRIAFFIGHFV

HQYYIPVLCCTGSIGNILSVFVFFRTKLRKLSSSFYLAALAVSDTCFLAGLFAQWLNFLNVD
IYNQNYFCQFFTFFSYLASFCSVWFVVAFTVERFIAVIYPLKRQTMCTVRRAKIVLFCLTLV
GCLHCLPYIVIAKPVFMPKLNTTICDLNSEYKEQLALFNYWDTIVVYAVPFTTIAVLNTCTG
CTVWKFATVRRTLTMHKMKPQTNSMPSNSSNSSGGASSAVASYRLSASLKRQKSTGTHPS
GQHNVANRQTDDQEQQQQSQQHQINNCQHHCEITQKPARRKVQNSSQLKVTKMLLIVST
VFVCLNLPSCLLRIEAYWETESARNQNSTIALQYIFHAFFITNFGINFVLYCVSGQNFRKAV
LSIFRRVSSAQREAGNTQVTVSEYCRNTGTSTRRRMMTQHCWNEMHELHPLK
>DmCG6986 Pro
MTMSSTSTATATSTATATLDEANATVGEMFSDADMAEVRHVVQRILVPCVFVIGLLGNSVS
IYVLTRKRMRCTTNIYLTALAITDIAYLTCQLILSLQHYDYPKYHFKLYWQLYGYFVWLCD
SFGYISIYIAVCFTIERFIAIRYPLKRQTFCTESLAKKVIAAVAIFCLLSTLSTAFEHTITIGTRQI
DDAYQPCNQTVANISPMPPPPVAVTPPLATPPLPTPATIWQSPDSAMESTTSGSSNQLVDWG
SGSGDGEPENIPRHRRHWQSSGFVTLPTLRKTLEEQDQKVADAAQRSGVTESLLQLWRRK
RSAENHNINNTDAFAFNVTEYCQNVTYYNHGLSELGYDELYSYLWNLFTLLVFVVFPLLL
LATFNSILILLVHRSKNLRGDLTNASSIRRTKRKSNSGLKGSVSQENRVTITLIAVVLMFIVC
QLPWAIYLIVNQYMEIQIGTQVVAGNVCNLLASLHAASNFFLYCVLSDKYRKTVRELITGY
RYRRRHARNNTSLYVPHTTTTLTQINGDHYGSNYGGAGSRRNRNTGRLIA
>BmRFaR/FMRF
MNGTEGEGCDSDVGVQPADKLFRFVVHGVLLNAIGAAGLLGNALSVVVLSRPQMRSSIN
CLLVGLAACDTVLILTSVLLFGLTAVYPYTGRLRYYYYHVCPHITPYAYPIANAAQTMSVY
LTLIVTVERWVAVCHPFRAKSLCTSSRARWYVLGTAAFALAYNAPKFLEAEVVTRSVDGE
PVYCVTADLHFRTETYIVVYIHSLYMIVMYIVPFSALAALNACIVRQVRRAQAERARLSR
VQRRELGLATMLLVVVLVFFLCNLLPLVTNSFEVFLGDQLENLDPLVKTSNLLVTINSSVNF
VIYVIFGEKFKRVFLKMFCAGGWRRRTRDSPEQTRDDSFASCGERVSLRLVRNGTLRRSEP
RAPPRGRSRRAPSPSVYYPAPLTDVTSALSVSEPPPAAMRWNGHTRSHF
>DmCG2114 FMRF
MSGTAVARLLLRLELPSPGVMPPPPTDYDYGGPISDDEFLASAMATEGPTVRYDLFPQNNS
QPTLQIVLNHTEVQTDLQYPHYEDLGLDPDPNWTRICEDVYNPLLENNRIEFWVCGVLINI
VGVLGILGNIISMIILSRPQMRSSINYLLTGLARCDTVLIITSILLFGIPSIYPYTGHFFGYYNY
VYPFISPAVFPIGMIAQTASIYMTFTVTLERYVAVCHPLKARALCTYGRAKIYFIVCVCFSLA
YNMPRFWEVLTVTYPEPGKDVILHCVRPSRLRRSETYINIYIHWCYLIVNYIIPFLTLAILNC
LIYRQVKRANRERQRLSRSEKREIGLATMLLCVVIVFFMLNFLPLVLNISEAFYSTIDHKIT
KISNLLITINSSVNFLIYIIFGEKFKRIFLLIFFKRRLSRDQPDLIHYESSISNNGDGTLNHRSSG
RFSRHGTQRSTTTTYLVATGGPGGGGCGGGGGNNSLNNVRLTQVSGSPGLVKIKRNRAPS
PGPVVYFPAREMQRSASTTNSTTNNNTSIGYDWTLPDSKKLGHVSSGF
>BmSPR SP/AstB
MAVTIDNSTNDFEFQKPFNYSINENITYFDYTNFTSDDFCASNNSHVYLNVTCEFAISYAEP
MYGYIAPFLLATTTVANTLIVVVLSRRHMRTPTNAVLMAMALCDMFTMLFPAPWLFYMY
TFGNHYKPLSPVRACQAWNYMNEVIPAMFHTASIWLTLALAVQRYIYVCHAPVARTWCT
MPRVMKCLIYIGIAAFLHQLPRFFDRCYTPHKTVWRGRVEEVCRIEMASWVKALSVDAYF
ISYFGFRVLFVHLIPCTSLVVLNVLLFRAMRTAQINRQKLFKENRKSECKRLRDSNCTTLM
LIVVVTVFLLVEIPVAVVTILHIISSTIVEILDYHIANILVLVTNFFIIVSYPINFAIYCGMSRQFR
ETFKELFIRGTVTSRKNGGSSRYSLVNGPRTCTNETVL
>Dm_CG16752 SP/AstB

MDNYTDVLYQYRLAPSASPEMEMELADPRQMVRGFHLPTNESQLEIPDYGNESLDYPNY
QQMVGGPCRMEDNNISYWNLTCDSPLEYAMPLYGYCMPFLLIITIISNSLIVLVLSKKSMAT
PTNFVLMGMAICDMLTVIFPAPGLWYMYTFGNHYKPLHPVSMCLAYSIFNEIMPAMCHTI
SVWLTLALAVQRYIYVCHAPMARTWCTMPRVRRCTAYIALLAFLHQLPRFFDRTYMPLV
IEWNGSPTEVCHLETSMWVHDYIGVDLYYTSYYLFRVLFVHLLPCIILVTLNILLFAAMRQ
AQERRKLLFRENRKKECKKLRETNCTTLMLIVVVSVFLLAEIPIAVVTAMHIVSSLIIEFLD
YGLANICIMLTNFFLVFSYPINFGIYCGMSRQFRETFKEIFLGRLMAKKDSSTKYSIVNGAR
TCTNTNETVL
>CG13229 Orphan
MMQETGNQMGQTHMHQRVPFNDTVLKDYHLTSTDIEKFVKLWQEYQMKNMTPQVDEC
QGYCQGEIYNWLRAYNSIHGYVSLMICIFGTIANILNIMVLTRKEMAKTPINNILKWLAVA
DMFVMLEYIPYTSYQYIYMGPGEKDLSYTWAVCLLVHMHFTQILHTISIGLTVTLAVWRY
VAIRHPNGGCANFLLAHSREAILLPFILSPILCLPTYFVFQVRETYDVDKVNSEAMYHVYF
DKDSVLYRFNFWIHSVLIKLLPCGILIVISAVLMHVLCEASRRRLKLRDYNNPAKYAIQLNL
NETKSKKPPRCDRRNDRTTLLLVAVLVLFLITEFPQGLLGLLSGVMEKCFFAHCYPPFGEL
MDLLALINAAVGFVLYGLMSKQFRTTFRSLFMKRHFGSTEMTRLTRVTTTCV
>CG33639
MITRLYNTEEDPAYCSFIWGSNLTSSVDVLAANATSVFSSDLRDDFYRDVEDPRTESLREY
CYGLVLPIICAMGIIGNVLNLVVLTRRNMRGTAYIYMRAYSTAALLAIVFAIPFGIRMLVHK
DRGQWEEFGPAFYTAHLELYLGNGCLGVGVMMLLVLTIERYVSVCHPGFARPVMGPPGV
VVFLTCLATVIVYLPSIFRGELIKCILGSSDVYVYLRRDNTIYQQTIFYRVYKIMLEVIFKLVP
TLVIGGLNMRIMMVYRRTCERRRKMVLSRPHAQGHGHGHGHGHGHGHGHAHGHGYLK
DDDPRKFAEERRLFLLLGSTSILFLVCVSPMAILHMTIASEVYPSFPFQVFRASANLLELINY
SLTFYIYCLFSEDFRNTLVRTIKWPWLKGKFCHQAEHENPTNGPGVPMACFTKVDRGHQK
HHITTTSGLGRSSSI
>RPRC005858+RPRC001892 Serotonin
MEWENTSNILWNITTILGNNSNITADDLERWSKHLQPTTAVTTIQQPTQQHSSHRDWTFLF
VLLFVAAGGLGNILVCLAVCLDRRLQNVTNYFLLSLAVADLLVSLFVMPLGAIPGFLGKQI
CKCFYLPQQYKDLEDGIVSLTGFGVSSDIMNGYNKKFRPVLTPGQSILLETHNFDFNTFVV
VSRIMDSTAIQNIIFHDTRFKLITLGTFYNNMAANVVATEQKASKVLGLVFFTFVLCWSPFF
VLNIIFAACPSCPVPTHVVDVCLWLGYVSSTINPIIYTIFNRTFRAAFIRLLMCKCSRWNRLA
RYRSVNEHRTGGGVNTPSTQTGMGSAVPLSLSLQGTPILSPGTASTYLRTPSTFPDSFTVGE
QQT
>RPRC010931 Serotonin
SLLQISQGWILGPELCDMWTSSDVLCCTASILHLVAIAVDRYWAVTNVDYIHTRNRGRIVG
MIVVVWSVALVVSLAPQFGWKDPDYLDRITIQQRCLVSQDVGYQIFATCSTFYVPLLVILV
LYWKIFQTARKRIRRRRTKQHTAQMKIDGKQKSSGPFKFLSKKKLSLICVNWFRLVQEWE
TKRTGEVALVEGNVETDKEGSDAGATTAFTISTNHGHTPSNVSPERSTAELTANSKPPPPLQ
PPSTPVVQTKPKRDAKKESIEAKRERKAAKTLAIITGAFVVCWLPFFIMALLMPLCHTCYI
NENLASLFLWLGYFNSTLNPVIYTIFSPDFRQAFKRILCGVSHSRPRNLR
>RPRC002007 Orphan
MTGSLSDDKMAVSSAKVKVAVFGCSGRYLLNGETSRRFLFLIESRQLPTFSSISTVLVSFEL
ANALILASLRWIRRPLSPTLHISLSLAGADMFTSLVIGIGLIVNSLLPQVFSIQIDKCSQLVIEA
LRMGGMYTSIGHLLTLAVNHYLGIKKPLHYPSLMTTRNITVIVLALWIIPPSSFAIYFSLLEQ

DGFAIIGCDYE
>TINF_H9TUR5Q02IXHQI_1 Orphan
FFVSQVLLVMNEKMGDYEDNTLKLHYYKIQNYSEKNNYSNEHLNFIIYLFHCCFCVFSVL
VNALILASLRWVRRPLSPTLHISLSLAAADMFASLVIGVGLVVNSLLPQVFRIQIDKCSQLVI
EALRMGGMYSSLGHLLTLAVNHYLGIRKPLHYPSIMTTRNITFILMTLWIIPPASFAIYFSLL
EQDGFAIIGCDYKVPNCL
>TDIM_IAZY42G01B8KLV_2 CAPA
AVVSTQSGHYGRGEISSEVVIYVCLSYLFKKIYFNKRKTCILKIFQIEQIISLWRNIIYVGQNI
YHLKILLPITITYSVIFISGLLGNLAVCIVIAYNKTMHNATNYYLFSLAMSDLVLLLLGLPND
LSVFWQPYPWVFGILVCKIRALVSEMSSHVSVLTIVAFSIERYIAICHPLQSYTTDKLNRVIR
VIAALWLISFMFLQYHLQFIQLLTTLISTRFWKEVIDSAFCVHAGLTCPPX
>RPRC000057+RPRC004783 ACP
MDPLFSNFTIEFNYTESIYYSPTNNTLYELPKFDDNALIVVIAYSLLFIIAAIGNLTVFITLVRG
RHRKSRISLMITHLAAADLFVTFIMIPLEIGWRLTTQWVAGNIACKLFLFLRAFGLYLSSNV
LVCVSVDRYFAILHPLRVSDARRRGKMMLTMAWIFSLICALPQMSIACYGAQVCSGVCKM
RLQTDQWRTGHEERTRGRMRLRRSDMSNIERARARTLRMTVTIVLAFIWCWTPYVVMTL
WYMFDRESAEKVDPRLQDALFIMAVSNSCMNPLVYGSYALNFRRECTTCFCYLFSSHQQL
DRRSTDAAAHTSRVLRVPKCVLTFVRGSGITRSTAVTGYGGTLGSRNHLTVPRKNVMRPA
SAEHLVIRGRMIETAPLNPEEFHSDPGTNTGIYLVTS
>RPRC007712 AKH
MTVTEVLRVGLRDLDLGALVNSSFREPAGDFMRKVWSASWSVCNFRHLPHWLQDNDYL
HDGHRPPLPSFKMCFKSIFRIHTETGNIWTHLLGCVAFIAVLLVFMTRPSEELPLSDKIAIGT
FFVGAITCLGLSFVFHTVYCHSEFVGKLFSKLDYCGIAILITGSFVPWLYYGFYCQIRPRIIY
LTVVIVLGIASIVVSLWDKFSESRFRPLRAGVFAVFGLSGVIPAVHYALAEGWLNALTNASL
GWLILMGSLYLLGAFLYAFRVPECLYPGKFDIWFQSHQIFHVLVIAAAFVHLHGITEMLTYR
MSIGACAVQSAALVL
>RPRC004706 AstA
MSGSPASVIVGAIGSMPPIKYDPHNNFTNNTINFNNNIHNFNNNNIDMRNFYSNVTDTEIF
MEEISPELTEKIVAIVVPVLFGIIVILGLFGNALVVIVVAVNQQMRSTTNILIINLAIADLLFIV
FCVPFTATDYIFTFWPFGDTWCKMVQYLIVVTAYASVYTLVLMSLDRFLAVVHPIASMLIR
TEKNAITAILVTWIVIVISNIPVFLCHGEVTFNYSSSEHTVCIFLEMDQLIRPDGFNKVAFQA
ALKWLHKIINADDQAT
>RPRC013486 AstC
DTTVSWLADSLENGSIESLYNSSYGNETQFCGSTDQPTLHIFTQVLYAFVCIVGLLGNTLVI
YVVLRFSKMQTVTNLYIVNLAVADECFLIGIPFLIATMSLQLWPFGNVMCKLYMASTSINQ
FTSSIFLTIMSADRYVAVCHPITAPKMRTPFISKIVSLSAWTASAIFMIPIFMYANIMDDDQVK
SCNILWPEGENLSGQTAFTLYSFVLGFAVPVVLIFCFYFMVIRKLQTVGPKNKSKEKKKSH
RKVTKLVLTVITVYVLCWLPYWITQMALIFTPPKQCQSKFTVTVFLFAGFFSYSNSAMNPI
LYAFLSDNFKKSFVKACTCAAGKEVNATLHLENSVFPRRTQRGGSERARAGKNRADHTD
EGAETGPLVSRGEHSTTALTSRSNITVTSDTTTPVKNGVKINLTPTEL
>TPAL_H9TUR5Q02IYTLQ_5 AT
TPSISSSVCRRTDWQLLSLSCSSSSGHENCHQLFIVNLAVADFLVILICLPPTLVWDTTETWF
LGHVLCKLVLYLQTVSVAVSVLTLTFISLDRWYAICFPLKLKSTTSRAKTAILIIWIISLLYDIP
ELITLRTARRKKFHVETILFTQCIASWDDVAERHYTTSKIVFLYLLPLTITSAAYFQIVKVLW

KSDNIPGHRYQRDVCFISGSSVDSRRYIYYVX
>RPRC007766 CCHa1
MEYKDQLNNDSLVNTTENAITVIPYSERPETYIVPIVFAVIFLVGVLGNGTLVLIFIRHRTMR
NVPNTYILSLALGDLLVIISCVPFTSTIYTVNSWPYGLFICKLSEATKDVSIGVTVFTLTALSA
DRFFAIVDPMRKLYSSIGGRGATRCTIMIACAIWLLAIACAIPGALFSYIRIFKQGNHTLFEIC
YPYPEELGSVYPRGLVMAKFLIYYAIPLTVIGCFYILMARHLVLSTKNMPGELQGQARQVR
ARKKVAKTVLAFVLVFAVCFLPQHVFLLWFYNNPNSDRDYNEFWHVFKIVGYCLSFINSCI
NPIALYCVSGTFRKHFDR
>RPRC000608 CCHa2
MDPETVEDFKYVSSVNSNISNVEEYTPYPERPATYIVPIVFAVMFLVGVLGNGTLVLIFIRHR
TMRNVPNTYILSLALGDLLVIISCVPFTSTLYTIESWPYGGFVCKLCEATKEISIGVSVFTLTV
LSAERYCAIVNPIRRHISTKPLTIVTVFCIWVISFLLALPAAIFTHVSKANITNGRTIEFCSPFP
EEYGPTYRKLNVLLRFIIYYAGPLLIIAWFYILMARHLLLSTKNMPGELQGQSNQIRARKK
VAKVVLVFVIIFIICFLPHHFFMLWFHFNPDSDEEYNLFWHVLRIVGFCLSYLNSCINPIALY
CISKAFRKHFNRYLLCSFVRDSTLDEISLGNMNSSTKHVRQSSIITSHYTITQSEKT
>RPRC001248 CCAP
MDWVIRDNYSNPAANITNTTDEINSFYFYQTEQFTVLWLLFAAIVLGNSAVLLALLFNKSS
KSRMNFFIMHLAFADLSVGLISVLTDIIWRITVEWKAGNVVCKVVRFMQAVVTYSSTYVL
VALSLDRLDAITRPMNFSGSWRRARLLVGFSWTLSAFFSSPILILYEERLIQGSFQCWIELGS
TLKWQIYMSLVAVSLFLVPALVITACYTVIVYTIWTKSIHISRDSQQSTPLKNGGDKGDDND
IRRASSRGIIPRAKIKTVKMTFVIVFVFILCWSPYIVFDLLQVFGYVPRTQTNIAVATFIQSLA
PLNSAANPVIYCLFSTHICRALSKLPPFSWICCCFANRGAESSSIIDTVTSTLRRATIRNQDNL
-
>RPRC000969+RPRC012063 CCAP
LIIFFRKIDELIEVKENHVRGLQSWLGQTEQFAVLWLLFLLIVCGNSAVLAALKCAKKPKSR
MNFFITQLALADLCVGVLSVLTDIIWRSTIAWNAGNIACKVIRFSQMAGKTLVFIVCWSPY
FIFDLLQVYGYVPTTQTNIAVASFVQSLAPLNSAANPLIYCLFSTRICRGIRVSTPQKIGKGIT
E-
>TDIM_isotig12092_6 CCAP
ICTKMANLHESCCSYIVSSTCIGNNCLLYCDCLYYMDEHSYLKRQPAINTIKKWGQRRKYP
KSKFKRNHPKSIKTVKMTFVIVFVFILCWSPYIVFDLLQVFGYVPRTQTNIAVATFIQSLAPL
NSAANPVIYCLFSTHICRALSKLPPFSWICCCFASSGTESSSIIDTVTSTLRRATIRNQDNX
>TINF_IAZY42G02HL8UN_4 CCAP
SGINAEWAVCGNRLGNVPVPAKYSLRTLLKVRSHRVGRRNGLVGILILYNTKNISWHVSIR
LRSCGTVKWAETLNSEYPCTQTLLKTALGRFNGANPISEMLLIFLRNYKLLFCFIQIICNFTA
GQWRTGYEERTRGRMRLRRSDMSNIERARARTLRMTVTIVLAFIWCWTPYVVMTLWDH
FFLFSLFKYPLYLLMGYLNKRK
>RPRC000523 Crz
MQTLFPNISDETTLRQLQDHLINSDDGHRFILPLELCDLWNITVSSSSRIQCLEHAPQLTSSA
RTRAIVLGVMAVISFIGNVLTIISIRSSRRRRRNQNWSAVYALILHLSVSDLLVTIFCIAGEAL
WSYTVAWTADNVTCKLFKFSEMFALYLSTFILVLIGLDRFVAVRYPIKAISTAKRCGRFVAG
AWFLSFLLSLPQVFIFHLSKGPFYEEFYQCVTYGFYTEPWQEQLYTTFSFVCMFMLPLLILII
SYVSTIITISRNDKMFRDESNNTSATRKLDINRRRLIHRAKMKSFRISLVIVVTFIVWWTPY
YTMMIIFMFLNPDKHLSEELQKGIFFFGMSNSLVNPLIYGAFHLWRPSKKTGSARSVSVYF

LLLLIFFL
>TINF_IAZY42G02H0ZZ5_5 Crz
SSIQCLIFLVTIFCIAGEAIWSYTVAWTADNVTCKLFKFSEMFSLYLSTFILVLIGLDRFVAVR
YPIKAISTAKRCGRFVAGAWFLSFILSLPQVSIFFFTLPFIEMNYYRIQCFLYINRRVINSISYV
NTGKTSLFLLVTRIYVHMSSSYSQLYEKFYYPIVELLYRNITIYSRNQTRPRGRNGHSALIPL
X
>RPRC001551 FaLP
MNFTNTNFSLDDNETAYISTDNENDDKSEILFEFITNGVLLNLVGILGIMGNIISMVILSRPQ
MRSSINYLLTGLARSDTVLIITSILIFGLPALFKYTNSQLLFSYYYRVYPFLAPVVYPLAVIAQ
TVSVYLTLTVTLERFVAVCHPLQARSLCTYGRARLYVLLIIIFSILYNLSRFWEVKLEQEYLV
QYNVTVYIPLPSSLRSNQIYISVYIHWLYLLFIYFLPFSCLAVLNAAIYRQVRKANQERQRL
SRLQKKEIGLATMLLCVVVVFFICNILALVSNVLEAFYGILLTKMVKTSNLLVTINSSVNFII
YVIYGEKFKRLFLKLFCSHSPRICDGGVRESPDCATLHEDSVMLSNGDARHSVRGNRANE
SVKRAARALPCVYYPARHNSKWNQDDTTTTTLNQI-
>RPRC015267 Pro
MGNVVTVIVMTRKRMKSSTNTYLTALAVSDLLFLIFNMILSFEHQPAIRQSQYVTYWHLH
KWTIWLVDATGACSNWLTVSFTLERYIAVKHPLRGKVLCTESRARKVIXTCNITSLPLNNY
YSIMECRRDRRVALQSTWLGQHPTYKSVFYWFSSITVTAIPLASLSVLNYLLVAAVRRSTK
GRNQLTEDARGVRGSFRPTCNAGNSIYSRCGSERSPPIQGMSQRRVLKERQENKVTIVLIS
VVFLFLICQAPSAITVIVKVFYEPESDTSGDYLLRSAGNICNFLMVINAASNFFLYCALSDT
YQRTLTTTFCRRERRWNERNDTLSTAASFRNSSVKQLRHENETQ
>TDIM_H9TUR5Q02GDFPZ_1 Pro
SGINAEWPLRRGPAMLASILISLGLDIFDISIRDGNKMVKCPLISSFINNKIYFCLIILMYFISI
NIMFCFTFTLMNKLKNIVVLGACPNWLTVSFTLERYIAVKHPLRGKILCTESRARKSYSVC
YIVGLLLNNYNSVVECRRSRRQVRNIINMKP
>RPRC000494 LKs
MGSRVSWGIFCSYELNKKIMNCSFLEDELGPLPPSANCSWLLHNQSVYFYEERHERAILPS
TSIKEKECIPHQSLYEVPAGVIVLLSVFYGTISVVAVGGNFLVMWIVATSRRMQNVTNCFIA
NLALADIVIGLFAIPFQFQAALLQRWNLPNFMCPFCPFVQVLSVNVSVFTLTAIAVDRHRAV
LNPLSAPPSKLRAKALLGAIWILAAILATPMAVALNVTYVEENDHVGHVYTKPFCINTKLS
NNHMMAYRMILVSVQYLTPLCVISYAYAKMALRLWGSRAPGNAQHSRDANLMRNKKKV
IKMLVIVVALFAICWLPLQTYNVLQDIFPQINGYRYINIIWFCCDWLAMSNSCYNPFIYGIY
NEKFKQEFQQRCPFSRRRKWTHGFGAGGSDSLDLDKTIHRFGSVNRNSSRWIRYSSRVQY
TPAQHYIYHCANSNTVHHSSQSEIEELCLYVLKVMKRLEISITGQNTIANYSTYCRVQDPCL
NY
>GL563029 NPF
MVCRLVGTFRNKIELCVIMELNDTFNFSLNEVYRILIEHKNDDHNVDPVAEAILIALYALLI
VVGILANLIVSFVVARRPQMHTARNLYIVNLTVSDMTLCLVCMPFTLVNILRRAWTLGIVL
CKLVPALQGTNIMVSIGTITVIALDRYFTIVRGQDSATTRRRVIISIALVWFFSFLATLPVVEP
FKFEAVILYETCIERWPSQELKVAYAVCVLMIQAVIPALVVGCIHAKIASYLNAHAKTQRDS
KRAQRELQRNKRTTLLLSGAVAVLFAVSWLPLGLFSLMADLLYPPGSETHISSQSLYITLAA
CHLLAMSSAISNPVVYGWLNSNIRRELVQLLPSRCTSRQQSQQQTTNAPSPTIMLCQNG
QNIPHQQPATTYTAL-
>RPRC008364 NPF

MGKGLKEIHKYKGDIPLHVSTLTLLLIAWDRHRFLKDPMKPRIPAFVCATGSWLTAICLVLP
YPVYTTYMDLGFSNFSPPMLYLYFWRSYGQKPDWGFMRTTGFDNDYENYIKNCEK
>TPAL_H9TUR5Q01BIIZA_2 PBAN
HVVAVVSTQSGHYGRGRYPYVFGEAFCLLRGLAAETSANATVLTITAFTIERYVAICHPFLA
HTMSKLSRAIRFILAIWVIALAFAIPQALQFGVIYGNEPFQILCDVKEILIVHSFEVSTLLFFI
GPMTLITVLYALIGLRLRRSALLTRNSGSFGHGDSGRKGANSCRHHSSQRVLKMLVAVVVA
FFICWAPFHIQRLVAIYIKEMNFISGTTPIX
>RPRC001000 RFa
LILVCIPVKLAKLFSFTWTMGVFLCKMMHYMQSVSAICSVFTLTAMSVERYYAIVHPMKA
KYVCTISQARKIIFTTWVASFFLAVPILFVQVQMPVGGRIKAYWCVRDWDWVVAWRCHE
VYMLVLVLLLPASVMTVTYSAICREIFRVMQRRFHMTSGKATMNCESFPLSKPKKKRPPR
>RPRC002266+RPRC002268+RPRC002269 sNPF
MNNTTIEEDLSMIVDCIVTQYNISQYKGQWPNCTEIGFRKDIIDDKIVQAIFCLLYTSIFVLG
LFGNILVCYVVGRNRAMHTVTNCFITNLALSDILLCTLAVPFTPLYSFLGCRWIFGNALCHL
VVYAQSTSVYISTLTLTSIAVDRFFVIIYPFKPRMRLSTCLAVIFFIWTFSLIATIPFGLFMDHK
SIAGRFYCEEKWPSENFRQVFGGMTSTIQFVLPFIVVTFCYVRVSVKLNDRARSKPGAKTS
RKEEVDRERKKRTNRMLIAMVTIFGVSWLPINLINVINDLYMHTSSWTYYNLFFFLSHAVA
MSSTCYNPFLYAWLNDNFRKEFKQIS-
>TDIM_H9TUR5Q02INV8X_6 sNPF
VLFFFLVCPSFILFLTFYFKVVWIIRCSVPSCGICPKYVRVYLNINIDINSCGSVFLFIIYPFKP
RMRLSTCLAIIFFYLDIFINRNNTVWVVYGSINCWQVLLRTMAIRKITSVWRHDSYITVVV
PFIVVSFCYVRVSVKLNDRARSKPGAKTSRKEEADRERKKRTNRMLIAMVTIFGVSWLPI
NLINVLNDLYMHTSSWTYYYLCFFLTHAVAMSSTCYNPFFIRLAELQKRITSTSMFWRTN
WSPVFRKAWLEVRKNV
>RPRC000835 SIFa
MATIVFGSEMANVSSNSMANSILFELGSEFEKYEETMRKSRFPNRANNPQKIDLVCQIRKP
GCHAQSIVGMRMNTEEEQPFKEYRRAMLRNYAWQAREQEVVAAGVSHVYNKLMMNSL
FPLTSITSDQSGDKKFECFLRAMNGEWGFSLISLNYEHVFLAIWWPLKCQITTRRARLMIL
VIWVVALTTTIPWALFFDLVVIFTDNPEVKVCSEVWPEYLNGSLYFLIANLLFCYILPMILIS
MCYVLIWIKVCKRHIPSDSKDAQMERMQQKSKVKVVKMLVVVVILFVLSWLPLYLIFARI
KLGGEISGWEEDMLPMATPVAQWQSLVNVRTLCP-
>RPRC013738 SIFa
MTEKTSKMMTIRVALPRPKWVKILIIINQHYYREVLAQLLERVRSVDVGNKNQTVPCSEG
NGTTCSTASIYEFRYSFPVTVFFCLAYTSVFLIGVTGNCFVVSVVYRSPRMRSPTNLFIANL
ACADLLVNVICLPFTLISNVMTDCDK
>RPRC004565 SIFa
MSQPRYLLTSLASNDLAIGVLVTPFGFLPALFKCWPYSETLCQIQVVECLKEINLEDLGTNII
ILKKNFVN
>GL545664+GL552047+GL562893 SK
MIHIISFILLQIKMLPTESWWEAGKVQIPTYSIIFLLGLVGNILVILVLVKNKGMRTVTNVFL
LNLAVSDILLGVLCMPFTLVGSLLKDFVFGHFMCRLIPYMQGFFSAVSVSVAVWTLVAISLE
RYFAICRPLKSRRWQTQFHAYKMIAIVWAMSLVWNSPILFVSRLLAMGGKGEGRHKCRE
VWPGRRSEGAYIIFLDIVLLMIPLLIMSLAYSLIVLKLWKGLQRELKHSNSCLKSFSLLLQVI
RMLFVVVAEFFICWAPLHVLNTWYQFRPDLVHQYVGSTGVSLVQLLAYISSCCNPITYCF

MNYRFRQAFISLF-
>RPRC003160 TK
MAYTENSTLGWNELTQNITVNETYDDEDGNQFILPWWRQLIWTFLFGGMVIVATGGNLIV
IWIVLAHKRMRTVTNYFLVNLAIADAMVSSLNVTFNYTYMVNSDWPFGTLYCKISQFVAV
LSICASVFTLMAISVDXXMAIMHPLRPRMGRRMTLCIAVSIWIVGSFFSLPMLIFFTTFVQE
FPNGDNRVICYAEWPDGSTNESRQEYLYNVLFMVMTYFIPIASMCFTYVRVGIELWGSQSI
GECTQRQLENIKSKRRVVKMMMVVVSIFAVCWLPFHIYFIITSHMPEITKLPYIQDLYLTIY
WLAMSNSMYNPIIYCWMNMRFRRGFKQFFSWCPYVHVPPEGLTRREAVTSRYNYSCSGS
PEAHYRIVRNG
>RPRC001687 TK
MRTTTNYFLVNLSISDLLMSLFNCIFNFTYMLDSHWPFGAIYCTINNFVANVSVAASVFTLV
AITLDRYMAIVRPLKHRMSRRKARIALLIIWAASSLLAIPCLLYSTTKSRRTINGQTSTVCY
MMWPDGHYPKSYRYNLIFLVVTYLGPVVAMAICYTLMGRELWGSKSIGEQTQRQLDNIK
SKRKVVRMFIIVVTIFIFCWLPYHGYFIYAYHNNSVVGSWYVQHVFLSFYWLAMSNAMV
NPIIYYWMNNRRFRVYFRQIICLCCCVRPSMHPELQSTPNNRLVRSELLLRSKSCKPQG
>TINF_IAZY42G02H6C37_1 TK
FVAVLSICASVFTLMAISVDRYMAIMHPLRPRMGRRMTLFIAVSIWIVGSFFSLPMLIFFTTF
VQEFPNGDNRVICYAEWPDGSTNESRQEYLYNVLFMVMTYFLPIASMCFTYVRVGIELWG
SQSIGECTQRQLENIKSKRRVVKMMMVVVSIFAVCWLPFHIYFIITSHMPEITKLPYIQDLY
LTIYWLAMSNSMYNPIIYCWMNMRFRRGFKQFFSWCPYVHVPPEGLTRREAVTSRYNYS
CSGSPEAHYRIVRNGVSSRMPRNLNNGPDLLCNLPVNTNNFHQEYLKLNEVHCIVQHVIX
>RPRC015456 Orphan
LTKNVLFVSLLQLSKDIGYVLYSALGSFYIPSCIMVFVYIRIYYAAKARARRGIRKAVARPR
PAEKVTSFSKKEPLTSPVDRNSNNSPVAVTVEKPVIPVVTCDFASDISTSDNIEQPDPTAPKD
TLNVSKLPTCSLTPNVTFKGSTLSVNGDLAAMSRCRAPSVGIDVDMVSEFDPSSSDSGVVS
RCAVVKPLKLRLCKPIFGRKSSKAKREVIDMGRVISTTGSQEIPQEIPKVQKPRDPEREKRR
LARKKEKRATLILGLIMGSFIACWLPFFFLYILTAICSACQIPDFAFAVAFWLGYMNSALNPV
IYTIFNKDFRRAFRRILFK
>RPRC004128 Orphan
QVRNATAVFIINLSVSDLMSCCFNLPLAASTFWRRSWRHGLLLCRLFPLLRYGLLAVSLFT
VLAITINRYVMIGHPTIYPKLYRKQYLGLMVAATWICGFGALIATWLGRWGKFGLDPKIGS
CSILPDSSGRSPKEFLFLVAFVIPCICIVVCYARIFYIVRKTALKSRVAGRSAASVTSGGTTLT
RSGYYGKVKLVRKGNTSSTDDSAFATSSTAQSFSTEKSSTILDNGEMGGNETVIKMVNLAP
SPHLLAPQRRSKICAEASSSSGIEEGLREDDEVVSTRSDSPISACSSSPPPAHYSVKVQKIKK
RSERVNSTLSHMASVFRRTSHARGVLSPSRRQSCAPPQPGKMTAKDKKLLKMILVIFASFV
TCYLPITLSRHTEI
>RPRC008570 Orphan
MMNLSNGSWPDEEEETLYDPPVSLVVFLSLCYGSISIAAVVGNGLVIWVILTSRRMRNVTN
YYIANLALADIVIGLFAIPFEERKLILLDGSYYILKTKKIAGEMTLVKAISAFKCDIWERHNV
LKKKCDSNDFLEKFLPPSKVPILGIVRFYHLHKYLIIKRLRNNPTIRVCLNSTKFQAALLQR
WVLPHFLCPFCPFIKVLSISVSVLTLSAIALDRYRAIIHPLTMKSRKQIDDRTQSMPIQSVVD
VEAISSSTSIISTAPAEDVLTPSLIRSAAFILLRPGTA
>RPRC00203 Orphan
MFNNDWNSSYLLGLDRRQHNGQTYYFTFYSEFGERLAETYIEVGLLVTTLAASVIFNLAL

VLPLWGSKPRTVTNCFLLNLGLADILFAVGIPAVVTVRINPRWPTFAGELMCKLLPYSQLVC
GFTILWSLTLISVERYRCLSLDPNFKISSPQSAHLANIIMWSSAMVLFSPFLFWFRHENDLEI
CTLLFPKPGINVALMFTILITFFTCILPMTILVFNYQRIFIKMVETRQKWATPCVLTSSLSRSH
HFLGALSVALLNTAVNPFLTARLRINSCLRKSMKDKTIFGGSGLFKIGSSIN
>Hheb089310.1 sNPF
MEDPSIMNTEIYDESMFSGNLTVPEDYVTSLIWVQAIFYFLYGLIFVVGIFGNALVCFVVVR
NTQMQTVTNLFITNLALSDILLCVLAVPFTPLYTFLGRWVFGKTLCRLVPYAQGVSIYISTL
TLSSIAVDRFLVIIYPFHPRMKIKVCLSVILGIWVVALFLTLPYGLYMSLEEPQGRVPLCEEH
WPDPTFRQIFSSFTSILQFVIPVLVIGFCYVCVSIRLNDRARHKPGTKTSRREEADRERKRRT
NRMLIAMVGIFTICWLPMNILNIVDDFNVDISNWAYFRLCFFITHALAMSSTCYNPFVYAW
LNDNFRKEFMHVYNFDYIY
>Hheb077620.1
MVRLLLLCFLAGALGYVDGHGCPAPCICKSVGPQNERLRVKCNKDIQDIKEINVNSVSIEL
YHLDLSKNSIYIIEPGIFQNLTNLRRLDLSINKITALEEGCFSGLENIERLDLSKNRIASIDALV
FRQLKNLKKLDLSGNKITTVETNLFHDLLALERLKLNGNLLKTLSEGTFHGLKLLRQVDL
TNNPWDCDCYLYWLSNWKNTSLFKLIPAPTCASPPPLHGHSLLDLRFSDELQCQFTSPIIDL
QPDQNQVVFAGDSMTLHCSVPSITDDRSARLKWYWNPSIFEEAGAFVDPQDTLSNIKVEN
RYLSDSGAIDSSITIFPVTKEHNGQWNCELTSVYGNRSKTISMIVISDETKYCPLVITRNNKG
MYAWPRTVVGWRVELPCEGLGLSGLVPIPLRASYHCNATGSWIDLNTEACPFISPITKALE
QYSKVNLSLTKGNLLETAIRFKNHTSDSTKITDPIEIHFITKTIENYLNFLVEEKELGAMLIDI
VSSIMNLPKDMLKFAETSYNACTRLIKAVELITEFTPSIQLHKNNMALEEFRVKRENFGGLT
CTWYSETSGDKEVKLLHCATNNKTSILSTRDKAIEASIQLPPSLLRRLDLTVAHQLMISMY
TDNSLFPVTSGFSPKIEVTSGVIGAKLIGLQVANLTEPVYVMLKVPEFLSRRPKPVIWDTVG
NSSEWSTTGCQLVNLINDLVIFHCDRLGYYGLLEDTSHLEAVMAPVGEKFRYSNPAIYIGT
FIIISCLTITSVTYIICHASIVMPKRAKHCVVNTWVSITLLCFLYTAGIQQTDNLEICQSVGLV
QHYLSLCSLLWMAVTASTMYKRLAKPDITQVPDDEIPEQPIQKPLLGLYLVGWGIALIVCG
ISGAINLREYASYSFCFLSSGAALAALFVPAVILIFYLTIFYLLVRCAIRSGDHNGQLSEGTQA
TENMDLELLEPNDNRADQNSVHSTQTVSSEVEDVEHSQITQLKGHIVVLVLYLIMWCAAA
AATSRPFNPHLPHEETIFAVLYAISASSLGFFVLLFYGIARSDVRSQWTIMRCWLRRKKNRC
CRTRSVTDANPSLPAQPLVQNLTMPVQGTPQIVSDTNSLSSSRITSASRACNALKISDGGSD
TPSITKKGPNMNLVVLHRQQYRSNNSVTTFTEATHSVEMFYNPHQSGVARKFFKKQRRHT
KHNNLGPRKQGDGGATSDGGSCVSIPRPAKIQDSEIERSIFGSSAKVNNTNIHVELNPVTAT
KNPNILSDSGGSISEDRTMPLRYVIGQEHTRISRKINNDDIRMIHNHPPERIRINPNAIECIRID
PNLASESVRMSQNVADCIRMSPNESDLETRTEEEKHLRNVSQQCSLEYSSEMDSGGQMLS
ERSDHDLPEIDETPETPDKITEGDFKCSSLHELTQMVDDKALARSEHSSSLYCLSEDRFEEP
PLRTSYRSSYNDVTSLGTSSRNCEDFESEGKPTTDDNSRCSSFSNVNRMIPSDEELELLNEL
TLPNLNERAIEPSDYEKEYNSMTDLTAIDITLGPTRHLDMNASIGVDYEDANYENSQHFSE
DGVPLDDAIIDANHGKKETSV
>Hheb081860.1 Orphan
MKAQLPLGLSEDIGYVLYSALGSFYIPSCIMVFVYIRIYFAAKKRANRNIRKAPRPRPAIAIP
PESPDIRQTSFTQLTPATDSRLSSGINTMDNVATIESPQIQIPIVTCDYASDVSTSEADPGANY
NNMEEKDTLKSLVNLQVNAPMPMQKLNLKATLSVNGNEGQSTSPKLPARCRAPSVGIDV
DMVSEFDPSSSDSGVVSKCAVVKPLKLRICRPIFGKKAINKVKKEHSSDGKHINRDGVVV
DNSTPRPRDPEREKKRIARKKEKRATLILGLIMGSFIACWLPFFFLYILKPLYANLNTPGAAF

AVAFWLGYMNSAFNPVIYTVFNKDFRRAFRRLLYK
>Hheb006770.1 AKH
MNATRELPIDMRFNDGHVVSIIMYPILMVISIIGNVTVLYLILQRRRSNRSRINTMLLHLAVA
DLLVTVLVMPLEIGWAITVDWRAGDAMCRIMSFFRLFGIFLSGFILICISIDRYYAVLKPLQL
MDVDRRGKIMLVSAWVGAFICSAPQVVVFQQKSHPEFTWYNQCISLGSFPSYAHELTYFIF
GMTMMYWLPLSVIIFTYSSILLEIYRKSKEAGDKIRRSSVGFLGRAKIRTLKMTITIVVVFF
VCWTPYNIMSVWYFLDRESAREVDQRVQKFLFLFACTNSCMNPIVYGVFNIRRDTRGSTS
QQAVTIGKKTSIENVVCRVSWRRQETGSIRDNNDQNGQDTDVCLDIANQLSYNIGKNHEN
LTKSFGEAQYDDLFAIAVKHPKEKKLNHHSGKQTLIATKYGSSDTKFLFMFDSTSRRDCQF
GGEIPLNTTFREAMENGLTESAFQVFSDKKYHPRVYKNSRIQEGWKHANCMSNSLLDVD
YLRDLNVTNMRVRRRGDQPSIHKLDELNCIGEQKLVSTLNTLIEYTNQILEELRRNTQETR
YVRKLLEECQGCKAPLPPLPPLRPSCDYNAPQCYPGGQCRDTASGPICVCPPGYRGNGIQC
ERVSACATAKCYPGGQCRETERGPVCSCPPGHIGNGITCERIRTCDDRPCFPGVRCENTPR
GYRCGPCPPGHEGNGETCQTIRITCEMSPCGRGVTCHPIHEPPHHRCGGCEPGWKQHGSE
CRDIDECDLENPCRPNEECKNTLGSYRCIPCAPGYRGSRTGCVDIDECSTNNGGCVPNSEC
INTPGSYRCGQCIQGFTGNQTSGCYQVGNLCPDRVTVCHERATCNCIVPNVEYTCQCQVG
WAGDGFACGIDSDNDRHPDEELNTDGDIIPADNCPHIPNSGQEDVDGDGVGDACDDDAD
NDGVLNSSDNCPYDANVAQEDTDRDRKDGVGDVCDNCPSIYNPHQEDTDDDGIGDACD
RDIDNDGIINENDNCRFVKNEDQYDSDGDGVGDVCDNCRSVPNSNQSDSDRDGVGDAC
DTGRDRDRDGIQDDVDNCPDVPNADQLDTDNDNIGNACDDDIDGDGVPNLIDNCPYVY
NPRQEKSHPGISGDACWNDFDNDTIANPYDNCPNNSQIWSTDFRQYERIPLDPVGDAQLD
PKWYTHDDGAEIEQTVNSDPGIAIGFDHFGGVNYEGTMFVNTDIDDDYIGFVFAYQNTHK
FYAVMWKKNSQTYWRSEPFRAVALPGIQIKLVDSETGPGKEMRNSLWHTEDTPKQVKLL
WRDPKNVGWKEKTSYRWELLHRPKIGLIRLWIHQGDKVVVDSGNIFNSALKGGRLGVLC
FSQEMIRWSNLQYSCRETVPQIVYDELPADLQAKVGVDSPKY
>Hheb008350.1 ETH
MKMLTTMSSTAFELNSSYYTTAIGSSSTIDGFSVSVLPPATNATSIPYVLPAYIRITSMVVVII
VMVLGIVGNLMVPLVVLRGKDMRNSTNIFLVNLSAADLCVLLVCAPTVLVEVNSGPQVW
PLGEHMCK
>Hheb008990.1 Orphan
MHSRMGNAILLSHRPSRDLNNLNGELNSGGSSKTLTLNEVNQDHHLHTPTKDKNLMKM
KREHKAARTLGIIMGTFILCWLPFFLWYVSTSLCGAHCHCPEIVVHIVFWIGYTNSALNPLI
YAYFNRDFREAFKNTLQCAFCSLCRREPFDLEALDIRRPSLR
>Hheb055420.1 Orphan
MEEPSTEALMQAGFIFVVSIAIILSNLLIIATYLNFRGPSEVINCYLLSLATADLLCGLLVVPL
SVYPALMKRWVYGDIVCRLVGYLEVTLWAVSVYTFMWMSVDRYLAVRKPLRYETVQTK
TRCQCWMAFTWISVAMMCCPPLLGFNKPIFDEQAFICMLDWGNMAAYTITLSILILGPSVI
TIVYTYFYIFSMKLKLRSGVPIHDKEYATALSENLSNPSHYMSFALIMTFWLSWAPYALLRI
YISIQGAAEIPLLHFAVVWLGITNSFWKAFILGTMSPQFRLAARVLCLTLCCRHRRLPPELL
GLDDDD
>Hheb074400.1
MSSDYLNVTNKIYPGTTKAELNLPYAVCEIVVAVCAVLGNGLVIIVFSREKKLRRRTNYYII
SLASADLMVGLFAIPFAILASIGLPTNFYACLFTVSVLVVLCTISIFCLVAVSIDRYWAILYPM
GYSRNVRTKTAIAIICVCWITGTLVGFLPLLGWNAGFKKTDEKCIFVEVMDYNYLVFLYFA

TIIFPAFLIAAFYAHIYRVVIQQQLMSRKPINIEGKRRKRKGNNNNNEPSSGTMLRLLGAAQ
KREVKATQNLSRIVIFFIICWFPLYTINCVQAFCPDCTVSEFVLNATIILSHLNSVGNPILYAY
HLKDFRAALKNFILRILNPGRCRRDNNAMGMNEIQNKIISQRNLAKKSLNTAIKHTNSNLS
EKGVTLALPEPNNIDSSPSSTPTINRSPTICIKYEFPEEDVGYVDESLSSNVKTSDNSENYNN
SNNYDLIEDSRDFKDDSVLQLREHQITIEESQLKEIE
>RPRC001428 GPA2/GPB5
MTKSSSQFARNLASTNSATIVGNMAAGTSNLVTTRTRTPSSQTKVTEMLLVVSTVFIILNLP
SYVVRVWIYLTDTHNVGTEQKVTMYVLQQYCNILFNTNFGINFALYCISGQNFRRALLSLF
RPEIQRRSGETTQTTED
>RPRC001049+RPRC001048 Opsines
MELMLMPSAGFLAASIILFLIGFLGFFGNLIVIIIMCRDKNLWTPVNFILFNVIVSDFSVAAL
GNPFTLASAIAKRWFFGQSMCVAYGFFMALLGITSINSLTVLALERYLIVSQPVSHGSLSRP
TALTIVGSIWLYSFVITAPPLVGWGEYGLEAANIRSANSTMNAGRVNKAESRVTWMIFVMI
FAFFLAWTPYAILALMIAFFDSNVSPAIATIPAIFAKTSICYNPFIYAGLNTQFRQSWRRVLGG
KREDSTTMATATSFGLNSKRYKEVSCVIDVKGKDKIKLSALNKSTATETAI
>DmCG11144
MKQKNNNGTILVVVMVLSWSRVVDLKSPSNTHTQDSVSVSLPGDIILGGLFPVHEKGEGA
PCGPKVYNRGVQRLEAMLYAIDRVNNDPNILPGITIGVHILDTCSRDTYALNQSLQFVRAS
LNNLDTSGYECADGSSPQLRKNASSGPVFGVIGGSYSSVSLQVANLLRLFHIPQVSPASTA
KTLSDKTRFDLFARTVPPDTFQSVALVDILKNFNWSYVSTIHSEGSYGEYGIEALHKEATER
NVCIAVAEKVPSAADDKVFDSIISKLQKKPNARGVVLFTRAEDARRILQAAKRANLSQPFH
WIASDGWGKQQKLLEGLEDIAEGAITVELQSEIIADFDRYMMQLTPETNQRNPWFAEYWE
DTFNCVLTSLSVKPDTSNSANSTDNKIGVKAKTECDDSYRLSEKVGYEQESKTQFVVDAV
YAFAYALHNLHNDRCNTQSDQTTETRKHLQSESVWYRKISTDTKSQACPDMANYDGKEF
YNNYLLNVSFIDLAGSEVKFDRQGDGLARYDILNYQRQENSSGYQYKVIGKWFNGLQLN
SETVVWNKETEQPTSACSLPCEVGMIKKQQGDTCCWICDSCESFEYVYDEFTCKDCGPGL
WPYADKLSCYALDIQYMKWNSLFALIPMAIAIFGIALTSIVIVLFAKNHDTPLVRASGRELS
YTLLFGILVCYCNTFALIAKPTIGSCVLQRFGIGVGFSIIYSALLTKTNRISRIFHSASKSAQR
LKYISPQSQVVITTSLIAIQVLITMIWMVVEPPGTRFYYPDRREVILKCKIQDMSFLFSQLYN
MILITICTIYAIKTRKIPENFNESKFIGFTMYTTCIIWLAFVPIYFGTGNSYEVQTTTLCISISLS
ASVALVCLYSPKVYILVFHPDKNVRKLTMNSTVYRRSAAAVAQGAPTSSGYSRTHAPGTS
ALTGGAVGTNASSSTLPTQNSPHLDEASAQTNVAHKTNGEFLPEVGERVEPICHIVNK
>Hheb080560.1 Serotonin
MMRDLNASACNELYEAVEWSGPGIVGTLVVLAIVDVMVILGNVLVILAVYHTSKLRNVT
NMFIVSLAVADLLVGVAVLPFSATWEVFKVWIFGDIWCSVWLAVDVWMCTASILNLCAIS
LDRYLAVTRPVNYPQIMSPKRARLLVAAVWVLSFVICFPPLVGWKDQMSHPTKTDPVPEK
NGPFNTTIILVPVKPCPWICELTNDAGYVVVYSALGSFYIPMLVMMFFYWRIYNAAVSTTKA
INQGFRTTKGSKMFGSRFDEQRLTLRIHRGRGSVHNGANNNSTSSPRSPESSRSASVRRDKI
KISVSYPSTETLNTKCNTLERTPSKCSQISVHYTNGQTQNQLCTTSRNTHLKVGGINRVGS
ARRPSRRSSCESQVTGDEVSLRELATSSEDKPPRVMKMGKRNIKAQVKRFRMETKAAKTL
GIIVGGFILCWLPFFTMYLVRAFCPNCIHSTVFSVLFWLGYCNSAINPCIYALFKATEAQVIQ
YVCHSRAYLSRTLIQIQALSIMSTHRVTLDDRRVKYGERLV
>Hheb033210.1 RYa
AVSVLVSAYTLVAISIDRYIAIMWPLKPRMSKKQAKLLILAVWLVALTVSSPIAFVSQLLQPN

ERYKKCNQFICQEYWPSAHQRYYYSIALLVLQYLVPIVVLMFTYTSIAIRVWGKRPPGEAE
NTRDLRMAKSKRKMIKMMMTVVIAFTVCWLPFNVLTLILDNNESINSWRGLPFAWTALH
WLSMSHSCYNPVIYCWMNARFRSGFISALAGIPCFRKFWPERRTPPYNTSTAGGIALTGNV
IYSVPKEQLKNPQQPLKKNQTKIQHCVDTKTL
>Hheb00794 CNMaR-2
MYTIQSYCAGSRLRRSLLQQEASLSSNDSYENGYDYEEEPLPCTMTDFTFLIALLFVWLNS
ELGWKVFNVAGWCEILVYVSAVCSSLSVWLIVAFTVERFIAVQYPLHRPQMCTISRAKTIIC
ALVVLSLVCHSYAFITAGVVTVGDSDYCDLKVEYMDVMKIISTVDSIASLIVPIVLIVVMNT
MIMRNLLKFSRRFKQTPMNSLITSNQCPSRERSDINLNQIPNKSSSQSGIMLATIVGKRGGS
QQSFHSSRNSHSHHSSSGSGGPVIITTTQTANVTSPAPPSSSGARNISYQVPEVGGAKCISLY
HHTKSIGTSSKSVVSTRNQQSITKMLLLISTVFILLNLPSYVIRLCIFFFTLAKRDSPDLLWCL
QQFFMLLYYTNFSINFLLYAMCGMTFRRCLEQLVQKAFKGVTR
>Hheb09984 NTL
MTSCCLENESNITMPAYMAIMRPLRYHLSRRRTIGALVLIWLASVLLAIPGLLYSTTMTRRY
SNGKTRIVCYIMFPDGDYLNSRIEYIYNLIFLGVTYLIPMTVMAVCYSLMGRELWGSKSIG
EHTKHQKESMKSKRKVVKMFIIVVMIFAICWLPYQGFSIYLYHHSDISSSSYIQHVYLSFY
WLAMANAMVNPLIYYWMNNRFRVYFQKIICSCCVIGRSNAGSCQMHELADFHRSDTARS
NSGRLKFTTIRWRQSTAESHVHPYKIKSRSICEKIHTSRQDVAVI
>Hheb08603 CNMaR-1
MEERVITSNKTNQSDTEDWIRMGSTTDWVETTMIAIQMYYTPVLVCLGTLGNCLSVYVFF
RTKMRRASSSWYLSALVVSDTGFLISLFFAWLDMVGIGIFNLSGYCQFFVYLTTLCSFLSV
WFVVSFTVERFIAVQYPLRRQSMCTVARAKMILIGLTCVGLVLCSPVLWFSSPRPIDKKPN
VTGCRLVEEWEAWASAFNVADTILTFVLPFTVIVILNGLIARTVYRLARVRRTLTTNGRNRR
DQKCNGIGVSQTKVTKMLLIVSTVFLCFNLPAYVMRVRAFLEVHDLRSTIIAQQICNIFFET
NFGINFVLYCASGQNFRKAVVRLIFRRPRRWHSGTPVSNHVSDFRRSSSTMGRQRTIVYEV
PWTEAYEMRGLDSRVSRQLSQQSNQGKL
>Hheb03416 SIFa
MVGGLYTAKTLNSFVKSYYESILKITGAQRVHTWVLGRFMCKTVSYIQGVSVAASVYSLV
AVSLDRLYLLPFWLSLLPPQVVSPVYPLTTDLATFTHPMLLLTHQSIMLPMLPMSMLSTSPL
LQSYTLHQLFLAIWWPLKCQITKRRARLLIVIIWCVALGITVPWLLFFDIKIIPSSVDQAFCV
ESWPRKEGERLFFLIGNLMFCYVLPMILISLCYIAIWVKVSRRNIPTDTKDAQMERIQQKS
KVKVVKMLVVVVILFVLSWLPLYVIFARIKLGSSPTIWEEDVLNIATPIAQWLGSSNSCINPI
LYAFFNKKYRRGFMAILKSGRCCGKLRYYETVAIMSSSTSMRKSSYYVNNNNSSTRRTCH
GPPVHQDSNVSYIFNHTGV
>Hheb05423 TKR
MLLTYFLPIGSMTFTYARVGLELWGSQSIGEATQRQLDNIRSKRRVVKMMIVVVVIFAVCW
LPFHVYFIVTSYRPEITNQPYIQDVFLAIYWLAMSNSMYNPIIYCWMNSRFRRGFAQFFSW
CPFVKVTPEPGLSRSEAVTSRYSCTGSPDGHTRIARNGTSTCSSSDQSLSTIASGTTRRDTIL
HERVDIDLCEFTSP
>NL A42 ELeR
MKSALIADSAHDNSSEFHLKNMSYDGDYLEEYDYDLNDTCRVPWEELTPAVAVYSLTFVL
GIVGNSLIVFTIFRYRRMKSTTNVFLASLASADLLLIIICIPLKIAKLFSYTWTMGVFLCKMV
HYMQNVSAICSVLTLTAMSIERYYAIVHPMKAKYICTISQARKIIIGTWLASLLLGAPIMFIQ
DHIEVGAKIKGYWCVRDYVNNREWVTAYELYMLILILIIPTSIMGITYSSICWEIWRVMKQ

RKTMTSGKATLTTETFPLSSKRSTCSIKTTAKSCIRTTDEENGTVRQVIKMLVMIVIVFVIC
WGPLLVDNVLTAFGVLPDQKSGTLKHMYTGFTLMAYFNSCVNPIVYGFMSKNFRESFQK
ALCRCCRGPPKRTLSMSQTRTTSIRVSRLRRKQSKFQDMELTVM
>NV10767
MIKIQRSVYGVKHEQQPDGRRGAAGCRTLAALAAPSLLLLLLIFLSVAGNVLVIVAICTDR
ALRRIGNLFLASLAVADLFVGCLVMTFAGVNDLMGYWLFGSGFCDTWVAFDVMCSTASI
LNLCAISLDRYIHIKDPLRYGRWVTKKIAWASIMTVWLLAILISFLPISLGLHRPNEPPVVHI
VGEVPMQSPHVSMYLQSYPTCALDLTPTYAIVSSCISFYVPCIVMLGIYCRLYCYAQKHVR
SIRAVTKLPEPSSLAKSFRSKSKRTKTPKPPTKAKPTSPYHVSDHKAAITVGVIMGVFLICW
VPFFCVNIVAAFCKTCIPDLAFQILTWLGYSNSAFNPIIYSIFNTEFREAFKRILTKGARARYN
QPSTSECGEFRSVVVARRNGSVVECNISPRSSADSCQVGTIGQRHRDTIVSAI
>DmDop2R
MLSPFDWRRGISSSGTGGTMAAQPLSSTAATTAAATGATAATAATAATTSATLSTAAASTST
TAAPSAGATWINHHLAVEADSSQPANGSDAQAGVEGPTMPAGYLPLYEDVETAAEDAGY
ALIDDISEWLLGSVGSEAAVGGPENSTNLAVTGANGTLAWLEALNSTQPAQSNSSAEDGE
RGRYSLRSFVEQQLAGGGAAGAGDGGDAGIALIDSGEEAALDNVADAETDYGMLGGFG
DAELLQRTATVARETLGNRTAPSTTSYDGGGSGDVGVAGGLAGTAGGGVGGAGGSGGST
FMLLLENFNDYFPNYNGSTVSGTSTIAPGVAITGSRGSGLLLEQNLTGLYLDGYRLNCTNE
TLNLTDSCGELRVVDHNYWALILILFPILTLFGNILVILSVCRERSLQTVTNYFIVSLAIADLL
VAVVVMPFAVYFLVNGAWALPDVVCDFYIAMDVICSTSSIFNLVAISIDRYIAVTQPIKYAKH
KNSRRVCLTILLVWAISAAIGSPIVLGLNNTPNREPDVCAFYNADFILYSSLSSFYIPCIIMVF
LYWNIFKALRSRARKQRAARKPHLSELTGGSVIENIAQTRRLAETALDSSRHASRILPDEA
ATNTASGSNEEEDENAISPDIDDCHVIVNDKSTEFMLATVVEETGNVVAQITTQPQLVVAD
PNGNHDSGYAASNVDDVLAGVAPASASAATSAAPRSSGSPPDSPLPSGATLQRSSVSSQRR
PTGDDSPKRGEPALRSVGVDNSSVAMKPLSFVRYGVQEAMTLARNDSTLSTTSKTSSRKD
KKNSQASRFTIYKVHKASKKKREKSSAKKERKATKTLAIVLGVFLFCWLPFFSCNIMDAM
CAKFKKDCRPGLTAYMMTTWLGYINSFVNPVIYTIFNPEFRKAFKKIMHMG
> BmTAR
MGQAATHDANNYTSINYTEIYDVIEDEKDVCAVADEPKYPSSFGISLAVPEWEAICTAIILT
MIIISTVVGNILVILSVFTYKPLRIVQNFFIVSLAVADLTVAILVLPLNVAYSILGQWVFGIYVC
KMWLTCDIMCCTSSILNLCAIALDRYWAITDPINYAQKRTLERVLFMIGIVWILSLVISSPPL
LGWNDWPEVFEPDTPCRLTSQPGFVIFSSSGSFYIPLVIMTVVYFEIYLATKKRLRDRAKAT
KISTISSGRNKYETKESDPNDQDSVSSDANPNEHQGSTRLVAENEKKHRTRKLTPKKKPKR
RYWSKDDKSHNKLIIPILSNENSVTDIGENLENRNTSSESNSKETHEDNMIEITEAAPVKIQ
KRPKQNQTNAVYQFIEEKQRISLTRERRAARTLGIIMGVFVVCWLPFFVIYLVIPFCVSCCL
SNKFINFITWLGYVNSALNPLIYTIFNMDFRRAFKKLLFIKC
>DmOct-TyrR
MPSADQILFVNVTTTVAAAALTAAAAVSTTKSGSGNAARGYTDSDDDAGMGTEAVANIS
GSLVEGLTTVTAALSTAQADKDSAGECEGAVEELHASILGLQLAVPEWEALLTALVLSVIIV
LTIIGNILVILSVFTYKPLRIVQNFFIVSLAVADLTVALLVLPFNVAYSILGRWEFGIHLCKLWL
TCDVLCCTSSILNLCAIALDRYWAITDPINYAQKRTVGRVLLLISGVWLLSLLISSPPLIGWN
DWPDEFTSATPCELTSQRGYVIYSSLGSFFIPLAIMTIVYIEIFVATRRRLRERARANKLNTIA
LKSTELEPMANSSPVAASNSGSKSRLLASWLCCGRDRAQFATPMIQNDQESISSETHQPQD
SSKAGPHGNSDPQQQHVVVLVKKSRRAKTKDSIKHGKTRGGRKSQSSTCEPHGEQQLLP

AGGDGGSCQPGGGHSGGGKSDAEISTESGSDPKGCIQVCVTQADEQTSLKLTPPQSSTGV
AAVSVTPLQKKTSGVNQFIEEKQKISLSKERRAARTLGIIMGVFVICWLPFFLMYVILPFCQ
TCCPTNKFKNFITWLGYINSGLNPVIYTIFNLDYRRAFKRLLGLN

>DmDop1R2

MVDDNGSSPEVEGAEGAGAPLLALLRVDGLNQTQTRSPSPSFFGSYNISEDVYFYFNGLPT
STELVLNATTSATSATLSPAMVATGGGGTTTPEPDLSEFLEALPNDRVGLLAFLFLFSFATVF
GNSLVILAVIRERYLHTATNYFITSLAVADCLVGLVVMPFSALYEVLENTWFFGTDWCDIW
RSLDVLFSTASILNLCVISLDRYWAITDPFSYPMRMTVKRAAGLIAAVWICSSAISFPAIVW
WRAARDGEMPAYKCTFTEHLGYLVFSSTISFYLPLLVMVFTYCRIYRAAVIQTRSLKIGTK
QVLMASGELQLTLRIHRGGTTRDQQNQVSGGGGGGGGGGGGGGSLSHSHSHSHHHHHN
HGGGTTTSTPEEPDDEPLSALHNNGLARHRHMGKNFSLSRKLAKFAKEKKAAKTLGIVM
GVFIICWLPFFVVNLLSGFCIECIEHEEIVSAIVTWLGWINSCMNPVIYACWSRDFRRAFVR
LLCMCCPRKIRRKYQPTMRSKSQRFATRRCYSTCSLHGIQHVRHNSCEQTYI

> Bm Dopr2

MNATEPDIVVARGQLDSSYRSPEYTLEFTGAAYNNAIEFNLTFDNISAYNISSEPEGLWNDY
IKLLHDRALLVSFLLLFSLTTVFGNMLVILAVVRERYLHTSTNYFVTSLAVADCLVGLVVMP
FSALYEVLEHTWFFGVDWCDVWRSLDVLFSTASILNLCVISLDRYWAITDPITYPMRMSG
RKAAFLIAAVWVCSGAISFPAIAWWRAVRTEEVPDYKCPFTENLEYIIFSSTISFYLPLFVMV
FTYYRIYRAATIQTRSLKIGTKQVMRPSGELELTLRIHRGGTMRQRNDVCHGVCTPEEAD
QEPLTALQNNGLSRSSTRLTNVTHGKHLPKNFSLSRKLAKFAKEKKAAKTLGIVMGVFIVC
WLPFFVVNLLSGICSACIAHEEIVNVVVTWLGWVNSSMNPVIYACWSRDFRRAFLRILCV
CCPRKLRRKYQPQLRFKQSQYPVATMMYSSVSQHEMCRL

The family B

>KAF7987641.1 hypothetical protein HCN44_003504 [*Aphidius gifuensis*]

MNIEVNTMAPNGSGYNSPYIQDAIIKWHEKNCRIGFVSSSDECPEIWDGILCWPPTTPNKL
AELSCPGYFAGFDTQKNATKQCMPDGQWYWSPENNQTWTNYTQCSTTKLVTVLMDVPK
HNSSLTEMYLPIIRNISTIGYTVSLSTLVVAFCILATIKKLRCSRNLLHLHLFVSFMTRALMA
LLKNIIVASGLSVASDIIEINGASYLHVNDYESNWLCKTFASLWQYFILANYSWILMEGLYL
HNLVFFALFPENNSSIAGYVILGWGLPVIFVIPWAILRTLFDNTYCWFTNESSYIFFYMIRLP
TGLSIIINFILFINIVRVLLLKLQSTISEETQRYKRWARSTLVLVPLFGVHYALFLGMSYSIGV
NEKVELIWLFVDQLFASFQGFFVAVLYCFLNGEVRVEVTRAFNSNRWSRLRRSQRTSTRSD
SACSCQLKKIRKHKKRGFINFILACFCTDNSMHHRSSHSMMSTQQDIATRGCSLASSRVFF
DSPGPGMYSTDNQDCTSLDSSACNRYTARSTLSFCSHMLADTAGPHLNIMTTDDRPNNT
WSDTESCLALKLDDYPQLEIS

>KAF7993218.1 hypothetical protein HCN44_006278 [*Aphidius gifuensis*]

MVDLQISKNNSETLCKLQYQYFTPPKGEIWCSAAWDSILCWPPISSSTTSKQRCPLDNGVD
TSKFAERRCSVNGSWEGRGKFGEQDSPNGWTNYTPCFTPEMLMLIRKLYAGTKDAAKLR
LEIAEATRTLEFVGLSISLAALVISLTIFCRFRSLRNTRTRIHKNLFVAMVIQVLIRLTLYIDQA
LLKSSVYGPKQGINNTPILCEASYVLLEYARTAMFMWMFVEGFFLHNMVTVSVFHQASY
YRIYRLIGWGLPIVMTSGWALVTALNYHPSKCWWGYNLSVYFWILEGPRMAIILLNFLFL
LNIVRVLVVKLRQSHSSEIEQAGKAVRAAVVLLPLLGITNLVSMTSAPLDKTFWFGMWSY
TTHFMTSFQGLFISTLYCFLNGEVRLAVKKSISVYLSLRTAEPNNQRRSTLGASQPPQLISG
KLK

>KAF7989120.1 hypothetical protein HCN44_007430 [*Aphidius gifuensis*]

MRKDMINMSDTFDVNHVQDILDHINQLDDTNLSKDLQCLKSKHDKIYNNACDIDWDSLS
CWPQTLAGTLAKIPCFDQLYGIKYDSSQNASRWCLTNGTWDSYSNYSQCHELQIPVNESS
VEITTMLYFIGYSLSLSTLVVAVSIFLYFKELRCLRNNIHTNLMLTYILADLMWILTTVMQVS
MQSDTPTCIIFYSLLHYFHLTNFFWMFVEGLYLYLLVVKTFTGDNIKLRLCLAIGWGAPVL
VIIVWGIAKSSAVDISNDHENQNGALYRHCPWMVYHAYDWFYQGPAIIVLGINVLFLFMI
MWVLITKLRSANNVETQQYRKASKALLVLIPLLGVTYILVIAGPSKGKLANLFSYARAVLL
SSQGLSVALFYCFLNSEVQNTMRHHFSRWSTARNLGMDRKYYNNWSPRSRTESIRLYCQP
SNPYRKRESTVSETTTTTVIGINSTTAFIDRSKTVISEDLNE

>KAF7990226.1 hypothetical protein HCN44_000031 [*Aphidius gifuensis*]

MMPNNLSTTVGSTLQDDEFRRVVEERRQECLEQLAINTTVPAGLYCRSTFDGWSCWPNTL
AGEKAYAPCPSFITGFDESRFAHKFCEADGTWFKHPLSNIVWSNYTTCIDMVDLGWQQKL
NVVFEIGYLISLIALLLSLAILTYFRSLRCARITIHMNLFASFAINNALWLLWYIFLANPGLL
MENGIFCRSLHVILQYFLLTNYAWMLCEGFYLHTILVNAFTSEHKLVKWLMALGWPTPAII
VTIYTILRATSDDPKDLSQCWIDESNYINVLIYPVCVSTLLNLAFLCNIVRVLLTKLRAGPAI
GSRPSRSMLQALRATLLLVPLLGLHYLVIPFRPPKGHPYAETYDVISAITISFQGFCVAMLFC
FCNGEVIAQFKRKWEGSAFIRKRQNSCTATTVSVRWRERRRCDYQLELPTIQKDGILVENH
NSPIVISTRNDSNRIKNSRGLIKRNDSAYDVNQYQTQC

Other insects:

>Hheb107890.1

MAWSDIDFNSPIIQQQMLEMGALRCRNGSAPEPGWCPETWDSILCWPPTPAGALALLPCP
RYIAGFDLQANATRQCMPNGQWYENPETNYTWTDYSQCYNSELVTVLMDLPEVEERNK
TLIKTYLPIVSKMSKIGYAVSFSTLLVALVILATLKRLKCSRNKLHMHLFVSFMLRAFMAM

LKDILFVSDLGLASDVITKNGESFWVVDDVVHNNWQCKMFTSFWQYFILANYSWIFMEG
LYLHNLVFFAMSADSNASIKGHVVSGWGLPVIFVIPWVVARALYDDTYCWTTNNYQSLFL
IIRVPTITTIVMNFLLFINIVRILYVKLRNSVAEELERYKRLARSTLVLMPLFGVHYAIFLTISF
SGLNEKVELVWLFGDQLFASFQGSFVAVLYCFLNGEVKTEVLRELRSKKWLGSRWGLGS
GPRHSTRSNSTCSCNGKSGKLRKPRWKRPWFGLFHRRTIHRSSHSMASTQDVTTRGGSLT
SSHGYLDSVVVEGNKTYNTDLHDRSVTQMSPLCSKFTDQSMLSFCSNYLPANFRLTR
>Hheb003130.1
MTNGDLERFLQDQQDLCDLHIAEQAGLNNGLCPPSFDGLLCWPATLANTTAQLACPPRVV
LGYENPNNGTATKRCLASAVWYNNSEGIAWSNYTACTPPQGYYITAIENIEITHAEYAKAN
NSTLLMKWLPIIRNVSKVGYATSMISLIIALIIFSLLRKLKNHRNRLHMHLFVSFVMRAFMA
LLKDWLFVDGIGLAWDIVLVDGKSAFIKEKNIWVCKAITSAWQYFIVANYTWILMEGLYL
HNLVFLAFCADTSAITLYVVFGWGLPAVIVGLWIIIRILMEDTLCWTTHTNPSLFLIIRIPIMV
SLLFNFMLFLNIVRVLLVKMKTSDHLQRKKMRYGRWARSTMVLMPLFGAHYTIFMGLSY
HEDHQIELIWLFCDQLFASFQGCFVALLYCLMNSEVLSEMRRAWRAQCARRGGVWLDLT
QRNFGKNSERGHGNKGTNNVTSNVSML
>Hheb011390.1
MEVEGKDDEVWCRWAWDSILCWPPTKASTTARLSCPLENGIDTTKFAERRCSDDGRWEG
KGGVINEQTDAPSGWTNYTPCFTPEMLLLLQKLYSGNEEDAKVKLNIAQRTRTLEFVGFS
LSLAALLISLAIFCRFRTLRNTRTRIHKNLFVAMVIQVVIRLTLYIDQALDKTEVYNAQQGIN
NTPVLCEASYVLLEYARTAMFMWMFIEGFFLHNMVTVTVFHETSYYRMYRLVGWGFPAI
MTITWATITAIYYHPSKCWWGYNLTGYFWILEGPRMAVVLLNFLFLMNIVRVLIVKLRQSR
SSEMEQVRKAVKAAVVLLPLLGITNLISMAAAPLEVTIWFALWSYSTHFLTSFQGVFIATLY
CFLNGEVRLALNKSVSLYMSVRTTERQARRQSTFSACQPQRIESENETAALGVNAINAPTI
EHTSWLQVCWHGKNIQDQKQPADYRMKQIVTNQTVKIPEGLTVTVKSRRVTVTGPRGTL
KRCFKHLAVDIHMVSPRLLKVEKWFGTKKELAAVRTVCSHISNMLKGVTKGYQYKMRA
VYAHFPINCVTTENNSVIEIRNFLGEKFIRRVKMAPGVTVCNSAKQKDELILEGNSLEDVSR
SAALIQQSTTVKNKDIRKFLDGLYVSEKTTVVQDDE
>Hheb104700.1
MALNASDQSEEVNYIHGTLNYINELNDSLLIKEFECLKAQHFEALEGRKTESTFCQVDWD
RLLCWPTSPPGTLVKQPCFEQLHGIHYDSSQNASRWCWQNGTWDSYSNYSQCQELRMN
VIESGIEITTTLYFIGYTISLSTLLVAVAIFAYFKELKCLRNNIHTHLMLTYIFADLMWIVTTIM
QVSMQTGMPTCIIFFSLLHYFHLTNFFWMFVEGLYLYQLVVKTFTGDNIKLRSCLAIGWGA
PMVIVIIWTALKSWAISADQSSQNVALYRHCPWMVHHNFDWIYQIPAIAVLAINVMFLFMI
MWVLITKLRSANTVETQQYRKASKALLVLIPLLGVTYVLVLTGPESGQVADIFTYIRAILLS
SQGLLVALFYCFLNSEVQTAVRHRLSRWSTARNLRSDRKFYSNCSPRSRTESI
>Hheb039610.1
TPGNLTTTVGSTVQEEELRRIILERKQQCMEMLALNTTLPAEPYCPGIFDGWSCWPNTPAG
QKAYTKCPPFVTGFDTSFYAHKVCEADGSWFRHPVSNQIWSNYTTCVNVEDLNWQQGIN
VIYETGYTISLIALVLSLAILTYFRSLRCARITIHMNLFASFAVNNTLWLMWYGLVLPNTEVL
VENGITCRFLHVILHYFLLTNYAWMLCEGFYLHTLLVSAFTSEHNLVKWLMAIGWPTPAII
VLIYAILRGTSDDPDDNSQCWIDEGNYINVLVYPVCVSTLLNLLFLFNIVRVLLTKLRAGPA
IGSRPSRSMLQAFRATLLLVPLLGLHYLLTPLRPPKNHPWSQSYEIISAITASFQGLCVAILFC
FCNGEVIAQFKRKWEGSALMRKRANSCTATTVS
>PpB1 PPU07227-RA

MALILKFLASILVLHYFDEVESVSNSTGESHLVEMLRRETMLEELYKCLMDIGPCNSEESV
HLRDAIPMVFMTYCRTCDNHQKHTLLIFLMWHMFHSLDTLLIDDNDSTEMNIPLLPGNNT
LSGVPTAFIAVGRLCQNCLLEPAYFAYAMTDCAFNYFCDIKMPGNTTMAGSTMQEDELRL
LKLERQQECLGQLAVNATPPPEPFCPAIFDGWSCWPNTPAGTTAYAPCPDFITGFDPTLKAH
KECEPNGTWFMHPVTHKIWSNYTTCVNLVDLSWQQQLNVLYETGYTISLVALLVSLGILT
YFRSLRCARITLHMNLFASFAVNNALWLIWYGSIVANAQLLLNNGFMCRFLHVILHYFLLT
NYAWMLCEGFYLHTLLVSAFTSEHKLVKWLMALGWPIPAIIVTIYTVLRATSNDNADTLQ
CWINEGNYMVVLIYPVCVSTLLNLLFLCNIVRVLLTKLRAGPAIGSQPSRSMLQAFRATLL
LVPLLGLHYLVIPFRPEKGHPWEYAYGVMSAITASFQGLCVAILFCFCNGEVIAQFKRKWE
GTAFARKRANSCTATTVSFVRSTAGPMAGEEKV
>PpDHR PPU05559-RA
MSNASLSLAYEAYEPDCQLLTSIYADENQRKEAKLLGDGYSCAAAWDSVLCWPKTKVGS
MAVQPCFEELHGIRYDVSQNASRWCWANGTWDSYSNYSQCRELHQPESTVGESGVEITT
YLYLVGYGLSLSTLIIAVIIYLYYRELKCLRNIIHTNLMFTYILADFLWIVMTVSQVSLQTNIP
MCIIFYSMYHYFQLTNFFWMFVEGLYLYVLVVKTFSGDTIKLKMCLFIGWGLPFIVVVIWG
MTKALAEDSITAGSHNEALSNHCPWMIPHPYDWLYQVPAILVLCMNVIFLFMIMWVLITK
LRSANTAETQQSRKAAKALLVLIPLLGVTYILVIAGPTEGQAANMFTYSRAILLSSQGLSVA
LFYCFLNSEVRNALRHHYQRWSTERSLGHHPRQYAQYAPRSRTESIRLYSRHGGPGSGPGL
DGCTNNHIKQHHNYEHSIHEHGNHVDTTMIQKEPHLMMSENSCVTAIVTVDDESTAATTY
CEPGRCC
>PpB2 PPU09199-RA
MKSANKRLDTKLYDVIKSDDVAASANRRRYGGGGGDGSSSIRWRTDDMDFPSANGNDT
QVHCELQNRNFIMPEDEIWCNWAWDTILCWPPTRASTVTKLRCPSANGIDTRKFVEKRCG
DDGRWEGRAGTAGERDSPNGWTNYTPCFTPEMLLLIRKLYTGSEDLAKVKLDIAAKTRY
LEFVGLSISLTALLISLSIFCRFRSLRNTRTRIHKNLFVAMVIQVVIRLTLYVDQAVFEPNGLQ
HGIQNTVLCEASYVLLEYARTAMFMWMFIEGLFLHNMVTVTVFQESSHYRTYRLVGWGY
PIAMTLTWAIVTAFYYHPSKCWWGYNLTLYFWILEGPRMAVILVNFLFLLNIIRVLVVKLR
QSHTSEIEQARKAVRAALVLLPLLGITNLISMAKAPLDKSIWEFALWSYTTHFLTSFQGLFI
ATLYCFLNGEVRLALDKTISVYMSVRGTDLTRRQSTISGCQPHRMASVIEVEESEIRPGAG
WIRLCCRGGNMPPPDRPAETTL
>NvB1 XP_003424370.2
MDDLELASEVPGNTTMAGSTMQEDELRLLKLERQQECLGQLAVNATPPPEPYCPAIFDGW
SCWPNTPAGTTAYAPCPAFITGFDPTLKAHKECEPNGTWFMHPVTHKIWSNYTTCVNLVD
LSWQQQLNVLYETGYTISLVALLVSLGILTYFRSLRCARITLHMNLFASFAANNALWLIWY
GSVVANAQLLLNNGFMCRFLHVILHYFLLTNYAWMLCEGFYLHTLLVSAFTSEHKLVKW
LMALGWPIPAIIVTIYTVLRATSDDNADTLQCWINEGNYMVVLIYPVCVSTLLNLLFLCNI
VRVLLTKLRAGPAIGSQPSRSMLQAFRATLLLVPLLGLHYLVIPFRPEKGHPWEYAYGVMS
AITASFQGLCVAILFCFCNGEVIAQFKRKWEGTAFARKRANSCTATTVSVRWRERRRYDY
QSAPSADPRDDNHSNNNRFSGSRDLPEPQLQPLTIVGTAPAETTSNGSAANHAPRLLLRRS
DSLLHEAANTTSNNNNNNNHQSQC
>NvPDFR XP_003427082.1
MDFPSANGNDTQVHCELQNQNFIMPEDEIWCNWAWDTILCWPPTRASTVTKLRCPSANG
IDTRKFVEKRCGDDGRWDGRAGTAGERDSPNGWTNYTPCFTPEMLLLIRKLYTGSEDLA
KVKLDIAAKTRYLEFVGLSISLTALLISLSIFCRFRSLRNTRTRIHKNLFVAMVIQVVIRLTLY

VDQALFEPNGLQHGIQNTPVLCEASYVLLEYARTAMFMWMFIEGLFLHNMVTVTVFQES
SHYRTYRLVGWGYPIAMTLTWAIVTAFYYHPSKCWWGYNLTLYFWILEGPRMAVILVNFL
FLLNIIRVLVVKLRQSHTSEIEQARKAVRAALVLLPLLGITNLISMAKAPLDKSIWEFALWS
YTTHFLTSFQGLFIATLYCFLNGEVRLALDKTISVYMSVRGTDLTRRQSTISGCQPHRMAS
VIEVEKAEIRPGAGWIRLCCRGGNMPPPDRPAETTL
>NvDHR
MSNATLGNLRLGEPEPDCQLLMSIYESKNQRKEDELVGKSCPVGWDTLLCWPKTKAGSM
AVQPCFDEFNGIRYDVKQNATRWCWPNGTWNGYTNYTQCRELYVPELPVDSSGVEITTN
LYLIGYGLSLSTLIIAVIIYLYYRELRCVRNIIHTNLMFTYILANFLWILMIVSQVSLEKNIPICI
IFYSMYHYFQLTNYFWMFVEGLYLYVLVVKTFSGDIVKLKTCLFIGWGIPLIVVLVWGIIK
ALAENSAAAGSPVRALSNHCPWMIPHPYDWLYQVPAILVLCVNVIFLFMIMWVLITKLRS
ANTPETQQYRKAAKALLVLIPLLGVTYILMIAGPTEGQVANLFAYSRAVLLSSQGLLVALF
YCFLNSEVRNALRHHYQRWSTERSLGHHPRYYAQYTPRSRTESIRLTLKEHKILES
>BmB1
MNDPDRIARDLLLCEEFNNRTLPPPGLYCEGTFDRWLCWPHTPANSTAYGSCPEFVPGFRP
DLLAHKECTANGTWYKHPETGLPWSNYTTCVVEEDDVNHIIVVYEAGYSVSLVALLLSL
AILLYFRSLRCARITVHMNLFGSFAVNNALWLAWYGLVVRDPTTLQEPPVWCCALNAVLQ
YAMLTNYMWMLCEGMYLHTVLVSAFISERRLVRALVAAGWMLPLPCILIYATRRALDGD
PLCWAEEPESRPELAVPVGLAVLLNLCFLCNTVRVLCTKLRAGGAAGGAARPSATALHAL
RATCLLAPLLGLQYLLMPFRPANTVSWWRAYEYATAAATSLQGLCVAVLYCFCNGEVLAQ
LRRRWRALTFRPRANSTTATTVSFVRSAATAGGEDKA
>Dm_CG17415   DH31
MSDQIGNPNATFSGSGSGSGTNVASIAESVAESGPDFDALRAACETRLNASGQLAGSGGPG
AEAGTHCAGTFDGWLCWPDTAVGTSAYELCPDFITGFDPARYAHKECGLDGEWFKHPLT
NKTWSNYTTCVNLEDLNWRHTVNLISEVGYGTSLLAILLSLAILGYFKSLKCARITLHMN
LFASFAANNSLWLVWYLLVMPNSELLHQSPMRCVALHITLHYFLLSNYSWMLCEGFYLHT
VLVAAFISEKRLVKWLIAFGWGSPAIVIFVYSMARGLGGTPEDNRHCWMNQTNYQNILM
VPVCISMFLNLLFLCNIVRVVLLKLNAPASIQGSCGPSRTVLQAFRATLLLVPLLGLQYILTP
FRPAPKHPWENTYEIISAFTASFQGLCVAILFCFCNGEVIAQMKRKWRMMCFSNRPRTNSY
TATQVSFVRCGPPLPGEEKV
>BmB2   NP_001127733.1
MTVESRRLTPELSLISTGVRYCQPRFDGYLCWPPTAAGETTYLRCPVARLSDSTKNAYRRC
GIDGLWNTKKFNETSEIGWTNYTPCFPMEIRNMLNELYEEDETGAQDKFNVALRTRYLEIF
GFTLSFIALSISLYIFIHFRALRNHRTRIHKHLFGAMLVQVLIRLTVYIDQAVVRSSISSTDDN
TTMTRGIDNMPYICEGSYVLLEYATSAMFLWMFMEGLYLHNVVAANNLRERVPYQWYC
VWAWGAPVVITSIWTILTALKYRGEKVQTCWYGYNFTGIYWIVQGPRLIVILINFVFLLNIL
RVLIMKLSKSARREIVKVRKAVRAALVLLPLLGITNIMNMFEAPLSSDPIRFAVWSYLTHFL
RSFQGFFIALIYCFLNGEVKQCLSKAYTNYMAERALLVRQNAICVPSMDEEPAKEKKASY
LSCCFPSKTEKRTSKDYRACALDLNKDEEPAKEKKASCLSFCFPSKTEKRTSKDYRAGAL
DFNNTARARRLELHMVSNSQLPTPLQSPCPAVVRRQMELQELKQSTPLIPRRHIVSAPKRA
VRIPFDDDDEAVTKEPFSCYADTLEMAELGAVSPWVYRRRCSPPEMGIFDTCSLTRGSCDE
QPPIHIERCIYATRAIVPSSPYVADTDAREVSK
>Dm_CG13758   PDF
MTLLSNILDCGGCISAQRFTRLLRQSGSSGPSPSAPTAGTFESKSMLEPTSSHSLATGRVPLL

HDFDASTTESPGTYVLDGVARVAQLALEPTVMDALPDSDTEQVLGNLNSSAPWNLTLASA
AATNFENCSALFVNYTLPQTGLYCNWTWDTLLCWPPTPAGVLARMNCPGGFHGVDTRK
FAIRKCELDGRWGSRPNATEVNPPGWTDYGPCYKPEIIRLMQQMGSKDFDAYIDIARRTRT
LEIVGLCLSLFALIVSLLIFCTFRSLRNNRTKIHKNLFVAMVLQVIIRLTLYLDQFRRGNKEA
ATNTSLSVIENTPYLCEASYVLLEYARTAMFMWMFIEGLYLHNMVTVAVFQGSFPLKFFSR
LGWCVPILMTTVWARCTVMYMDTSLGECLWNYNLTPYYWILEGPRLAVILLNFCFLVNII
RVLVMKLRQSQASDIEQTRKAVRAAIVLLPLLGITNLLHQLAPLKTATNFAVWSYGTHFLT
SFQGFFIALIYCFLNGEVRAVLLKSLATQLSVRGHPEWAPKRASMYSGAYNTAPDTDAVQP
AGDPSATGKRISPPNKRLNGRKPSSASIVMIHEPQQRQRLMPRLQNKAREKGKDRVEKTD
AEAEPDPTISHIHSKEAGSARSRTRGSKWIMGICFRGQKDKCVMPGSQKTQQIFMTSQMPP
TSTLAAVATTITTTSTTTTAAKTTIASIATIATMTKSKAKAKAISKSHQIQMPKA
>BmB3 BAG68437.1
MFRCCSVEDLLYLLILFVSLTKGESFSDDAFDLLDGDLDLWHERGSRVAIREDAPTCERQS
FYICEEPLNGVPPEAHKTCNYRNVDYHEQVFRWVAGRGCIIYTPSFLFVGGANAMNTSQC
FYRNYRVRCLEIPKEDGTCGCYPFDPSFAEVANIIREAIIPSAHGRWERCFYAAQDCCSHYM
LEDTNSIDKNECGVTFDGWTCWMAAEAGTVSNAVCSEFAYSNTGPSCNHFSSKECYQNG
TWEVESVYNTCSVTPRLVQRYRFHIAMLSISVASCLPAVFIFFFYKRLRITRVALHRNLLIAII
LRNTFVIISRNEIYLDELQNTGDTVMTIHGLACRFLAIGERVAGNAVFVCMLVEGIYLHRLI
VAVFKQKLNIKSLYAIGAVIAVAPVIAWVAVMAVHNDHSCWLVYTIEHVQWILDVPRVAILL
VNTVLFGDILRVLLTKIRNNENSHQLSTTKATLFLMPLFGTQFLLTAFRPNTTDCTGEQIYY
YVSYTVEGLQGFVVAILYCYINKEVRSLIKATYKKTESAVVSRIRRDSTHPSMSVDPSSGRR
MTYSTGIPSQGDIIKEQYAVVAPKLHVAEIISIQPSERLVEIIEPVYETISNSVNNDSYDALDR
SDNDPKGRSKIDDYYNFTNASSITIDCPEWIRCVSSPNSSIYNNSLNDNENKYLASHIPDIPE
SCNEMKHSKNEQAEKNNLPKHDIGENNEDFEDYDADFETKTSADDSGNMLEEIMQCIEN
KNDLSIPNLDPERLCPNRNGEDKIIFLNE
>BmB4 NP_001127735.1
MLSRFLNKLFSQTNVTENPQLKVLYTRYEECFVSNETHNTIVQSEYDENAPRCPKTFDGFS
CWDETPSNTTVVQNCPELINGFDPSRTAFKECLENGTWLEHPESKKVWTNYTTCIDYDDL
RFRNVINNMYVGGYAISLIALILSLMIFVFLRHFQYKRTRIHANLFISFILNNIMWIVWYKT
VVDYIEVVQENTIWCQSLHILTYYHMMTSYMWMFCEGLHLHVGLVSINEYISVRSYCAIG
WAIPALVVALYTGVRMQLRYGTERCWMDQSHALWIIVIAVVIILTLSFLFLVNVIRVLLKKM
QPPASNQPNEAAKKAARATATLVIYFMIPLYGLHFILIPFQRAPESIGEKIYQVVSALLTSLQ
GLCVSILFCFTNNDVKTALTNYLARYKRKTEAIQMTGITTGESAVNPNAA
>BmDHR XP_004933474.1
MIAAIIERLYGQEMLNDTAIIEAKTKVPCTIRKYFCRLPMFTFGALSLDKLEHKPRVNIFIM
YIFNGKLEDLQTLESAESVIENATARTECLARNANLTEYYCPAYFDGLLCWNPTPSHTVAV
QKCFKEFFGIKYDETQNASRLCLDGVWQNYTDYSNCTERIANVSPTDVASLIYLTGYSLSL
AVLSLAVFVFLYFKDLRCLRNTIHTNLMTTYILSACSWILNLALQNWSDEAQQDQTSCMIL
VICMHYFYLTNFFWMLVEGLYLYMLVVETFTAENIKLKVYTTIGWGAPAIFITIWVVSRCF
VNVMPSTGPDGLALAGEAKMCIWMHEHQVDWIHKAPALAGLALNLFFLVRIMWVLITK
LRSANTLETEQYRKATKALLVLIPLLGITNLLVLCGPSDDSWFAYSFDYARALMLSTQGFT
VALFYCFMNTEVRHAIRYHVERWKTGRNIGGGRRRGASYSKDWSPRSRTESIR
>Dm_CG12370 DH44-R2 NP_725175.3
MADDDLRALVDSLDDASQEDLAKVIANFSVDMLQRASALIGAQQGSSGGQLQNRTLQCQ

QQQQREEEQASLEALASGGKRILQCPSSFDSVLCWPRTNAGSLAVLPCFEEFKGVHYDTT
DNATRFCFPNGTWDHYSDYDRCHQNSGSIPVVPDFSPNVELPAIIYAGGYFLSFATLVVALII
FLSFKDLRCLRNTIHANLFLTYITSALLWILTLFLQVITTESSQAGCITLVIMFQYFYLTNFFW
MFVEGLYLYTLVVQTFSSDNISFIIYALIGWGCPAVCILVWSIAKAFAPHLENEHFNGLEIDC
AWMRESHIDWIFKVPASLALLVNLVFLIRIMWVLITKLRSAHTLETRQYYKASKALLVLIPL
FGITYLLVLTGPEQGISRNLFEAIRAFLISTQGFFVALFYCFLNSEVRQTLRHGFTRWRESRN
IHRNSSIKNRRHRASKDYSLRSRTESLRLTSTSPIPTGHYE
>Dm_CG8422 DH44-R1 AAF58250.1
MSDHNHIDSVNASGSDPLLDLHNLDGIGESVELQCLVQEHIEASTYGNDSGHCLTQFDSIL
CWPRTARGTLAVLQCMDELQGIHYDSSKNATRFCHANGTWEKYTNYDACAHLPAPESVP
EFEVIVELPTIIYYIGYTLSLVSLSLALIVFAYFKELRCLRNTIHANLFFTYIMSALFWILLLSV
QISIRSGVGSCIALITLFHFFTLTNFFWMLVEGLYLYMLVVKTFSGDNLRFNIYASIGWGGPA
LFVVTWAVAKSLTVTYSTPEKYEINCPWMQETHVDWIYQGPVCAVLIINLTFLLRIMWVLI
TKLRSANTVETRQYRKAAKALLVLIPLFGITYLVVLAGPSESGLMGHMFAVLRAVLLSTQG
FSVSLFYCFLNSEVRNALRHHISTWRDTRTIQLNQNRRYTTKSFSKGGGSPRAESMRPLTS
YYGRGKRESCVSSATTTTLVGQHAPLSLHRGSNNALHTMPTLAANAMSSGSTLSVMPRAI
SPLMRQGLEENSV
>Nl B1 BAO01101.1
MTGSNNTTEIVYEQEIIKQKYQQCMVQLELSNQTGPHFKEPYCKGVFDGWSCWPDTPAG
TVAFAPCPEFITGFDPTFIAHKDCTANGTWFKHPISGLVWSNYTTCVNMEDLDWSQKINTL
YETGYAISLVALLLSLIILSYFKSLRCPRITLHMNLFTSFAINNLLWLLWYRLIVPYPDIVKQN
GVWCQCLHVILHYFLLTNYSWMFGEGFYLHTLLVSAFISEERLVKWLTRLAWIIPFIFITIY
MKLRLFFGQTDKCWIEDSPYMSVLTVLVCCSMVLNLIFLCNIVRVLLTKLRSTPSGMASST
SQAPSRSLLQALRATLLLLPLLGLNYLVTPFRPHKGHPWEKYYELTSAITASFQGLCVATLF
CFCNGEVIAQIRRRWQYIMFRPRANSYTATTVSFVRSTACPNTGEDNV
>Nl B2 BAO01102.1
MPVRRVSSDGNRDPLVAHRASLLAVPGLHPTSGVGSDVGSCRQLDLSLTSPADTTAAAMS
DDISPECVSPAVLLVPPPYPEASDEVSSTSKSDQGIQITVVEDTTEDDKQPQSQPQPQSLHH
RPVANKRSLELCAARYHEYSAPLGVLYCNWTWDQVLCWPPTRAGTTATQRCPRDKGIDP
TKFATKKCSAEGRWEGKHPGEEATSPQGWTNYTPCYTPEMLELFRKLYVGSEKAAMKKL
EIAERTRTLEIVGFSISLAALLISLAIFCHFRSLRNNRTRIHKNLFVAMVIQVVIRLTLYIDQAL
VKGNSLTGQRVSSSSSSSGGFRQGIDNTPVLCEASYVLLEYARTAMFMWMFIEGLYLHNM
VTVTVFQEKSYYAAYTMVGWGVPVLMTAAWAMTTASTLGSTRCWWGYNLNKFFWILE
GPRLIFILMNFLFLLNIIRVLVVKLRQSHTSEIEQVRKAVRAAVVLLPLLGITNLVNMTEAPL
TRAVWEFGLWSYTTHFLTSFQGLFIAILYCFLNGEVRMAVRKSVSTYLSLRPQHFTPQRRN
SAFVSANMTEQVVSTPPPRPATSAPRSWFRSCCSHPSQPTPETRV
>Nl B3 BAO01103.1
METFLEGDLTLEQIIITRRENCEDIKDFTPANDTGEVYCPREFDGWSCVNWTSAGQVAHFP
CPYFILGFDPKRFGQRTCLMDGTWFRHPDSNKTWSNYTTCVDLEDLELRKHVNFIYKTG
YSISLAALIISLFIFFYFKSLSCTRIQIHKNLFLSLTINNCLWLIWYEAVVDNLPVLMTNGLGC
QALHLLVQYFLVATYLWMFCEGLYLHTLLVVTFLTESRVMPLLYFIGWGVPAILVLIYAAM
RSSLREEKLHCWIHESLYSWTLSGPVCISMLANLVFLINIVRLLLTKLHTGQPVSPKASFQD
PQSSVRLRRRNTVLSQTGVMSGRTRKAVRATFILIPLLGLQYILMPFRPQQGAAWEPAYQII
SAVVTSYQGLCVASLFCFFNGEVSKEGN

>Nl B4 BAO01104.1
MSPEQQNASLRFFKKLKEDCDHKRRLKLVSAAEGVTEIEELQCPAVFDGFTCWDATPAGE
NAFAPCPDFVTGFEKTRFAFRSCMENGTWFRHPQTGKYWSNYTTCVDMEDLEFREFVNS
LYVTGYSVSSAALLISLLIFLTFRSLRCTRIAIHVHLFVSFAANNLMWIVWYKQVVGVTKV
VQENDIYCQALHIILQYLMVANYMWMFCEGLHLHLALVVVFVKDDCAMRLFYFIGWCL
PLFITTVYTLVRMSYPHDTSQCWMGDSYSQWVLIVPVLLSMLASLAFLINVVRVLLTKLH
CNSANPAPIGMRKAVRAALILIPLFGIHHILIPFRPEPHAPLEMTYQIFSALLVSLQGFCVSVL
FCFANVDVHCAFKNVLRRMRRRRAGDNNGTVTQQTQTREMV
>Nl B5 BAO01105.1
MGRAQYASHCPRSWDSLLCWPPTKPGHTATMACFPELNGIKYDTTLNATRRCLLNGTWD
NYTDYTSCKDLSPDQPDLEPGIEVTTMIYSAGYALSLIALVLAVSIFLYFKDLRCLRNTIHTN
LMCTYIMADFMWILNITVQMSMPTNVPACVILVVLLHYFYLTNFFWMFVEGLYLYMLVV
ETFSSKNIKLRAYVCIGWGVPCAVIIIWTIVRSLMPPTSDNMGLSGIVLKSCPWMATHNID
WIYQGPAAVILFVNVIFLVMIMWVLITKLRSANTVETQQYRKAAKALLVLIPLLGITYILVI
YVPSHGVTANPLAYCRAILLSTQGFTVALFYCFLNSEVQHTLSSHFERWKEARQIGGETLC
>NlB6
MTPGGELVSLPCPEYYRDFDPQKRATRRCLEDGRWYWSDESNATWTNYTECFSKKAPYV
FPTPSTLISSYLPIVKAVSQIGYSVSLITLIVAFCILATFKGLRCPRNKLHMHLFLSFILRACTT
LFKDLVTDSLSSRLVISETSVSETVILHQQTEDWTTCRAVTSLWQYFLLANYCWILMEGLY
LHNLIFLALFTDSSSIALYIVLGWGLPAIFVLPWIFVRAAWENTLCWTTNSNPYYLLLIRGP
TTVSILINFALFINIVRVLLSKLQASICEENKKFRKWAKSTLVLVPLFGVHYAIFIGMSYVEG
SELEIAWLFGDQLFASFQGFFVAVLYCFLNGEVRTEISKKWRRWWRSRKSSPHNSNYVDK
NAYGRCSTLRGCFNALAHGKNGYNMREGDYSKREST
>NlB7
MTEANDDDELTIKVKELEAKCHLIQNRIEGVGLTCPPVWDGLLCWPATPAGIVKSLQCPD
YFHGFNPTRQATKRCLEDGTWYWSHESNSTWTNYTQCFSKETVANTDFSSHHQILFRYVP
IVKFVSQVGYSVSLFTLIIAFCIFATFKGLRCPRNKLHMHLFLSFMLRAFSTLFKDLETNRLE
RFSATSGELISLREDWASCRAVVTLWQYCLLANYSWILMEGLYLHNLIFLALFSDTSSISLY
VALGWGLPLLFVAIWALARIVWENSMCWTTNENALIVMLVKGPTTISVLLNFVLFIKIVRV
LLTKLQASVNEQNRRFKRWAKSTLVLMPLFGVHYAIFISMSYVDCGPEIEIIWLFGDQLFAS
FQGSFVALLYCFMNGEVRAELNKKCLRRWPSNSAANKRRRQSETTGQYSNTCAVRPHHY
GKGRDNRKSLGRLETSVTSITTSCPFDPSSRKSLPSNGLHRNHSESTWLTEQSSFVNSKVTA
GNGIELMALHEKYEEL
>Tc67 XP_969953.1
MTNIYVKKILDNQETARKAAKEKCISMFGPGVLQEFAEPVYNETNKTLKCPAFWDTILCW
PDTPSGTVINQSCPVYVAGFLASSNATRQCMENGSWYIRDNRTWSNYSSCYKDKITTIYLD
LNKTNISGVLQNYAPVVQVISETGYIVSFATLIIAFAIMLFIKKLHCARNILHMHLFASFILRA
LTFIVIKSTFVEGLGLPSDLNYRNGSLYFDINSETNNWACKLLTSLWQYFITANYSWILMEG
LYLHNLIFRALFADSSNSIKWYVVMGWGLPLIIVGFWVAARLLVEDNLCWTTHENYDVFL
IIGIPTMVSILINLLLFMRISMVLYSKLRSPINEDSRRYQKWVKSTLVLVPLFGVHYALFLAL
YYLIKTNKIVEVVWLFCDLLFGSFQGFFVAILYCFLNGEVKSEIQPHLYYFLTYLATNKYSK
CLFPCRKKFLRSVVGRSSVCTTMSCSSLYTNGVLHRNSKCRLDSLPKIKTTDKPKDHFCN
NTSRRNSRSQRHSQAFNSSATIPTAETTLCIENNSGKCRSESEINEESLKMVVHSDF
>Tc68 XP_975039.1

MSEFQDILNTAKRKCYASKTTEIITGKFCELIFDDILCWPPTPAGLLANQSCSNEYIKWTRN
NYATRQCDSDGQWFIPENSTESWTNYSQCGNISEFYPILDDSLRNHTLYNKWLPIIKNITQC
GYILSTVSLIISLFVFIRIKRLHCARNKLHIHLFASFVMRALMSLIKDGLFIEGTALPHEIIQIN
GKLVYNKTNFSWVCKAIISLWNYFIISNYMFLLMEGAYLHNLLFLKLLSENGVVIYYSLG
WGIPLLFIIPWIVLKAGNENIYCWTTKSSKFIAMLIDVPIGLTVVINFILFTIIVRILFVKLTSM
YIQQRWTKYQKLIRAILILVPLFGIPYTISFVLSFYALEDQTFEIMWLFFDQTFTAFQGLFASL
VYCLLNSEVQMEIMRKYNSFKDRNREFKRRSRTISHTQQIPLTEELQEMPHNAGIKDQLCI
NKTSDYF

>Tc69 XP_971738.1

MCDEIKECAQITRTTRTIEMVGLSFSLISLIVSLIIFFQYRVLKNNRTKIHKNLFIATLLQVVF
RLIKYVDQELKAGDRIIENTPILDEACITLLEYSKTAMFTWMFIEGLYLHNVITVTVFQEYS
YIKIYFYVGWTAPAVITAVWVWTMKMVRSNWGFYYFLPYYWILEGPRSTIIVVNLLFLINII
RVLIVKLRESHTSEIEQVRKAVRAAIFLLPLMGIANILFWMGYRFTQGWKLALWSYTSYFL
NTFQGFFVAILYCFLNGEVQTAVKNSFYLHMSLRNHDYTPCRNLTLISTAPDPEQHIEKEST
NWIRYCLKQNKKSPEEKEISVCDLNDRRNSAIVTALTMVETKPLTRFVVATRISQYVAKAIR
RNRYDDNRRQKFRTFATPESHNEHGERKNLFPSIAISMTLMSPYHKVYKSDITT

>Tc70 XP_969030.1

MEQMRQHCLVFYKCEKDGEWNFNEQFNKSWVNYTTCINIEDFEFRQQIILIYCVGYGVSL
VALLVSLALLTYFKSLRCARITVHMNLFSSFAMNNFLWLLWYSLVVNDQDVLHENKLWC
RVLHVVLFTFLISNYSWMLCEGIYLHTVLVSAFISERRLLRCMLALGWGIPLLTTSIYAPVR
SVLGENVDELGRCWTQDGRFNKILMVPVVITVFLNVIFLVNIVRVLLIKLRKGPANGGSGS
GASRTSLQALRATMLLVPLLGLNFLLTPFRPEANHPWEYVYEVVSALTASLQGIQPFLMFF
WYRDQNHSW

>Tc71 XP_008193920.1

MDVTNGTNCGGKYTRPGYCPEIFDEMLCWPETLGGTTVNQSCPKKMGYDSRRFAYKDC
LENGSWFKHPKSGKIWTNYTTCVDHEDLAFRTHINHLFVIGYSISLAALVISLAIFFTFRTL
KCTRIRIHIQLFISFALNNLMWIIWYKEVVPNPFVTIRNELWCQALHLVVHYLMLANYMW
MFCEGLHLHLALVVVFVRDAETMKWFFALGWGAPFIIVLIYSVVRIFILKDNYMCWMAD
SYYSSWILTAPVCISLLVSLIFLINVLRVILTIMHPNSANPAPMGLRRAARAALILIPLFGLQHI
LIPFRPDMYDPYEHLYQYVTVVVVTLQGLCVSCLFCFANQDVHQAIRGFMHRKVYRTTR
WSNYHYTGAADSAGVYVVNGSSHCNNVGLLSLKRKSTTTVKL

>Tc72 XP_968807.1

MRFNYIVVILISVCLLISTKNIQAQAKLCRSSQGLIHRSFWNRWTGYLCYHYDKRVKFKYN
YTIIPYDSSYIPVTFINNKTDFFWYDFRNETSLNYIRPSYKSDTIFEKWKTCAQEASNCCDY
SMTSENINPSDEYPCPAIWDDWSCFSPAKAGSVSKILCPDLTFSSEIRECELQAQKECFRNG
TWKSKTDYGPCSVPAVLKTRHRFHIIVLSVAAVLSAPAVAIFYSFRAFRLQLRFILHRNLILVI
IIKNLLVVITKEMVIMEALTSDGDDTILNGNSVTCRVLAFFENVAKNGVFAAMFLDGFDLH
RSIVRPFADAMSSRFVYCAFFVLSCCPALIWALLRGLHQGEFCWVVDNTGDQWVTDAFR
LTILTVNFVLLVDIIRVIVLKLRHDSVSQQTKTTLRVTLFLVPLFGVHIVITVNRNMVPDNTC
DSMDVYYFATYLIEGLQGVLVAFLFCYINKEVHHEIMNAWRKTVICLQQKLDAKSERTTS
ATIVESMTCY

>Nl A50 BAO01100.1

MQRRAVATALVTAVTLTHFTVKADQDGVEPHPTWPFAVKADQNGVEPHPTWPPPPQLPPM
VQSHEPGCSCSDHRDPPRLLVKCKCRGDHLQRVPSDLQRGLHILSITRAAIEVLAADSFQP

YRESLTDLSLVRLPNLRLIEPGVFNNLPHLRTIDIHSAPMLTIISDAVFQTHLPRLRIFRCTNT
GLQQIPALRDLESKHQLHLVDLDSNRISLIPERSFYITSDQVSLNYNQIEEIEAFAFHNSTIAT
LSLKGNRNLHILSEDSFSGLNSLRKLDLSETAITFLPTLGLRGLDELRLQGTTSLKVFPSVYS
FDSLKDVYLTYSCHCCAFRFPARHDPAGFRRHKEFVEKMIRDCSSSFSGDNVSYNDRANQ
QNISFGPANTSFWNNIEFTSGSSTTSSEETFHSIVAVSPNGQLQVRCGHMLGGGGSKGRLQ
GEGPRCFPAPDAFSPCEDLLGSGWKVRISAWLVSLFALVGNTCVLLVLLSSRFRMSVPKFL
MCNLALADLCMGLYLLLIAIADARSQGAYFNYAIDWQNGIGCQAAGFLTVFASELSVFTL
SVITSERWYTITYAIHLNKRLRLGSASRIMAAGWLYSIAMAALPLLGVSSYSITSICLPLQTS
SAVETVYLATMLAVNGVAFGVVCVCYGLMYASIRGQGQGRSGRVRSDLSVAKRMALLVL
TDLVCWAPVAFFGLTALAGHPLIDLPSAKLLLVSFYPLNACANPYLYALLTRQYRRDLLAL
FARYGICSKQAATRHRGGLAGGGGHGEARGTGGRGSRAGEGEMVLGHSPHHTGGLYSPP
>GL561032
MSDETGNQSFLDPHAELVNSRYLQCLTTINESLSRSLQGNICGLQCEATFDGWSCWPATSA
GETAFAPCPHFITGFDPLIFIYFQMSMDTDMVSCIILVILLYYFHLTNFFWMFVEGGLYLYM
LVVETFNRENIKLRAYLAIGWGVQRGCAWMSPNSSDWINQAPAIIVLAVNLIFLVMIMWV
VLITKLRSANNVETQQYRKAAKALLVL
IPLLGITYILFIVGPTEGQYAVIYSYIRALLLSTQGLTVALFYCFLNTDVQNTVRHHLSRWRE
ARDIDARRYTHTKDWSP
>RPRC004735
MGTWFRHPASNKTWSNYTTCVDLEDLKMRTQVNMIYKGGYAISLAALTLSIFIFFYFKSLT
CTRIQIHKSLFISLAVNNLLWLIWYEAVADNLPVLFANGFGCQLLHILVQYFLVATYLWMFC
EGLYLHTLLVVTFVTESKVMPFLHLIGWGVPAILVTIYAILRMSNKEDSVHCWIHESLYSW
TLSGPVLVSMIANFVFLINIVRLLLTKLHTTAQTSNENAPSGRTKKAVRATLILIPLLGLQYI
VTPFRPNQGTPWEYAYQVTSALVASCQGLCVALLFCFCNGEVVAVMRKKWRQCRISKKRP
WHSCSGVTSVSRHRRI
>GL562334
LIFIYFQMSMDTDMVSCIILVILLYYFHLTNFFWMFVEGGLYLYMLVVETFNRENIKLRAYL
AIGWGVQRGCAWMSPNSSDWINQAPAIIVLAVNLIFLVMIMWVVLITKLRSANNVETQQY
RKAAKALLVLIPLLGITYILFIVGPTEGQYAVIYSYIRALLLSTQGLTVALFYCFLNTDVQNT
VRHHLSRWREARDIDARRYTHTKDWSP
>GL563066
ADYCPRSWDGILCWPPSPSATIVYLPCFEELHGIKYDTSRKFLFLCINASRWCLWNGSWAN
YSDYDSCSHLQIPPFAADPGLVVVTMIYLIGYGISLIALCVAVAILIYYKLRCLRNTIHTNLM
CTYILAAFMWILNFTLQVSLIFIYFQMSMDTDMVSCIILVILLYYFHLTNFFWMFVEGGLYL
YMLVVETFNRENIKLRAYLAIGWG
>RPRC009680
VSKRCLENGKWEGIGETGWTNYTTCYSPDLIQLFKKLFTGPYSHDAIKYQIAERTRTLEIY
GFSISLAALFISLYIFSHFRVLKNNRTKIHKNLFAAMVAQAVIRLTLYVDQAIIRARKVQGID
NTPILCEASYVLLEYARTAMFMWMFIEGLYLHNVVSVRVFQETFHYKLYTSLGWGAPVIM
TSAWAVTLAVQMKTECWWGYNLSIYFWILEGPRFAVVILNFLFLLNIIRVLVVKLRQSHTN
EIEQVRKAVRAAVVLLPLLGITNLANMLGAPLDRQVWEFAAWSYATHFLTSFQGFFVAAL
YCFLNGEVSIQTI
>DmCG11144
MKQKNNNGTILVVVMVLSWSRVVDLKSPSNTHTQDSVSVSLPGDIILGGLFPVHEKGEGA

PCGPKVYNRGVQRLEAMLYAIDRVNNDPNILPGITIGVHILDTCSRDTYALNQSLQFVRAS
LNNLDTSGYECADGSSPQLRKNASSGPVFGVIGGSYSSVSLQVANLLRLFHIPQVSPASTA
KTLSDKTRFDLFARTVPPDTFQSVALVDILKNFNWSYVSTIHSEGSYGEYGIEALHKEATER
NVCIAVAEKVPSAADDKVFDSIISKLQKKPNARGVVLFTRAEDARRILQAAKRANLSQPFH
WIASDGWGKQQKLLEGLEDIAEGAITVELQSEIIADFDRYMMQLTPETNQRNPWFAEYWE
DTFNCVLTSLSVKPDTSNSANSTDNKIGVKAKTECDDSYRLSEKVGYEQESKTQFVVDAV
YAFAYALHNLHNDRCNTQSDQTTETRKHLQSESVWYRKISTDTKSQACPDMANYDGKEF
YNNYLLNVSFIDLAGSEVKFDRQGDGLARYDILNYQRQENSSGYQYKVIGKWFNGLQLN
SETVVWNKETEQPTSACSLPCEVGMIKKQQGDTCCWICDSCESFEYVYDEFTCKDCGPGL
WPYADKLSCYALDIQYMKWNSLFALIPMAIAIFGIALTSIVIVLFAKNHDTPLVRASGRELS
YTLLFGILVCYCNTFALIAKPTIGSCVLQRFGIGVGFSIIYSALLTKTNRISRIFHSASKSAQR
LKYISPQSQVVITTSLIAIQVLITMIWMVVEPPGTRFYYPDRREVILKCKIQDMSFLFSQLYN
MILITICTIYAIKTRKIPENFNESKFIGFTMYTTCIIWLAFVPIYFGTGNSYEVQTTTLCISISLS
ASVALVCLYSPKVYILVFHPDKNVRKLTMNSTVYRRSAAAVAQGAPTSSGYSRTHAPGTS
ALTGGAVGTNASSSTLPTQNSPHLDEASAQTNVAHKTNGEFLPEVGERVEPICHIVNK

LGRs

>KAF7989315.1 hypothetical protein HCN44_007989 [*Aphidius gifuensis*]

MKHKRITLTGTLMISSVVVLGVLMYYFSQDKCPMGTFLCQNTTNNCRPQRQWCNGNKD
CPDNDDESITNCFDEIHGEWGWFLKKRGGSPGKICDSSKCPTAECICRRCQVSCINSTKITK
IPSNFSPNITRLNMRNNSLIEINADAFVNYLEIRSLYIELNKIEKLKKGIFLKQKNLRWLILSH
NRLVEIETGDLIGLSSIESIWVIKNNLTKCDFTDFENSTTLERMDLSMNALTYDSIYLPYLPA
LKKLSLNENRIEKIDNYLFSQLPSLKCLELQKNNLQIIEENAFINLEQLTELKLEDNNIIIIPD
KLFTSLINLKKLTIGYNPIENLQIIIFEKLKNIRSLGLQEIEMENLDKNSFNQFTELDFVYFKK
FHYCMSYAPNVKKCRPVSDGVSSLSHLLGKPLLRGAVWGISSVTCLGNLLVLWGRFTAKD
ENRVLSIVIRNLAVSDMLMGIYLLIIGLVDIQFRDKYTQEANVWMSSWFCIFLGMLAMTSS
EVSVLILSFMSIERFLLIAAPLRGQQRTLTPITAKFSMTIIWIIGIIIASLPVIHWRSSTKFYGLN
GMCFPLHIDDPFIIGWEYSAFIFLGVNLTGLVIIGYVYVAMFTSIWKTRKNVNCQLSIGDSE
FALRFFFIVLTDASCWAPIIVLKILAFLKYPIAPDLHAWVVIFILPVNSAINPLLYTFTTPKFRE
RFMNEKWIYQIQNYFYKKEESSQDVKDVGNDKMENKFEKCCGLGKSWANEGLKCDKFI
GPVSGLSQVEQGLCLEAVDICCNRVYHEISCKIGKKKAKEGLGCTDETNKKLENDHYQKD
CCEGCKLGILTGSMGQGCAFNGFSFGIPWDPAFMECCHETESPSNTSTSTTINNKKSTTVV
PTESTPTSSSDLDLSLEFTDDDNSTSAENTKSKNICTILKGLLCSDICVPVDGSYYCDCPKG
WVLLEDKKTCRSETPINRCESDNPCEHRCKDNGTAVICDCDPGFILADDGHSCNQKPTETT
TIKITTLKPTKKKIKTTKKIISTTQKLITTTTPKLTTIPPTTIPTTIPTTIPTIIPTTIPTTTVKKLTT
IKNNRNEKLTTRSSSNTDNLPQCPIGYHYNPEAGVCDDINECEDDQEICTFGKCENNIGSYI
CHDNLPDESSMRNVLIQPITDIKNRCPPGYEWDHQAENCLDIDECGVLIDQPCPINTHLCV
NTHGSYSCHELSETTGCPAGFKYNTFDKKCQDVDECKEELHGCLSGVEVCRNTEGAYEC
DISCRKGFEFNRNSNMCIDINECEDNPCPLEKPICVNLPGFHQCRNKRKIKKKENLKATYK
ENEIIDEQKFEESIKKEDTIFIDQNNTLDFNLQSSCSSGYKNNGINCTDIDECTDGPGCQEHE
RCINNPGGYDCLPLCTAGTFFDRISKTCQDVNECLLGQHSCLENEICENTNGSFICKEPPSC
DPGHRLQNNSCIDIDECTDNTHNCQENLHQYCVNRLGSFDCITRLPICEFGFNFSLSTNKC
EDIDECNDTIKSPCDARLGERCVNLNGTFSCERPTINFNNIYQRRGACPSGYRYNSQQRDC
EDVDECAEHLDSCGTEVCYNQPGGYTCASRPSPITPRPTRQETQPPPSSSDSNAQKKCATG
LKYVRNRGCIDINECEEIEEACTSNEECINTSGSFTCKCKLGFRRDNLTQACVDINECQFQQ
NDCLPTQRCDNTLGSYICVRFLSCGTGYTLNAATESCEDDDECVLGTHDCSNGYHCRNT
VGSYRCDKNQRGQVLTTTKKITTSTTSRPLSTTSRPLSTTTKIPIITTTMTTAMTTILPEIDAP
RSCPPGFLRGSSNQCIDIDECQYGRSNPCSRQPMSRCVNTIGSYQCVSRKICEPGFKADLQT
SRCIDIDECEDGTHQCGRGQLCENLPGRYACSCPRGYTIGDNNNCLDINECKIFGRNPCGG
NGKCENTDGSYRCVCEEGFEKLNGDNVCQDIDECRINSNTCQHDCINMWGSYRCTCQHG
YRLNHHDNRTCIDINECEEFKENNLCVGFCDNTMGSYLCRCPDGYRLSSDGRTCQDIDEC
SSGPVCRDLNDMCQNTRGGYKCNKIKCPIGYHRDEYRKNRCVRTSRYCPSGDLSCFRSPS
HYSFNFITFVSMFPIPESTGQLELFTMRGTHLPGSTIQFNKSLVNVRSPPGLPRVTESYFTLR
HPSPSDAVLCLSRSIPGPQEIELELSMEIYHGEAFAGSAVAKLFIYVTQYEF

>KAF7997781.1 hypothetical protein HCN44_009179 [*Aphidius gifuensis*]

MFKTFYGDKTVGIVLTIFIIGFYIIFVESGDNDTVLHICNVTRNEILCRGAGLRSVQMAISNS
SSGKYNNNNVTKIDLSNNNITHIPAHAFHHVPNIEIILLRRNRLAVINGGAFTNLSNLRILEL
DDNHLLEIPEAISNLKNLTDLSISNNKIRGLGIGVFHENVNLVSLDLRGNPIKTIYWEVFINK
SKLRKLIMSDIRGLQDFPNLNGTSSLEVLKLDRASLTDVPEDLCKHAPKLKSLDLKSNYLL
KIPNLIDCKELRLLDVGSNEISSLWGQPFLNQWNLHDLLLSNNNIRIIPQDAFTGLVRLQVL

ELESNDIEYIHPDAFRQIKQLENLNLGNNIFPTLPIKGLSNLLHLKTFNNPALREFPTPDRFP
KVQTMVLSYAYHCCSFLSIELDEPVTKGTVEDSVLFPGDADFDMDFWNSSLTDIWPRLHN
MSNKFGSQINELWDNFGTDFTYPGNLPSYIEDYFEDEEGKTLSPSGQTLPTHVQCLPRPGP
FLPCEDLFDWWTLRCGVWVVFLMAMLGNGTVVFVIIFSRSKMDVPRFLVCNLAAADFF
MGVYLGLLAVVDASTLGEFRKYAIPWQMSIGCQLAGFLGVLSSELSVYTLAVITLERNYAI
THAMHLNKRLSLKHAGYIMTIGWSFALTMATLPLFGISDYRKFAICLPFETNGTSSLTYVV
FLMFINGVAFLILMGCYLKMYCAIRGSQAWNSNDSRIAKRMALLVFTDFICWSPIAFFSLT
ATFGFHLISLEQAKVFAVFVLPLNSCCNPFLYAILTKQFKKDCIMICKAIEESRVTRGIGRCR
HSSNFSNRQTPANTNSLVDRSSRENQPQCACSARLIEANQCTERWWAAKLFWPCGKDKR
QRHNRSDQYAYQIAEIQQKQHKRASSVSSSENFSSSRSDSWRQAHHCGIPLRLLDPKRRAS
SWLITRKPSQDSNLSSSRNDSSGSGTTASTSTWRMSRSSASLEVNTRTTPRPARSKPRLTRQ
FAIQEPEPPASPGRLATRLLATIPSAAEMSEQQDDELPSQAKE

Other insects:

>Dm_CG11144
MKQKNNNGTILVVVMVLSWSRVVDLKSPSNTHTQDSVSVSLPGDIILGGLFPVHEKGEGA
PCGPKVYNRGVQRLEAMLYAIDRVNNDPNILPGITIGVHILDTCSRDTYALNQSLQFVRAS
LNNLDTSGYECADGSSPQLRKNASSGPVFGVIGGSYSSVSLQVANLLRLFHIPQVSPASTA
KTLSDKTRFDLFARTVPPDTFQSVALVDILKNFNWSYVSTIHSEGSYGEYGIEALHKEATER
NVCIAVAEKVPSAADDKVFDSIISKLQKKPNARGVVLFTRAEDARRILQAAKRANLSQPFH
WIASDGWGKQQKLLEGLEDIAEGAITVELQSEIIADFDRYMMQLTPETNQRNPWFAEYWE
DTFNCVLTSLSVKPDTSNSANSTDNKIGVKAKTECDDSYRLSEKVGYEQESKTQFVVDAV
YAFAYALHNLHNDRCNTQSDQTTETRKHLQSESVWYRKISTDTKSQACPDMANYDGKEF
YNNYLLNVSFIDLAGSEVKFDRQGDGLARYDILNYQRQENSSGYQYKVIGKWFNGLQLN
SETVVWNKETEQPTSACSLPCEVGMIKKQQGDTCCWICDSCESFEYVYDEFTCKDCGPGL
WPYADKLSCYALDIQYMKWNSLFALIPMAIAIFGIALTSIVIVLFAKNHDTPLVRASGRELS
YTLLFGILVCYCNTFALIAKPTIGSCVLQRFGIGVGFSIIYSALLTKTNRISRIFHSASKSAQR
LKYISPQSQVVITTSLIAIQVLITMIWMVVEPPGTRFYYPDRREVILKCKIQDMSFLFSQLYN
MILITICTIYAIKTRKIPENFNESKFIGFTMYTTCIIWLAFVPIYFGTGNSYEVQTTTLCISISLS
ASVALVCLYSPKVYILVFHPDKNVRKLTMNSTVYRRSAAAVAQGAPTSSGYSRTHAPGTS
ALTGGAVGTNASSSTLPTQNSPHLDEASAQTNVAHKTNGEFLPEVGERVEPICHIVNK

>Hheb110870.1
MRGRRIVITAVTLITMVIGLAVMMYYFNQDKCPMGTFLCQNSTECKPQRYWCNGRIDCPE
GDDESFTNCFDASGNWEWFFKKRPQPPVLICDPRDCPSPECSCQGCRANCKGFTEHLPNL
SPNITSMTLFNASVKQWRAHNLERYAEIRSLYLASNKIETLEEEAFSKQAKLYWLVLSHNK
IKQIKRGHFKGLSSLETLLADDNRIAKADFSDFAESTSLEMIDLANNSLTEMTLIFPNLPAM
KELILDNNNIKSINRDFLAGMPQLRSLSIERNKLITIDSGVFRNLGELTELNLADNRIRVIEE
HLFDPLVNLTQLMIGYNPIENLPVSSFNELTNLRSLGLEDVDMENFDTNAFVPFAQLEFVY
FKKFHYCTTYAPNVRKCRPASDGVSSLSHLLGKPLLKVAVWSISSVTCLGNALVLWGRFT
AKDENRVLSILIKNLAVSDMLMGVYLLIIGLVDVQFRDTYYQQASSWMSSWSCTLLGILA
MISSEVSVLILSFMSVERFILIAQPLRGQQRALTPQAAAFSMTFIWICGIILAFIPVIHWRSST
RFYGLNGLCFPLHIDDPFLVGWEYSAFIFLGLNLVGLVTIGYVYAGMFTSIWRTRHNTPLF
VGDSEFALRFFFIVLTDAACWAPIIALKIVAFWNYPVPPDLHAWVVIFVLPVNSAVNPLLYT
FTTPKFRERLNEGWMGQLKNYVFDRRATQDSQASAGSSQDVVPNGFLSLAAIGKWAEFD
RKPSTHKQN

>Bm LGR1
MFWRFNVVTIISILLFANQPVCCQLSEGVSVVLHQTNITRLTARALHAAGYHLHIIEHLSIM
GAPKLEHVVVEDLIRMPRLKSFFITQAPLLHRVAPLPALPELRTFMITTSGLLEVPNLSHVH
DSRKANTSLSYLQAIDLEGNHIKTIPSHALRVRADQVSLNYNLIEEVPKHAFKNAQISKLSF
KGNTKLKRLDEHAFAGNLLLRQLDLSNTAITSLPTKGLEKLQILRIERTPSLKYIPSIYEFQQ
LEKAYLTHHFHCCAFKFPEIHNPARHKLYETQMAMMMQRCASIQKSQARKRRSLEPIRPV
TDGAQTVTALEDDDASTMTASEEYENFEEYFSDSGSYEDGDQGEFHDIVNDTVVSISADC
GNFNTRNRNVECTPASDALNPCEDVMGWSWLRASVWVVVAAAVVGNVAVLLVLLTNHT
ELTVPRFLMCNLAFSDLCTGLYLLMLAVVDLRSYGEFFNYAYNWQYGVGCKIAGFLSVFS
GQLSVITLTIVTLERWFAITYAIYLERRISLSTAAKIMLGGWLFSSLMAGLPLLGVSDYSSTS
ICLPVESKDIGVVIYQGSLFLTNALAWVTIVVCYVQIYRSLGGGGENYGGRRAAAAAERRI
ANKMALLIGTDLLCWAPVAFFGVTALAGVPLVDVSHGKVLLVFFYPLNACANPFLYAILTK
QYRRDFITLVARTGQCNWLVEKYKLSTTPPPTAHTNPSTPAQLMPLVDQKNHSQISKEFSD
FKA
>DmCG7665 GPA2\GPB5
MEKHPSLSQRMGTTYRPRKGLKCLSFEFQCRLLLHHLLLTSLSGRHFVYATSAVGGALSA
NNCHDIHHGFDVYPNLTAVSLAQSTDTPLTATMPRSAWKCCCWNASNQAEEVECRCEGD
GLNRVPQTLTLPIQRLTIASAGLPRLRHTGLKVYGSTLLDVAFTDCLQLELIQDGAFANLTL
LRTIYITNAPKLTFLSKDVFLGISDTVDIIRIINSGLTRVPDLGHLPPHNILQMIDLDNNQITRI
DSKSIKVKTAQLILTNNEISYVDDSAFFGSKIAKLSLKENKKLQMMHPNAFDGIIDITELDL
SSTSLVGLPSAGLQNIEALYIQNTHTLKTIPSIYNFRNLQRAYLTHSFHCCAFQFPSRHDPQR
HAQRMLEIEKWRKQCKSDSGTRKERSTLDNPFNMPEDFGSFGGTDDSATDITPITFASFDY
MADDTMNKGTFHEKIILNPGDDSSAELCGNFTFRKPNIECYPMPNDLNPCEDVMGYQWL
RISVWIVVALAVVGNVAVLTVILSIRPESTPVPRFLMCHLAFADLCLGLYLLLVACIDAHSM
GEYFNFAYDWQYGLGCKVAGFLTVFASHLSVFTLTVITIERWLAITQAMYLNHRIKLRPAA
LIMLGGWIYSMLMSSLPLFGISNYSSTSICLPMENRDVYDTIYLIAILGSNGVAFSIIAVCYA
QIYLSLGRETRQAHQNSPGELSVAKKMALLVFTNFACWSPIAFFGLTALAGYPLINVTKSKI
LLVFFYPLNSCADPYLYAILTSQYRQDLFTLLSKLGLCQQSALKYKDSLSGQATTRFTIHGSI
QRHSSLTCKMQTVMGAETQKMLKNSEDYV
>NlA50
MQRRAVATALVTAVTLTHFTVKADQDGVEPHPTWPFAVKADQNGVEPHPTWPPPPQLPPM
VQSHEPGCSCSDHRDPPRLLVKCKCRGDHLQRVPSDLQRGLHILSITRAAIEVLAADSFQP
YRESLTDLSLVRLPNLRLIEPGVFNNLPHLRTIDIHSAPMLTIISDAVFQTHLPRLRIFRCTNT
GLQQIPALRDLESKHQLHLVDLDSNRISLIPERSFYITSDQVSLNYNQIEEIEAFAFHNSTIAT
LSLKGNRNLHILSEDSFSGLNSLRKLDLSETAITFLPTLGLRGLDELRLQGTTSLKVFPSVYS
FDSLKDVYLTYSCHCCAFRFPARHDPAGFRRHKEFVEKMIRDCSSSFSGDNVSYNDRANQ
QNISFGPANTSFWNNIEFTSGSSTTSSEETFHSIVAVSPNGQLQVRCGHMLGGGGSKGRLQ
GEGPRCFPAPDAFSPCEDLLGSGWKVRISAWLVSLFALVGNTCVLLVLLSSRFRMSVPKFL
MCNLALADLCMGLYLLLIAIADARSQGAYFNYAIDWQNGIGCQAAGFLTVFASELSVFTL
SVITSERWYTITYAIHLNKRLRLGSASRIMAAGWLYSIAMAALPLLGVSSYSITSICLPLQTS
SAVETVYLATMLAVNGVAFGVVCVCYGLMYASIRGQGQGRSGRVRSDLSVAKRMALLVL
TDLVCWAPVAFFGLTALAGHPLIDLPSAKLLLVSFYPLNACANPYLYALLTRQYRRDLLAL
FARYGICSKQAATRHRGGLAGGGGHGEARGTGGRGSRAGEGEMVLGHSPHHTGGLYSPP
>NlA49

MTCVLDAVTITRFVRLHWLMVSILESTASSAEATSVVRNWTSQQDVLLPPLIQSTDSGCTC
KNLTGSDFTGCTCRGDDLKHIPDNLAPNLRTLTVTNAAIEVIEADSLISYRSTLADVSLNHL
TRLRVIEQGVFNNLPALRTIVINYAPSLKSIPDAFFRTSLPELRIFRCTYTGLGVVPAMKYLG
SKHPMSIIDLDNNQIEKLTRGSIQVTSDQLWLNYNKIREIEALAFFNSTLATLSLKGNRELK
MLDSDAFTGLRSLRHLDLSDTSITFLPTSGLRGLEELKIQGTKSLKVFPSIYSFDSLKEVWL
TYSCHCCAFHFPAQHDPLGYLRHQEFMLRIKEEQCTSQKNHHTELKKHHGMGAFDQDW
GFSVTKVLPTGSFLEGQFHSIVSTNKKINALCGNISKNYEEVKCFPRPDAFNPCEDLMGNW
VLRVAVWLVAILALLGNLAVLLVLLSSRFRMTVPKFLMCNLALADLCMGLYLLLIAIMDA
RSIGDYFNYAIDWQNGIGCKLAGFLTVFASELSIFTLTVITCERWYTITYAIHLNKRLRLSSA
AQIMVLGWIYSITMATLPFFGISSYSITSICLPMENAKTSDLVYLVTLLVFNSLAFWVICACY
SRMYVSIRGGQGQATADPLSCPDMRVAKRMALLVFTDFACWAPIAFFALTALAGLPLIDVP
KTKILLVFFYPLNSCANPYLYALLTQQYRRDFFILLSR
>NlA48
MMSFQVSSIILHLALIANHAVAKDRIGMYWKCDCNFNNNSQDVPLKTDCHSFGVESKHLP
HNKNATILWSLSNGAVTDIVKKCFLSHNTTLTDISFARLKFVDEFDKQAFTNLRLLESISITQ
SPALRYLPNGVFYPVSKNLRVLRMTHTGLEAVPTLPALSKKIMHLIDFESNKIRSIPSNSINI
QTEQLVLAYNSITKVEGWAFNGSSIGKLSLRGNNRLVELSSDAFRGLKGLLDLDLSQTAITF
LPVVGLEMLEMLKLQGTTTLKIIPSIHDFKSLQVAELTYSFHCCAFKYPARHNPARHELHV
EYLQIAKERCGGNAPKRIANRFSRIKREADGSFGEIQNDGDEYGKFSHSVEDDSQQYPQS
GSVSQENGDRNHSKLGGWTDAFEPPSTKKSESWKDGGVFHQTHASPTSFEAFCGNLTIRT
NPVDCWPKPDALNPCEDIMGYDWLRISVWFVISTALFGNTAVLIVLIANRSDTTVPRFLM
MNLAFSDLLMAVYLLLLAFTDIQSTGMYFNYAFDWQRGYGCRIAGTLTVFSSQLSIYTLTL
ITIERWFAIRHALYANVIDLKVAIQAMIVGWVYSIGIAVLPLFGISSYSTTSICLPMDVHNAA
STIYVLTLLMVTGVAFVIICICYIQVYLSLGKNTRHCPESSVARKMMLLVGTNFACCAPIAF
FSLTAVAGYPLIDVTRSKILLVFFYPINSCANPYLYAIMTAQYRKQLIQLMAKCGFCTECAQ
QYKMVYQPELEQKPRPSRATLLSTAHSTEDTVFNNNDSTRDTCNDISEREHQTEDNV
>Tc TcasGA2_TC009127
MIFCMFMIWLVSAVEGSDMLHKLPEETAFVSKVEPHETKPCTCECYNTTENFQIDVECTCS
GKELQHIPDFLNKTLTKLVITDSDIKRIRKDELKPYRDTLKDVTLGNLPYLRVIEDGTFADIP
NLRTLYISHAPQLKFLDGLLMGVTSKKFYSLRIVQTGLAEVPDLSYLPPENVMHLLDLDL
NKIDKLKANSVKIQAEQVTLNYNEITVVEDLAFNGSQIGKLNLGGNKRLKKLEPNAFKGL
QSLRELDLSSTSIENLPVVGLGEIETLRIEDTPSMKVIPSIYDLENLKVAKLTHPFHCCAFKY
PEQHNPERHAQYEETTKRACKESTVAVDTGQPDGGNKRTKRWFQDDYDPSLNVLGYEHI
GSRPLPPKDWTPNLDHFKGHPNKSDHSHLNPLRGRTIDHEEARPLDYEEDFGTFHAKSAEI
PQQHKVYAVCGNLAMMRTPSVKCYPEANALNPCEDIMGFSWLRISVWFVVVLAVVGNL
AVIVVVLFSGGELTVNRFLMCNLAFADFCMGLYLLLIASMDLHSVGTYFNFAFDWQYGFG
CKLAGFLTVFSCHLSIFTLTIITLERWFAITYAIHLTRRIRLGAAAKTMLGGWMYSILVASLP
LVGVSNYSSTSICLPMEVNRVADRAYLYSIILVNAIAFALIAFCYAQIYLSLGQETRHEMAIA
KKFALLVFTDFATWAPISFFSVTALAGYPLIGVTKSKILLVFFYPINSCANPYLYAIMTAQYR
KDFFILLS
>tetur01g15880
MWILLMLNGFTLSSGFTLTTKKPINSNHDSRHLPRGWFGAIPDDDASNHNNDDNDEVALV
VENVDPSEEAKKDEKDHFFHDNQTPSSVPSDKSNIDGQSSDVSVDFSHSFSPTNPSIDSHFS
TSLPTSSTKQTFSFENSSDDWVTTDLVYNNSDENENEVDQQNKVNEEDGFMIRGSSETQE

EATAFDKHFCACKNETTHSWDHVEISCKCFGETIVDIPNHLTKGVAKLSMISTGIQTLVQN
AFHSYSASLKDLYLESVKKLRWIEPGAFNNLPFLRTVYIKQAPLLKFIHDGVFFGSFPKFQV
LKIIQSGLEVLPSMKYFETKGIISLLDFDSNRLKNISSASIRVRASNMILDYNSIEKVYSYAFL
GSHIAKLSLKGNRRLVTIDDEAFVGLRNLAKLDLSETSITRLPTKGLEEVEVIKIVDTFTLK
VFPSVFNFKNLKEAQLTYPYHCCAFKFPATHDPKEFASRVFEERRNCRQGTSGSTVLSTPSS
TTTVATTSTTLLTSLHSLNLINNYELKSNGDLIGSYWKFIGDGIKHYTRRLNSLLSDNNFIGS
INSQKSIESSSLLFSDNIISEDEQDENEDNNNENNEKDKIDDDNGARDKMRREMKNHLDD
SVRFNSSSIINSNSLPNGEYITTTLSPLMDQSIFGKLIVSPSDAIDQKVNSSSIDILAGALNPQS
PGFGQVQPSIFELPNEQLKHSEGDFIGQFHPTAASVLPEKPIHAFCGEYAQIFRDIKCSPEPD
AFNPCEDVMGNLMLRIADWIISIAAVLGNLAVMVVLMSGRFKMNVSKFLMCNLAFADFC
MGIYLLIIAIIDIHTVGVYFNYAIDWQHGLGCMITGFITVFASELSIITLTVITLERWYAITHAI
HLHRRLKLNLAVKTMIGGWIYSLLMASLPLFGISGYSKTSICLPMENKDTVDIIYLITLLSF
NALAFLLITACYGKMYHAVASQHGRITANDQTIAKRMALLALTDFACWAPIAFFGLTAVAG
YPLINLTNSKILLVFFYPLNSCANPFLYAILTKQYRRDFFILISRYGLCRGSAARYKGTSNNC
KHRRKGSCKKGRFCQVAYDCRTDRHKCKRCRHCLHSNTSSEENSSRSVIYSAIELYPDSSV
SGDSVHEVLKMGHVICDDKQQSCSHHHHHYHHHYHHHKSNKESKKFPNHRPISQTTIIYS
DDESNSSISLKKSPLIIKNGKELRRLPSSERDEVLRKMLGDSTLAPTTAGKNVVPSVWTTS
NSCTSIHKSHKNCTSWIQRDSSSGEKSSAGNNNSSSNGTAIEIIKREKRCYCCKHCKRIPIEN
GSFVLSSHSNSAADDTEI
>PpLGR2
MLPRPRPSLVVGLLLLAAIGLEVVRAEVVLGCDVLGGPAARELVCRAAGIETLDRLPDLA
NVTRIDLTSNNLTNIPARGFQRYPYLEILLLRRNRIESIDVEAFDNMTRLALLELDDNNLTEF
PRALARINSLEELSLSNNRIRVIEADSLQDARNLLSLDLRGNPIREVQPAAFQQLSRLRKLIL
SNLKELSEFPSLEGCRSLEFLRLDRARIERVPDELCRHAPKLKSLDLKSNRLTVVPNLKDC
HDLRVLDLATNLIFSLEGKPFEGLGALHDLLIPNNLLESVPQDAFTGLTKLQVLDLESNSID
FVHPDAFEQIKQLEDLNLGNNIFPTLPIKGLSGLLHLKTFNNPALREFPAPELFPRVRTMVLS
YAYHCCSFVSAEELEASYEADAGLSGGLETAGEEPVQESVLFPTDNDFDMSLWNQSLTDI
WPQLHNLSDKFGSKINELWDNFGTDFTYPGNLPAYVEEYFDDQDGRSTSTPATSAHPHVQ
CLPQPGPFLPCRDLFDWWTLRCGVWVVFLLAMLGNGTVVFVLIFSRSKMDVPRFLVCNL
AAADFFMGLYLGMLAVVDASTLGEFRKYAIPWQMSVGCQLAGFLGVLSSELSVYTLAVIT
LERNYAITHAMHLNKRLSLKHASCIMAAGWSFALGMAALPLLGVSDYRKFAICLPFETTS
PSAMAYVVFLMMINGVAFLILMGCYLKMYCAIRGSQAWNSNDSRIAKRMALLVFTDFLC
WSPIAFFSLTATFGLQLVSLEQAKVFAVFVLPLNSCCNPFLYAILTKQFKKDCVLICKAIEES
RVTRGIGRCRHSSNFSNRLTPANTNSLVDRSAGSRDLHPRPHPHQQHHQINLYQQQQQQ
QQQATCVCSNSARLILERGQSAGSTTTTAAAVRWWQAKIAWPCSKSQRQRRNVAGDPYA
YRIAAEIQQKQHKRASSMSSSENYSSSRSDSWRQNHPCGIPLRLLDPKRRTSSWLITRKPS
QDSNLSSSRNDSSGSGNTASTSTWRMTRSSTSLDRPKPRLTRQMAFQEPDSPGSPGRLAVR
LLATIPSAAEMSEQCDEESAALAEKEEEEEPNADDDDDDGEEVKIRIKDAKES
>NvLGR2
MLLSLVGRLLLLWAAIGLQAVRTETPDCELLGGPAARELSCRAAGIEALDRLPELANLTRID
LTSNNLTNIPARGFRRYPHLEILLLRRNRIERIDPEAFDNMTSLALLELDDNKLTEFPRALAR
MSSLEELSLSNNRIRLIEADSLRDAKNLLSLDLRGNPISEVQPAAFQHLSRLRKLILSNLKEL
SEFPSLEGCRSLEFLRLDRARIERVPDELCRQAPKLKSLDLKSNRLSAVPNLKGCRDLRVLD
LASNLISSLEGKPFEGLGALHDLLIPNNLLESVPQDAFTGLTKLQVLDLESNGIDFVHPDAF

EEIKQLEDLNLGNNIFPSLPIKGLSGLLHLKTFNNPALREFPAPELFPRVRTMVLSYAYHCCS
FVSAEELEAGVSGASEAAAGEELLQESVLFPTDNDFDMSLWNQSLTDIWPQLHNLSEKFG
SKINELWDNFGTDFTYPGNLPAYVEEYFEEQEGRSPRPATQPARIQCLPQPGPFLPCRDLFD
WWTLRCGVWLVFLLAMLGNGSVVFVLVFSRSKMDVPRFLVCNLAAADFFMGLYLGML
AVVDASTLGEFRKYAIPWQMSAGCQLAGFLGVLSSELSVYTLAVITLERNYAITHAMHLN
KRLSLKHAGYIMSAGWTFALGMAALPLLGVSDYRKFAICLPFETSGPAAMAYVVFLVLVN
GLAFLILMGCYLKMYCAIRGSQAWNSNDSRIAKRMALLVLTDLLCWSPIAFFSLTATFGLQ
LVSLEQAKVFAVFVLPLNSCCNPFLYAILTKQFKKDCVLICKAIEESRVTRSIGRCRHSSNFS
NRLSPANTNSLVDRSAGSRDLNLLQHPHQPQVNAYQQQQATCVCGNSARLILERGQSASS
TTTAAVRWWQANISWPCSKSQRQRRNLAGDPYAYRIAAEIQQKQHKRASSMSSSENYSSS
RSDSWRQHHRCGIPLRLLDPKRRTSSWLISRKPSQDSNLSSSRNDSSGSGNTASTSTWRVT
RSSSSLDRAKPRLTRQMAFQEPDSPGSPGRLAVRLLATIPSAAEMSEQCDEESSALAEKEEE
QPDADDDEDDGEAVKIRIKDSDES
>BmLGR2
MQSAYSSSVGAPGAGPSLRRGTETILRRDRCHSKTTNHRPKASPLDEEVTQALKNQNSHL
LGDLAADQTRLAVKYRRRTRNPHIGHVILQVSPGVWQRLTAAGRVHVDLQRIRVFDQSPL
VQCSICLGYGHGRKHCNDTVPLCSHCGDPHLRADCPLSLAGTAPHCRNCRETKNSNAEH
NAFSTECPVNLQRSKLATDELMLEAQKRKVLGTLLQEPYVGSVKKMKSYRGTRIFQNSA
VGEGTVKAAIVIYQPELDIIQYPQLTTNNIVVVGVRTRAWNITLVSYYFEPDQNMGPYLEH
LKRIELETGQSRLIIGGDCNAKSAWWGSPNEDQRGEQMCGFLEELGLQVLNTGDIPTFDTI
RGGKRYSSHVDVTACSADILDLVDGWRVEEGLTGSDHNGITFGINVIKSKGISKYNLSSNS
LRHSCLRRKMFGIRTPVKKSNGSEEKSELKELPCLSVRRSISDWPPTAEKEQQEQQRPKIPS
PPKAVPQPLPPRPKTKASIAQEAKADLGRQATRVSDSPPNIVPSRFPSKTAEAKACLMKIRK
QLNVVQRGFAQKLCRAYRTVSLNSALVLAGILPLDLRIREVATLYKAKRGVPQPVLGDRE
VERMAPMIKAPHPAEQVNLEFKSLIDEEQYEHHNNFEVRIFTDGSKLEGKVGAALSLWDR
VSETKALKLTLPPFCTVYQAELLALQRAVREALNHSGTAFGIFSDSMSALQTVTNVSSPHP
LAVETRDTIRRCMLQNKSVSLFWIKAHVGLEGNERADQLAKEAALRSKRKPDYDLCPISF
VRRQIRLETLDEWDRRYRRFLRTFQANSAPSHVMYLLAAMKSPAPPRCVPGSPLHRVRDG
VRVRRRMWAAALWAAGALWAAAAMCSGGGGGERLDCRSAGLQALPPLHHNLISLDVSN
NNISSLPRDALLPATGLRDLNLSSNRLELVSAGSLSGAALARLWLDRCSLRRLPAHALRDL
RRLHYLSAEDNLISELEGGAVGARGLRTLRLSRNLLRAVPTHALAPLHHLQTLSLSGNLIT
ELSDSSLPPLPALHTLVLKRNRITHIDRPAYSGAPSLSRLRLEENLLSELPPAIQLLPVLQDLL
LSGNRIEVVEAGILQQCRLLKRLDLRGNPLTRLHRHALQHLPHLRTLILSEARGLREVPSL
NGSAQVRTLRVERARLTRLSTDLCRHAPLLQSLEMKSNYIDRVPDLHECSELHLLDLSSNE
ISAVQGSSFRGLHKLLDLLLARNRLRHIPSDTFIHTPELQRLNLEENQIEHIDMEAFVSISKL
EDLNVGNNIFPWLPASGLQRLLHLKAHNNPNLRHFHPPDVFPRIQTLVLSYAYHCCEFMPL
MEGGTVTEEETSEEDASTDLVIIPSQSIDAEAWLNATDVWSQLNVSAAGGSRWQAMLED
WESDLVEGVSEHRVEPSRRVQCLPLPGPFLRCVDLFDWWTLRCGVWAVFLLALLGNGTV
VFVLICSRSRIDVPRFLVTNLAAADFFMGIYLGFLAVVDAGTLGEFRAHAIAWQMSGGCR
LAGFLGVLSSELSVYTLAVITLERNYAITHAMHLNKRLSLRHAAFVMAAGWGFSLTAASL
PLLGVSDYRKFAVCLPFETSSPVALGYVVSLLAINGVAFLVLLGCYLKMYCAIRGSQAWNS
NDSRIAKRMALLVFTDFLCWSPIAFFALTAAFGLQLVSLEEAKVFTVFVLPLNSCCNPFLYA
ILTKQFKKDCAIVCKAIEESRVTRGIGRCRHSSNFSNRQTPANTNSLAERSSRGQHHAACA
CRRMLPAGAPPPPPPAARRARLLRWLRACGGIESIIPWHYGNITIRPATVCAGGSAPPPIPSL

P
>Dm_CG8930 Bursicon
MAARCRWSWRLALCPLLLQLLLQLLLLPPSAMGHDETKENPAPDMQNSQEQEPYVHLQ
HLQQQQQQNPQTVQQLSQITVNRTSKSASVTPTGIRENVMLPSADPEKEAQILYEKSLQEY
HGSQLSTASTATDVIAGKRTLHSICERWLQKHCHCTGSLEVLRLSCRGIGILAVPVNLPNEV
VVLDLGNNNLTKLEANSFFMAPNLEELTLSDNSIINMDPNAFYGLAKLKRLSLQNCGLKS
LPPQSFQGLAQLTSLQLNGNALVSLDGDCLGHLQKLRTLRLEGNLFYRIPTNALAGLRTLE
ALNLGSNLLTIINDEDFPRMPNLIVLLLKRNQIMKISAGALKNLTALKVLELDDNLISSLPE
GLSKLSQLQELSITSNRLRWINDTELPRSMQMLDMRANPLSTISPGAFRGMSKLRKLILSD
VRTLRSFPELEACHALEILKLDRAGIQEVPANLCRQTPRLKSLELKTNSLKRIPNLSSCRDL
RLLDLSSNQIEKIQGKPFNGLKQLNDLLLSYNRIKALPQDAFQGIPKLQLLDLEGNEISYIH
KEAFSGFTALEDLNLGNNIFPELPESGLRALLHLKTFNNPKLREFPPPDTFPRIQTLILSYAY
HCCAFLPLVAMSSQKKTSQVQEAVLFPSDAEFDMTLWNNSMMNIWPQMHNLSKQLGAS
MHDPWETAINFNEEQLQTQTGGQIATSYMEEYFEEHDVSGPATGYGFGTGLFSGMSTEDF
QPGSVQCLPMPGPFLPCADLFDWWTLRCGVWVVFLLSLLGNGTVVFVLLCSRSKMDVP
RFLVCNLAAADFFMGIYLGILAIVDAATLGEFRMFAIPWQMSVLCQLSGFLAVLSSELSVY
TLAVITLERNYAITHAIHLNKRLSLKQAGYIMSVGWVFALIMALMPLVGVSDYRKFAVCLP
FETTTGPASLTYVISLMFINGCAFLTLMGCYLKMYWAIRGSQAWNTNDSRIAKRMALLVF
TDFLCWSPIAFFSITAIFGLQLISLEQAKIFTVFVLPLNSCCNPFLYAIMTKQFKKDCVTLCK
HFEESRVVGGGGPGGRGAVARTKRGDLPPPLLPAAAVAHPPGCRCLRMLPSEMPNWHKM
EQTPSMWQRLRTFCCGENRRRRKQRRQPQQRRQRAYTAAAANPYQYQFAELRQQRQNR
ASSISSENFCSSRSSSWRHGPPSSAPVPPGNCSMPLKMLEPHAHPHGHGRRRHSAWLITRK
TSQDSNLSSSRNDSSASATTASTSTFRLSRSSAGSSTPLPSIIAHNGKAQLDAVKPRLVRQEA
VQEEEDSSPPRLGVRFLPTIPSAADSSVVMEDGDSANTGVASFLGMPLPGASSGFLIAPTTA
ATTSPPPVVLQPAKPPPDPNDAPL
>Tc Bursicon
DLTSNNITALNETSLSHYTHMEELTLSENKLESIHPKSFAKNVQLKRLLLQGCALSEIPVEV
LRPLSKLQTLHLGNNEIWKLDGTTFQQVPALRSLRLDGNRLRGVPSEALSSLLHLEVLNIG
NNLINALPPAAFPSLDKLVVLLMKRNQISEIAEEAFANLTSLKVLELDDNFLTEIPAAVTKLA
KLQELSISGNRIKYIRGGLLQKTPALALLELKGNPLTGVDAHAFSFLPRLRKLQVLYVTSCA
YISLRINTRTLSAFTTRNSISKSVLDDFMGVATLRKLILSEARELSTFPNLNGTTALEILRLDR
AGISSVPSTLCTTCPRLKSLDLKSNKLKTIPDLNDCREMRVLDLASNHIRTLENRPFRGMY
QMHDLLLAHNEIQYIPQDAFYNLSRLQVLNLEDNQISFIHPDAFLPISKIEDLNLGQNVFPH
LPSAGLERLLHLKTFNNPNLREFPPPEEFPRIQTLVLAYAYHCCAFLPLIPSNPPPKAKDFIVF
PDIEDIDMNMWNSSLNDYWPSQQNLSHKFGKKFETIWENLRSDFTYPGNFPSYMEEYAEE
EAQRVAGGDGPPGKIQCLPLPGPFLPCQDLFDWWTLRCGVWIVFLCAMLGNGTVVFVLIF
SRGKMDVPRFLVCNLAAADFFMGIYLGFLAVVDASTLGEFRMYAIPWQMSAGCQLSGFL
GVLSSELSVYTLAVITLERNYAITHAMHLNKRLSLKHAGYIMICGWSFAIVMGLLPLFSVS
DYRKFAVCLPFETKDAASLTYVVFLMFINGVAFLILMGCYLKMYCAIRGSQAWNSNDSRI
AKRMALLVFTDFLCWSPIAFFSLTAAFGLQLISLEQAKVFTVFVLPLNSCCNPFLYAILTKQF
KKDCVMICKAIEESRVTRGIGRCRHSSNFSNRQTPANTNSLADRSSRENQNHVPTCTCNV
KLLGERSAPPPRTDRKTRTREWLLSKARWLLCVRRQPRHRPRSDQYTYQIAEIQQKQHKR
ASSVSSSENFSSSRSDSWRHNHHCGIPLRLLDPKRRASSWLVTRKTSQDSNLSSSRNDSSG
SATTASTSTWRMSRSSASSEPNRIRAKPRLTRQSAIQDEADLPGSPGRLTVRFLTTIPSA

>NlA46
MVGVWWWIAVVLWGLIVGGGLGGAGGGHPPACVVQGGDALCRGAALTKIPTNLPQAIV
KLDLTSNNITELGDNAFVELPQLEELILGDNKISKIHPRAFSANPRLKRLSLQNCGLAEVPW
ETFQPLRQLTSLQLDGNEISQVEAVSFAALGNSLHSLRLEGNRLTAPPTAALATLPHLEALN
LGSNLISSIPVNSFPDLGNLIILLMKRNQISTIDEDAFSNLTALRVLELDDNLLTHIPIALTKLT
SLQELSVSGNRIKFVAGGILQRAPSLSLLELKGNPLVGVDPLAFSALPKLRKLILSEARELR
EVPCLNGTTGLEILRLDRASITSIPSSLCHTCPRLKSLDFKSNKLTRVPDLGGCKDLRVLELS
GNQINSLAGRPFVGLHQLHDLLLAHNVIPYLPPDAFTGLTRLQVLDLESNQISEIHPEAFLT
FTQLEDLNLGNNAFSELPTAGLERLLHLKTFNNGHLRDFPPPSAFPRVQSLVLSYAYHCCA
FLGPPGGGQAPLQVGGHAPGANRGTPLHESVLFPTDNEFDMTLWNSSLTDIWPQLQNLSK
KFGTQINELWDNFGSDFTYPGNLPAYVEEYFEEQERTGGGGAVAEGGYGGAPGRIHCLPA
PGPFLPCVDLFDWWTLRCGVWVVFLLAMLGNGTVVFVLVFSRSKIDVPRFLVCNLAAAD
FFMGVYLGMLAVVDASTLGEFRKWAIPWQMSAGCQLAGFLGVLSSELSVYTLAVITLER
NYAITHAMHLNKRLSLKHAGYIMLCGWAFAVVLATLPLMGVSDYRKFATCLPFETTTGP
WALAYVVFLMFVNGVAFLILTGCYLKMYCAIRGSQAWNSNDSRIAKRMALLVFTDLLCW
APIAFFSTTAAFGLQLVTLEQAKVFTVFVLPLNSCCNPFLYAILTKQFKKDCVLICKAIEESR
VTRGIGRCRHSSNFSNRQTPANTNSLVDRSSRDGGQPCSCSAKLLSEAAAAASSPANARG
ANRQPLTTLQRLLRCLRRSTGSGRHRTRSDQYAYQIAEIQQKQHKRASSVSSSENFSSSRSD
SWRQNHNHHHCGIPMRLLDPKRRASSWIVTRKTSQDSNLSSSRNDSSGSATTASTTLSRVS
RSSASSGMSELTGISSRGGTKPRLTRQAAVTEVPPAAPPHYEGGPDPNRGLTVRFLATIPSE
GGNTTTNDEASDSSTAILHDNGN
>tetur11g03260
MKQLFCQEQHQQPTRQPIDINGVTLLIFCIILSSVIIPIGCNTHQLKSGLSSIVTPKCPIECSCN
GVYVDCSNRRLINFPGNISSATKKLNLSFNKLTSWINGTFRSYPDLTHLFLTSNMIEEIVIGM
FDGLESLILLDLSNNNINQIDPDSLKPLVKLKELDLSSNALAEVPVAIRKLPLLKEIMINKNR
IEVIDEDSFNSNNYLQLIEIKGNPLKSLDYSSFLNCTRLRKIVISDGHGLSRWPNLSNANSLQ
HISFDRTNIQTVSPLLCISSAALKSLIVKSSRLRYLPALNNCAKLELLDFSYNQLTMIGEKHFI
TQSQLTHLFLSHNWIQEIHGNSFTGLENLKMLDLSNNLIKSIHPNSFLPLLNLKDLNLGAN
KFSSLPTIGLRGVQQIKVHNNPYLISFPGPEFFPSIQVLALSYAYHCCSYNNLIDEASSSSSTN
RINHFEEDIIWLHRDDVNMSIWNTSWPNIWSTRINSSSSPIWSPETDSFLMNISQFSEEYLD
DYKTEINYDNIVFKYPIKCLPQPSPFTPCEDLFDWWTLRCGVWIVFLLALLGNGVVVVVLI
FGRSKLDVPRFLVCNLAMADFLMGIYLGMLAVVDAATLGQYKVYALPWQRSFGCQIAGF
LGVLSTELSVYTLAVITSERNYAITHAMHLNKRLSLKHAAYIMAVGWTFALIMALLPLFGV
SDYRKFAVCLPFEITDIWSRTYVISLIVVNGLAFFILMGCYLRMYCAIRGSQAWNSNDSRIA
KRMALLVFTDFLCWAPIAFFTLTAIAGYELITLAEAKVFTVFVLPLNSCANPFLYAIFTKQFK
KDCILLCKRIEESRVTRGIGRGRNSSNFSNRHTPVNTNSVADKKSSGDSNQLVCKCGLAST
AQSTVSINKLLPNGSCNGQPLFRKMATKWWQGRSVTKDLVGKLDATDSGSVQRRSNAPN
NRLDSISSGNYSSRSDSWRHSYISPQLLTTKRSAAMVRRRSSWTASSSTSTRKLSTTRSSVS
SDSSSAVFRHDLKGPARLPIADDYLLTFNSKQRSNSVVSSLVNFKPSSAKATFLCPECSRIGT
MKQNEASTSINVNNLTTKYGRETEKIMMAAVVASSRDSNKFFNKLTELSRKDKKDSKEKH
FVLSSGESIKNETTETSLQDSIATDTQMLSSRSNSISSPLTEIQIECTDDDDFNDDYDDLFSPE
RDYDNSYTLYPEPESSRASSNLSIRTQIPKGGTASPIGVGVSSDAELHVDESNNLSGSRELT
GHENGSDQTADNNHEPMGSNLTQSRKVSSDNSLKSPFQVIPSLFKSFSSVGSQIFRSNKNA
NENQNFKENRKAGKSEGKQSQPAQRYLLSPDVPDCSIRVSKSCQTMVTSENFESTLRKSC

TMAQLADKNWNDQIDTLTSPKLKNVSTNMDKDDTRDLHLDEDVPLIFFTHRDA
>DmCG34411
MCIAHLPITFTLAILLAIASNEGAQGVESATRTAIEAIRTGIGTKPETEIADATEAEAPVREVI
SLLGIIDGAESDILVPDADDKCPGGYFHCNTTAQCVPQRANCDGSVDCDDASDEVNCVNE
VDAKYWDHLYRKQPFGRHDNLRIGECLWPNENFSCPCRGDEILCRFQQLTDIPERLPQHD
LATLDLTGNNFETIHETFFSELPDVDSLVLKFCSIREIASHAFDRLADNPLRTLYMDDNKLP
HLPEHFFPEGNQLSILILARNHLHHLKRSDFLNLQKLQELDLRGNRIGNFEAEVFARLPNLE
VLYLNENHLKRLDPDRFPRTLLNLHTLSLAYNQIEDIAANTFPFPRLRYLFLAGNRLSHIRD
ETFCNLSNLQGLHLNENRIEGFDLEAFACLKNLSSLLLTGNRFQTLDSRVLKNLTSLDYIYF
SWFHLCSAAMNVRVCDPHGDGISSKLHLLDNQILRGSVWVMASIAVVGNLLVLLGRYFY
KSRSNVEHSLYLRHLAASDFLMGIYLTLIACADISFRGEYIKYEETWRHSGVCAFAGFLSTF
SCQSSTLLLTLVTWDRLMSVTRPLKPRDTEKVRIVLRLLLLWGISFGLAAAPLLPNPYFGS
HFYGNNGVCLSLHIHDPYAKGWEYSALLFILVNTLSLIFILFSYIRMLQAIRDSGGGMRSTH
SGRENVVATRFAIIVTTDCACWLPIIVVKLAALSGCEISPDLYAWLAVLVLPVNSALNPVLY
TLTTAAFKQQLRRYCHTLPSCSLVNNETRSQTQTAYESGLSVSLAHLGGGVGGGSGRKRM
SHRQMSYL
>DmCG31096 LGR3
MVYGRSIAVGFCLMTVVLLLAAVIFYLSLGPCPAASFACDNGTLCVPRRQMCDSRNDCAD
SSDENPVECGLLYGSKEIADKIVRNAIEKKQQRLISAVSNASGADSTTSMVPRNQSLTLNM
TCDIVTYPKACQCGQGTILYCGRYAKLRRFPRLSSEVTNLIIIRNNLTLRDNIFANFTRLQKL
TLKYNNISRVPLGSFSGLFHLERLELSHNNVSHLPHGVFLGLHSLQWLFLVNNHLHHLPVE
QLRFFRRLEWLVLSRNRLTLRNVQLPKIPTLYEVYLDFNRIEYIGEETFSQLDNLHLLDLQH
NLITHIHGRAFANLTNMRDIRLVGNPIKELSGETFLHNTRLEALSLALMPIHISSSLMEPLNI
SFLNLTGIRYDHIDFEAINSMRNLTYIIYDRFFYCSMTPRVRMCKPSTDGVSSFQDLLSKPV
LRYSAWVMATLTIAGNVLVLWGRFIYRDENVAVTMVIRNLALADMLMGFYLVTIGVQDY
RYRNEYYKVVLDWITSWQCTLIGTLAVSSSEVSMLILAFMSLERFLLIADPFRGHRSIGNR
VMWLALICIWITGVGLAVAPVLLWRTSTLPYYGSYSGTCFPLHIHEAFPMGWLYSAFVFLG
VNLLLLVMIAMLYTALLISIWRTRSATPLTLLDCEFAVRFFFIVLTDFLCWVPIIVMKIWVFF
NYNISDDIYAWLVVFVLPLNSAVNPLLYTFTTPKYRNQIFLRGWKKITSRKRAEAGNGNVA
TTTTGTATGSSQHPDDFTIFAKAAMRCH
>Tc TcasGA2_TC015772
LEQNKICPILGYFQCENTTICIPQQNNCDGKVDCPGGSDEVIGCDDRAKDDYWDHQFKKR
PSALNDHLAHICNLSYNGSCVCRGRDLLCGHKNMVKIPGDLPADNITLLDFEGNNFGVLS
GNVLEKVPLFVEKINFAHCNIDELKPHTFHQLTHIHDLHLDNNDLKTFPSDLFPESNNLRLL
SVTHNHISRISSDAFRKLRALEELDLSGNKISEIKREVLAPLAKLRLLILRNNQIRYISNSTFP
QLPLRKLSLIENRIDRLDPGAFANLTQLQELYLTNNRLVHLKNGTFFNLSQLLVLFLGDNFI
KTIEVGVFIDVLNLTSLTLERNQFRTLDKKVLAPLTILQHIYFDRFELCESALHVRDCEPKG
DGISSQYHLLDSIVLRTSVWIIAAIGCTGNLIVLLGRLLAPTNNVVHSLYLRNLALSDLLMG
VYLFAIAIADQHYRGDYLRYQYSWRHSYVCNICGFLSTLSCESSVLILTLVTWDRFVSVTQ
PLARKQPSPKTAAFTLVVLWSIAAAVALAPLSEGYFGDEFYGNNGVCLPLHIHEPYAKGW
EYSAAMFMLVNALALTFICYAYMRMINEIIASGVACRSTRQSQERDKVAQRFGIIVFTDCLC
WVPIIVVKLVALAGYPIPKDLYAWLAIFILPINSALNPVLYTLTTTVFKKQ
>tetur15g00700
MTLNQVCTVISLIAIFATVFLLPLILLFGEDVANIERSYNEPHLDLFSDSDDNSSNFSINISTL

DSDEDPETPICANIYDFPCHRSFDQDNQVICLNRDYQCNKVKDCPNGEDEEECNDIYGAL
DEHFTRQAKKWGTLIVSNKSQSNFIHANCTLADIYQSRCPCILNDSQRILCTNHDFTEIPSN
LSTTITVLNLDNGKIDSIDSQVIAKYFNLTTVSLKNNRLTTIKSDSFSSLIQLKRLYLIGNEIN
HIDQDAFQKLINLKFLELHTNRLENIDAAQLFKGLTKLETLYLEFNQLKSVGKFPHLDSDL
SNNQLTEIQDIFRNLVSLKVLNLSSNKLKRLPADAFHYNKRLIHLEVALNSIESIDVTAFHNL
RDLRKLNLSYNPLTTLPRHVFKESNALQSLDLSGIEIRNIHPGHFNRTVALRHVYFSKFRYC
MYAPRVRVCRPFSDGVSSVKHLLLYPILRMSVWIVAGITCAGNTLVLVWRSMAKKEHQSL
SLLVKNLAIADLMMGIYLVVIGSYDQVFREKYNNYALQWMHSWKCNFCGFLATLSSELSI
FIVLMITIERYRSITMTCRLVTLRWTASILLLIWTVSLAVSLFPILYWSDPEDPLYYASNGVCF
PLHLEDPFMFGWQFSTFIFLGINLPAIVAIIGLYTRLFLVIKRDRQLTRPALLGKADHEDVILA
FRFFCIVVTDCLCWVPIVSIKIFSFTYMYISPKVYAWLVVFILPINSALNPMIYTIAAPTGFRR
KATKYLRQARRHLGPLLGWTSSGSMHQDRYSSSVPSNGTSSLDHTRSSNCSVLSMAAVNS
ITKNGLITNHVSSEKEIVKMRIK
>AmLGR
MRYKYIGTIGAFLIATTCLLSGLMYYFSQDTCPMGTFLCQNTTICLPQRNWCDQEIQCPYG
DDEQNCCRFIVFIFHPSFSLPKRNPLQKNNDLDILLFAHDYHGVLDWFGSDRDSKAVKEFV
CDSTDVPVSCKYVMCRATCQGYSDIPRNLSSQTTSITLYDSSIERVSAGAFAQYSEIRILYLD
GSNIRELEKGAFANLTKLFWLALDNNEIGEFLPGHFTGLIKLESLKANKNRLTMADFSDLK
GSVYLKLINLNENQLTSKGLRLSELPELSEISMQRNKIKGIEEDTFQNLEQLVELNLAFNEIT
TLPLNVFQPLKNLTSLQLGYNHLHNLPITVLSPLTRLRSLDLEGINLDSLEKDTFNVFEMLD
FIYFKKFHYCATYTPNVKRCRPTSDGVSSLSHLLDKTLLRAAVWIISGVTCVGNVLVLWGR
FTAKDENRVLTIIIRNLAVSDMLMGLYLFIVALTDIIFRDNYYQTASTWMSSWFCTFLGILA
MTSLEVSVLILSFMSMERYVLIAAPLKGHRTMTPQTASTSVIIIWIIGITLALAPVIIWRSSTR
FYGVNGMCFPLHIDDPFLIGWEYSAFIFLGINLMGLITIGFAYFGMFLSIWKTRHACSLSVG
DSEFALRFFLIVLTDAACWAPIIVLKIRALLKYPIPADLHAWVVIFILPVNSAVNPLLYTFTTP
KFREKLHDEWLRKMHNCVTRKSSQGISL
>Nl A47
MSKVMLMVICTCILHLRDAGVIGSSAVASKVVTQETDTQMVPFSCNTTVEAFQCDAATCI
PTTSACNGIMECPNGQDESVSVCGCLPNEFRCRNSCVDLVKRCNRQKDCEEGEDEEDCKT
FLCPITHFKCSNHYCIPLDSVCDFKDDCGDNSDELKCVRRKCWNPEFKCDSGECIRPGFLC
DGSPDCKDGSDESPDTCHPRKFVKCGDGTRIHKYYWCDGWPDCTDNHADELNCEECLG
EDDFKCPNGRCIRKANVCDSQCDCVNSFNNSLTYYYDKVCADEVNCTHSYKLHQGTGA
LECELGSTLGCSTPGSSHTKSRCIAPQFLCDGVNDCHNGDFLSDEFGCPYNQKHQKLDLD
EVFQCRDNRSLPKRFRCDFNIDCLNGDDEEDCPEIEICHENEYRCENGQCIPKSGYCNLVF
DCYDRSDESDCYNNLCGEGLLRCATGQCLPEDQWCDFFIDCLDASDETNCTIRECQSDEF
KCDNGQCVSSTQKCYTSGNPRTGCADRSHLLNCRDWSCPESQFKCHQGPCLNMSLICDG
NIDCPGSWADEDNCTFSCSNSAPQCECQDVMINCTGKGLSQLPDAEKEITSFHLGNNDLG
PTLNNETFLNLDRLLYLDLSNNSITHLMPLMFCNLWRLSVLNLQNNKISILGNGSFYGLFG
VSGLYLQGNQIQKIQTMAFIGLSSLTFLDLHGQRINQIEPSAFVGLRNLAGLDLDLSHNEIK
YLEEGILQGMSRLLSLDLRYNKIKVIATQVFLNTPNLQKLITDEFRFCCLARHVEHCEPPPD
EFSSCEDLMSNIVLRICVWVLAIVATLGNVLVIMWRSRYKHCNQVHSFLITNLAVGDLLM
GSYLLLIALVDWHYRGVYFIHDSDWRSSQLCAFAGCISTFSSELSVFTLTVITLDRFLGIIFP
FRVRRLEMARTRQLMAFGWLLAGILSALPLSRFTYFHNFYGRSGVCLALHITPDKPSGWE
YSVLVFLVLNLLSFGFIAVGYLWMFLVARTTQKAVTKERQMNEAAMAWRMTMLVATDAA

CWVPIILLGIVSLAGYTVPPQVFAWVAVFVLPLNAAINPVLYTLSTAPFLTPARRGLLRFRRS
CKMSLSADPRRTYSSGVATPMPIRRYSSACSNADLYAVTKRASAKWTRVDTTVSERGEVFP
LSRLDNK
>LsGRL101
MATMSGTTIVCLIYLTTMLGNSQGVNLKIESPSPPTLCSVEGTFHCDDGMLQCVLMGSKC
DGVSDCENGMDESVETCGCLQSEFQCNHTTCIDKILRCDRNDDCSNGLDERECDIYICPL
GTHVKWHNHFCVPRDKQCDFLDDCGDNSDEKICERRECVATEFKCNNSQCVAFGNLCDG
LVDCVDGSDEDQVACDSDKYFQCAEGSLIKKEFVCDGWVDCKLTFADELNCKLCDEDDF
RCSDTRCIQKSNVCDGYCDCKTCDDEEVCANNTYGCPMDTKYMCRSIYGEPRCIDKDNV
CNMINDCRDGNVGTDEYYCSNDSECKNFQAAMGFFYCPEERCLAKHLYCDLHPDCINGE
DEQSCLAPPKCSQDEFQCHHGKCIPISKRCDSVHDCVDWSDEMNCENHQCAANMKSCLS
GHCIEEHKWCNFHRECPDGSDEKDCDPRPVCEANQFRCKNGQCIDPLQVCVKGDKYDG
CADQSHLINCSQHICLEGQFRCRKSFCINQTKVCDGTVDCLQGMWDENNCRYWCPHGQ
AICQCEGVTMDCTGQKLKEMPVQQMEEDLSKLMIGDNLLNLTSTTFSATYYDKVTYLDL
SRNHLTEIPIYSFQNMWKLTHLNLADNNITSLKNGSLLGLSNLKQLHINGNKIETIEEDTFSS
MIHLTVLDLSNQRLTHVYKNMFKGLKQITVLNISRNQINSIDNGAFNNLANVRLIDLSGNV
IKDIGQKVFMGLPRLVELKTDSYRFCCLAPEGVKCSPKQDEFSSCEDLMSNHVLRVSIWV
LGVIALVGNFVVIFWRVRDFRGGKVHSFLITNLAIGDFLMGVYLLIIATADTYYRGVYISH
DENWKQSGLCQFAGFVSTFSSELSVLTLSTITLDRLICILFPLRRTRLGLRQAIIVMSCIWVL
VFLLAVLPLLGFSYFENFYGRSGVCLALHVTPDRRPGWEYSVGVFILLNLLSFVLIASSYL
WMFSVAKKTRSAVRTAESKNDNAMARRMTLIVMTDFCCWVPIIVLGFVSLAGARADDQV
YAWIAVFVLPLNSATNPVIYTLSTAPFLGNVRKRANRFRKSFIHSFTGDTKHSYVDDGTTHS
YCEKKSPYRQLELKRLRSLNSSPPMYYNTELHSDS
>PcGPRgph
MFFFFFFFFLETFQCSKSQFKCGNGFCISREHLCNFEDDCGDLTDETDCKYRECWPSEFRC
DNQECIRPGMVCDNVPDCRDSSDETGCLSETCDDGKKIHKNTKCNGWPDCDDHKDELN
CKLNLRNCDLSSGNYFQCPNTRCLRKSRICDGICDCLGETPCFDEINCDDYYTIVNGYQM
CRVGSTISCRTTISGVTIERCISHEYICDGFNDCLNGNNNLSDEYGCVSGVDVWKNILEPNG
EKFIFCGGLDQRKLSHSFICNYQIDCLNGEDELDCVWPNCTENEFQCDNGYCIPIRDRCNA
QIDCSDKSDEINCTGFKCIVDIRDLDEKSNKLKQCDNGQCINSNFWCDFIQDCSDGSDENN
CGNQKEFCTGNEFKCETSGQCIPKNEYCYKNENLRQGCADKSHLKGCANFTCPEGTFKC
KMGPCLHTSLLCDGKFDCLDFWMDEKNCTHECFEFCTCIDITANCTGLGLQTIKNIDKME
DPYRKFYFANNNLSKVLNENFTYNIRFAILLDLSNNSICSITPGIFKDLRDLKTFVLQNNCIT
VLESQTFDGLSNLNGLHLEGNKIQTIKEKAFYGLSSLPTLNLKHQLIKHIAEGGFIGLRKLS
NLDLSQNKIEILYHGTFQGLQKLTHLDLRGNPLRKLESDVFKRPELSIQVLLFDNFKFCCLA
KHVPNCQPTSDEFSVCEDLMGNFVLRVCIWVLGIIASFGNLLVIGWRMNYKHKNKVHSFL
ITNLAVGDFLMGFYLLIIASVDAHYRGVYSVHDEEWRSSKLCSLAGFLSTLSSELSVFTLIL
ITFESFLVIMFPFKVTRLQMSEIRWVMLGVWIAAVCLSGLPLLYKDYFKNFYGRTAVCLAL
CITIDKNSGWKYSAFIFIFLNFISLILIAMGYIWMYGAAKNTRLAVSRNLESKRMEQVMAR
RMIFIVATDAACWVPVILLGILSLNGVSVPSQVFAGIAVFVLPLNAAVNPIFYTISTATFLNPA
KKNIKRFNNSFRFSPSGCVKNSTTSSIITHP
>ApGRL101
MNTENLQFIENMTLGCLQNEYRCTNKCIELVKRCDKIADCDGGEDEKDCTCDFKDDCGD
GSDELQCKHRECWHGEFKCKNSECIRPGYLCDGEVNCADGSDEEYCETSDFIKCGGSHSV

HSTFWCDGWPECADNHADELLCNASCAGNKFQCPNGRCINDANVCDGLCDCLPSIDGN
CADELNCTKFYNKTDDVIVCTTGSTLSCWMPGGNPSRCIRQKYICDGQNDCFNGFSISDE
FGCDKPNSHLNDEFFRCHDGRWLPFKHRCNYKAECLDGDDETDCEVSLPCGEEQFRCAS
GECVKSENRCDGRTDCWDKSDEIGCASVPCPGENWSRCKIGKQCVPMEKWCDYRVDCM
DGSDEKNCDYRLCKADEFRCDSGQCIPLEYKCKKYREEQMGCVDKSHLRNCVDSKCAE
NEFKCHRGPCIHQSMVCDGQLDCDLTWDDEDNNCYFMCSDIASGCQCQDVHINCTGHG
LDQFPYDVEKEITFFHLGGNNFSEGLHENTFEHLDRLVYLDLMNNSIKHLEPLVFSTLWRL
KTLNLQHNEITILRNCSFLGLGQLTGLHLQGNNIYKLSSMAFQGLSSLTTLDLSHQNITDIE
TEAFVGLRSLKSLDLSHNSLTHIRDGTFRGMPQVVFLNLKNNQLRVIDKNVFFTMPLLETL
FTDEFRFCCLARYVKQCDPLPDEFSSCEDLMSNIVLRVCIWILAVVAITANLLVIVFRAKYK
HTNQVHSFLIVNLALGDFLMGSYLLVIAVVDWYYRGVYFIHDSDWRRSSMCNVAGFISTF
SSELSVFTLTGYRFKSYT
>tetur15g00150
MNCQFNVSPVLLFNLFISLVNGKSRQQLKFQLLLMTFLIPISVVNCCDPGFFPCNDSDICIE
QRLNCNNEPDCPDGSDEEHCEDNHKREYWDKLFRKRPDEDREKKNSTKCAMKNIPGECS
CSMFNVFCEQQNLDRIPKIPMEARILDLSGNRVEKLRRTSFSYLPRLKTLILTSTEIQTIEKD
AFANLPNLSSLFMSGNQLYTINNKIFSNNSNLVLLFLSHNPIQILKNQSFTGLSSLEELDLRN
CRLSKFPKRVFEPLVKLQTLLLDGNLITSLPAKIFRSLNNLQVLSLTKNRINIIQDVNFIGLIS
LRSLSLSINNIEELKDNAFGNLTTLLKLDLRKNKLKLLESNVFSNLSDLESLDVRKNMIKK
LPLNIFDNLQSLTHIYFDEFRLCSYALHVRVCEPRGDGISSFQHLLDNIVLRFSVWVVAFIAC
VGNVFVLIGRMLMHEPNQVHSFFIKNLAFADLLMGIYLFIIAYYDASFRGQYIKHEDAWR
HSWQCNLSGFLSTLSSESSVFILTVITVDRYASVIYPLSQKRRTKGFAISCMVAIWTGAVVL
ALLPLLTDDLFGDEFYGNNGVCLALQIHDPFSKAWEYSTFLFCGLNSGAFIYITYAYINMSL
TIASSRLGLRTTQQQQDRCITKRFGFIVATDCLCWMPIVIIKILALAGVPINDDLYAWVAVFL
LPVNSALNPVLYTLTTKLFKQHFARIIAYGLQRSNSPGGDNNSGIHETRHSNGSTKEDLRSP
SKDSFKNGFSFRKYCLSEQLSTFHREKSTDDEDGRTETF
>tetur09g05380
MEAYFPLVTLQAVVQLHIQGTTATALVEDASMINPMESEFDTNNETICLSGEFRCNDLCIED
VLRCDTKSDCSNGQDELNCESYICPSNHIKCDNHFCISIDRVCDFVDDCGDGSDEQNCTFR
QCWYQEFRCKNEQCIQGYRVCDGKVDCVDGSDEAYCDEKSHFKDCGDGNRAHKSVWC
DGYVNCPKNHADELNCINCSSDEFSCSNTRCIPSSSVCNTICDCVDNCEDEVDCKQFYHQ
VNGLKFCNTNVTFACPWDGPCVTIDSICNNISNCPYDDAYSGALDEYGCGITKEKCETFG
NGFWCPEERCISASLKCNFIPECLNGEDELDCFSEPCDKFQCKNGQCIDFDKRCDSKIDCF
DKSDELDCQNYPCPDDWVQCASGQCVKRSFWCDYTEDCLDGSDEAYCDYKSNPPECDP
LTEYTCKNGQCIKLLNRCLVTQDRRDSCSDGSHLVNCSDYICPTNSIKCANSFCVHSSLVC
DKKIDCLRSWTDEEGCPFVCSSTLCPCIDIVINCTNFGINYIPDDIETGISRIQVKDDEQEESK
DEDTYQQGNNLGSNLTQSTFAKLDTKMVYIDLSNCSIQRLESGVFQSLNLLKVLVLSDNQI
TELTNENIFAGLISLRTIFLDGNGIKMIASYAFKGLSGIKSLDLANQQLTIIKRNTFNGMRSLV
TLDLSNNQLFYLEEGSFTGLIKLTSLDLTGNKFSEMGTQVFTGLTNLKKLSTDEFRFCCLA
RHVQNCLPEPDEFSSCEDLLSNLVLRICIWILGVLSIVGNCMVIFWRTMHRYRAAVSSFLIA
NLAIGDLLMGVYLIIIGTVDFTYRGKYFIHDAHWRSSKMCQLAGFISTLSSELSVFTLTVITI
DRFLRITFPLRFHRFKMTNARLVILATWVFTVILAGVPLLDIKYFDNFYGRSGVCLSLHITN
QRPNGWEYSVFVFLVLNFISFTTIAIAYIWMFTVAKNTRSALKSSDIRLSSTMAKRIMLIVM
TDFWCWMPIIALGVISLNGVKLPPQVFAWVAVFVLPLNAAMNPILYTISTLPFFKRTYSRSA

QESKSSVVLKNGRSKSVIQRKNYRAKHHYFFNSKFTNQSEIKTV
>RPRC001663
MIKNNNPLLLTAYILVHRMMYVCDRTSAENLSKKFGTQINQLWDNFGTDFTYPGNLPAYV
EEYFEEQEYNNKQTDPPPAKIQCLPTPGPFLPCVDLFDWWTLRCGVWIVFLLAMLGNGTV
VFVLIFSRSKIDVPRFLVCNLAAADFFMGVYLGLVTLTLVDASTLGEFEMYAIPWQMSAGC
QLAGFLGVLSSELSVYTLAVITLERNYAITHAMHLNKRLSLKHAGYIMLCGWSFATIMATL
PLLGVSDYRKFATCLPFETSTTWSLTYVVFLMFINGVAFLIRWVAI
>RPRC014721
ETYTCPTTHFKCNNHYCIPIDLLCNFEDDCGDKSDESKDCNHRQCWNLEFRCENGECIRP
GFVCDGRKDCKDGSDEALCAEDDFVMCRDGSRVHRSYWCDGWPDCPGNHADEWNCE
VCDGPNDYKCPNGRCIKKANICDSQCDCAPHNGSLECADEMNCSKYYRSVHGKVDRCIA
SKYICDGSNDCHNGKYLSDEYGCQPSENQYSESTFRCLDNRTLPESLLCDYKNDCLDGDD
ENLCRALYQCDETMFTCNNSQCIDKNGRCNVTYECLDKSDELGCLDVPCPEGMVKCTYG
GQCIPEKLLCDYFIDCPDESDEKNCPVTECNKLQFQCDNGQCVSIEHHCFISGNQRDGCAD
NSHLKNCKNFTCMRDHFKCRLGPCLNQSLLCNKKIDCQHTWEDEDNCTFTCSEKYPECP
CKDIYINCTALGLESVPLDTEGEITWFHLGSNKLNASLTNETFSSLDRLLYLDLSNNSITGLP
PMMFSNLWRLTVLNLQNNRIHTLVSSSFYGLASLKGLHLQGNGIRVVRMLAFYGLSSLRN
LDLHDQNINLIEPDAFLGLRSLVGLDLSQNKIEYISDSTFRGMPHLLYLDISNNYIDVIDAN
AFRMATTLEKLVTDEFRFCCLARHVKSCLPPRDEFSSCEDLMSNMVLRICVWALAVIATV
GNILVIACRARYKHCNQVHSFLITNLALGDLLMGSYLLLIAVVDWHYRGVYFIHDSSWRS
SQLCSFAGFISTFSSELSVFTLTVITLDRFLVIIFPFRVRRLEMTRTRRLMAFGWIVAISISAVP
LIHIDYFKNFYGRSGVCLALHITPDKPNGWEYSVFVFLFLNLVSFTIIAVGYLWMFLVARTT
QHAVNKDRRTSESAMAWRMTLLVATDAACWVPIIILGIVSLAGYTVPPQVFAWVAVFVLPL
NAAVNPVLYTLSTAPFLTPARHGFLTFRRSCKMSLSQDQRRTYTSGLNHYAGKSS
>TPAL_H9TUR5Q01C6ORS_3
TYKQWYQRRVAITAAGSIVSGDGPHCSNLKKIRVVQGYSMDIDNGEVSPIGYIMTIPSSKQ
EQIETLNNERIDCILKNDTNTFADGCPTVWDGILCWPNTPSNTLASLPCPVYFAGFSSHVSQ
NCLFLFFTFHHHHIICIFFKNIWAIYNGISITRLINKNFLFWENV
>Hheb00749 LGR
MERIDSRAFTNLSKLRVLELDDNLLSEIPEAIETISTLEDLSISNNRIDRIPANAFRGNKNLMS
LDLRGNPIKVIDEGALQNHRKLRKLIISNVRSLADFPNLNGTRSLEVLRLDRASLKTVPED
LCKQCPKLKSLDLKSNYLESIPNLTECSDLKVFNMITSLANKPFSGQRFLHDLLLSNNNIKT
IPEEAFAGLVRLQVLNLENNLIEYIHPAAFEAIKQLEDLNLGNNVFPDLPTKGLGNVLHLKT
FNNPALREFPPPHLFPRVQTMILSYAYHCCPFLTAEYNEEVTKSSLQESVVFPDENDLELDP
WNSSLTDSWSSSENSTNKFGAELKNLWDSYEGEYTYPGNVPTYIEDYFEDQEGRVSAPSG
SGFPAHVQCLPQPGPFLPCQDLFDWWTLRCGVWVVFLLAMLGNGTVVFVIVFSRSKLDV
PRFLVCNLAAADFFMGIYLGFLAVVDASTLGEFRKYAIPWQMSLGCQIAGFLGVLSSELSV
YTLAVITLERNYAITHAMHLNKRLSLKHATYIMIVGWSFAFSMAILPLFGVSDYRKFAICLP
FETNGIASLTYVVFLMLINGIAFLILMGCYLKMYCAIRGSQAWNSNDSRIAKRMALLVFTD
FLCWSPIAFFSLTATFGLQLVTLEQAKVFAVFILPLNSCCNPFLYAILTKQFKKDCVLICKAIE
ESRVTRGIGRCRHSSNFSNRPTAANTNSLVDRSSRENHQPQCACNARLLEVNECRKTWWN
SRILWSCRREMRSRPYNRSDAYAYQIAEIQQKQNKRASSMSSSENYSSSRSDSWRQAHHC
GIPLRLLDPTRRASSWLITRKTSQDSNLSSSRNDSSGSATTASTSTWRMSRSSASLEFNSRTT
PRPARSKPRLIRQFAIQEPEPPGSPSRLAVRLLATIPSAAEMSEQQEEESNADKE

RGCs

>KAF7988833.1 hypothetical protein HCN44_007143 [*Aphidius gifuensis*]

MRPGPPSPSRRRPARRQQQPHHHHYHHHHHHHHHYNNISLNTIKTNNHNKHNIFGDTSHT
NTWSILFLLILLVVGPCLALNETSNNPIDIINHGNDNSNLTIINNNNNNFYHNINNTNHERA
NFKPKNLTIGYLTAIKGGLKDRQGLAISGAISMALDEVNNDPNILPTVNLLMKWSDTRGET
VDATRAMVDMICDGVAAFFGPEGQCFVEATVAWSKNIPMISYKCSDYMASTVPTFARTEP
PDTQVTKSVIALLRHYGWNKFSIITEKAWSSVAKSLENQATAKPNNLTINHHKIVEDRHTC
CEERLPCCQPNLWFQLIQETKNMTRIYIFLGTAISLIDMMNAMQNQRLLDNGEYMVIYID
MMTYSPREAQKYLWKPENFDNTKNCLEPKDFIKRARSLLVVASTPPIQSYEEFTTKVRKY
TSRDPFNFTVPDLFVNFEKYVSIHAAYLYDSVKLYATALDQLIREQPNLSVEEVASNGTKIIE
TIIQKHMYQSVSGQTIKLDMNGDSEGNFSVLALKKGRYEIHNFSCDYQMKPVGQFQQQG
DTLVYRPSESMDWPGKNKPEAEPGCGFLNEHCPKNDNHTASLIVAIVLSVVLLCTILVISSI
YRRWKIEQEIEGLLWKIDPKDIHGYPHLDNLMSSPSKLSLVSAMSYESRCGGQVFAQTGR
YNGATVRIKELKFSKKKDISRDVMKEMRALREIRHGNLNSFIGACVEPMRILLITEYCAKG
SLYDIIENEDIKLDAMFIASLVHDLIKGMLYIHESTTLRCHGNLKSSNCVVTSRWILQVSDF
GLHDMRHCAESDSIGEHQYYRNMFWKAPELLRDPSASLKGTPEGDIYSFAIILYEMIGRKG
PYGGVDMEPKSIIDKVKCPPINKEKPFRPNIDILLDTNIKCPDYVISTITDCWSEYKELRPEF
KTIRTRLKKMKAGKHRNIMDQMMDMMEKYANNLEDLVSERTRLLAEEKHKTEDLLHR
MLPEPVANCLTNGIGVEPEAFDLVTIYFSDIVGFTAMSAESTPFQVVNFLNDLYTLFDRVIK
GYDVYKVETIGDAYMVVSGLPIKNGNRHAGEIASMSLDLLSVVKQHTIAHRPNETLKLRI
GIHTGPVVAGVVGLTMPRYCLFGDTVNTASRMESNGEPLRIHISGECKESLDKIGGYIIEER
GLVHMKGKGNVKTYWLTGANEKAIQKREVDIGDLPPLFCRPRRSPKLNCDSRQASLLGG
FCAGSRLITTLPRLNTEDTLSQFDNSSPALARNLRIGKLDKNQIIMIDSSSKTTIDNPTLREE
KQIRAANIKLALDDYYFELSPEKNKNKRILSTIASSSTTSDYPLQIIRESRSLDPFPLYNNND
SSNLKNRLSKKSFKSLESCNKCYKNYHDNNYNKIVNNNYSNGNTLQCNNDINDDLIDND
VDEPLLSDSYHCINDTIDLSKSTQSLDHVLPEQCNDNVQDKKTSARNSIKSWIVNFISGNN
GIRNSDVSLRNGGVIGYDIQSERESIV

>KAF7988832.1 hypothetical protein HCN44_007142 [*Aphidius gifuensis*]

MDWSTILSTAKPSPLAILLGPNDVLVNTGELGKRGHKLEFIIGETYGEEETSIRVTADFWTK
NVSAYIGPQETCVHEGRMAAAFNLPMISYFCMHRETSNKAEFPTFARTKPSDTQISKSVAS
VLLAFNWTKITFVHINNSDTEFNNKMLTVSTAILETCETFGIIVNHHKRWSESYHTERMINP
FHDIVESTYIETRIYVILGNYDEHMGLLMALDQKKLLDNGEYWVVGVDIEQYDKNRPDK
YLHGLLEENTNPSLLRAYESYFSIVGSATDYSDNFTNIINEYRKKPPFNFINPIENYGGVVQI
VPETIYLYDAVHLYAKSLLSALSEGRDPRDGRKMLAGLYGTHYRSAMGYLVYMDKNGDA
EANYTLIALENNPDKGHGLYPIGHFVGKEEITNLPKLRLVKKISWPDGAPPIAEPFCGYHGE
KCNSHTGEIIGGIAGGFLLILMAIVLVLYRNWRYEQELDSLLWKVNYKDIQIKEEKEETVQ
NDAIQKSNIKPVVRTSQVSLSSNPDADFRYSMIYTQIGFYRGRMFAIKNVRKKSIEITREMK
KELKVMRDLRHDNLNAFVGACTDPPNICIVVEYYMKLDNMFMASLVGDVIRGMMFLHE
SVIKYHGNLTSSNCLVDSRWVVKIADFGLREFKRDAESPELLRTRMDEPNTREYQKSDVY
SFSLVLYELHGRHGPFGVTQLTSSEILKRIIDSGPDIDLFRPPLDQLENCFDFVRDCLVECWS
EDPENRPDFKIIRNKLRPLRKGMKPNIFDNMMAMMEKYANNLEALVDERTDQLNEEKKK
TDALLYEMLPRFVAEQLKRGHKVEAECFDCVTIYFSDIVGFTSMSAESTPLQVVDFLNDLY
TCFDSTIENYDVYKIETIGDAYMVVSGLPIRNDVQHAGEIASMSLCLLEAIKQFTIRHRPMD
KLQLRIGIHSGPVCAGVVGLKMPRYCLFGDTVNTASRMESSGSPLKIHCSQETKFLLDRLG

GFHLVERGIVSMKGKGDRLTYWLVGEELLMREKRKHDRIERKNGKMPDLLVPKSSMKN
KKLNRHFLRCSNNSQKRLRFASSDHIDKKIINNKLDSIIDNSPCKAISSICNLKNFMDHSRS
SSNSCPCVEQLDYSDEPIDCQQFVQKNDCFFPPLLYDNYCKSEPSSPRHSANILNCQKNVQ
SIDEVDGWDATPLIRSNTYRWLQLNCEISNARKNWPKDDCKIANKIRASFYRCQSAMLFR
>KAF7995591.1 hypothetical protein HCN44_006698 [*Aphidius gifuensis*]
MASRWFGKIAILSVLLLIDPVFINMICHETKLPRDCESLCSKDQWGFVSCELRAAVLLPND
TRFDISLQHVLPVLELALTKVRAQKLLPNWLEIKFLPKDDHCDATYAQIAAIDSYADCVHL
FLGPACEYCVANVGRVVKFLGAPLITTGGFTYDFTEKKTECSDEYFMTTRIGSLSFRDISSF
FLSIMDKYQWKKVHLIYAPNGQPNFAGRHTCQLMMKSMAQAIKNHENISFGNFDVEALT
LEEYPESLRTNIGNYYGDAFFGPVCDYAIAPVARYAGVWGIPVLTTGAQADAFRHKGENF
PTLTRMMGSHRLVGEALRHILQGFGWTTAALIYHNHPMESSKGMSNCHFTLSAVFSALNK
TSVHKSFNQETNTPNDYRNLLTFVANSARSNDNNSKEPWRMENDTDERNEKAKKAYQAL
LTVTARTPDNDEYLNFSREVKSLAQSRYNFTFGNSSLSTFVAAFYDAVFLYALALKESLPD
KPGEVNLDGGNLTRRMWGKSFKGITGDVNIDENGDRIADYSLLDMDMETSKFEIVANYY
GANKTLEYVPGKRIHWSGGRLEPPPDTPKCGYDGSLCPDNTLPGYAILSIVLSSIVVGLIVV
SIIMYRHYKLEAEIASMTWKVHWEDVIVVAPGKPRGSMYSLTGKQHRGSQLTVYSDDNM
SLAGGVDRMVYIPTGFYKNSKVAIKPIPRNKVEISRPLLLELKRMKDLQHDHLVRFSGACV
EPPHCCLLTEYCPKGSLQDILENEQIKLDRVFRGSLIHDIVRGMAYLHASEVKSHGNLKSS
NCVVDSRFVLKIADFGLHELRKPCPGDPDDDKNSHAYWRRQLWTSPELLRMERSPPEGT
QKGDVYSFAIIVHEIVVRQGPFYLGEGNKYSPKEIVEAVRKGGGSPLRPLIDEAAVEEEVAT
LMRRCWAQDSADRPDFPALKQTIRKINKDYESSNILDNLLSRMEQYANNLETLVAERTAD
YLEEKRKCEELLYQLLPKSVASQLILGQSVIAETYDQVTIYFSDIVGFTSLSAESTPLQVVDL
LNDLYTCFDSIIENFDVYKVETIGDAYMVVSGLPMRNGMNHAREIARMSLALRDTVMTF
RIRHRPHEQLNLRIGMHSGPCVAGVVGLKMPRYCLFGDTVNTASRMESNGEALKIHVSPK
TKEILDKFGTFELCCRGEVTLKGKGKMTTYWLVGEKPLNNNIQQSTVSTTIDPSSPQHQQI
QNQQCQRIVNITGNGTSSNHSLTLSPTQQQCNINNSMSTHIGNTIHQSDNVPNHNESGPNA
PLLMPAGSIPRA
>KAF7996629.1 hypothetical protein HCN44_002275 [*Aphidius gifuensis*]
MARSRLHQDWLILSLILSLSSAIPPPPPQPPPQRPIITNANRLVVDDSNLMTSTFSTMRKNIT
KTTSDVVIFLRDNCNNNTSNNYNNQDNEIINFLGSYKTNGKLSIGILQKSLACESKSWGLG
ALVQVLEQSTTKALIAALDVNTCQVAERLAFLWNKPLLTWTCQTKTLKNNEATSMIRLTP
FLPIIAQALSELFLHFKWKSVAVISIDEEPWLSLENLIIGALKNIGIIPRHHAIISRQSTSQEIHQ
SLVSLESIISRVIVLCIPADDEEFISRIFKKLETISKTDKSLFILVHPGGPELFLPPSSTTTKIKSNI
EKSLSDNSTSKKVLSFANTTYENNSNNKHQKFPRKFQGHMKNLMVFLSFDKRYNLEEIYN
GTNVYHEKNLIDYLNESLGILLNDSNNNNDNLNSNNNKMFTYALLDSRKFLNNVNNNNN
TDDYNWQPLVQVIESWDNLIVQELVVNNNLTDEEIILDPINCLTDNDCKDEHVDNSADNN
NVNNNNNLPLRTSHIVAVVLVCLLLTLLIIIIGILIRRHLINKRISKGPFKIILTASDFVFPHIAD
GRRVDEGIEAMLCCWLQQLQEFGGPEIEKPDLLQGSVGSLKPHFKTSTGSLSRLNIVGDSR
ARYNGDLVQLKEVPCQTTFELKSKAMDVLVMMHGLRHENLNPFIGCLTEPSRPCIVTEYC
SRGSLEDVLVQDVKLDWSFRLSLLTDLVRGMKYLHNSPIRVHGYLTSRNCVIDARWVLKI
ADYGLPAFYEAQNIVMPAKTARDLLWTAPELLRHTALRKRGTQPGDVYSFGIIMQEVIVR
GEPFCMLARSPEEIIEKVKKPPPLIRPSVSHQAAPPEAINIMRQCWAEAAEMRPDFNAVHDL
FKKLNHGRKVNFVDTMFQMLEKYSNNLEELIRERTQELDIEKRKTEEILNRMLPGSVAEK
LKLGMPVDPEEFDEVTIYFSDIVGFTTISANSTAFQVVDLLNDLYTCFDDTINAYTVYKVET

IGDAYMVVGGCPVRIQDHPSQIATMALDLLHQSGKFKVRHLPKTQLQLRIGLHTGPCCAG
IVGLTMPRYCLFGDTVNTASRMESTGAPWRIHLSQATRDRLTQVGGYHIEYRGSTDIKGK
GKMPTYWLLGKQGFDKKLPTPPPFGLDESLMIKKSASTEDLKVTIENFDNSTKLPQVQVQ
VQTTDECIDADAPSISSKDEDENCKSNSSISTNSKYNGANGGLGVVASDCAGVITKNVVVT
VHDEKDKDTSVITTNNNNNNNSSINTGIQSASASRVAAALLGSCGASTTSLSSIVTATTAFRN
SSARHRHRVFNADDLSSPYNHYRRLSPNEHGKGSSGRMLKRQFSLDRADEPSISIISDSIMS
AAASSRISSRLYKQNSAGAANDLEKIEEVVQLNNNNNSNNNPATGSSLSSSSSSHHPPYRH
AASMSLSVESLTMR

Other insects:

>Hheb03289 NPLP
MQSIENQQLEQIGQNSIALIGNDRIKNLTIGYLTAIKGGLKDRQGLAISGAISMALDEINNNP
RLLPNVQLVMRWSDTRGETVEATKAMIDMICDGVAAFFGPEGSCYVEAIVAQSRNIPMISY
VPTFARTEPPDTQVTKSVIALLLHYGWNKFTIITEMAWISVAKSLENQAARNNLTVNHYKT
VEDRHTCCEERLPCCQVSVWFQLIQETKNMTRIYIFLGTAMSLIDMMNSMQNQRLFDNG
EYMVIYVDMMTYSQKEAQKYLWKPEHFDNLKNCLEPKDFLKRARSLMVVASTPPTQSY
EEFTKKVRNYTSNEPFNFTVPDLLMNMKFDKYVSIHAAYLYDSVMLYAMALDQLIRERPE
STIDELASNGTLIIETIIKKHTYLSVSGQTIKLDKSGDSEGNFSVLALKKEFFQRNNFSCDFQ
MKPVGQFQQGETLVYRPSESMDWPGKNKPEAEPGCGFLNEHCPKDDTHMRGVVVAGLL
AVTLFCAAVITMSIYRRWKIEQEIEGLLWKIDPNDIAGYPVNDKIMASPSRLSLASAMSMES
RVGGQVFAQTGQYHGVVVRIKELKFSKKKDVSRDVMKEMRALREIRHGNLNSFIGACVE
PMRILLITEYCAKGSLYDIIENEDIKLDIMFIASLVHDLIKGMLYIHESSVLVCHGNLKSSNCI
VTSRWVLQVSDFGLHDMRHCAESDSIGEHQYYRNLFWKAPELLRNPHASIKGTQEGDIY
SFAIILFEIIGRKGPWGGVNLEPKAIGEPDKECPEYIVSTITDCWSESPELRPDFKSIRTRLKK
MKAGRHRNIMDQMMDMMEKYADNLEELVSERTRLLFEEKQKTEDLLHRMLPEPVAHCL
TNGIGVEPEAFDICTIYFSDIVGFTAMSAESTPFQVVNFLNDLYTVFDRIIKGYDVYKVETIG
DAYMVVSGLPIKNGNRHAGEIASMSLDLLNAVKHYPIAHRPKDTLKLRIGIHTGPVVAGV
VGLTMPRYCLFGDTVNTASRMESNGEPLRIHISAQCKEALDKIGGYIVEERGLVQMKGKG
EVKTYWLVGANEKAIQKREVDVTDLPPLFCRPRRSPKLNPDSRQASLLTGLAAGSRRTSC
VPRPSPDDSASQCGNSSPAPARQMRLSKLERNQLHLVDSKTTLDNVTVCEDAEIRAATIKQ
VLDGVFPENQQEAQRVLSTIASSSTTSDHPSAIIRESKSLDPFPSELHRDSPDVQRKEPKRSF
RSLENVDNYGSKGDLRILNNNHPNGDIILKDNFVQDEEVNAPLLGDHNEAEMGIIKKWRS
LDQVLVNNVSGDMVPEKKSSARNSIRSWLVNLFNGNTIRSSNVSIRRTVITGYDLQGERES
IV

>Hheb03286
MQLINLERACLLVSLLSGFVGAETFTLGYITGSKRRINDLEYERPGIRISGAINLAVEEVNSG
ELGKLGHKLDFIVAETYGEEDTSILVTADLWTKNISGYIGPQETCVHEGKMAAAFNLPMIS
YFCTHHETSNKAEYPTFARTRPPDTQISKSVVAVLLAFNWTKVTFMYMNSTVTEFNSNWA
SVASTILELFQSSGITVTHERCWDEPYHTINVSNPFYRLVETSYKETRIYVILGNYDEHRGL
LMALDEKKLLDNGEYWVVGVDIEQYKAKYPDEYLRGLLQDRTGPSLLRAYRSYFSIVAS
APNISKKFTKIINEYRTKPPFNFKNPLQRFGGIVEVVPETAYLYDAVHLYARSLLSALKEGR
DPRDGRKMVEMLHGVHYRSAMGYMVYMDRNGDAEGNYTLIALENHPDKGHGLYPIGH
FVGKEESSNLPKLHLTRNITWLAGGPPVAEPACAHTGEIVGGIAGGILLILLAIVLVLYRNW
RYEQELDSLLWKPVVRTSQVSLSSNPDADFRYSMIYTQIGFYRGRMFAIKKIRKKSIEITRE
MKKELKVMRDLRHDNLNAFIGACTEPPNICIVVEYCPRGSLKDIIENEDMKLDNMFMASL

VGDIIRGMMYLHESVIRYHGNLNTSNCLVDARWVVKIADFGLREFKRDAECDSQDILKKY
QSLLYRAPELLRSRQLPPSIRDFQKADTYSFSIVLYELHGRQGPFGPTHLSPADILAHLCNPT
PSTPPLRPALDDLENCFDFVRDCLEECWSEDAELRPDFKTIRNKLRPLRKGMKQNIFDNM
MAMMEKYANNLEALVDERTDQLTEEKKKTDALLYEMLPKYVAEQLKKGHRVEAEGFDC
VTIYFSDIVGFTHMSAESTPLEVVNFLNDLYTCFDATIENYDVYKVETIGDAYMVVSGLPIR
NGIQHAAEIASMSLCLLDKIKEFTIRHRPCEKLQLRIGIHSGPVCAGVVGLKMPRYCLFGD
TVNTASRMESTGLPLKIHCSQETKELLDKIGGFYLEERGIVNMKGKGERLTYWLYGEHRG
IRDARYQQIEQSLVPKSSLKNKTIRMSFLRCCSESPKRLRFASSDQLDGGNIENDEWSPCKG
CMEGSKSASSSCPCVEKIEFGGSCFKDACKSVPASPNFLGKGKFCEILEGEADPLI
>Hheb087320.1
MARSGLHQDWWWLRMTIFICLLTGQYALPQRPIRTGLPIPMPPIDRDQSDVVIFLRNNCNT
DKTSTISDEANEIGQFLSKYNEDGQLSVNILEKYLACGTKSWGLQVLIDVLGQASTKALVA
ALDVNICEVAEKLAHLWNKPLLTWTCPARMEDTNERTSTIRLSPSLPAVAQALGEIFLHFK
WKSVSIISLDEEPWLSLDRAVIGVLRSVDIIPRHHVLVSRNARSEQIHQSLSPLESVIPRGIVL
CLPIEDEKLMSRVMGELKAIRTSRTDNSLFLLVDPEGPGFFLPSPLTGEEEKSVESLADNST
GRPASMTSRWWHTQRQNFDQIPKRLGGRTTMNLLAFAPFDKRYDLLKIYNGTQEYSEPR
MLDHLNESLTILTRDNSTLNTNNNKMFTYGLLDWRSSGSGANDGHWQPIAEVIESRDRLD
VRELIENDLIDEETLDLALCLSGNDCGGHNNNVEGDEKETEEREDHQLPLKTSHIIVIILICF
LLIVVLFIITLLVRRHIMTKRVAKGPFKIILTASDFVFPQPADNRRVDEGIETMLCCWLQQLQ
EFGGPEVEKPDLLQGSVGSLKPHLKASTGSLAWHTILKDPRARYNGDLVQLKELPCQSNF
ELKSKAMDVLVMIHGLRHENLNPFIGCLTEPARPCLVSEYCARGSLEDVLVQDEIKLDWSF
RLSLLTDLVRGMKYLHSSPIRVHGYLTSRNCVIDARWVLKVADYGLPAFYEAQNIVPPPKS
ARDLLWTAPELLRHTGLRRKGTQPGDVYSFGIIMQEVVVRGEPFCMLALSPEDIIEKVKKP
PPLIRPSVSKGAAPPEAINIMRQCWAEAADMRPDFNAVHDLFKKLNHGRKVNFVDTMFQ
MLEKYSNNLEELIRERTEQLDMEKKKTEQLLNRMLPSSVAEKLKLGMPVDPEEFAEVTIY
FSDIVGFTTISAHSTPFQVVDLLNDLYTCFDDTINAYNVYKVETIGDAYMVVGGCPVRIQD
HPSQIATMALDLLHQSGKFKVRHLPRTQLRLRIGLHTGPCCAGVVGLTMPRYCLFGDTVN
TASRMESTGAPWRIHLSQATRDRLCQVGGYHIEYRGCTDVKGKGKMPTYWLLGKQGFD
KQLPTPPPLGDDHGLEESLEGFKLEGIDDPKSECSEDSPTTTTTTEAHVLHKQATDERTTSS
LKDEDDNSSGQSVCSFTTTCTSSKFSNAPTSLTRKVAVSVHDEREPTAAPSNLSAATGPLLG
PAASTTSLSSISSSAFRPATSAPSRHRRVGHIDEDDLSTPYNHYRCLSPNEHHTKSSSRLLKR
QFSLDRADEPVSIISESSMSLISTRPPPRLYKQNSAGAANDLEKIEEVPSLPPPHQTYRHAAS
MSLSVESLTLH
>PPU05511-RA NPLP
MRPGPPSTSRRRPPRRQQQHQTPQEQHLHRIHQPSLHRPTRLYRPSVGSSRQQQQQQRHR
CGPLGVLLLLLLLATCPGRAESPSSGQDVTSSSLPVQHQPRKPQNLTIGYLTAIKGELKDRQG
LAISGAFSMALDEINNDPNILPNVKLVMRWNDTRGETVEATKAMVDMICEGVAAFFGPEG
SCYVEAIVAQSRNIPMISYKCSDYKASNVNTFARTEPPDTQVTKSVIALLLHYNWNKFSIIA
EKPWSTVAKSLQNQAAYNNLTINHFLSVEDRHVCCEDRLPCCQGGGWFPIIQDTKNMTRI
YIFLGTPISLIEFMNAMQNQRLLDNGQYMVIYVDMMTYTPKEAMKYLWKPEHYDNLRD
CQDPKDKDFLKRARSLMVVASTPPTQNYEEFTQKVRDYSSKEPFNFPVPERFLLDKFEKY
VSIYAAYLYDSVKLYARALDQLLRDYPDQPLEEIASNGTLIIETIIKNHTYQSISGATIKLDSH
GDSEGNFSVLALKKEPLHIQNFSCDFQMKPVGQFQQGDNLAYRPSEAVDWPGKNKPEAE
PGCGFLNEHCPKDDTHMRSIVAAGVLAVLLFCAAVITMSIYRRWKIEQEIEGLLWKIDPSEI

HGYPHLDNMMSSPSKLSLVSAMSYESRCGGQVFAQTGHYHGVVVRIKELKFSKKKDISR
DVMKEMRILREIRHGNLNSFIGACVEPMRILLITDYCAKGSLYDIIENEDIKLDDMFIASLIH
DLIKGMLYIHESSVLVCHGNLKSSNCVVTSRWVLQVSDFGLHDMRHCAESDSIGEHQYYR
NLFWKAPELLRNLHAPIRGTQEGDIYSFAIILFEIIGRKGPYGGVNLEPKEIIDRVKRYPEDG
EPPFRPNVDILSESEADCADYIVNTITDCWAESPELRPDFKTIRTRLKKMKAGKHRNIMDQ
MMDMMEKYANNLEDLVSERTRLLFEEKQKTEDLLHRMLPEPVANCLTNGIGVEPEAFDL
VTIYFSDIVGFTAMSAESTPFQVVNFLNDLYTLFDRIIKGYDVYKVETIGDAYMVVSGLPIK
NGNRHAGEIASMSLELLNAVKHHTIAHRPAETLKLRIGIHTGPVVAGVVGLTMPRYCLFGD
TVNTASRMESNGEPLRIHISAQCKDALDKVGGYIVEERGLVQMKGKGEVKTYWLTGATE
KAIQKREVDVNDLPPLFCRPRRSPKLNPDSRQASLLAGLGAGSRRQSSVPRPTPSDNTDSA
SQGGNSSPLQARPALTARKLERSPLYLTESSSKTTLDNMAIVREEAETRAANIKLVLDDLFP
NNSDRHPVLAGNGLPSLQPSGAKFRKNARVLSTIAASSTSTSDHHGQPAAQCKALALRES
RSLDPFPVDMLNSNSRKLEPAQLLTWKLQPRKSSFRSLENCDKCSPSRRGSKTSLANEKLL
NNNYPNGNVIIVPAQSNGDDQRANSNVTSHQQQLVPFQHNDQDAAETPLLLGENCLLSSG
ELSMPVKRWRSLDQVAAPDNGSSGGGCVAVGGGLSDKKSTARNSIRSWLANLFNGNGLR
SSDASLRRGVIPGYDMQSERESIV
>PPU05512-RA   EH
MPVAQVERACLLVTTLLVHALLDGSGSVNAETFTLGYITGSKRRPGDWEYSRPGLQISGAI
TLAIDEVNSGELGRRGHRLNFNVAETYGDEERSILMTADLWTRNVSAYIGPQETCVHEGR
MAAAFNLPMISYYCTNHETSNKKEFPTFARTRPPDTQISKSVVAVLKAFNWTKSSSTTAVA
FMYMNASSFDPYERPTVAKTILSSLRSAGISVNSISSWEESYRVVENLINPFHKLVAETHVE
ARIYVILGNVEEHIGLLMALDKRKLLRTGEYWVVGVNTETYTDREPDVYVRGLMRNHTD
THSLRILQSYFSIIATAPIGYLNFTNKVNEYRQKPPFNFRNPLKNFKEEGSIQAVPETAYLYD
AIHLYAQSLIKALDEGRDPRNGKEIISSLYGLHYRSAMGYMVYMDENGDPEGNYTLIALD
NQAPKGPGLYPIGRFIGKENHTNLPKLHVIRSIPWVNGRPPVAEPYCGYHGEKCYSHTGEI
VGGIAGGLLLVLLAISLMLYRNWKYEQELDSLLWKVNYKDIEIKETKDDSTSPSEQQLFK
NNSKNPLQPHVRTSQASLSSNPDADFRYSMIYTQVGIYKGRIFAVKKVKKKSIEISREMKK
ELKIMRDLRHDNLNAFIGACTDPPNICIVVEYCARGSLKDILDNEDIKLDNMFMASLVGDI
VRGMIYLHESIVKFHGSLTTSNCLVDSRWVVKLADFGLHEFKRDAELEPADVMKKYRGL
LYKAPELLRPRAVEPTIRDFQKGDVYSFAIVLYELQGRHGPYGITELSAPDILKRIITIENPPF
RPPLDQLENCFDFVRDCLLECWAENPEFRPDFKVIRNKLRPLRKGMKPNIFDNMMAMME
KYANNLEALVDERTDQLSEEKKKTDALLYEMLPRYVAEQLKRGHKVEAENFDCVTIYFSD
IVGFTAMSAESTPLQVVDFLNDLYTCFDSTIENYDVYKVETIGDAYMVVSGLPIKNGIQHA
GEIASMSLHLLDAIKQFSIRHRPLDKLQLRIGIHSGPVCAGVVGLKMPRYCLFGDTVNTAS
RMESTGSPLRIHCSTETKQLLDQLGGFSLAERGLVSMKGKGERLTYWLIGEEPSMREERN
RERLARRAGLTGGTDNLLHPSSSGGYLDPLVPRSSLKNKSLARAAFLRCSSESPKRIRFASS
DHLDQAAKGSRNNDGNKLESIVDGSPCNGRGLCSSGRSSCMEGTRSSSSSCPCVEHIGEA
PLPLRLPAAVQLKLAPHLDCNDQLTNSAPILRAESPLAKDSEIDDEDNDGDTGWRVGGKV
LRFGKKGLLRAACRSAPSSPKRGSTLLVSSPKRAVQSNEELDEWDVAPLIYCNSSSRRFE
>NV15866-RA
MPVAQGERACLLVTTLLVHALLDGSGSVNAETFTLGYITGSKRRPGDWEYSRPGLQISGAI
TLAIDEVNSGELGRRGHRLNFNVAETYGDEERSILMTADLWTRNVSAYIGPQETCVHEGR
MAAAFNLPMISYYCTNHETSNKKEFPTFARTRPPDTQISKSVVAVLKAFNWTKVAFIYMN
ASSFDSYERPTVAKTILSSLRSAGISVNSISTWEESYRVVENLVNPFHKLVDETHVEARIYVI

LGNVEEHIGLLMALDKRKLLRTGEYWVVGVNTETYTDREPDVYVRGLMRNHTDTHSLG
ILQSYFSIIASAPIGYLNFTNKVNEYRQRPPFNFQNPLKNFKEEGSIQAVPETAYLYDAIHLY
AQSILKALDEGRDPRNGKEIISSLYGLHYRSAMGYMVYMDENGDPEGNYTLIALDNQAP
KGPGLYPIGRFIGKENRTNLPKLHVIRSIPWVNGRPPVAEPYCGYHGEKCYSHTGEIVGGIA
GGLLLVLLAISLMLYRNWKYEQELDSLLWKVNYKDIEIKETKDDSTSPGEQQLFKNNSKN
PLQPHVRTSQASLSSNPDADFRYSMIYTQVGIYKGRIFAVKKVKKKSIEITREMKKELKIMR
DLRHDNLNAFIGACTDPPNICIVVEYCARGSLKDILENEDIKLDNMFMASLVGDIVRGMIY
LHESVVKFHGSLTTSNCLVDSRWVVKLADFGLHEFKRDAELEPADVMKKYRGLLYKAPE
LLRPRAVEPTIRDFQKGDVYSFAIVLYELQGRHGPYGITELSAPDILKRVITVENPPFRPPLD
QLENCFDFVRDCLLECWAENPDFRPDFKVIRNKLRPLRKGMKPNIFDNMMAMMEKYAN
NLEALVDERTDQLSEEKKKTDALLYEMLPRYVAEQLKRGHKVEAENFDCVTIYFSDIVGF
TAMSAESTPLQVVDFLNDLYTCFDSTIENYDVYKVETIGDAYMVVSGLPIRNGIQHAGEIA
SMSLHLLDAIKQFSIRHRPLDKLQLRIGIHSGPVCAGVVGLKMPRYCLFGDTVNTASRMES
TGSPLRIHCSTETKQLLDQLGGFSLAERGLVSMKGKGERLTYWLIGEEPSMREERNRERLA
RRAGLTGGTDNLLHPSSSGGYLDPLVPRSSLKNKSLARAAFLRCSSESPKRIRFASSDHLDQ
AAKGSRSNDGNKLESIVDGSPCNGRGLCSSGRSSCMEGTRSSSSSCPCVEHIGEAPLPLRLP
AAVQLKLAPHLDCNDQLTNSAPILRAESPLAKDSEIDDEDNDGDTGWRVGGKVLRFGKK
GLLRAACRSAPSSPKRGSALLVSSPKRAVQSNEELDEWDVAPLIYCNSSSRRFE
>NV15865-RA
MGEERKEALTGGGGGHAPRPAEHEPPQTSLHRPTRLYRPSVGSSRQQQQRHRCGVLGVIL
LLLLATCPGRAEPPSSGQDVTSSSLPVQRQPRKPQNLTIGYLTAIKGELKDRQGLAISGAFS
MALDEINNDPNILPDVKLVMRWNDTRGETVEATKAMVDMICEGVAAFFGPEGSCYVEAI
VAQSRNIPMISYKCSDYKASNVNTFARTEPPDTQVTKSVIALLLHYNWNKFSIIAEKPWST
VAKSLQNQAAYNNLTINHFLSVEDRHVCCEDRLPCCQGGGWFPIIQDTKNMTRIYIFLGTP
ISLIEFMNAMQNQRLLDNGQYMVIYVDMMTYTPKEAMKYLWKPEHYDNLRDCQDPKD
KDFLKRARSLMVVASTPPTQNYEEFTQKVRDYSSKEPFNFPVPERFLLDKFEKYVSIYAAY
LYDSVKLYARALDQLLRDYPDQPLEEIASNGTLIIETIIKNHTYQSISGATIKLDSHGDSEGN
FSVLALQKEPLHIQNFSCDFQMKPVGQFQQGDNLVENVQSSLTRGAVDWPGKNKPEAEP
GCGFLNEHCPKDDTHMRSIVAAGVLAVLLFCAAVITMSIYRRWKIEQEIEGLLWKIDPSEIH
GYPHLDNMMSSPSKLSLVSAMSYESRCGGQVFAQTGHYHGVVVRIKELKFSKKKDISRD
VMKEMRILREIRHGNLNSFIGACVEPMRILLITDYCAKGSLYDIIENEDIKLDDMFIASLIHD
LIKGMLYIHESSVLVCHGNLKSSNCVVTSRWVLQVSDFGLHDMRHCAESDSIGEHQYYRN
LFWKAPELLRNLHAPIRGTQEGDIYSFAIILFEIIGRKGPYGGVNLEPKEIIDRVKRYPEDGE
PPFRPNVDILSESEADCADYIVNTITDCWAESPELRPDFKTIRTRLKKMKAGKHRNIMDQM
MDMMEKYANNLEDLVSERTRLLFEEKQKTEDLLHRMLPEPVANCLTNGIGVEPEAFDLVT
IYFSDIVGFTAMSAESTPFQVVNFLNDLYTLFDRIIKGYDVYKVETIGDAYMVVSGLPIKNG
NRHAGEIASMSLELLNAVKHHTIAHRPSETLKLRIGIHTGPVVAGVVGLTMPRYCLFGDTV
NTASRMESNGEPLRIHISAQCKDALDKIGGYVIEERGLVQMKGKGEVKTYWLTGATEKAI
QKREVDVNDLPPLFCRPRRSPKLNPDSRQASLLGGLGAGSRRQSSVPRPTPSDNADSASQ
CGNSSPLQARPALTARKLERSPLYLTESSSKMTLDNMAMVREEAETRAANIKLVLDDLFPN
NSDRHPVLAGNGLPSLQPSGAKFRKNARVLSTIAASSTSTSDHPGQPAAQCKALALRESRS
LDPFPVDMLNLNSRKLEPTQLLTWKLQPRKSSFRSLENCDKCPTSRQGSKTSLANEKLLN
NNYPNGNVIIVPAQSNGDDQRANSNITSHQQSHQQQLVPFQHNDQDAAETPLLLGENCLL
SSGELSMPVKRWRSLDQVAAPDNGSSGGGCVAVGGGLADKKSSARNSIRSWLANLFNGN

GLRSSDASLRRGVIPGYDMQSERESIV
>CG42636
MTRWPFNLLLLLSVAVRDCSNHRTVLTVGYLTALTGDLKTRQGLAISGALTMALDEVNKD
PNLLPNVYLDLRWNDTKGDTVLATKAITEMICDGIATIFGPEGPCYVEAIVSQSRNIPMISY
KCAEYRASAIPTFARTEPPDTQVVKSLLALLRYYAWNKFSILYEDVWSPVADLLKDQATKR
NMTINHKQSFIDNRVKCCEQMLDCCRSGYWYQLVQNTMNRTRIYVFLGAANSLVDFMSS
METAGLFARGEYMVIFVDMMVYSEREAEKYLRRVDQITFMSNCHSTENFNQMARSLLVV
ASTPPTKDYIQFTKQVQKYSSKPPFNLEIPRLFVESNFSKFISIYAAYLYDSVKLYAWAVDK
MLREETRVLTDDVIFEVASNGTRVIDTIIKNRTYMSITGSKIKIDQYGDSEGNFSVLAYKPH
KWNNSNNMPCNYHMVPVAYFHQGEEHPEYKLINGSIDWPSGGEKPADEPMCGFANELCK
KDDTHYTSTVAAVVLGVLLFCSGVITMSIYRKWKIELEIEGLLWKIDPNEIKGYSGNEIVSS
PSKVSLMSAQSYGSRWTNQFVTSTGRLRGAVVRIKELKFPRKRDISREIMKEMRLLRELR
HDNINSFIGASVEPTRILLVTDYCAKGSLYDIIENEDIKLDDLFIASLIHDLIKGMIYIHNSQL
VYHGNLKSSNCVVTSRWMLQVTDFGLHELRQCAENESIGEHQHYRNQLWRAPELLRNHI
HGSQKGDVYAFAIIMYEIFSRKGPFGQINFEPKEIVDYVKKLPLKGEDPFRPEVESIIEAESC
PDYVLACIRDCWAEDPEERPEFSVIRNRLKKMRGGKTKNIMDQMMEMMEKYANNLEDI
VTERTRLLCEEKMKTEDLLHRMLPQSVAEKLTMGQGVEPVSYDLVTIYFSDIVGFTAMSA
ESTPLQVVNFLNDLYTVFDRIIRGYDVYKVETIGDAYMVVSGLPIKNGDRHAGEIASMAL
ELLHAVKQHRIAHRPNETLKLRIGMHTGPVVAGVVGLTMPRYCLFGDTVNTASRMESNG
EALKIHISNKCKLALDKLGGGYITEKRGLVNMKGKGDVVTWWLTGANENAIQKKLVDM
MDMPPPLFSRPRKSPKLNPDSRQPSIQAMHFCGTGSRRQSTVPRAMDGESTYSLQGSVRE
SPRMVSKRDRDRERPPINGLGAGHFVGGALLESAQASLSTLNHSETNETNCDMDGGSGG
VSGSGSGLVRQPNALHKPLAMVRPHRIISAAQLPQLGDNDDDSADTLLRESRSLDPMPMQ
QLRKRHDRVKLPPSKLSKNNSRSLDTGVSLISGNPNGEVHSSQLDLDNEMTANPVDATDG
YDDELGLLMRHDNGQLPALRYSGSFPNAQISIVPTGRSAGGGGGGREGGGSNCAKHLNN
NCNGGVNVEDDLESPLLQRQASLSVPPEEMLAHNKRWHSLEHMDGPGGHGGNSVSYAA
DIDNRHPGDLDFFSGSSNQHHRSKAAGGSKLTNWMTNIFKGNGVRSGEARRVGILPSGVH
GARTGFTDMAASAAARDRESIV
>CG42637
MTRWPFNLLLLLSVAVRDCSNHRTVLTVGYLTALTGDLKTRQGLAISGALTMALDEVNKD
PNLLPNVYLDLRWNDTKGDTVLATKAITEMICDGIATIFGPEGPCYVEAIVSQSRNIPMISY
KCAEYRASAIPTFARTEPPDTQVVKSLLALLRYYAWNKFSILYEDVWSPVADLLKDQATKR
NMTINHKQSFIDNRVKCCEQMLDCCRSGYWYQLVQNTMNRTRIYVFLGAANSLVDFMSS
METAGLFARGEYMVIFVDMMVYSEREAEKYLRRVDQITFMSNCHSTENFNQMARSLLVV
ASTPPTKDYIQFTKQVQKYSSKPPFNLEIPRLFVESNFSKFISIYAAYLYDSVKLYAWAVDK
MLREETRVLTDDVIFEVASNGTRVIDTIIKNRTYMSITGSKIKIDQYGDSEGNFSVLAYKPH
KWNNSNNMPCNYHMVPVAYFHQGEEHPEYKLINGSIDWPSGGEKPADEPMCGFANELCK
KDDTHYTSTVAAVVLGVLLFCSGVITMSIYRKWKIELEIEGLLWKIDPNEIKGYSGNEIVSS
PSKVSLMSAQSYGSRWTNQFVTSTGRLRGAVVRIKELKFPRKRDISREIMKEMRLLRELR
HDNINSFIGASVEPTRILLVTDYCAKGSLYDIIENEDIKLDDLFIASLIHDLIKGMIYIHNSQL
VYHGNLKSSNCVVTSRWMLQVTDFGLHELRQCAENESIGEHQHYRNQLWRAPELLRNHI
HGSQKGDVYAFAIIMYEIFSRKGPFGQINFEPKEIVDYVKKLPLKGEDPFRPEVESIIEAESC
PDYVLACIRDCWAEDPEERPEFSVIRNRLKKMRGGKTKNIMDQMMEMMEKYANNLEDI
VTERTRLLCEEKMKTEDLLHRMLPQSVAEKLTMGQGVEPVSYDLVTIYFSDIVGFTAMSA

ESTPLQVVNFLNDLYTVFDRIIRGYDVYKVETIGDAYMVVSGLPIKNGDRHAGEIASMAL
ELLHAVKQHRIAHRPNETLKLRIGMHTGPVVAGVVGLTMPRYCLFGDTVNTASRMESNG
EALKIHISNKCKLALDKLGGGYITEKRGLVNMKGKGDVVTWWLTGANENAIQKKLVDM
MDMPPPLFSRPRKSPKLNPDSRQPSIQAMHFCGTGSRRQSTVPRAMDGESTYSLQGSVRE
SPRMVSKRDRDRERPPINGLGAGHFVGGALLESAQASLSTLNHSETNETNCDMDGGSGG
VSGSGSGLVRQPNALHKPLAMVRPHRIISAAQLPQLGDNDDDSADTLLRESRSLDPMPMQ
QLRKRHDRVKLPPSKLSKNNSRSLDTGVSLISGNPNGEVHSSQLDLDNEMTANPVDATDG
YDDELGLLMRHDNGQLPALRYSGSFPNAQISIVPTGRSAGGGGGGREGGGSNCAKHLNN
NCNGGVNVEDDLESPLLQRQASLSVPPEEMLAHNKRWHSLEHMDGPGGHGGNSVSYAA
DIDNRHPGDLDFFSGSSNQHHRSKAAGGSKLTNWMTNIFKGNGVRSGEARRVGILPSGVH
GARTGFTDMAASAAARDRESIV
>AGAP012163-PA
SGSYITKMPENSPHQKFRNNRTVLTLGYLTAVKGDLIEKQGLTISGALTMALDEINNDPELL
PNVTLALRWNDTRGETVVATRVITEMICDGVAAFFGPEGTCQTEAIVSQSRDIPMISYRCSE
LQRSSPIPTFARTEPPDTQVTKSIISLLTYYGWRKFSIIHEQLWKNVATSLETQAKNNNLSVN
HVEMVFDNYKCCQDDMDCCRSGYWYTVIQKTMNRTRIYVFLGNSNQLVDMMATMDG
MQLFAKGEYLVISADMMTYSPKLSNKYLWRVEKPPNVKNCMDLPGDFERRSKSLLVVVA
SEPLPTFEAFTHKVREYTQKEPFFFKQPSLFHQFVKYVSIYAAYLYDSVKLYAWALDKLLK
EEQQHRPLTSDVIRDVASNGTKIIQTIINNRTYHSVAGATIKIDDYGDSEGNFSVLALKREFY
EEANFSCEFQMRPVAHFQMRQPQHLNDTNQHRNDEIPEFKLSKVGNAIDWPGSDRPMDE
PSCGFMNEHCMKDDTHIMSMVVAGVLALILFCAGVITMSIYRKWKIELEIEGLLWKIDPG
DIKGYFNTEIVSSPSKLSLASAQSFGSRCSNQVFTPTARFRSVVVRIKELKFSRRKDISREIM
KEMRLLRDLRHDNINSFIGACVEPMRILLVTDYCAKGSLYDIIENEDIKLDDLFIASLVHDLI
KAMIYIHSSALNYHGNLKSSNCVVTSRWMLQVTDFGLHDLRHCAENESIGEHQHYRNLF
WKSPELLRQPSVYGTQKGDVYAFAIILFEIIGRRGPFGYTELEPKEIIDRVKALPEPGKDPFR
PDIESVIENENVSDYVINCIRDCWDENADLRPDFPNIRNRLKRMRGGKSKNIMDQMMEM
MEKYANNLEEIVQDRTRLLCEEKRKTEDLLHRMLPQPVAEKLTKGLGVEPVSYDSVTIYFS
DIVGFTAMSAESTPLQVVNFLNDLYTVFDRIIKGYDVYKVETIGDAYMVVSGLPITNGNRH
VGEIASMALELLQAVRSHRIAHRPNETLKLRIGIHTGPVVAGVVGLTMPRYCLFGDTVNTA
SRMESNGEALKIHISQQCKDALDTLGGYVIVERGLIAMKGKGEVMTYWLEAATEQAIQKI
PVDVRDLPPPLFCRPRRSPKMTYDSRHPSIIGLPGGGMITTVSGMGSYMPGSRRQSCAHRV
GGGADHESSYSLQGSMFGPMVPMMRADSSPPPRHRLDACTLDQRPMGPTAPTVTTLGSK
IASSDNLKRALFGTNSPGRRLSHKLRSITSADDYKQLCSSMAANGGRSPEAALLRESRSLD
PFPVTLEPRRKRLDALTKRVPRLAKTIAAPSRTGSQSFVSVPPAVSTISHESIADSTHHHHHH
HHHHKYLHNNNCNGSIGNGAAEEQATCPLLTRQTSLTTPQEHEHHCPGLYTNNSSSKRWY
SLEHVGVPDEDSCSKKSLTRSSLKSWLVGFIHGNGFKSSDSSLRKVGVLPVGVGGVTGFG
ELQPTPEKESMV
>AAEL008390-PA
MTRWPVLLLSLLSVAFAGAPNSDGTFQPSAAGSATILSQPLPPASPMLAAAQTALLGTSNQ
QQQQQQQLSQSLFSLPQAQNPYASGATFIPKLTTSNSRHQKFKNNRTQLVVGYLTALKGS
MKDKQGLAISGALTIALEEINDDPDLLPNMTLALRWYDTRGDTVTATRAITEMICDGVAAI
FGPEGTCKTEAIVSQSRDIPMISYRCSELHRSTPIPTFARTEPPDTQVTKSIVSLLMYYGWK
KFSIIHEEMWKNVAVSLENQAQRNNLTVNHVEEVIDYHKCCENNLDCCRSGYWYTIIQNT
MNRTRIYVFLGNPNALVDMMTQMDAMQLFSKGEYLVIFVDMMTYSLKESKKYLWRLDR

VSHHKNCNDMESFLQRGRSLLVVVASEPHPEFEQFTNKVREYNQKEPFSFRMPVLFKEYA
KYVSIYAAYLYDSVKLYAWALDKLLKAEQAHRPLTSDVIYEVASNGTRIIETIIQNRTYKSIA
GAMIRIDEFGDSEGNFSVLALKNEFYTEGNFSCDFQMKPVAHFQMRQFQQSNDSNQKRS
DEIPEFKLSRAGTAIDWAGSERPMDEPSCGFMNEYCTKDESHVTSMVIAGILGLVLFCACV
IMMSIYRKWKIELEIEGLLWKIDCNEIKGYFNTEIVSSPSKLSLASAQSFGSRCSNQVFTPTA
RFRGVVVRIKELKFSRRKDISREIMKEMRLLRDLRHDNINSFIGACVEPMRILLVTDYCAK
GSLYDIIENEDIKLDELFIASLVHDLIKAMIYIHSSALNYHGNLKSSNCVVTSRWMLQVTDF
GLHDLRHCAENESIGEHQHYRNLFWKSPELLRAEQRTEATYGSQKGDVYAFAIILFEIIGRR
GPFGYSDLEPIRIIELVRAIPEDGQEPFRPDIKSVIENDCIPDYVINCITDCWDENPDSRPDFA
SIRNRLKRMRGGKSKNIMDQMMEMMEKYANNLEEIVQDRTRLLCEEKRKTEDLLHRML
PQPVAEKLTMGLGVEPVSYDSVTIYFSDIVGFTAMSAESTPLQVVNFLNDLYTVFDRIIKGY
DVYKVETIGDAYMVVSGLPITNENRHVGEIASMALELLQAVRSHRISHRPNETLKLRIGIH
TGPVVAGVVGLTMPRYCLFGDTVNTASRMESNGEALKIHISGQCKEALDRLGGYVVVER
GLISMKGKGEVMTYWLEAATDKAIQKQPVDYRDLPPPLFCRPRRSPRLTYDSRHPSIIAIA
NYTAGSRRQSSALKDLESNYSLQGSSFEPSVRDSPRAYQRKLERIPLCINDDSQSTLEQQN
AIGKTHSSDSLVKKVIFNNSSKKVRNMLRSIASTDDYHNICSSGTFLRESRSLDPFPSDVRK
RLESIKLERKPRMGHKGSQSLDAGVSTISSESIDKDQSSKMIITTVPEDYSQGDGHKYLNN
NCNGSIGNNEESHCPLLMRQTSLVTPGQEDSLSTHKRWYSLENVAVPDEDSCSKKSLTRGS
IKSWLVGIIGFKTSDSSLRKVGVLPVGVPGVTGFGELQSTPEKESMV
>CG10738
MFAHPCPAPAGNYHSGLLHPLLLLLFLLAFSNFRPTHGEVFTLGYLTASQRRPGNLDYNRP
GLTISGAISLAVEEVNAGRLRDRGHSLQFVVAETYGEEVVSIRQTAALWTQQVAAYIGPQE
TCVHEGRMAAAFNLPMISYYCTHRDPSNKADFPTFARTRPPDTQISKSVVALLLAFNWTQ
VSFLYLDDASGQYQPVAETILSTLTDAGVSIRDIRTWNTIYHHGFMDNPFEALVEQTYANT
RIYLILGHYYEHVGLMVSLQRRGILSKGDYFVVGIDIEQYDPAKPEKYLRGLLLEDVEPLA
VQAFQSYLGIVPTASVSFATFANEVNKYMERPPFNFPNPLGPFGGVKQISAEAAYLYDAVH
LYAKALMEVLDSGGRPRNGSAIVAAIKGSRYRSAMGYHVYIDENGDAAGNYTVLARGSV
RNGRNQTVLGLRPVGTFIHRNSSLSSISKALPNLKLFSPIDWVGGTRPAAAPRCGFGGEKC
VNYTGEISAAIAGGALLLLSLVSLVLYRNWRYEQELDSLLWKIDFREVQIHENEREQQSQK
QTRSTHPLIRTSQVSLSSNPDADFRYTTIFTPIGLYKGQLYAIKKVRKKSVDITREMKKELKL
LRDARHDNICAFIGACTDPPNICIISEYCTRGSLKDILENEDVKLDNMFIASMVADIIRGVIY
LHDSPIRFHGALCTSNCLVDSRWVVKLTDFGLFAFKQGIEDSSTDMQHMSAKCLKLLYRA
PELLRQGPSSLVMGTQRGDAYSFGILLYEMHVRRGPFGETGLTPMQCLQKVLQPQDYLNP
YRPSLQPLETAFDCVSECLRECWAERPEDRPDFKTIRTKLRPLRKGMRPNIFDNMMAMME
KYANNLEALVDDRTDQLQEEKKKTDALLHEMLPRCVADQLKKGHKVDPEHYEQVSIYFS
DIVGFTAMSAECTPLQVVDFLNDLYTCFDSIIGHYDVYKVETIGDAYMVVSGLPLRNGDL
HAAEIATMSLHLLSAVSEFKIRHRPTNRLLLRIGIHSGPVCAGVVGLKMPRYCLFGDTVNT
ASRMESSGVPLKIHCSWQCRQLLDRLGGYHFAERGVISMKGKGDQRTYWLLGEDEEART
RRTYERSQRRGSRALNKFIQGTIKQAQEQANEYGIRSSLKQKNLPRNSLTRSSSLESPKKLR
FAAGSLLEHHRYHSDEALLEVDSYTGLRRSSGGSTQSRYEETTLSLTLSCQSIEIVGGQHNK
RRPSSYPTANTPLLMNHVEV
>AGAP012161-PA
LQISGAITLAMTEVNDRYFSQHGHELRFEVAETYGEEVTSIRKTADLWTRDVIAYLGPQET
CVHEGRMAAAFNLPMISYFCTHNETSNKKHFPTFARTRPPDLQISKSVVSLLLAYNWTQV

SFLYRASDNGELDAVAETLKTTLRTASIRIRSVGTWTDIYHHGYSSNPFERLVEDTYEDTRI
YLVLGYHYEHIGLLVSLRRRGLLDRGDYFVVGVDIDQYDAALPTKYMHGLLQTTPDPDA
VEAFRHYLGIVPSAPVRFEEFAVKVSWSARDRSRLSRRQRAAVNKYLELPPFNYRNALIFF
GGVKQIRAEAAYLYDAVHLYANALMQVLLSGGSPKNGSAIIEAIKGRAYISAMGYLVHIDE
NGDATGNYTILARKPVPSTGATNQYGLFPIGRFSSPTVDRIPVSGVCSEIRLFDTIDWVGSG
PPVAEPRCGFRGEKCISYTGEITGGIAGGALLLLGVVSLVLYRNWRYEQELDSLLWKVDFR
EIQMHENEKETAGQKMTRVSVCVCRSHAYYSTHPLIRTSQVSLSSNPDLDFRYSTIFTPIGL
YKGQLYAIKKVKKKSIDITREMKKELKLLRDMRHDNLNAFIGACTDPPNICIITDYCNRGS
LKDVLENEDVKLDNMFTASMVADILRGMIYLHDSPLRFHGSLRTSNCLIDSRWVVKLSDF
GLFAFKQGSEEVPDEKEKLEEKCQKLLYRAPELLRAGPTATVPGTPKGDVYSFGIVLYEIFT
RRGPFGEIECTPMECLKRVLNPLDPNTPFRPAIQPLETSFDCVRDCLKECWAERPEDRPDFK
TIRNRLRVLRKGMRPNIFDNMMAMMEKYANNLEQLVDERTDQLQEEKKKTEALLLEMLP
RPVAEQLKRGHKVEAESYDLVTIYFSDIVGFTSMSAESTPLQVVDFLNDLYTCFDSIIGHYD
VYKVETIGDAYMVVSGLPIRNGLIHAAEIASMSLQLLEAVAEFKVRHRPNDRLYLRIGIHSG
PVCAGVVGLKMPRYCLFGDTVNTASRMESTGQPLKIHCSLQTKEILDSLGGYQFQERGLV
PMKGKGDQRTFWLVGEDPDARARRTKERTERRGSRALNKYLGMLKSVTNLPGVRSSLKS
RSLGLPRGSLPRSSSLESPKRLRFASGAMLEQHRYHRDDALMEVISDSSIRRSDYSISDGAE
DITASCPCIEHLGTDPPADPAYLLTNGPTVTTPLLNNSIAT
>AAEL008387-PA
LQISGAITLAMEEVNDIYFSRHGHKLQFEVAETYGEEVTSIRKTADLWTKDVIGYIGPQETC
IHEGRMAAAFNLPMISYFCTHNETSNKKHFPTFARTRPPDLQISKSVVSLLIAYNWTQVTFF
YRTSENSEYDAVAETLKTTLKTNGVRIRSTRTWSTIYHHGYSPNPFDRLVEETFIDTRIYLVL
GHHDEHIGLLVSLKRKGLLTAGEYFVVGVDLEQYDAALPKKYMHGLLQTAPDQDAVPAL
QHYLGVVPSAPVKFEEFAVKVSFSDRVIKLLIPCSSRTLGVNKYLERPPFNYRNPLVYFGGV
KQIRAEAAYLYDAVHLYANALLRVLLAGESPKNGTAIIEAIKGRAYLSAMGYLIHIDENGD
ATGNYTILARKPIPSATSERDYGLFPIGRFSTPNADTIPVSCVVRDIKLFDAIDWVGYGPPVA
EPYCGFQGEKCISYTGEISAAIAGGALLLLGVVSLVLYRNWRYEQELDSLLWKVDFKDIE
MHENEKENAGQKMTRVSENDRAELNPSRNTHPLIRTSQVSLSSNPDTDFRYTTIFTPIGMY
KGQLYAIKKVRKKSIDITREMKKELKMLRDMRHDNLNAFIGACTDPPNICIITEYCTRGSL
KDVLENEDVKLDNMFTASMVADILRGMIYLHDSPLRYHGSLRTSNCLIDSRWVVKLSDFG
LFGFKQGADIQEEKEKAESKCEKLLYRAPELLRAGPVSCVPGSPKGDVYSFGIVLYEIFTR
KGPFGEIECTPAECLKRVLHPLDPNNPFRPAIQPLETSFDCVRECLRECWSEKPDDRPDFKT
IRNKLRVLRKGMKPNIFDNMMAMMEKYATNLEQLVDERTDQLQEEKKKTEALLLEMLPR
PVAEQLKRGHKVEAESYDLVTIYFSDIVGFTSMSAESTPLQVVDFLNDLYTCFDSIIGHYDV
YKVETIGDAYMVVSGLPIRNGLTHAAEIASMSLQLLDAVSEFKIRHRPNDRLYLRIGIHSGP
VCAGVVGLKMPRYCLFGDTVNTASRMESTGLQLKIHCSLQTKEILDSLGGYHFEDRGFIP
MKGKGEQRTFWLVGEDPEARAKRTQERAERRGSRALNKYLGMLKQSNNSNGVRSSLKT
RAVIPRSSLPRSSSLESPKRLRFASGSMLEQHRYHHDDALMEVVSDASTRKSDNSLAETED
FTSSCPCIDNLGLNTERFLAQSCPTVTTPLLTNTVAT
>CG33114 OGC1
MPGPCASAAAFSCILVLLLLGCQRSNPLAAGATVSSMRRLTDTINIGFLAEYSQMRVTLGG
LPLAIEDVNKNPNLLPGKKLAFKPVDIGHKMSAYRVKPLRAMTQMREAGVTAFIGPDESC
TTEALLASAWNTPMLSFKCSDPIVSNKSTFHTFARTLAPASKVSKSVISLLNAFHWNKFSIV
VSSKPIWGSDVARAIQELAEARNFTISHFKYISDYIPTTKTLSQIDKIIEETYATTRIYVFIGEH

IAMVDFVRGLQNRRLLESGDYIVVSVDDEIYDSNRRVNIMERIDICSKIKDYARKTPFLVPY
HQRVFDNISVPIYGLHLYDSVMIYVRAITEVLRLGGDIYDGNLVMSHIFNRSYHSIQGFDV
YIDSNGDAEGNYTVITLQNDVGSGASIGSLAKMSMQPVGFFAYDKNSVIPEFRYIKNDRPI
QWLNGRPPLAEPLCGFHGELCPRKKLDWRYLVSGPLCALVVVVAIALLIKHYRYEQTLAG
LLWKVDMKDVTVINLGEYNNPTNKNIFQICRQSILVVGEPNKRSFTNIALFRGNIVAMKKI
HKKSVDITRSIRKELKLMREVRHENIINFIGASTDHGSVIIFTTYCARGSLEDVLANEDLHL
DHMFISSLVSDILKGMIYLHDSEIISHGNLRSSNCLIDSRWVCQISDFGLHELKAGQEEPNK
SELELKRALCMAPELLRDAYRPGRGSQKGDVYSFGILLYEMIGRKGPWGDTAYSKEEIIQF
VKCPEMLQHGVFRPALTHTHLDIPDYIRKCLCQCWDEDPEVRPDIRLVRMHLKELQAGLK
PNIFDNMLSIMEKYAYNLEGLVQERTNLLYEEKKKTDMLLYQMLPRPVAELLKRGDPVEA
ECFDCVTILFSDIVGFTELCTTSTPFEVVEMLNDWYTCCDSIISNYDVYKVETIGDAYMVV
SGLPLQNGSRHAGEIASLALHLLETVGNLKIRHKPTETVQLRIGVHSGPCAAGVVGQKMP
RYCLFGDTVNTASRMESTGDSMRIHISEATYQLLQVIGSYVCIERGLTSIKGKGDMRTYWL
TKRQQPELTPDLISTVDTLDTYCSGPRESMEVSVHQYCSPASNNYRLGSCNCDTKCLYSRR
SDDNVTNSHGTSEFPKVSEPAQVNCNQLCVCRLNSSQMFNNRGPRSAPSITFRL
>AGAP008848-PA OGC1
LSCRKMTEMRDNGIVVFIGPDETCTSEALVASAWNLPMISYKCADIAVSDKTVYSSFARTL
PPATKVSKSVAALLLANNWHCFSIVASKHPAWSMEIAHAIELQAELNNLTVNHFRIYSDYIP
SKIYELQEIVDNTYRNTRVYVFVGDHIEMVDFVRCLQNRKLLSTGDYIVISIDDEIYDPNM
KRNIYQGNYSDFYQKYIGNSKDKHQQNRKRYNYKDQERLQEAFQSVLRISPLFPMNPKY
RKLCHQFKLYSRKDPFRVPLPYNRHIFDEIQVPIYGAYLYDALIIYARAATEVLRDGGDVSD
GRLIMRHIFNRSYHSIQGFDVYIDANGDAEGNFSVIALQKDDKVNNSLHMSMQPVALFAY
GARNATGTGTTLPEFRYLNPNRPIMWLKGRPPLAEPVCGFYNEKCRPKAKDWRYITGALV
VILFMAIFTIILFKHYRYEQTLACLLWKVDMKDVILITSPDALYNNELRKNLVSRIKYKELQ
INHFQRGEKVCQQSIMVNSAGDVNKRAYTTIGLYRGNIVAIKYLHKRTVDITRNIRKELKQ
MREIRHENLITFVGASIDHGTVSILTSYCARGSLVDVLSNEDLKLDHMFVSSLVSDIVKGLI
YLHDSDVGSHGNLRSSKILIDSRWVAQIADFGLHEFKSCQEEPSKFEKELRRSLWKAPELL
RDPNCPPKGTQKGDVYSFGIVLYEIIGRKGPWGDLNMSWQDIVARVMSPEEYGIFRPSLRG
IDAPEYVIQLLHSCWEEDPEDRPDIRLVRVKLKPMQAGLKPNIFDNMLAIMEKYAYNLEGI
VQERTNQLSEEKKKTESLLLRMLPKSVAESLKRGERVEAECFDCVTIFFSDLVGFTELCAQ
STPFEVVEMLNDLYTCCDFIISSYDVYKVETIGDAYMVVSGLPLRNGDRHAGEIASLALHL
LNSISNLEIRHRPGEFIQMRIGIHSGQCVAGVVGLKMPRYCLFGDTVNTASRMESNGEALK
IHISSITYGLLKKLGGYKCEERGIIKVKGKGEMRTYWLLGEDDQKRMDR
>AAEL005330-PA OGC1
FQVILGALLLAIENINNDSTLLPGKRIKLKPVDIGAQKSLKAFPIRKMTEMRDEGIAAFIGP
DETCTTEALVASAWNIPMISYKCADAAVSDKTVYSSFARTLPPASKVSKSVISLLAAYNWH
CFSIVAGKHPAWSMEIAQAIKVQAEGNNLTVNHFREYSDYIPSKIYKLQNIVDETYRTTRIY
VFVGDYIEMVDFVRCLQNRNLLSSGDYMVISIDDEVYDPSTKRNIYQVTWVQRYSFKDIQ
ERLQEAFLSVLRISPLFPMNPHIKPVPIYAAHLYDAVIIYARAATEVVQAGGNLHDGRQIMR
HILNRSYHSIQGFDVYIDENGDAEGNFTVIALQKDEKVNNSLNKSMQPVGMFVYSSNGTN
LPEFKYLSSQRPIMWVKGRPPLAEPPCGFHGEKCRPITRDWRYITGIMMGTLFITIFAVILFK
HYRYEQTLACLLWKVEMKDVTIMSSPDLMYGSDSKKKLTQVCRQSILANGADTTKRAFT
NIGLYRGNIVAINYLHKRSVDITRTIRKELKQMRKLRHENLITFIGASVDHGVVAILTSYCA
RGSLADVLANEDLSLDHMFVSSLVSDIVKGLIYLHDSDVGSHGNLRSSKILIDSRWVAQIS

DFGLHEFKSGQDEPNKFEKELHRSLWKAPEILRNPNTPSRGTQKGDVYSFGIVLYEIVGMK
GPWGEINLNYQEIIARVISPQNYGIFRPPLRGLEASDYVIQCLQACWEEEPDDRPDIRLVRV
KLKPMQAGLKPNIFDNMLAIMEKYAYNLEGIVQERTNQLTEEKKKTESLLLRMLPRSVAE
SLMRGERVEAENFDCVTIFFSDLVGFTELCAQSNPFEVVEMLNDLYTCCDFIISSYDVYKV
ETIGDAYMVVSGLPIRNGDRHAGEIASLALHLLNSLSNLEIRHRPGEFIQIRIGIHSGQCVAG
VVGLKMPRYCLFGDTVNTASRMESTGDAMKIHISSVTYNLLKKIGGYRFEERGVINVKGK
GDMRTYWL
>AAEL006806-PA OGC2
MLKSKRALVLSVISLLSSWICSVQSENRTSRSYNNADSSHYKYNHSSIFNNISNSSSGSNVIE
HNNPMATNDLHRNPPLWDSSNEKTAYQYAINDNDSNDAPTIASHLHFSSTLSAEHGSIVEK
TVFNGGTFEGSHTPYHKKTVVRSASEDSYHEPHIKFAILLPEHGRSRDSRILSTVRPVIEMA
TNLVTGPNGVLHNLKIEIDYRDTQCSSTYGALGAFDILLKRKPDVFFGPICDYVIAPIARYN
AVWGIPILTTGGLADAFTIKSPNYPTLTRMMGSYSDPGLALREMYRHFNWTIQAFIYHDN
DEKRGMGHSDCSMAILSIFRVLNTTEYFSHSFDETETDYKGYLRILEETKRKARIVIMCASP
STIREIMLAAAELNMVDSGEYVFFNIDIFSSMAATKIPSWHMANDTEERNLKAKNAYTAM
LQVVARQPEDEEYRRFSEEVKLLTKTKFNYTYAEDEPVSTFVTAFYDAVLLYAYALNDSIG
LLGEQRALKQPINGTYLTHLMWGKSFKGITGNVTIDSNGDRISDYSLLDLNPETGMFEIVA
NYFHDGGLQFVEGKEIHWSGGRTKAPPDRPICGFDGSLCPDKSLPGYAILSLILGLCVICM
GIASFMGYRHYKLEAEINSMTWKVQANEVLSCHSSQGHRGSLHMMAKRGSQVTFYSDE
LNSLPGDRQIYIQLGYYKGCKVAIKKININNLNLNRTMMLELKRMKDIQHDHLVRFYGAC
LDPSPDPFILTEYCPKGSLQDILENETIKLDWMFKISLMHDIVKGMAFLHSTELHSHGSLKS
SNCVVDSRFVLKITDFGLHQLRGSLDDQDQESYAYWKKLLWTAPELLRDPHRDPAGTQK
GDVYSFGIIVHEIVSRQGPFYTGDDEKSPKEIIKLVINGPDGYNPPFRPKVDEMYYEDVNNI
MVKCWSEDPMERLDFTVLKTIIRKINKENESGNILDNLLQRMEQYANNLEALVEERTQDY
FEEKRKCEELLYQLLPRSVAAQLIMGKSVIAETYDQVTIYFSDIVGFTSISAQSTPMQVVDL
LNDLYTCFDSIVENFDVYKVETIGDAYMVVSGLPVRNGNLHAREISRLALALLAAVHKFTI
RHRPNEQLKLRIGLHSGPCVAGVVGLKMPRYCLFGDTVNTASRMESNGEPLKIHISHTTKT
LLDTFGTFEVTERGLVPMKGKGEMLTYWLNGERTVPMQTGFKSNKFSDNNTLESGPLAL
LNGTPPTGILNNNNNSNMYSLNSAVGGPNKKLNNVSYNFIKSSSTKNMLNSKGRRSLGGE
DTIRSVTQPLLTQIN
>AGAP003283-PA OGC2
MSARANDDWGRAVVRCCVTLLLYVLASSVPGHERTSLVYGSIDSGTVAAQLATRNSTNY
HLLSSDNGSNSSHPSDPSPLAAVTVTAPRDGNHFVNRSSRDTVGEAPYDDGCDHVRANTG
VVNQHPRIADDTQGNYVKFAILLPKKPSKNRDIRILSTVLPVIEMATRVVTAPGGLLQNLRI
EIDYRDTQCSSTYGALGAFDIFLKRKPDVFFGPICDYVIAPIARYSSVWGIPLITSGGLTEAF
TLKAPHYRTLTRMMGNYHAFGLMMREIHRHYNWTIQAYLYHEFDEKSGRGFTDCSMAIT
SINRAIGGNETSSGTFDEETAKYADYLRLLRNIKKRARIVIMCASPSTIREIMLAAAELNMV
NSGEYVFFNIEIFGSMTATKQPPWYAKNDTDERNQKAKEAFTALLQVVAREPEDEEYRQF
SKEVKELTKTKYNHTYAEDEPVSTFVTAFYDAVLLYAYALNDSIAQLGVERALRQPINGTH
LAQLMWGRSFKGITGNVTIDSNGDRISNYSLLDLNPETGLFEVVANYYYGGGLQFVEGKA
IHWAGDRTKAPPDRPTCGFDGSLCPDNSLPGYAILSLVLGVCVVCMGIASIVGYRHYKLEA
EINSMTWKVNPNDVLSCNPSRGHRGSFHSMVKRGSQATIYSEDLNSLPGDRQIYIHFGFY
KGCKVAIKKINVQNLSLTRSLMLEFKRMKDIQHDHLVRFYGACLDLHPEPFILTEYCPKGS
LQDILENETIKLDWMFKISLMHDIVKGMAFLHSTDLHSHGSLKSSNCVVDSRFVLKVTDF

GLHQLRRSTDDADIESYAYWQKLLWTAPELLRDPQCPPAGSQKGDVYSFGIIIQEIVSRQGP
FYLGTEEKSPKEIIKLVRDGPGILDAPFRPKVDESSYEDVNNIMIKCWSEEPTDRPDFSGLK
TIIRKINKENESGNILDNLLQRMEQYANNLEALVDERTQDYFEEKRKCEELLYQLLPKSVA
AQLIMGKSVIAETYDQVTIYFSDIVGFTSISAQSTPMQVVDLLNDLYTCFDSIVENFDVYK
VETIGDAYMVVSGLPVRNGNLHAREISRMALRLLAAVYKFTIRHRPNEQLRLRIGLHSGPC
VAGVVGLKMPRYCLFGDTVNTASRMESNGEALKIHVSHTTKLLLDTFGTFDLTERGLVPM
KGKGEMLTYWLNGERTEAIAAAPLPNGLKVMSPAGTSPASIMASRNGPTLLATDSVPASD
PQQQPLLLPTATSASQVNGTVASPPAGILINHHNHHHNNNHNNNNNNNNNSPCSINHNHS
NSSPISCHNPSAPAKKLNSVSYSFAKAPGNSSSNNSNGAAAAAAAAASAKASKPKLPEVE
ALRGASVTKPLLT
>CG31183　OGC2
MPQAGQGLSLLLILCLLQAHLFPSAAWFYDAESVGGVASIGVGSDEHSFKTPETKNSHKS
QSTELVQHFPSLPMPDHRRLGARRQLVFVALLPSVESDNKNDCIMPKVLPVLELAIRHVQR
MGFVGGSHFDIQLISRDTFCSSKYGPIGFFEIYTQWPEVNAVFGLPCEYVLAPISRYADVWQ
VPVLTTGGNAKEFNKKSESYSTLTRLKGAQVNNLGNVVRAILNSFNWTRTALIYQNENAK
VKGNSVCFLCLAAIHDTIEEHSVYQLGFDTSTWTKADITRMLKNVAMQSRIVIMCADPQSI
RQIMLTAEELNMIDSGEYVFINIELFSRVQYLTSQPWYDKNDTDLNNERAQKAYTAMLTV
TPKQPNDNEYTRVSNEIKAIAAEKYNYTFSDNEPISAFVTSFFDGVLLYANALNESIREDPT
MLTRPINGTDMVRRMWNRSFTGITGNVTIDANGDRLSAYSLLDMNPTTGRFEIVAHFLHN
RLEFEANKEIHWAGDREEAPPDRPICGYDGALCPDNSLPGYAILSIVLGTMVVVMAVCFFF
GYRHYIAEAEINSMSWKVSLEDVMFRDAAERGLRGSFHSLVKQSSQLTLMSEDMVSING
DRQIFIPVGMFRKSKVAIKPVEVDNVQGLLTRSLMLELKRMKDLQHDHLVKFYGACLDQ
RRSFLLTEYCPKGSLQDILENEQFQLDWMFRLSLMHDIVRGMQFLHSSDIRSHGNLKSSNC
VVDSRFVLKITDFGLHTLRRTRFDLESDGGNCNSHAYWSKLLWTAPELLRVEHNRPPEGT
QKGDVYAFGIIVHEITTRQGPFYLGRCAYEKSPQEIIELVKGYNPHRMQKPFRPELEPNGDT
KADINGIIRRCWAEDPAERPDFNTLKSMIRRFNKDNETGNIVDNLLKRMELYANNLEELVE
ERTQDYHEEKKKCEKLLYQLLPQSVAAQLISGQPVVAETFDQVTIYFSDIVGFTAISAESTP
MQVVQFLNDLYTCFDSIVENFDVYKVETIGDAYMVVSGLPIRNGNQHAREIARLALALLE
AVHNFRIHHRPEDRLKLRIGLHTGACVAGVVGLKMPRYCLFGDTVNTASRMESNGEALKI
HISETTKEALDEFGTFVTTRRGFVPMKGKGEMLTYWLEGEVPRPNSLISPSKLMLTRRSSL
KQPQRSQSHNLHKQYSELVVASPPPQLPPPAIALPPPPSPSKDSPNLRVKRKISSSSPKLNGG
GFDYHKTENHYLDTAAAAQRNCDIYSSRSLRDMEETSDSWELAHFRLPLSGALKSHHHL
NNNSHGYVHSNSSSNLSGILQPPPLGVQRTRLKPSPTISTLLTSARSEANASPGPETGSMGS
GQLRIKFAEGDETPLLPTPPPLAAPPPIPQMVAASPQRSGSGQNIPADSSHYGFLVKSYKQQ
CPPVAGSAVTQPLLAKINS
>AAEL011318　OGC3
GERVAIKKVAKKKVYITSTLLWEIKQARDVSHENTVRFVGACIDLPRPTILILTEYCPKGSL
KDVLENEAIQLDWNFRMSLIHDVVKGMAYLHNSDVGVHGKLRSCNCLIDGRFVLKISDF
GLRTLTTPSEFVRDQNYYNSNLNALDLLPTTVIPGTPATQKGDVYSFAIILEEIVVRGGPYE
TARQVAAHESPPFRPFVGQRDCPPDLLDLMEKCWSDSPDDRPSFIQIKSTVKLIMKGFCEN
LMDDLLRRMEQYANNLESLVEEKTEQLSMEKRRTEELLYQVLPRPVAQQLLAGEMVQPE
QFECVTIYFSDIVGFTALCAQSRPMEVVDFLNDLYSTFDRIIEFYDVYKVETIGDAYMVVS
GLPERNGHDHAREIGLMSLAILDAVKSFTIKHKPEQQLKIRIGIHSGPVCAGVVGQKMPHY
CLFGDTVNTASRMESTGHPLKIHVSEAAKQILDKFGTFRLELRGEVELKGKGICTTFWLLE

CSEPDPRPPTPMKNYIDNDVPFPILFPAIGK
>AGAP008691-PA OGC3
VYHVGVLMASHLDSPFDLERCGPAIDLALELVNQSLMKVHNVRLSKVQRSYATCSGSKSP
GLAADLHFKHSVIAFIGPACAFALEPVAQLADYWNTPIITGMGDQPPSEGELSVTSGILGRL
SNRWKNDSSGMFKDKSRYQTLTRMSYCQCRLKLVFSSIFRQFGWRHIALIIDRSDLFSLTV
GKNLEYGLKDEELLKFVRELDGNDEEDIEAYLKDASMYARVIILSVRGSLVRKFMLSALA
LGMTRGEFTFLDVEIFQSSYWGDHYWELGDEDDFKARKSYEALLRVSLLQPTSPTYQYFA
EKVRALAKQDYNYTFVEDEEVNFFIGAFFDGVYLLGMALNDTLNEGGDIRDGTAITRKM
WGRDFEGITGHVRIDDNGDRDADYSILDLDPITGRFEVVAHYYGITREYSPVKGKKIHWP
GGREGPPPDVPKCGFLGTSPACQGNDMIIRYGLVGFGIISAFAAAVTYILCKQMKLNSELN
NMSWRVRPDEVLLEVGKMFGSKMGLQKLNYENFSLQQFGLNSGRVSIASGNSQLPAQLF
TTIGIYKGERVAIKKVAKKKVYITSTLLWEIKQARDVSHENTVRFVGACIDLPRPTILILTEY
CPKGSLKDVLENEAIQLDWNFRMSLIHDVVKGMAYLHNSDVGVHGKLRSCNCLIDGRFV
LKISDFGLRTLTTPSEYLLPATVIPGTPATQKGDVYSFAIILEEIVVRGGPYETARQFLDPQAI
VERVALHESPPFRPFVGQRDCPPDLLDLMEKCWSDSPDDRPTFSGIRSSVRLIMKGFCENL
MDDLLRRMEQYANNLESLVEEKTEQLSMEKRRTEELLYQVLPRPVAQQLLAGEMVQPEQ
FECVTIYFSDIVGFTALCAQSRPMEVVDFLNDLYSTFDRIIGFYDVYKVETIGDAYMVVSG
LPERNGHDHAREIGLMALAILDAVRSFTIKHKPEYQLKIRIGIHSGPVCAGVVGQKMPHYC
LFGDTVNTASRMESTGHPLKIHVSEAAKQILDKFGTFRTELRGDVELKGKGIVTTYWLLE
CSEPDPR
>CG3216 OGC3
MHLLGISIHFFFLMYVNCFSAHPNPRRNDITWDDLNKDISLDSTTSLAGLNASDAGLEQR
MYERSRESKSTQLSRYTEVGEMGSTMRVYNVGVLMASHLDSPFDLERCGPAVDLALDEI
NKVFLKPHNITLLKKKGSYPSCSGARAPGLAADMYFQDDVIAFIGPACAFALEPVARLAA
YWNKPIITGMGDQPPSSEGELTVTSGILGRIHKWKNENTGMFKDKSKYPTLTRMSYCQCR
LILVFASVIRQFNWNHVALLVDRSELFSWTVGKNLEYGLRQEGLLSFVKELNGNEEEVYE
NYLKDASMYARVVILSVRGVLVRKFMLAAHSLGMTNGEWVFLDVEIFQSEYWGDKGWE
MKDEHDAKARKAYEALLRVSLLQPTSPKFQDFADNVRENALYDYNYTFGEGEEVNFFIG
AFYDGVYLLGMALNETLTEGGDIRDGVNITRRMWNRTFEGITGHVRIDDNGDRDADYSIL
DLDPINGKFSVVAHYSGVHKQMKLSKELNNMSWRVRPDDVLIEMGGMFGSKGGLQRLD
VENISLQQFGIHSGRASIASFTSLPPQVYTTIGQFKGERVAIKKVNVKKVDLTPQLLWEIKQ
ARDVSHENTVRFVGACIDLPRPTVLILTEYCSRGSLKDVLENEAIELDWNFRMSLIHDIVK
GMNYLHNSDVAAHGKLRSCNCLIDGRFVLKISDFGLRTLTTPSDFVRDQNYYLKLLWIAP
ELLPLTTIPGCCPATQRGDVYSFGIILEEIVNRGGPYQEARQQMDVHTILHKVRQCNGFRPL
IRERECPPDLLELMEKCWADNQEERPTFSTIRSNIRTIMKGFCENLMDDLLNRMEQYANNL
ESLVEEKTRQLSLEKQRTEELLYQILPRPVAQQLMAGDLVEPEEFSSVTIYFSDIVGFTELCA
RSSPMDVVNFLNDLYSTFDRIIGFYDVYKVETIGDAYLVVSGLPEPNGDKHAREIALMALD
ILRAVSSFNLRHKPEYKIQIRIGMHSGSVCAGVVGKKMPHYCLFGDTVNTASRMESTGQP
GKIHVSSATKAILDKFGTFQMEQRGDVELKGKGTVTTYWLNSTSEGEARPPTPQILTTDEV
PFPLLFAGMGK
>AAEL007359-PA OGC4
MAHRLFNIPMEKEEFVEERMKIHEAAAVNGYDEEFVNKILRKHERKKHRQIATTLQPHKE
EPLRISLPFYPKLTNPIQGILKQYGMQAAYKSGHTLKENYLLKNRLSKGPNKIVLSPSDFVF
PVDMRRVDEGIEAMLCCWLQQLQEFGGPEVEKPDLLKGSIGSLKNLGLPPPPKTGSGSDT

LVRHNTTVAGALELKARYNGDLVQLKEIPPSSGSSSSQELKTKAMDLLVMAHGLRHENIN
PLIGWLNEPTRTALVYEHCSRGSLQDVLIMDEIKLDWSFRLSLLTDLVRGMRYLHGSPIRV
HGTLSSRNCVVDARWVLKITDYGMLNFYDAQGITPPSKSAKDLLWTAPEALRATKGYPK
GGTQAADVYAFGIIMQEVVVRGEPFCMLSLSPEEIIAKIKKPPPLIRPSVSKGAAPPEAINIM
RQCWAESPEMRPDFVMICERFKQLNHGRKVNFVDTMFQMLEKYSNNLEELIRERTEQLD
MERKKTEQLLNRMLPSSVAERLKLGLAVEPEEFSEVTIYFSDIVGFTTISAHCTPVQVVDLL
NDLYTCFDATINAYNVYKVETIGDAYMVVGGLPVRTPDHAEQIATMALDLLHQSGNFKV
RHLPGVPLQLRIGLHTGPCCAGVVGLTMPRYCLFGDTVNTASRMESTGSSWRIHMSQQTC
NLLEQAGGYIIEPRGPIEIKGKGKMHTYWLLGKKGFDKVLPTPPPIGLDIAILRKSLFQSEQ
HNQQQFVVASGSHNGSTANHSSSHSPSVAGESIDVKVEITPPVGCDNPHLAQSFSVDSNSS
NANNCTLNMTDFQSKTALPSPQARKLSEIVTDTAFLSANSSFNRLNPSPTGTTSTRLIKRIE
ELMDLSSPYNYYKCLSPSESNLSQCMDNRYHGYQINRLDCSSKPGSTRFLRRQFSLDKDD
VSPSMHTSQKATLDTVSSISIDREQLARMGTLSSIPSISSTTSISLGSKQQRAIHKQQSASVA
QDLEKIEEIPISPQSFLLNHTTTSSNSTSSLTSDVNDKTRTRSSIQNGKELCLSIEALGLR
>AAEL012988-PA OGC4
MDEIKLDWSFRLSLLTDLVRGMRYLHGSPIRVHGTLSSRNCVVDARWVLKITDYGMLNF
YDAQGITPPSKSAKDLLWTAPEALRATKGYPKGGTQAADVYAFGIIMQEVVVRGEPFCML
SLSPEEIIAKIKKPPPLIRPSVSKGAAPPEAINIMRQCWAESPEMRPDFVMICERFKQLNHGR
KVNFVDTMFQMLEKYSNNLEELIRERTEQLDMERKKTEQLLNRMLPSSVAERLKLGLAV
EPEEFSEVTIYFSDIVGFTTISAHCTPVQVVDLLNDLYTCFDATINAYNVYKVETIGDAYMV
VGGLPVRTPDHAEQIATMALDLLHQSGNFKVRHLPGVPLQLRIGLHTGPCCAGVVGLTMP
RYCLFGDTVNTASRMESTGSSWRIHMSQQTCNLLEQAGGYIIEPRGPIEIKGKGKMHTYW
LLGKKGFDKVLPTPPPIGLDIAILRKSLFQSEQHNQQQFGVASGSHNGSTANHSSSHSPSVA
GESIDVKVEITPPVGCDNPHLAQSFSVDSNSSNANNCTLNMTDFQSKTALPSPQARKLSEI
VTDTAFLSANSSFNRLNPSPTGTTSTRLIKRIEELMDLSSPYNYYKCLSPSESNLSQCMDNR
YHGYQINRLDCSSKPGSTRFLRRQFSLDKDDVSPSMHTSQKATLDTVSSMSIDREQLARM
GTLSSIPSISSTTSISLGSKQQRAIHKQQSASVAQDLEKIEEIPISPQSFLLNHTTTSSNSTSSLT
SDVNDKTRTRSSIQNGKELCLSIEALGLR
>AGAP002233-PA OGC4
MSSLRNYIIKTRLSKGPNKIVLSPSDFVFPVDMRRVDEGIEAMLCCWLQQLQEFGGPEVEK
PDLLKGSIGSLKNLGLPAPTKPSSGSETLIRHSTAAIDHKARYNVSILSFPVSGDLVQLKEIPS
SSASHELKTKAMDLLVMAHGLRHENINPLIGWLNEPSRTALVFEHCSRGSLQDVLIMDEIK
LDWSFRLSLLTDLVRGMRYLHASPLRVHGSLSSRNCVVDARWVLKITDYGMLSFYEAQGI
APAPRNAKELLWTAPEALRDSRTYPKAGTQPADVYAFGIIMQEVVVRGEPYCMLSLTPDEI
IAKIKKPPPLIRPSVSKGAAPPEAINIMRQCWAENPEMRPDFATICERFKQLNHGRKVNFVD
TMFQMLEKYSNNLEELIRERTELLDIERKKTEQLLNRMLPSSVAEKLKLGLAVEPEEFAEV
TIYFSDIVGFTTIAAHCTPVQVVDLLNDLYTCFDATINAYNVYKVETIGDAYMVVSGLPVR
TPDHAEQIATMALDLLSQSGHFKVRHLPGVPLQLRIGLHTGPCCAGVVGLTMPRYCLFGD
TVNTASRMESTGSSWRIHMSQQTCNLLEKAGGYVIEPRGPIEIKGKGKMHTYWLLGKKG
FDKALPPPPPIGLDVAILKHSLFQSQQDHGAKINNASIQSNTCSTANHSSSHSPSVAGESIDV
KVEITPPVPPSLDPGQQQQQPGQDTAAVLSTSFSIESSSSNNTFTLNLGEFGQPKASPLPSPQ
ARKLSEVIADGSFLSAGATFDRLNPSPSSASASSTRLFKRIEELIDLSSPYNYYKCLSPSENN
LSQCTESRYGSYQLRSDSCSSKPGSTRFLRRQFSLDKDDVGPSSGGHGVATLSAKATLDTIS
SISVDREQLARMGTLTSIPSISSTNSATLPGGGQKQRSLAMHRQQSASVAQDLEKIEEIPLSP

QSFIINHTGGGGGGGGGGSSSTSSLQSEVNERGRSFRNGASTAPNGHSGKELRLSVEALGL
R
>CG34357 OGC4
MKLTTCQIAKVFPLLTAFFFLNSPSAASLSFGLSPTFASASASSSLSSSSSSSSHFHSEKPLHS
DVLVLRAAKPSRAIATSADAATRSVPASAAETTLGSKSIWPTAIQTKSPRNAREAGRHKRQ
MQKPAIQQQPLQQQQQRINPPQQKRQQQQQQQQQVQHQAQRVGVLIPPSLHVDLTHVQQ
GFQNFLDFFQLHLFNVTVDFLRDVDLSGFIKLLELPKYTSVIKTLNAGIMVASHVDVDQNS
TIYIGTPSQPRQQHRLHSKQEQEPHRHHHEDSKAKQFPAGEEESHSSGETEENHWNDARIF
SGHCHQLAEQLALDFNKTVVLWPCPRMKISSNFLPSFEAISLAVQSISTKLNWSQVDIYIGD
DNWGLGLAIAANLHVPYRIEIGRTIRDLHSQDKPGKAIIITAPLNDASTILLLSTIELDWTEQ
ARKDGQGSRPGRQHTKILLIDMAASSLDTQHGFYKYLSRMGGSQSDAFARNTGSPAEVV
SSNLLVLTLLNDRHRLFLNAAGLMATMQNFRAPLNGNYKDPPPSGHRNASLHKLYPYRN
LLPLYDTIVTTTTQSDTSETSPQFDFVVLDIVRDAPVATYKWRPLLILESDPGSRGGGSVVE
GGGSYISHSIHPGYDEWLLVSSVLLWQCGAICWTIAAICVCLLVIMIAGIVAGGIAMRNYFL
RKRLSKGPNKIVLSASDFVFPVDSRRVDEGIEAMLCCWLQQLQEFGGPEVDKPDLLKGSI
GSLKNLGFVIPGAAAPGSAGAITTTANGKSGSSATGSLARHNPAHLDMRARYNGDLVQLK
EVNINGSAELRTKAMDLLVMAHGLRHENINPLIGWLSDPNRTAMVFDYCSRGSLQDVLIM
DEIKLDWSFRLSLLTDLVRGMRYLHTSPLRVHGALTSRNCVVDARWVLKITDYGLNSFYE
SQGLPPRTRSAKELLWTAPELLRNMKLHQHHHQHGRIQLGTQLGDVYSFGIIMQEVVVRG
EPYCMLSLSPEEIIVKIKKPPPLIRPSVSKGAAPPEAINIMRQCWAEQPDMRPDFNSVYERF
KMLNHGRKVNFVDTMFQMLEKYSNNLEELIRERTEQLDIERKKTEQLLNRMLPSSVAEKL
KMGLAVDPEEFSDVTIYFSDIVGFTTIAAHCSPVQVVDLLNDLYTIFDATINAYNVYKVETI
GDAYMVVSGLPVKIPDHAEQIATMALDLLHQSGRFNVKHLPGVPLQLRIGLHTGPCCAGV
VGLTMPRYCLFGDTVNTASRMESTGSSWRIHMSQETRDRLDARGGYAIEPRGLIDIKGKG
MMNTFWLLGKKGFDKPLPAPPPIGESHGLDESLIRNSITLKAQANKSRTSTNPSSSQSSSLA
GESVEVKVEITPPTNADLASGTNLPNSYSLDSNSTNTISPNATLCPEFPGKTTPSSTSPQSRK
LSELTPENLLNPNSFNRLPSSTGGSSSRLYKKIEEMMDLSSPYNHYKCLSPSESNLTQFYDG
KYLYGSVAGGAGGGQAGGGACASIALSGSGCGGGASRFDSKPGSSRLLRRQFSLDRDDQ
QAKGEQQHHQHSLQANTYSGLVGGCGGGVKSSMLDIPLLHDTTRSPKGTLTRSHKQNSA
SITQDLEKIEEIPLSPASSQHHSSLDSNLNRSPPSTLEAQSPPLPPMSPPQLSAPTSPAPSRTLN
GIYAHNSNSTSSHGAANNNNGTHPGPELTLNAEQLLSR
>DmCG11144
MKQKNNNGTILVVVMVLSWSRVVDLKSPSNTHTQDSVSVSLPGDIILGGLFPVHEKGEGA
PCGPKVYNRGVQRLEAMLYAIDRVNNDPNILPGITIGVHILDTCSRDTYALNQSLQFVRAS
LNNLDTSGYECADGSSPQLRKNASSGPVFGVIGGSYSSVSLQVANLLRLFHIPQVSPASTA
KTLSDKTRFDLFARTVPPDTFQSVALVDILKNFNWSYVSTIHSEGSYGEYGIEALHKEATER
NVCIAVAEKVPSAADDKVFDSIISKLQKKPNARGVVLFTRAEDARRILQAAKRANLSQPFH
WIASDGWGKQQKLLEGLEDIAEGAITVELQSEIIADFDRYMMQLTPETNQRNPWFAEYWE
DTFNCVLTSLSVKPDTSNSANSTDNKIGVKAKTECDDSYRLSEKVGYEQESKTQFVVDAV
YAFAYALHNLHNDRCNTQSDQTTETRKHLQSESVWYRKISTDTKSQACPDMANYDGKEF
YNNYLLNVSFIDLAGSEVKFDRQGDGLARYDILNYQRQENSSGYQYKVIGKWFNGLQLN
SETVVWNKETEQPTSACSLPCEVGMIKKQQGDTCCWICDSCESFEYVYDEFTCKDCGPGL
WPYADKLSCYALDIQYMKWNSLFALIPMAIAIFGIALTSIVIVLFAKNHDTPLVRASGRELS
YTLLFGILVCYCNTFALIAKPTIGSCVLQRFGIGVGFSIIYSALLTKTNRISRIFHSASKSAQR

LKYISPQSQVVITTSLIAIQVLITMIWMVVEPPGTRFYYPDRREVILKCKIQDMSFLFSQLYN
MILITICTIYAIKTRKIPENFNESKFIGFTMYTTCIIWLAFVPIYFGTGNSYEVQTTTLCISISLS
ASVALVCLYSPKVYILVFHPDKNVRKLTMNSTVYRRSAAAVAQGAPTSSGYSRTHAPGTS
ALTGGAVGTNASSSTLPTQNSPHLDEASAQTNVAHKTNGEFLPEVGERVEPICHIVNK

RTKs

>KAF7989857.1 hypothetical protein HCN44_008531 [*Aphidius gifuensis*]

MLSSSPASSSSSNCTVTSCLLCERLHGESTIPRPFNNNKKTNNKNNNSCCDNNQRSRINENI
FFDDNSNHQSITRTMRQCISNNLHNKKSRGSVRCASSVVRWLTIINNLKINKLLLSFFYFIL
LTNIITKTEAVDRSFRSLIPPTPLPINLTNSEDIQPNTKPKVCQSIDVRNSVTQLDVLKGCTVV
EGFVQILLIDNAHESQFENYTFPNLIEITGYLALYRVSGLRSIGHLFPNLTVIRGSTLFINYAL
VVFEMQNLQEIGLHSLTDVLKGSVRFEKNPQLCYVDTIDWNAITRSESDNIISGNKPKNGC
PVCERIKGCPSKKEKKSEYLCWNAQHCQKVCERQCGNLPCSPSGECCHSECIGGCSDLSP
NTCYSCKNYLSSNGTTCVSECDANSYLYMERRCISKDECKSMKKPLEALPDIAEFPYKPFR
NICDINCPVGYTENKTEICTQCSGQCQKKCPSINVDSVGKIQSLRGCTIIEGSLEIQIRGGKN
IVKELEDCLSSIEVINGYLKIVRSFPIISLNFLRNLKEIKGELLENNKYILSVLDNQNLQELW
NWQIRPPIKLGNANDTIRPKIFFHFNPKLCLQNIRDLRDKSNLSDFTDLEVAPNSNGDKVAC
NVTELYTIVTKRTSEAALIQWNAFEHHDPRSLLGYVVYFIEAPNRDVEMYDGRDACGND
GWHVDDVPNSSSEPYLIHILTQLKPYTQYAFYVKTYTISTERSGAQSKIQYFTTLPGIPSSPR
ALTTWSNSSNEMVITWLAPLRSNGNLTHYKIVGRLELDDPSFLAKRNYCDEPMSPEKKSIL
LLADEERKRAEAEKYIPKQTDTSCDCTDHEAKQSMREKEVASSIAFEDALHNQVYVKRV
HNRRRKRFVENDIGNDGDHHHHNHKIESNIMHNTRCRRDVPSNLMINQTTTTTNVVDKIE
NGTYTVFTREIPANNFTFVMKQLRHYGAYNIELSACREAVVNESLPTCSPKGMRTYRTLPL
ESADDIPNGTFKFTLLGGNNSQQIVKLEWDEPVQPNGQIITYQIVYKRIDIENAQPTTVCIT
RYDFIISSKSYSLKDLPAGKYSIKIKATSLAGSGNYTLSQIFYINEVSTHNYILITVFSVIVILFI
IFISGAYIIKRKYMPNVPSMRLIATVNPEYVSSLYVPDEWEMPRNKIELVRELGNGSFGMV
WEGLAKDIDSLPDMKCAVKTVNETATDQERIDFLNEASVMKTFKTHHVVKLLGVVSIGQ
PTLVIMELMQYGDLKGYLRSHRADEADCTSEPPPTFHRILRMAVEIADGMAYLSAKKFVH
RDLAARNCMVAADLTCKVGDFGMTRDIYETDYYRKGSKGLLPVRWMSPESLKDGVYTI
NSDVWSYGVVLWEIVTLASQPYQGLSNDQVLKYVIEGGVMEKPENCPERLYYIMRKTW
DQRPSRRVTFCSIVSMLLSYENPDYEGFDKVSFYHCNDGIEARKANKLQQQQQQQLQQ
QQQQQQQLQPSIRR

>KAF7992320.1 hypothetical protein HCN44_001645 [*Aphidius gifuensis*]

MRTMQKLMLMSTMTIFIIICQQLLIVHGQDNYQKFNQHIHKIQNYHQKKLSDNNYDPGIY
QDDDKNIYENRKKRAIQNSSNDNESVGTSGQSLFEYDSGKKSSTKKKKDDEICNNMDIRN
DVKFFDSLYGCRIIEGFLQIVLIENNDSSAFDNITFPKLREITGYLLLYRVSNLKSIGNMFPNL
EIIHGHTLLADYSFMVYEMQNLQEIGLSKLKRISRGGVRIEKNTALCYTNTVDWSSIVVAG
ENFIQNNIMLAEKADSCPSCAHCPGGYCWTSNKCQKTDQKSTCHPQCLGECFGPNDTDC
YTCKNYEIDKKCVEKCPDNLYTYLSRRCLTKEECLEINNNNNGIRTDEDKKWRPFKKSCIS
QCPDRWEDAINENNEKTCRECVGNCRKIIYGAIIRHISDAQKFRGTTVVNGALEFQIRNGN
PNIINELTEAFGTIEEITEYLKVTHSSSITTLSFFKKLKLIKGTKLDINNASLVVLDNPNLSSLF
SDNQTIEIRAGRFFFHYNPKLCLSKIQALGYKSKMGKFTEMEVQPESNGEKVACDIVNIKII
TNNTMSNSTDIVWDVYQPSEGQSLLSYLLNYIQTDSPTISYEVNSCGNNNTWNVIDIDISK
QHGTLNKITKKIDNLKPWTRYAVYVKTLSTRNNNSLPSPTGQSEIIYFRTKPSRPSPPINVISF
SPNSNEIVIQWEPPLVPNGPIGSYLIQGAYLDEDVDTILKRDYCLYEPSDRESDMLYDNLPL
TKLPLLLPSSSSSSSTTSDNNNNNNNNKENCCKDTNQPSNIVISKSMDISCFKNGTLATTSI
GGTEYCGKKENFYINPWLKNSSINNKKIIDNKSIRKTFKKRESIKLLERIIIDNVSSTHSSTIIS
NLKHSSTYVFSITACGVQYNNNNNNDDDEYNMCSMWQYTHAKTKKIIDADNINNIDTKV
ISDTHTIVYWTKPIDPNGIIVAYVIEYINKNIEKAKKLTACISGNVTQYNLYHLNPGTYYIRV

KSMSIGGHGKWSRQHEFTTGPKVNNYEFLIGIFALIVFTICFILSYKLYESYRKKKLTERLIA
TVNPDYIETQYLRDHWEIDRSKIKITGNIGMGHFGMVYHGILNDNIKVAIKTIPEPCTEKSK
NLFLNEASVMKKFSTYHLVKLIGVVSDGTPPYVVMELMEQGDLKNYLRRVRQTPKRPNK
LRLVRMAGEISDGMAYLEAHKYVHRDLAARNCMISQNLVVKIGDFGLARDIYETDYYKV
GNDKSLPIRWMSPENLSDGVFSSYSDVWSFGIVIYELQSMAALPYQGMSHEEVMSYILRH
GKIQPCLIEDNPCEVLESIQERCFSWNPTDRPSFVEIVAELESYLEQDFCDKSFYHSEEGVK
VRNAGIKKFYLPPAQVRFHWEFDDVQIGVVISPVMSNNMIREIGIYWKNPNIKIGDSVGLY
NKDPNFNDALPIFKFTPDIPHGFRKTGISADFIPTSKLSFSKKCLGYYVAWIRDDVVKKTNC
LSTNPSWMGDRRKTLEHLKMRDIFLPGTHDSAAYAKHKNSQLELIVDKYTITQDEDVLAQ
LIYGARYLDIRIGRYPNHEHTFWGNHGPFRIVPLKTVINAVKYFLDKTDEIIIFDVQEFPVGF
KTMAAHYELTDYLEKEFAHYLLPRPANGWKITLEDIWNSNRRLIIGYDYASVVASRTCVW
PQVGHQWGNVRTIGALYKHLNKVETQAAEESFNPNPSSQPRAAMAQLTPTTFDVIFDRLG
GLRKMAENVDINITEWYNEEWQHTANVVAVDFLKATGIVETAIEWNDKRAAGC

>KAF7993905.1 hypothetical protein HCN44_011174 [*Aphidius gifuensis*]

MELTLQRGDKLIKILLLFSNLFILCSATQIHDHMTDEEILKTFQVTRDKVPEHEVVSVDYHS
RNNDDTHRIKIRSITKDIDLYLRPNEGLFISETTPVWLVQPHSLYSERLSYTKLEKNLHCRD
TDLLSLHHSDGIFIIQQIVQGIMSIFSGLKPGQFYRYRVKRVENNSVSKPELTGWFQTIKNDE
KPEGIREINVQDIVPISDDIHHLQAQVTFEPGQDLSCYYDVLNWSEKQGLSIGHVNATQYF
KFQLNKLDYYCNNTITILATNKGTKEKSNNVSMIIKTPSCLESHKSLIICAPNKVSGLRVKK
LQQFDDNIKFQVSWDKPKFLPDNYTVLIEPFDHSKKQITINLSGNLTEFFYYLDEIGLPHVIS
IVAESHGGISQPISIMNTIKFEKRKQTIINDILIILSSFGMVILAGVIYIYYKNKKTTIKNQYNY
CSNINDKVNLAEIYEKSKIIESDIKMEIDPLLSKDEFEIHPVQLRIGKILGSGVSGIVRLGSLE
VNNNDWIQVAVKMLQDGPSEDDVKNFKQEILIMKSAGIHRNIVSMIGCCTTSIKPMLIVEY
CSQGDLQTYLRNIWNNLLNDANNRKIQIENIKLCHEKNSDIISSMSCEYENFKRSEQTVAIS
NQLYDLQQGMLNTKEEISASDLLSFAHQVAIGMEFLSMNRIVHRDLAARNVLVCSDKTVK
ISDFGLSRDVYQENIYRKQGNGKLPIKWMAIEALTHQIYTTKSDVWSYGILLWEIVTLGCS
PYPDISTSIVLELLKEGYRMPRPQNCGIELYEIMRSCWNTRPRCRPTFTKLKQDMDKLLET
VSQNDYLNLCNLQQERDDSITDSE

>KAF7994006.1 hypothetical protein HCN44_011275 [*Aphidius gifuensis*]

MWKYRATIGLLIILSIFQSTNSNRCLTMLDETGPKRSIYYNNQILDILLELNERPTQRIVTKIT
KIYLTEVLGYTNIKFINYKDDFNIDSSLRRLSDIYTIQKNKQKIPYSMINMEVWVPPQFNKIS
ELNNYDIKSCGSLVSSPGRFGWFIPKILSDSNDNSYVFNNHEKASKFDINNTTFDRIKNLTIN
KITNDFFCQSSKCNNGIYIPKQCELKTCGLLLASNYEATHNVIKQIDDNELYVKVAWFGKN
IGKVIKILMPEYEKYNNDNEKKKSFVILHWTPSDIITNENEFIQIKFSQCQNDIKYDNYNCL
YDKNKLSKIVWSKLETIGTHLINVIKNINFNSSIYDVLKIDNVNNNSEDDIACNWMINNLN
YTINNWTPSDVNKEKIYVGGIFPMTSKLFSDNTIVIGARMAKEKINSNSTLLMNYNIELVA
RDGHCKSDTVMKSFIDYIIYNSYDKFIGLLGPGCSDTIEPLVGVSKYYNTAIISYGAEGSSF
HDRKRYPYFFRTIPENKQYGHVYVKLFQKFKWQRVAALSEDGQIYSEHISYMQDIFRNNG
IHFIATTKYSKNSENFNITMYLEDLKTNRARIILADINNEVIVRQIMCEAYHLEMTMYQGY
VWFLPLWIQKDWYSDDENYDFSRNTSCTIAQMNEAVNGHFSVSHEFFASDNTIMQEGKT
VRQWRDEYEHQVKQSSNNIGKPSNYAGYAYDAMWTYAYAVDRLIKENNGYISDYHTEPI
VKQLTSIIAETDFQGVSGRIKFYGGASRFSDSIIVQRINGKINNIGNFYPNISEITSDVIDGKLV
LNESAIIWLSNVIPNDGSVAPIVCVLPGLSSLLNVSCESAIIIACIILFIIITSGGIILFIMFKRKY
DKEVRMKEKAMVTMDMVMRRPVLDKKWEIPRSCTSINRIIGEGEFGRVYGGLVDLTDGT

GCRDAAIKVLKEGSSSSSQMDFFSELEYMKLFNHKNIVKLLGVLTVSSPEYIILEFMLYGD
LKNYLLSRRGLVYDDNYNNDDKNEVSPMSLTGFALDIARALSYLAELKFIHRDIAARNCLI
SSKRVAKLADFGLARPMYDSDYYKILKTRSLPVRWMSPESLKTGNSTPASDIWAYGILLWE
IITFGNFPYSKIRTEDVVKYVTNGNTLDVPKNAKPELKNLIKSCWSYNAKDRKRAPYIVDI
LARNPNIVTPCIDSVTTNIPLVNTNTLDFPLPEKDFSMNLQWGAVPLCSTASQAPSTASTSS
VASTTLTQLQSYAAFNSRDIHNNSAILDMNYSDNYNSDIYELQNNELHESAKPLLNNLTNN
VLKIQQCVNGTIRNNIPSYVNCQDNNNINNNLFVKNNDINNTT

Other insects:

>DmCG11144
MKQKNNNGTILVVVMVLSWSRVVDLKSPSNTHTQDSVSVSLPGDIILGGLFPVHEKGEGA
PCGPKVYNRGVQRLEAMLYAIDRVNNDPNILPGITIGVHILDTCSRDTYALNQSLQFVRAS
LNNLDTSGYECADGSSPQLRKNASSGPVFGVIGGSYSSVSLQVANLLRLFHIPQVSPASTA
KTLSDKTRFDLFARTVPPDTFQSVALVDILKNFNWSYVSTIHSEGSYGEYGIEALHKEATER
NVCIAVAEKVPSAADDKVFDSIISKLQKKPNARGVVLFTRAEDARRILQAAKRANLSQPFH
WIASDGWGKQQKLLEGLEDIAEGAITVELQSEIIADFDRYMMQLTPETNQRNPWFAEYWE
DTFNCVLTSLSVKPDTSNSANSTDNKIGVKAKTECDDSYRLSEKVGYEQESKTQFVVDAV
YAFAYALHNLHNDRCNTQSDQTTETRKHLQSESVWYRKISTDTKSQACPDMANYDGKEF
YNNYLLNVSFIDLAGSEVKFDRQGDGLARYDILNYQRQENSSGYQYKVIGKWFNGLQLN
SETVVWNKETEQPTSACSLPCEVGMIKKQQGDTCCWICDSCESFEYVYDEFTCKDCGPGL
WPYADKLSCYALDIQYMKWNSLFALIPMAIAIFGIALTSIVIVLFAKNHDTPLVRASGRELS
YTLLFGILVCYCNTFALIAKPTIGSCVLQRFGIGVGFSIIYSALLTKTNRISRIFHSASKSAQR
LKYISPQSQVVITTSLIAIQVLITMIWMVVEPPGTRFYYPDRREVILKCKIQDMSFLFSQLYN
MILITICTIYAIKTRKIPENFNESKFIGFTMYTTCIIWLAFVPIYFGTGNSYEVQTTTLCISISLS
ASVALVCLYSPKVYILVFHPDKNVRKLTMNSTVYRRSAAAVAQGAPTSSGYSRTHAPGTS
ALTGGAVGTNASSSTLPTQNSPHLDEASAQTNVAHKTNGEFLPEVGERVEPICHIVNK

>Hheb037010.1
MRREMCQSIDIRNRVDQFSKLRGCRVVEGFVQILLIDHANETAYINQSFPELVEITGYLVLY
RVSGLKSIGRLFPNLRVIRGHSLFINYALVAFEMMHLQEIGLHSLTDILRGSGNKPQNGCPV
CDKKCPMRSMTHLKRYEERLCWNQEHCQLVCGTSSCLAKNTSSCCHESCLGSCEGTSAG
HCLVCKDVSVDNMCVNKCPDNTYEFMNRRCVQEGECRRMRKPREALNTVKEYPYKPFN
GSCIMECPAGYMEEEIDGKASCKKCKGICLKECSGINVESIATAQKLRGCTHIRGSLEIQIRG
GKNIVKELEDNLNMIEVIDGYLKIVPVLDNQNLQELWDWSTHPKITIGAEGTEPKLFFHFN
PKLCLQKIEELRIKAGLQPFTDLEVAPNSNGDKVACNVSKLHVRIHKRTAEAVLVGWEPFE
HHDSRSLLGYVVVYSIEAPEQNIAMYDGRDACGGDRWHVDDVASNEQKNDGNETRLQNH
ILTLLKPYTQYAFYVKTYTIATERSGAQSDLMYVTTMPGTPTPPRALTMWSNGSSELVIQW
QPPMRANGELTHYNVYGRMEPDDAEFLQQRNYCNEPIHLPEKKSIATMAAEERERAEVE
RQLAKQPENPTCQCSGDKNFNEEDDREKEVSSSIAFEDALHNQVYIKRTINHRRKRHIDLS
IPTDFETLYQQIFGDADKNEQCNDDDTCLAFKNKRETLDSQDNIEKELTDETIGNNTYMSF
VKRVMVQNNSKDELPSITITGLRHFAAYNIEVQACRQRADGGKDLDLNPECSTKTMRTYH
TLPLDGADDIPAGTFKLEMTGNSSQPVVQLQWAEPPEPNGIIVTYQIEYKRVDIQNIQAIPIC
ITRTDFLRANKYYYLRGMTSGNYSIKVRATSLAGPGAYSEVKYFIIDASTTSSYVWIILGVF
TSIILFSIVAFAFFCRRKIMGNVPNMRLIASVNPEYVSPTYVPDEWEVPREQIEVVRELGIGS
FGMVYEGIIKDADKEDAEELRVAVKTVRKKASDHAKAEFLNEATVMKAFHTNHVVKLLG
VVSCSQPTLVIMELMRNGDLKSYLRSHRPDNKDPKNQPTTEPPTLKRLMRMAVEIADGM

AYLTSKKFVHRDLAARNVMIAEDMTVKIGDFGMTRDIYETDYYRKGTRGFLPVRWMAPE
SLKDGVFTNSSDVWSYGVVLWEMVTLASQPYPGLGNDMVLKYVISGGLMECPDNCPNS
LYLLMRKTWHQKAHRRPTFIDITKILINNIHYEDFSQVSFYHSPGGIESRYLNVQHSTVKA
KDLEISFDDLWEDYIEEEEQTENSPLRRDFGDFACIEPGRMPDCGDSHNGSEPCGESSKLVT
NFHDLNSSEAPRKTVPIDSNGRSLNSSRDTLDCPFADSLRSSKNSPSIRHNNVSTTPHISPAS
ASNSIPLTKRNNTSMGNFANNSSLSKKTHDYENHTPEGVATIENKDTVSIRITCPSVEDIEV
DVINGDDRGEPSTVDHNNGTETLNGYIGNTAT
>Hheb003390.1
MGKNLMKTVVLGVLGIWWFLVICADGNSRLLRINNELATDKDTRKNESDRDLERRVERA
IGDSSSNDGNLLMDSVPSQPGYTELTPIPETNSSEIKAEKKKIKGTKRFKDITVAPGGICRTV
DVRNYLADLEILRPCRVIEGTLQVVLMENVDGNKTIEPFDQLREITGYLLFYRVANLKSIG
QLFPNLEVIRGQQLLADNAFMVYEMATLQELALPNLVEISRGGVMIQKNPSLCYAETIDW
DRIVRAGENSIRENNNNPSQCPVCTLNCPEGHCWSTRECQKMEKEECHPQCLGHCFGPTD
RDCHVCRNFKLDGQCIPECPKHLYNYLERRCITAEECWAIEEPDINSLETVHLTPFAGMCK
QTCPHGYEIGRDAKNMSTCVECKGKCQRSSLGVTIRRASEAQRLRGVVVIKGSLELQIRT
GNSNLIMSELAASLGQLEEIMGYLKVTHSFPITSLSFLKNLRLIHGTKTDTNNASLIVLDNP
NLSQLFPEDQEVKILSGKLFMHYNPKLCMSHIMRVVEKSGITNITNMEVEPESNGDKVAC
DIVDINITVSEKGPTYANLEWTAYKPATGQTLLTYLLNYVDTKYENITHDTNSCSGKQWQII
DVESDPESLDPVIKLKITDLKPYTKYAVYVKTMMTKDKNRAGSGTGQSRIIFFMTEPDTPD
VPIDVTSFSVSESEIMVNWLPPERPNGPLGYYKIAGFLRPENPSVLHNRNYCDFPPELYVPD
DVSEVTIKTPIVPKSCCDKDTSPSLSSHKFHIFCYDNMAISALPLNGRKQCDTQNHYGVNL
MSPMSSIGKQSSLDYTHDVLDYQMTRMVNDTYYSFVFDVPSRNTSYLLKKLRHYSLYTI
AVAACAEKRSNSGEMCSMFELTSAMTNKKDAADNIRKLEAQESNDTIVMLTWEPPIDPNG
LTVAYTIEWVNLMIKDAKSTSECLPASLFHGYKIISNLSPGRYSARVRAISLAGEGPWSEGV
TFTVGMDSSNISTIAMGITFFSIFGIVVFVFFLFRNHQKRKKQQRLIASVNPDYIESKYVKDS
WEVPRENVMIIRQFGEGNFGTVFWGILNGEKPVAIKTPPRGSSEEGKNEFLNEASVMKKFS
SHHIVRLVGVVSDGIPPYVIMELMENGDLKTYLRKLREAGQLSLDVPRIIRMASEIADGMA
YLESKKFVHRDLAARNCMVSKDIVCKIGDFGMTRDIYETDYYKVGQKSMLPIRWMAPES
LSDGVFTSDSDVWSYGIVLYEILSLAELPYQGLSNDEVMHHVMRKGTIDIDRDCPEVIQRV
MEKCFKWRPFERPTFMEIISELEPFIGQDFCEKSFYHSEQGVEIRNSGAKKVYHQAAQIRF
HWGNETARWIREFEDSAALLEPDKASTSRGKIFKNGFQQLGTEPIMEDVPLNR
>PPU05140-RA PTTH
MSCSSSRSLSLPVVFWLLLLLLLPPLQHVQALVLEDSLRLALCQARCPDDLKCIKECDSAS
NNVTNIPYLQTSSESNIHLQCRDANRLVLRHQAGIYVIETVDSFYNNWTSPTISTNCSHESA
NLMPEHRYRYRIRKVTSQGVSLPEITEWFQTNAETYVPKEVKNLQIGKINPNKNIPGHLQA
RLDVIPADDLNCQYNVVHWGGEHDLYQYQLDAAREYSLELRHLNYGRNNTVYVVSRNE
LGSRQSKNATLTFQTPPCLSVHRNLNVCAPAPVTGLHVVHQSNKNAVDFKVAWEKPELQP
NNYTIIVQSLDFENSSVEVTVSGNETAVDIAKVVPSHQYKIAILAESEGGTSLEWKLITADN
SGLTVESQVIIAISTLTTLVLVVVFGYTCIRYKRIKGHSSCRYSFFENINRKACGFHDEKGPL
KSHLSPYESEDNSGSSKMRDKFEIQMDRLSMKKILGSGISGVVKLATLQEDKKKIIQVAVK
MLREDASSDDVQNFHREIALMKSAGNHPNIVSIIGCCTLSKQPLLVVEFCSKGDLQTYLRT
ILEEMMTAVFKKVNWSTAQSNVPSTLQRESNNFSLAINNRLYDIQQESGMENSEINPEDLL
NFARQVASGMEFLSSNRIVHRDLAARNVLVCADQTVKISDFGLSRDIYQENVYKKQTNGK
LPIKWMAIEALTHQVYTTHSDVWSFGVLLYEIVTLGGNPYPGLPCCEILHFLKSGYRMERP

SNCGFQLYEIMRSCWHENPLNRPTFYNLKQQLDKLLTTFAGADQDEP
>PPU00285-RA NP
MHYISGDADAAYQEDRTLAADFGFRPPADRMNSLQKECAAKRDARSAFNLNRHRAQMK
LKMWGLRSVAILLILAFGIRAVNSNQCLTMLEEVNPKRTARLDGQTLNIQFEISRRVTQRLV
SRVMRIFLQEILGYTDITLVEKEDEFNVTATFARLSDNLTNSRKSMIPESMVNMEVWIPPHL
DTVPLLNKHDVLKVGVVAPPGHFGWFIPDRLSRLNDSWITFTKQETAARFDVDEVNLRKII
NATMKTDNEYYCQESFCHEGMYIPPQCQSHKLKSQPCALLLADYSEATKFIKDHIDNWKL
YVRVAWVGPNLKQIIKSLTKEYLQLTQNSALADRSLVILHWTPSNIIPNEREFASVEFPRCGS
RGTSMGCQYETSKLEKLVWNGLESIAKLAFEAINRVQYTESMYENLISKYNEKLINKYNS
YNKLSAMEQEVACDWLKENLNYTLDNWMPNDEDKNTLIIGGIFPMTGTFYTAKSIVLAA
SMARIAIDKNNTVLRDYNLKLLINDGQCKSDFVMKSFIDYILHNYYRKLIGVLGPACSETI
EPLAGVSKHYYTVIMSYGAEGSSFSDRSRYPYFFRTIGENRQYKHVYLQLLKKFNWNRVA
AFSEDGLKYTEYISYMQEMLRENGITFVANIKFPREWQPEILTKYLEDLKQKRARIIISDVY
DQVARLVMCEAYRLEMTAAQGYVWFLPLWLRENWYNTDYYNQLGENTPCSTVEMMKA
INGHLGISHATFAPDDSIMQEGITVREWRNNYEHRCAIQKEITSPYAGYTYDAMWTYAYAI
DKLLKENQSYIFDLHSEHTTARFTDIISKTDFNGVSGRIKFVGGPSRFPVINIYQHVDGKSR
LVGNFHPNISEELNQVIGGTLDLNMSAIVWLSGKKPDDGSEPPKKCVFSGFAELLNVSCEG
AIVIVNIIGFGLLGIFLVIVVILIKRKYDQKVRLHEKYMKSLGIDLLQPDTSSLDKWEIPRDR
VVINRKLGEGAFGMVYGGEAFFPEKGWLAVAVKTLKVGSSTEEKLNFLSEVEVMKRFEH
KNIIKLLAVCIKCEPVLTVMEFMLYGDLKTYLLARRHLVNDQHYEDSDEISNKKLTAMAL
DVARALSYLAQLKYVHRDVASRNCLVNAQRIVKLGDFGMTRPMYENDYYKFNRKGMLP
VRWMAPESLGLGIFTPASDVWSYGVLLYEIITFGSFPFQGMSNNQVLTHVKSGNSLTVPKG
VKPQLEGLIRSCWSVEHTKRPTAPEIVDFLATNQRVIAPCLDVPLASVQLEHTGEMEMQLN
TSTDRKFSFPWPGQSNKQNNKSPPTIDMPLVDINDSTSTKNQNIDSILGTSSTSEIESTKPLL
GTDTTDSVPSNSQSVGSSRYVNLQPGVTSAFLDVPSSDSDNYGIAMSERRSMLPATNNFN
NMPLL
>PPU07299-RA ILPs
MTTTTSSCNESTSCRCCCCSCGRELSDAVRLCPLCQRLQHDSEAGGDERRDRRRKNHHQ
QEDSGRRRRYDDNTEKDFRSTTTSTRRRRSASSVVRWVSDKCSRTKITRRTTGFWPGALS
TALVLLILLLTLGRAVAENAKTAVEGTAPNSASGKVCQSIDIRNKLSEFSKLDGCHVIEGFL
QIILLESTNEAHYENMSYPDLTEITGYFMVYRVQGLRSLGHLFPNLTVIRGHSLFMNYAFV
AFEMTSLQEIGLNSLTDIMRGSVRFEKNPVLCYADTVDWDLIAHAGKGGNVISANRPKNE
CPVCEKDCPRRQTNLEERLCWNKQFCQKVCTSCQDKDGQDRTCTIGSRPDNKTTCCHRN
CLGGCTGPTHLDCKVCRDVVVKQNECVSTCPADTYQFLNRRCITKRECQMMRKPREAPP
DTRPYPFKPFNNECTMQCPPGYEEVAEQDMWSCKKCTGPCLKECSGMVVDSIAAAQKLR
GCSHIRGNLEIAIREGQNIVHELEESLSNIEVINGYLKIFRSFPLISLSFLKNLVEIKGQELDM
KDYSLVVLDNQNLQQLWNWTGRPPLKIGSKNPVPKVSFHYNPKLCIQTIEELRERTGLNPF
REVEVSPTSNGDKVACNVTEIKTQVYTISSKAAMIKWEPFEHHDMRTLLGYVVYSKEAPF
QNVSMYESRDACGGDGWKVDDVSVTDDDNNRRQKIDTTISEKNPDSTSYNVTNNRSST
YTQYTILAPLKPFTQYAYYVKTYTISTERSGAQSKLKYFKTLPGQPSQVRALTIYSNASDTL
VISWLPPHESNSNLTHYEITGQMERYDLTFLRQRNYCIEPMQVLEYKPISEIAAEEKKRAEQ
ELEASKTPEPSACECKKREPVQSELDVYSSIAFEDALHNQVYVKRYDNRRRRDVSYTSVM
LKAVRMKRELTNYQEKDVISHSKKSLQNQSKTNSEQERTLEQQKVLNPDDDYIEDGAIVY
FKRKVPANELNFVMRGLRHYGKYNIKVTACRDKAANVDELPCSSESMKSLQTLRLDHAD

EIPDGTLRLQKQANNDSTTSIKLTWDEPPRPNGVIVTYQIEYKRVDVQYLKAEVACTTRDK
FNEDGNSYILQNLATGNYSVRVRATSFAGNGEYTEYKYFYVPESNYSTPTWKVILIVLLSIS
VILGACFIVAWFFKKKFMRNVPSMRLIATVNPEYVSTSYIPDEWEVPRKNIQLLKELGNGS
FGMVYEGIAKDVIKGIPEMRCAVKTVTENASDKERVDFLNEASVMKGFNTHHVVRLLGV
VSQGQPTLVVMELMMNGDLKSYLRSHRPDVCENFSRQPPTLNRIMRMALEIADGMAYLT
AKKFVHRDLAARNCMVSDDLTVKIGDFGMTRDVYETDYYRKGTKGLLPVRWMAPESLK
DGVFTSFSDVWSYGVVLWEMVTLASQPYQGLSNDQVLRYVIDGGVMERPENCPDILYG
MMRQTWCHKATGRPSFIDIVRELMLELDTTEFEKVSFYHSPDGVEVRNQNPSQYRTDKEL
EMAMQESHEEEAEGGEESPLRQDFGDFSSFEPMTRRHNGSSGNFGMEPFTDNSKSPMSLA
LGFLDLNSSKAPLKAGFDDFDGVSVGSLASSKDTLNLPFAEDSVKPAKNSPFTEKKSSSKG
NVSQSSAGKSSLSPQSPSIIATTCNLGSKPSPSLVSSTRSDPEYENRSPEVVDQPQQQPPTRD
VASLRVSFPSMDAIDSDETAIPETTSSIVVDSNSSSATLPSAKQQYANKSGEQQAAPLNNGY
IGGPAT
>NV17744-RA
MWGLRSVSILLILAFGIRVVNSNQCLTMLEEVNPKRTARLDGQTLNIQFEISRRVTQRLVSR
VMSIFLQEILGYTDIALVAKEDEFNVTATFARLSENLTNSRKSMIPESMVNMEVWIPPHLDT
VPLLSKHDVLKVGVVAPPGHFGWFIPDRLSRLNDSWITFTKQETAARFDVDEVNLRKIINA
TMKPDNEYYCQESFCHEGMYIPPQCQSHKLKSQPCALLLADYSEATKFIKDHIDNWKLYV
RVAWVGPNLKQIIKSLTKEYLQLTQNSALADRSLVILHWTPSNIIPNEREFVSVEFPRCGSRG
TSMGCQYETSKLEKLVWNGLESIAKLAFEAINRVQYTDSMYENLISKYNEKLINKYNSYS
KLSAMEQEVACDWLKENLNYTLDNWMPNDEDKNTLIVGGIFPMTGTFYTAKSIVLAASM
AKTAINRNNTVLRDYNLKMLINDGQCKSDFVMKSFIDYILHNFYRKLIGVLGPACSETIEP
LAGVSKHYYTVIMSYGAEGSSFSDRSRYPYFFRTIGENRQYKHVYLQLLKKFNWNRVAAF
SEDGLKYTEYISYMQEMLRENGITFVANIKFPREWQPEILTKYLEDLKQKRARIIISDVYDQ
VARLVMCEAYRLEMTAAQGYVWFLPLWLREDWYNTDYYNELGENTPCTTAEMMKAIN
GHLGISHATFAPDDSIMQEGITVREWRNNYEHYCAMQKELTSPYAGYTYDAMWTYAYAI
DKLLKENQSYIFDLHSEHTTTRFTDIISKTDFNGVSGRIKFVGGPSRFPVINIYQHVDGKSR
LVGNFHPNISEELNQVIGGTLDLNMSAIVWLSGKKPDDGSEPPKKCVFSGFAELLNVSCEG
AIVIVNIIGFGLLGIFLVIVVIIIKRKYDQKVRLHEKYMKSLGLDLLQQDTSSLDKWEIPRDR
VVINRKLGEGAFGMVYGGEAFFPEKGWLAVAVKTLKVGSSTEEKLNFLSEVEVMKRFEH
KNIIKLLAVCIKCEPVLTVMEFMLYGDLKTYLLARRHLVNDQHYEDSDEISNKKLTAMAL
DVARALSYLAQLKYVHRDVASRNCLVNAQRIVKLGDFGMTRPMYENDYYKFNRKGMLP
VRWMAPESLGLGIFTPASDVWSYGVLLYEIITFGSFPFQGMSNNQVLTHVKSGNSLTVPKG
VKPQLEGLIRSCWSVEHTKRPTAPEIVDFLATNQRVIAPCLDVPLASVQLEHTGEMEMQLN
TSTDRKFSFPWPGQSNKQNNKSSPTIDMPLVDINDSTSTKNQNIHSILGTSSTSEIESTKPLL
GTDTTDSAPSSSQSAGLARYVNLQPGVTSAFLDVPSSDSDNYGIAMSERRSTLPATNNFNN
MPLL
>Nv torso   XP_008211645.2
MRCSSTWPSLCVPVLWLLQVRASLFEDSLRLALCQARCPDNLKCIEECHSASNNVTDIPY
LQKSSESNVHLQCKDANRLVLKHRAGIYVIETLDSFYGNWTSPIISKNRFYESANLMPRHR
YRYRIRKVHSQGVSLPEITEWFQTNAETYYVPIAVKNLLIGEINPNKNRPGQLQARLDIIPAD
DLNCQYNVIHWGGEHDLYQYQLDAAREYSLELRHLNYGRNNTVYVVSRNELGSLQSEN
ATLTFQTPSCLSVHRNLSACAPAPVTGLHVAYQSNKNAVDFKVAWEEAELQPNNYTIIVQP
LDFENSSVEVTVSGNETTVDIPKVALSRQYKIAILAESEGGTSLEWKLITATTRLTVESQVII

AISTLTTLVLIVIFGYTYIRYKRMKGHSGCQYSFFENINRKACGFEDIKAPLKTLSPYEAED
NKSRDKFEIQVDRLSIKKILGSGISGVVKLATLQDDKKKIIQVAVKMLREDASSDDVQNFH
REIALMKSAGNHPNIVSIIGCCTLSKQPLLVVEFCSKGDLQTYLRTILEEMMTAVFKKVNRS
AESNGPQSILQRESNNFALAINNRLYDIQQESGVENKINPEDLLNFARQVASGMEFLSSNRI
VHRDLAARNVLVCADQTVKISDFGLSRDIYQENVYKKQTNGKLPIKWMAIEALTHQVYT
THSDVWSFGVLLYEIVTLGGNPYPGLPCCEVLHFLKSGYRMERPSNCGFQLYEIMRSCWH
ENPLNRPTFYNLKQQLDKLLTTFTGNEYLNLSELLREPTTQHRYFLKHDRINSNILTIPHII
>NV14476-RA
MSNLHRSTMTTTTSGCIETSSCRCCCCSCGRELSDAARLCPLCQRLQHDSKACGDERRGS
RRKNHHQREDSGRRRKYDDNTEKDFRSTTTSTRRRRSASSVVRWVSAKCSRTKIARTTGF
WPGALSTGIVLLMLLLTLGRAVAENAKTAVEGTAPNHASGRVCQSIDIRNKLSEFSKLDGC
RVVEGFLQILLLENTNEAHFENMSYPDLVEITGYFMVYRVQGLQSLGHLFPNLAVIRGHSL
FMNYAFVAFEMTSLQEIGLASLTDIMRGSVRFEKNPVLCYANTVDWDLIAHAGKGGNVIS
SNRPKNECPVCEKDCPRRPTNLDERLCWNKQFCQKVCTSCQDKDGRDRTCTVGSRPDNK
TICCHENCLGGCTGPSNLECKVCRDVVVNQNECVSSCPNGTYQFFNRRCITKRECQMMR
RPREAPAETRAYPFKPFNNECLMDCPAGYEEVAEKDMWSCKKCTGPCLRECTGIVVDSIA
TAQKLRGCSHIKGNLEIAVREGQNIVHELEESLSNIEVITGYLKIFRSFPLISLSFLKNLVEIK
GETLDMKDYSLVVLDNQNLQQLWNWTTRPPLKIGSSNPVPKVSFHYNPKLCLQTIEELRE
RTGLNPFREVEVSPTSNGDKVACNVTEIRTIVFSISSKAAMINWEPFEHHDMRTLLGYVLYS
KEAPFQNVSMYESRDACGGDGWKVVDIGVNEEDKIKMKKIDTSISDSNTNNVTNNRSST
YTQYTILTQLKPFTQYAYYVKTYTISTERSGAQSKLKYFKTLPGQPSIVRALTIYSNASDTL
VISWLPPTEANSNLTHYEITGQMERYDLTFLRQRNYCIEPMQVLEYKPIAEIAAEEKKRAE
QELEASKIPEPSTCECKKRDPVQSESEVYSSITFEDALHNTVYVRRQDNRRRRDVSYTSVM
LEAVRMKRELTNYQEKDVISHSKKSLQNQSKTNPEQERSLEQQKILNPDEDYIEDGAIVYF
KRKVPANELNFVMRGLRHYGKYNIKVTACRDKKANAHELPCSSESMKSLQTLRLDHADE
IPSGTLRLQKQANNDSTTSIKLTWDEPPRPNGVIVTYQIEYKRVDVQNLKAEVACTTRDKF
NEDGNSYLLQNLATGNYSVRVRATSFAGNGEYTEYKYFDVPESNYSMPAWKVILIVLFSIS
FILGAFLVAAWFFKRKFMRNVPSMRLIATVNPEYVSTVYIPDEWEVPRKKVHLLKELGNG
SFGMVYEGIAKDVVKGKPEMRCAVKTVNENATDRERVEFLNEASVMKGFNTHHVVRLL
GVVSQGQPTLVVMELMVNGDLKTYLRSHRPDVCENFSRQPPTLNRIMRMALEIADGMAY
LAAKKFVHRDLAARNCMVSDDLTVKIGDFGMTRDVYETDYYRKGTKGLLPVRWMAPES
LKDGVFTSFSDVWSYGVVLWEMVTLASQPYQGLSNDQVLRYVIDGGVMERPENCPDILY
GMMRQTWCHKATRRPTFIDIERELMLEVDTTEFEKVSFYHSPDGVEVRNQNPPSQYRTDK
ELEMVMQESREDEAEGGEDSPLRQDFGDFSSFEPMTRRKNGSSGHFGMEPFTDNSKSPTS
LALGFLDLNSSKAPLKAGFDDFDGVSVGSLASSKDTLNLPFAEDSVKPAKNSPFAQKKSSS
KGNVSQSSAGKSSLSPQSPSIIATTGNLGSKPSPSLVSSTRSDPEYENRSPEAVDQLQQQPPT
RDVASLRVSFPSMDAIDSDETVIPETTSSIVVDSNSSSATLPSAKQQYANKSGEQQAAPLNN
GYIGGPAT
>CG1389 torso
MLIFYAKYAFIFWFFVGSNQGEMLLMDKISHDKTLLNVTACTQNCLEKGQMDFRSCLKD
CRINGTFPGALRKVQENYQMNMICRTESEIVFQIDWVQHSRGTEPAPNATYIIRVDAVKDD
NKETALYLSDDNFLILPGLESNSTHNITALAMHGDGSYSLIAKDQTFATLIRGYQPSKMGA
VNLLRFVPQPDDLHHIAAEIEWKPSAESNCYFDMVSYSTNSVNMDEPLEVQFRDRKKLYR
HTVDNLEFDKQYHVGVRTVNIMNRLESDLQWLPIAVPSCLDWYPYNYTLCPPHKPENLT

VTQKQYLPNILALNITWARPRYLPDNYTLHIFDLFKGGTELNYTLDQNRSHFYVPKITVLG
SHFEVHLVAQSAGGKNVSGLTLDKVHREGNMVKLVLFIIVPICCILMLCSLTFCRRNRSEV
QALQMDAKDAKASEFHLSLMDSSGLLVTLSANESLEVMDELEVEPHSVLLQDVLGEGAF
GLVRRGVYKKRQVAVKLLKDEPNDEDVYAFKCEIQMLKAVGKHPNIVGIVGYSTRFSNQ
MMLLIEYCSLGSLQNFLREEWKFRQEQNAIGLKKNLEQNVDNRRFNRLPRNSIHDRIEDIN
NSMLSTVEEESESDQTHSSRCETYTLTRITNAADNKGYGLEDIENIGGSYIPKTAEAPKDRP
KRKLKPQPKKDSKQDFKSDNKKRIFENKEYFDCLDSSDTKPRIPLKYADLLDIAQQVAVG
MEFLAQNKVVHRDLAARNVLISVDRSIKIADFGLSRDVYHENVYRKSGGSGKLPIKWLA
LESLTHQVYTSQSDVWSFGVLLYEITTLGGMPYPSVSPSDLLQLLRQGHRMKRPEGCTQE
MFSLMESCWSSVPSHRPTFSALKHRLGGMILATNDVPERLKQLQAATESKLKSCDGLNSK
VEQVPCEEELYLEPLN
>AGAP005763-PA
SNASLVKIENLAPNRRYNVTATMLTSEYEYYYVEKHQYRTLPHDYMPGIVTDIMVERFET
NARDSRLVDAVISWTPAKDRTCHYEIVCHASYSPDFLLKPIDVQQPEVLYKYTIKALKLSA
NYMIAVRSKNTQNAMRESQLHWQSFHTPSCTNHTNGTQVCAPEPITNVRVTQVPLYGDH
YQLNISWDRPTIAPDSYVVKVFDLHNPETEEPGNSVTKNLTGDAVGVLIESFEMFGPHFEV
LVAAYSRVGVSSENTIKALQIGRVSSESWIRTKLVFIILTPVLMIGLLKISISLICRRRAKLKRY
EERCEYFKELEQKAPVDPSTEFEPTSKQMQDLLATSHPFSPDLIAPINDELEIEMEHIKLHD
MLGEGAFGLVRKGVLQRTGDDDEPAQPVAVKMLKECPRVEDILEFRREMEVMKSVGTHP
HIVGIVGHCTKNVRKMMLLTEYCGRGNLLNYLRLQWQRLLRQNRTTGTRSIGGTSGTNIT
ATLPSPPPPPPPPTRSLEMAEHECLTPSLDNNKMPENVFNFDTSFVNDKKSLTYKNISDHR
QTLLGVAFGLAGGSDGSSASRSPQIIENKLYPLFSDESDGDETAQLTHCTNACKNAVEIVEG
TWEVGEDVKGSVRIKPCSCHGAPVDSTLTNNTVENRWYQSCSPQSEEKEDPLPNSGQLLE
FSRQIALGMEFLARNKVVHRDLAARNVLVCDSNTVKIADFGLSRDIYQENLYRKTSNGKL
PIKWLALESMTHQVYTSQSDVWSYGILLYEICTLGGSPYPAISTNRLLRYLESGYRMERPK
SCSELLYDLMYSCWNLHPGERPTFSKIVHTVEQLQARDTANDPVVIDLSAIVDSHCTKNTT
EENSYLKPVEY
>AAEL002404-PA
MRNVSGDATALLIDSLAIHGARYELVISAYANNRSSHTTNINDAPIWRSPTDHWHGGRLAV
IILTPVLTLGLMKIFISIICKRRAKVKRYEQRCEYFKELEQKAPIDPSTGFEIKVKNIQEIVHPA
TFPSDLIAPINDEMEISVDQIRLLDLVGEGAFGRVRKGILLHPVGTYTEVAVKMLKECPSLE
DVKEFRREIEVMKSVGVHPNIVCIIGHYTQNVNEMMLLTEYCSEGNLLNFLRSEWHKVLQ
NRDRSASTTKQSLKALTPKDDVFEGCRSPSLECNKKPENVFNFDAPFIHDKKSFAYKNISD
HNTPEPTESAPEMKITENRLYPLLNDTFDNNFSLCAEVESNDLKDNTKICTNSCKCNVEIL
QSNSSEDIETWTKRTPCSIKVSGCECDSISNGSGAQSKQDEICNMVNNQCYYKELCSEKRN
QSEEYIITSRQLLEFAKQIAIGMEFLARNKVVHRDLAARNVLVCYDKAVKISDFGLSRDIY
QQNLYRKTGTGKLPIKWLALESLTHQVYTSQSDVWSYGILLYEICTLGGNPYPLLSTCDLI
MELKRGYRMEKPDSCSKELYELMLSCWSALPIDRPTFTSIHNRMEELMFQNMKKDMIDL
DAIIDIQSTKTSSSEHSYLKPVEY
>AAEL001915
MTAENGYIWFLPVWLTNLWNLSNDSPIRSMVRCTRQEMLKAINGHFSLAHAPFADSHSSL
DTIEGTVGKWRSDYRETLRRHSYMESDYAGYAYDAVWVYALALDRLIREDPSYLSDLHSI
KTTKRLMEVIRATDFQGVSGRIKFGDEGSRYTIINVLQWINGTPNIVGQFTPNISESKYKLL
GGSLALNQSAIVWMTKDGKTPEDGALDCTLSGLARFFGMGCDGTVYVLVGCLCVMTIAI

ISLASFCYFQVRYDRKMKHSAKYLQKFGIDLLSPSSIPVNTLDKWEVPKDRVVINRRLGEG
AFGTVYGGEAQIGDEGWTAVAVKTLKIGSTTEDKVDFLSEAEAMKRFDHNNIVKLLGVCL
QTEPVYTIMEFMLYGDLKTYLLARRHLVNSKQSEDSDISPKRLTMMALDVSRALSYLAEQ
KYVHRDLACRNCMVNAQRVVKLGDFGMARPTFENDYYRFNRKGMLPVRWMAPESLAL
GIFTPASDVWSYGVLLYEIISFGSFPYQGMTNNQVLEHVKEGNCLTIPTGVKPQLEGLMKA
CWNQDYKKRPSASEVSEFIANYPRLLSPCLDVPLASVQMAETDSDQFELLPGLRRRKDEP
TADLLLGTSQMNDLNQTTTGYTKMNMRRGINLNDLNVDTDFRRNTLPNAETTTTTTNGG
VTLNMYNPVEPLLQRQPEVAKSSNNILRYVPMFGLGRNKAPVLITHGNGSVTIGSRSTSTS
VL
>CG18402   InR
MFNMPRGVTKSKSKRGKIKMENDMAAAATTTACTLGHICVLCRQEMLLDTCCCRQAVE
AVDSPASSEEAYSSSNSSSCQASSEISAEEVWFLSHDDIVLCRRPKFDEVETTGKKRDVKCS
GHQCSNECDDGSTKNNRQQRENFNIFSNCHNILRTLQSLLLLMFNCGIFNKRRRRQHQQQ
HHHHYQHHHQQHHQQHHQRQQANVSYTKFLLLLQTLAAATTRLSLSPKNYKQQQQLQH
NQQLPRATPQQKQQEKDRHKCFHYKHNYSYSPGISLLLFILLANTLAIQAVVLPAHQQHLL
HNDIADGLDKTALSVSGTQSRWTRSESNPTMRLSQNVKPCKSMDIRNMVSHFNQLENCT
VIEGFLLIDLINDASPLNRSFPKLTEVTDYIIIYRVTGLHSLSKIFPNLSVIRGNKLFDGYALV
VYSNFDLMDLGLHKLRSITRGGVRIEKNHKLCYDRTIDWLEILAENETQLVVLTENGKEK
ECRLSKCPGEIRIEEGHDTTAIEGELNASCQLHNNRRLCWNSKLCQTKCPEKCRNNCIDEH
TCCSQDCLGGCVIDKNGNESCISCRNVSFNNICMDSCPKGYYQFDSRCVTANECITLTKFE
TNSVYSGIPYNGQCITHCPTGYQKSENKRMCEPCPGGKCDKECSSGLIDSLERAREFHGCT
IITGTEPLTISIKRESGAHVMDELKYGLAAVHKIQSSLMVHLTYGLKSLKFFQSLTEISGDPP
MDADKYALYVLDNRDLDELWGPNQTVFIRKGGVFFHFNPKLCVSTINQLLPMLASKPKFF
EKSDVGADSNGNRGSCGTAVLNVTLQSVGANSAMLNVTTKVEIGEPQKPSNATIVFKDPR
AFIGFVFYHMIDPYGNSTKSSDDPCDDRWKVSSPEKSGVMVLSNLIPYTNYSYYVRTMAI
SSELTNAESDVKNFRTNPGRPSKVTEVVATAISDSKINVTWSYLDKPYGVLTRYFIKAKLIN
RPTRNNNRDYCTEPLVKAMENDLPATTPTKKISDPLAGDCKCVEGSKKTSSQEYDDRKVQ
AGMEFENALQNFIFVPNIRKSKNGSSDKSDGAEGAALDSNAIPNGGATNPSRRRRDVALEP
ELDDVEGSVLLRHVRSITDDTDAFFEKDDENTYKDEEDLSSNKQFYEVFAKELPPNQTHF
VFEKLRHFTRYAIFVVACREEIPSEKLRDTSFKKSLCSDYDTVFQTTKRKKFADIVMDLKV
DLEHANNTESPVRVRWTPPVDPNGEIVTYEVAYKLQKPDQVEEKKCIPAADFNQTAGYLI
KLNEGLYSFRVRANSIAGYGDFTEVEHIKVEPPPSYAKVFFWLLGIGLAFLIVSLFGYVCYL
HKRKVPSNDLHMNTEVNPFYASMQYIPDDWEVLRENIIQLAPLGQGSFGMVYEGILKSFP
PNGVDRECAIKTVNENATDRERTNFLSEASVMKEFDTYHVVRLLGVCSRGQPALVVMEL
MKKGDLKSYLRAHRPEERDEAMMTYLNRIGVTGNVQPPTYGRIYQMAIEIADGMAYLA
AKKFVHRDLAARNCMVADDLTVKIGDFGMTRDIYETDYYRKGTKGLLPVRWMPPESLR
DGVYSSASDVFSFGVVLWEMATLAAQPYQGLSNEQVLRYVIDGGVMERPENCPDFLHKL
MQRCWHHRSSARPSFLDIIAYLEPQCPNSQFKEVSFYHSEAGLQHREKERKERNQLDAFA
AVPLDQDLQDREQQEDATTPLRMGDYQQNSSLDQPPESPIAMVDDQGSHLPFSLPSGFIAS
STPDGQTVMATAFQNIPAAQGDISATYVVPDADALDGDRGYEIYDPSPKCAELPTSRSGST
GGGKLSGEQHLLPRKGRQPTIMSSSMPDDVIGGSSLQPSTASAGSSNASSHTGRPSLKKTV
ADSVRNKANFINRHLFNHKRTGSNASHKSNASNAPSTSSNTNLTSHPVAMGNLGTIESGGS
GSAGSYTGTPRFYTPSATPGGGSGMAISDNPNYRLLDESIASEQATILTTSSPNPNYEMMHP
PTSLVSTNPNYMPMNETPVQMAGVTISHNPNYQPMQAPLNARQSQSSSDEDNEQEEDDE

DEDDDVDDEHVEHIKMERMPLSRPRQRALPSKTQPPRSRSVSQTRKSPTNPNSGIGATGA
GNRSNLLKENWLRPASTPRPPPPNGFIGREA
>AGAP012424-PA
MCLLLRGRAELCQSAQSLYKKCVYFCSSVDVRNTPLHLDRLRNCRVVEGFVQIMLIDKY
GNDSFDNYTFPLLTEITGYLLLFRVNGLQTLGQLFPNLTVIRGSELANNYALVVYELMHIKE
LGLTSLIDIQRGGVRIEKNPNLCHADTIDWKAIAPYGENWIKGNQDGNECTTCPSNVTVSL
PSGITQTIRCPLRDVNRLIHNEKASHLCWSTNHCQQKCPAHCPKSCNKTGECCSTSCLGQC
SSNNKSHCMVCRKYYYIHNNQTRCVDKCPDHMFLFSESRCLTEEECYKIYKPLQRIADIT
DNYPYVPAQGECRLDCPLGYTLTRATGSQRLACVPCKGPCRSECKGMVIESISQMQQLRG
CTIIQGSLSIRLRQLGGENVVRELEKVLYSIEEIYGYLTIVRSYALMSLGFFRNLKIIHGTVLN
ANLSLSVIDNQNLQELWNQNVTIKRGNVRFNDNPMLCVKKITSLKSHFDEGVGIENEEQL
NKTNGVRVACEIKKLNTHPTKISLELAVIQWDAFKDLPDMRQLLGYVVVYYIEAPHENVTF
YDGRDACNSQGWRVDDVAYIQENDDATHILTKLQAFTQYAYYVKTYTLSSENLGGQTDIT
YFRTSPGTPRIVKDLHVYIDNDTLIVSWQEPTKINGKLSAYRIGATLNDERNEMIRQRNYC
HDGMIYSFTSIATTVVPAKDNQNQCSKEECEAFCKTSSISESDSGPKIDINEVEMSIGFEDW
LHNYVYIKNPKSSRKRREDGSSSYYRSLGATTNQTRITFPLSHFKHFALYSFRVLACREPAA
KVPGVVVQDMADACGPEAMFIYRTPTKPEADDIPIDSIELDDQSNHTQRVIRVRWKGPSKP
NGVLVSYSIKYHRTDLDSVQPTVRCVTVDVHTLLGYALLTKLEAGNYSVRIMATTTAGNG
PYSPPKFLYLEKRDTDSTVVTWWVITATILIIMVLVIGVVYYLKHNYIPMSNMRLFAQVNP
DYAGVTYKVDEWEVPREHIIQLEELGQGSFGMVYKGIMTKLGNDVNVPCAIKTVTENAT
ERERDSFLIEATIMKEFHTHHVVRLYGVVSVGQPTLVIMELMANGDLKSYLRRHRPDYEN
GEESSPQPPTLKQIYQMAIEIADGMAYLAAKKFVHRDLAARNCMVAEDLTVKIGDFGMTR
DIYETDYYRKGTKGFLPVRWMAPESLKDGMFSSSSDVFSYGVVLWEMATLASQPYQGLT
NDQVLRYVIDGGVMERPENCPDKLYELMRICWQHRASARPSFIDIIRMLLPDANDNFKRV
SFFFSPDAMDNSIQPGNGNISPNIFRNLINFIVFDFVFHFIYRIVR
>AAEL002317-PA
MALSGQNMHNLGVCGSVDVRNSPAHLDRLKDCVVVEGFVHILLIDKYIDSSFENYSFPLL
TEITEYLLLFRVNGLKSLRRLFPNLAVIRGDALVGDYAMVIYELMHIEEIGLISLMDITRGG
VRIEKNPKLCFANTIDWKAMTVPGTNNYIKDNQKDNVCPICPAESTAVMLPNGSKQKCPA
APVRGGNKDHKRTLCWNANHCQTICPPECPKACSKTGVCCDAESCLGGCNLPNTSSCSV
CRHLSIDPAGKRQCVAKCPPNTFKYHTRCVTRDECYAMKKPISLDSNPDLPDQPFIPHNGS
CLMECPLDHELITELNKTRWCRKCSGTCPKRCEGSNIDNIQSAQLLKGCEIIDGSLEIQLRS
RGGENIVKELENFLSSITEIKGYLKVVRSYPLLSLGFLKKLKIIHGKGNKVSNSSLYVVENQ
NLQELFDHNVTIGEGKLFFFNNPMLCTDRIKAVKKYNPGIEIENESQLESNNGDRAACSITE
LETSLKSIGSETAIIQWAPFTELSDARMLLGYVIYYIEAPYANVTFFDGRDACNTEGWRLD
DISDFNMDKETTKILTQLKPYTQYAYYVKTYTLGSEGLGGQSKIKYFTTAPGTPSVVRDVE
VSVNKNMLTVKWLPPLKMNGRLKEYEVFIELNADDNEQLMLRDYCEDDKLRDIVPETPT
SAPPPKTSICTADQCRNYCKAPTSGGSTGTIDVTDKENQITFEDQLHNYVYIKNPLLRDKT
TRRKRSTNLLFPNNTENKKNDTTDRRTEKVKDEPYYQYIFNATNETSITFPLSYFNHYSLY
VFKIRACRHPGDPPAPSVRLVDVELACGNEVFENFRTPKKEGADDIPPESILIEEQSNNTQR
QIRVQWKEPSKPNGPIVKFVVKYQRVDLESVSSTDICIRYSSFNQTRGALLTKLEPGNYSIR
VMATTIAGDGAPSAARYVLIAKDDSMGTTLIWLGTLIVIFLCSVGFVAFYWYKYRYMSKQ
IRMYPEVNPDYAGVQYKVDDWEVERNHIIQLEELGQGSFGMVYKGILTQLRGEKCNQPC
AIKTVNESATAREKDSFLLEASVMKQFNTHHVVRLLGVVSQGDPTLVIMELMANGDLKS

YLRRHRPDYENGEDPSPQPPTLRQIIQMAIEIADGMAYLSAKKFVHRDLAARNCMVADD
MTVKIGDFGMTRDIYETDYYRKGTKGFLPVRWMAPESLKDGIFSSSSDVFSYGVVLWEM
ATLASQPYQGLTNDQVLRYVIDGGVMERPENCPDNLYNLMRRCWQHRPTARPTFMEIISE
LLPDASPHFQDVAFYNSQDALDMLRGQHQTVIIDEATTPLRPGDDHDEEPGEDDDLVGHG
EGHIGDVGTDDEFSMEMTNSHLVRNNGPMATIRSPHSPLR
>CG7223   Fibroblast growth factor
MAAAWSWRASHSTITMTSGSLVVLFLLLSIWQPAVQVEGRRQMANSQEMIKDHLGARSQ
NKTPAITNNANQSSTSSADLDDGAADDDDNKADLPVNVSSKPYWRNPKKMSFLQTRPSG
SLLTLNCHALGNPEPNITWYRNGTVDWTRGYGSLKRNRWTLTMEDLVPGDCGNYTCKV
CNSLGCIRHDTQVIVSDRVNHKPILMTGPLNLTLVVNSTGSMHCKYLSDLTSKKAWIFVPC
HGMTNCSNNRSIIAEDKDQLDFVNVRMEQEGWYTCVESNSLGQSNSTAYLRVVRSLHVL
EAGVASGSLHSTSFVYIFVFGGLIFIFMTTLFVFYAIRKMKHEKVLKQRIETVHQWTKKVII
FKPEGGGDSSGSMDTMIMPVVRIQKQRTTVLQNGNEPAPFNEYEFPLDSNWELPRSHLVL
GATLGEGAFGRVVMAEVNNAIVAVKMVKEGHTDDDIASLVREMEVMKIIGRHINIINLLG
CCSQNGPLYVIVEYAPHGNLKDFLYKNRPFGRDQDRDSSQPPPSPPAHVITEKDLIKFAHQI
ARGMDYLASRRCIHRDLAARNVLVSDDYVLKIADFGLARDIQSTDYYRKNTNGRLPIKW
MAPESLQEKFYDSKSDVWSYGILLWEIMTYGQQPYPTIMSAEELYTYLMSGQRMEKPAK
CSMNIYILMRQCWHFNADDRPPFTEIVEYMDKLLQTKEDYLDVDIANLDTPPSTSDEEED
ETDNLQKWCNY
>AGAP003108-PA
MVSLHQKLRFERVRKTDAGYYSCGSEFHEWSNLTLSVFSQETDQYQSDGAGMANLERKS
LHSSSVAAAAAATPILDNELKEGPPRILGTRDEPYPSPVVRGVGEAYRLKCDAVGQPAPHV
SWLKDGAEYRDNSYKSTIVFKQLLPTDAGTYVCNVCNVYGCVNATTVLEVVESGEHEPA
YIQHNTMESHSQLQGDPATQDTQVMMRALGRYYPAGATEKRPQHHTYQPEDADMDEDE
DEDGEEEEEEEEGPTNGTGVHGSDTTEDSAGAVNGSGTPPPDRAPIFTKKDQMTKIVSKPSG
NMVRLRCPADGYPKPNITWTKDGRKIERAMGQVKKVNWAIVLEDLVPQDSGSYTCAVCN
QVGCINFTTKLEVKDRFPARPHITERPKNVTALVNTTVIFSCPILSDLEPHIEWVKLGLVDLE
NMSIPENVTKLKRDPDNPEVLTLENVTHADEGWYTCIAANSLGATNESAYLQVLDELPPD
DTPTAHPVRTHSTLIMGMTIFLCACFTVLAVIVIIVCKKLKREKMKHRAMEHVNQWTKKV
IVLKQPVVESSIPGMSEALQMPIVRIEKQRSTLVQSGNCDPTMISEYEFPIDLNWEFPRNKL
HLGKSLGEGMFGKVVMAEAHGLVKGHPSTVVAVKMLKEGHTDADVKDLVCEMEVMK
MIGKHVNIINLLGCCCKDGPLYVIVEYAPHGNLKNFLRSHRFGSNYEATNEKEKKILTQKE
LISFAYQIARGMEHLASRRCIHRDLAARNILVSDNYVMKIADFGLARDIHDQEYYRKTTTG
KLPIRWMAPESLEEKFYDSQSDVWSFGVLLWEIMTLGGNPYPSIPTWDNLLEHLKKGKR
MEKPPLCSIEIYLFMRECWHYRPEERPTFSEIVQHLDRLVSITSNEEYLDLGLPQLETPPSSD
DSDDDVEEDEEDCDDQAGTEHERVHMYRFNRSYNNDCIY
>AAEL002172-PA
MVSGFVQGLFSIELSIKYHHAYHAIHEVMSFTKISSFNSSHFRLKFYPVKKTDAGYYSCGS
EYGEWSNLTLSVISQHEASDHYQNDSPGSGLGPASSTLTRVANLEKKSLLPALPLSQPSPSP
AAPLDVVDNDLTDSSYPTVNGGSMITVTKQVGENYKLRCNGTAEPNSQISWIKDGEVVSN
TKNRPNLMLHSLIVDDAGVYICRVCHAQGCVNSTTILEVIDDPKESYIHYKNMEIHSQVSD
DEEPDDPDYVDDEHDDYEDAEEDEEVDEEISATGSQQGSADGEFTESQSAPPRANGQLPP
GPPVFTKEEHMVKLMPKPSGNMVRLRCPADGNPVPNITWTKDDEKIVRSMGSVKYAKW
SIVLEDLVPKDSGKYTCHVCNSHGCINFTTKLEVKDRVNHKPILTKPLTNITAVVSTNVSME

CKVLSDLSMYIQWFKFKGLCHDCAIIENKVMDMSNSDNPEVLTLENVTYADEGWYTCVA
ANTLGASYESAYLRVVDEFLVDPPIVHPVRPHSTLITIMTTVLSGCFMILAVIVVIVCKKLK
REKMKHRAMEHVNQWTKKVIVLKQPVIENSIPGVTESMQMPIVRIEKQRSTLVQSGNCDP
TMISEYEFPIDLNWEFPRSKLVLGKSLGEGAFGKVVMAEANGLVKGQASTVVAVKMLKE
GHTDADVKDLVCEMEVMKMIGKHVNIINLLGCCCKDGPLYVIVEYAPHGNLKDFLRSHR
FGTANYEDMISGEKEKKILTQKELISFAYQIARGMEHLASRRCIHRDLAARNVLVSDGYVM
KIADFGLARDIHSQEYYRKTTTGKLPIRWMAPESLEEKFYDSQSDVWSFGVLLWEIMTLG
GNPYSSIPTWDNLLEHLKKGKRLEQPPLCSIDIYLFMRECWHYRPEERPTFSEIVQHLDRL
VSITSNEEYLDLGLPLLETPPSSDDESEDNDNDGCERVRMYPFNHHSHTTESTF
>AAEL004932-PA
MLLKFYPVKKTDAGYYSCGSEYGEWSNLTLSVISQHEASDHYQNDSPGSGLGPASSTLTR
MANLEKKSLLPALPLSQPSPSPAAPLDVVDNDLTDSSYPTVNGGSMVTVTKQVGENYKLR
CNGTAEPNSQISWIKDGEVVSNTKNRPNLMLHSLIVDDAGVYICRVCHAQGCVNSTTILE
VIDDPKESYIHYKNMEIHSQVSDDEEPDDPDYVDDEHDDYEDAEEDEEVDEEISATGSQQ
GSADGEFTESQSAPPRANGQLPPGPPVFTKEEHMVKLMPKPSGNMVRLRCPADGNPVPNI
TWTKDDEKIVRSMGSVKYAKWSIVLEDLVPKDSGKYTCHVCNSHGCINFTTKLEVKDRV
NHKPILTKPLTNITAVVGTNVSMECKVLSDLSMYIQWFKFKGLCHDCAIIENKVMDMSNS
DNPEVLTLENVTYADEGWYTCVAANTLGASYESAYLRVVDEFLVDPPIVHPVRPHSTLITI
MTTVLSGCFMILAVIVVIVCKKLKREKMKHRAMEHVNQWTKKVIVLKQPVIENSIPGVTE
SMQMPIVRIEKQRSTLVQSGNCDPTMISEYEFPIDLNWEFPRSKLVLGKSLGEGAFGKVVM
AEANGLVKGQASTVVAVKMLKEGHTDADVKDLVCEMEVMKMIGKHVNIINLLGCCCKD
GPLYVIVEYAPHGNLKDFLRSHRFGTANYEDMISGEKEKKILTQKELISFAYQIARGMEHL
ASRRCIHRDLAARNVLVSDGYVMKIADFGLARDIHSQEYYRKTTTGKLPIRWMAPESLEE
KFYDSQSDVWSFGVLLWEIMTLGGNPYSSIPTWDNLLEHLKKGKRLEQPPLCSIDIYLFMR
ECWHYRPEERPTFSEIVQHLDRLVSITSNEDI
