# Supplementary material for: Genome-Wide Identification of Neuropeptides and Their Receptors in an Aphid Endoparasitoid Wasp, Aphidius gifuensi
Source: Insects. 2021 Aug 18;12(8):745. doi: 10.3390/insects12080745 (PMC8397052; doi:10.3390/insects12080745)
Supplement: Supplementary file 1 [file insects-12-00745-s001.zip › Supplementary Files/Table S2.pdf]

Table S2. Primers used in this study.

Name	Primers	Size (bp)
DopR1 (KAF7998503.1)	F: CCTTGTTATCTGAGCTGTTTGTGGG	234
	R: CACAAAAACGTGGACCAAACATCC	
DopR2 (KAF7997032.1)	F: GGCAAGAAGAAATTGTTTCGGCTG	245
	R: TGTTCCCATTCCACTCACGTACAA	
TAR (KAF7998304.1)	F: ATGAATGCCAGTGGTGAATCAGC	129
	R: TAAAGAGGCAGCTGCTGCTTCC	
AstAR (KAF7996862.1)	F: CAACTTGTATTGCGTGGCTCGTT	233
	R: TCCACGTGAACTTCGCCATAATC	
sNPF (KAF7990818.1)	F: ATTGGTTTTGCAGTGGCTAGAC	197
	R: CGAAGTGATGGTGAACGATGTGAT	
ITP (KAF7996763.1)	F: AGAGAACCACAGCTTCATAC	147
	R: AACCGAAGGTTCAGCACCATGA	
OAR (KAF7995300.1)	F: GACGACGCAAGATCCAACGACA	136
	R: TCCTACTGCGACGGATGTATCTGG	
CAPAR (KAF7988142.1)	F: TGGACTACCATTTGAACTGAGAGGC	172
	R: CAGGTGCAAAGGATGGCATATTG	
SIFaR (KAF7995013.1)	F: ATCGTTCACCAAGAATGCGTACTG	133
FMRFR (KAF7994648.1)	R: CGACCAAGAACCCAAGGAACAAG	155
	R: CGACTCATTGCACAGTAGAGTA	
