# Supplementary material for: Genome-Wide Identification of Neuropeptides and Their Receptors in an Aphid Endoparasitoid Wasp, Aphidius gifuensi
Source: Insects. 2021 Aug 18;12(8):745. doi: 10.3390/insects12080745 (PMC8397052; doi:10.3390/insects12080745)
Supplement: Supplementary file 1 [file insects-12-00745-s001.zip › Supplementary Files/Table S3.pdf]

Table S3. Summary of head transcriptome.

	Transcripts	Unigenes
Raw Reads	49,641,558	
Clean Reads	48,430,060	
Clean bases	7.26G	
Q20(%)	94.98	
Q30(%)	88.85	
GC(%)	28.32	
Mean Length	351	293
Min Length	101	101
Max Length	19,032	19,032
N50	546	370
N90	143	133
Total Nucleotides	85,137,487	63,959,957
