# Supplementary material for: Genome-Wide Identification of Neuropeptides and Their Receptors in an Aphid Endoparasitoid Wasp, Aphidius gifuensi
Source: Insects. 2021 Aug 18;12(8):745. doi: 10.3390/insects12080745 (PMC8397052; doi:10.3390/insects12080745)
Supplement: Supplementary file 1 [file insects-12-00745-s001.zip › Supplementary Files/Table S4.pdf]

Table S4. The amino acid sequences of neuropeptide precursors. Predicted signal peptides (highlighted in yellow), cleavage signals (red), putative bioactive mature peptides (light blue), amidation signals (pink), and cysteine residues (deep yellow) are indicated.

>KAF7998325.1 hypothetical protein HCN44_009723 [*Aphidius gifuensis*] Allatostatin A
MTKRKFIKIILIISLSLIDVIASINTEKLSILRRPLREINKHRCIVDNKKDYDIVTEYKRLPENR
YISEYKRYPDYRYSFGIGKRWFNDNTQRNLPYSFGIGKRENIDKINQQFSNDYVIDDVSND
NNIFENEKLQIKRVPLRKYNFGIGKRDIHDDDQQNDIEINHEKLNDDNQQVWDEN

>KAF7990447.1 hypothetical protein HCN44_000252 [*Aphidius gifuensis*] Allatostatin CC
MGMILLPFIIMATLSATTVARVFPKRTISTDLSSSDTIDYPDYSFKSEIYPVGTKRAALLLD
RLMVALQKAVDDQGIGDSRDERGLRKSEPIIEIPNIGALRSGSTKMDLQRRGHVNNSVYW
RCYFNAVTCFKKK

>KAF7990446.1 hypothetical protein HCN44_000251 [*Aphidius gifuensis*] Allatostatin CCC
MMMSRKVIYIAIFIIGFIGFCSALPADKERFLNELDLVDDDGSIETALINYLFTKQIVKRLHS
QMDVNDLQRKRTYWKQCAFNAVSCFGK

>KAF7994885.1 hypothetical protein HCN44_004357 [*Aphidius gifuensis*] Bursicon alpha
MFNVLTIFLLGTVSGTMIAQQNDENCETLTSEIHITKDEYNEAAILVRTCSDDVSVTKCEGF
CNSQVQPSISTPTGFLKECFCCRESYLKERMVTLHHCYDADGVKLDGEMSTMEIKLREPA
DCKCFKLLHCQQSLVEGVIGVDECQ-

>KAF7994885.1 hypothetical protein HCN44_004357 [*Aphidius gifuensis*] Bursicon beta
-
VTRVLHTLRAPGCVPKLIASYACTGRCSSYLQVSGSKDWQMERSCMCCQESGEREASVSL
YCPKAKPGEKKFRKVITKAPLECMCRPCTSVEEYSIIPQEIAGFAEEGPLTSSAHFRRSTSIQ

>KAF7994999.1 hypothetical protein HCN44_004471 [*Aphidius gifuensis*] CAPA
MKDYPIITVIIFFLFTSLNCGEKTNDRRASGMLALPRVGRAPSFARPDGVAGLVQYPRVGRS
GQAAVNNLEKIYSVNNDIDSSDAEAHQEETIDLNYQGEPLK

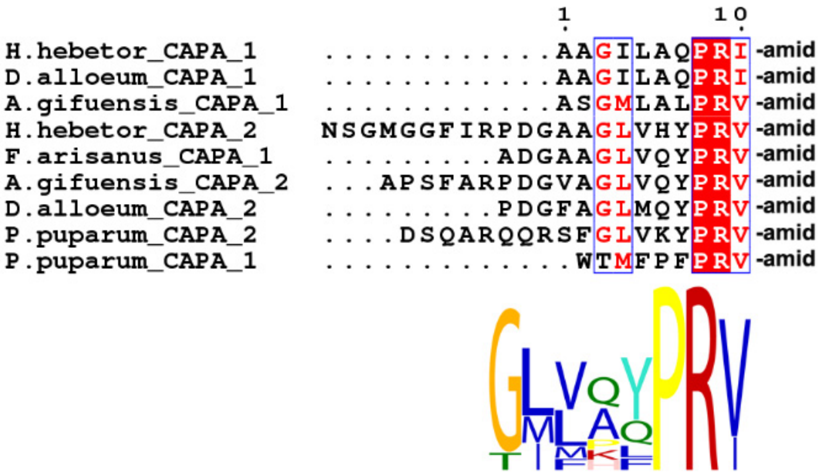

>KAF7995080.1 hypothetical protein HCN44_004552 [*Aphidius gifuensis*] CCAP
MKASKILNWAVAGLLLIASTNGLKIDEKDDQEIVESPFRDGSMRSKRPFCNAFTGCGKKRS
YNNNALESKEDLQKIQVPIDIYKAMIRDLSEDIRNTIEHEIDEQNTKYIDQEYLPYSTRTMPI
RKRYQN

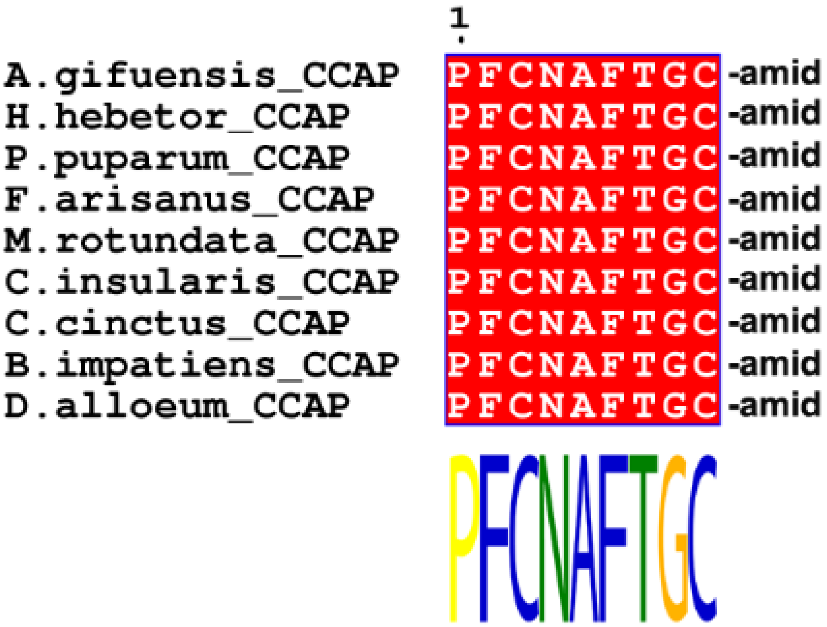

>KAF7990444.1 hypothetical protein HCN44_000249 [*Aphidius gifuensis*] CNMamide
MNNRVMTNKIRRHLTCLLIILSVSTCYGEPEPLPGMIYAENFADDTEGFLLLQRLKQVAEL
KNEMIEDEQELKEAQYEIQAMLEAKARNHRIQPIQTEFSGEQSEMLPIPSARVNQGSINSG
KRTSYMALCHFKICNMGRKRQSRADLNGKL

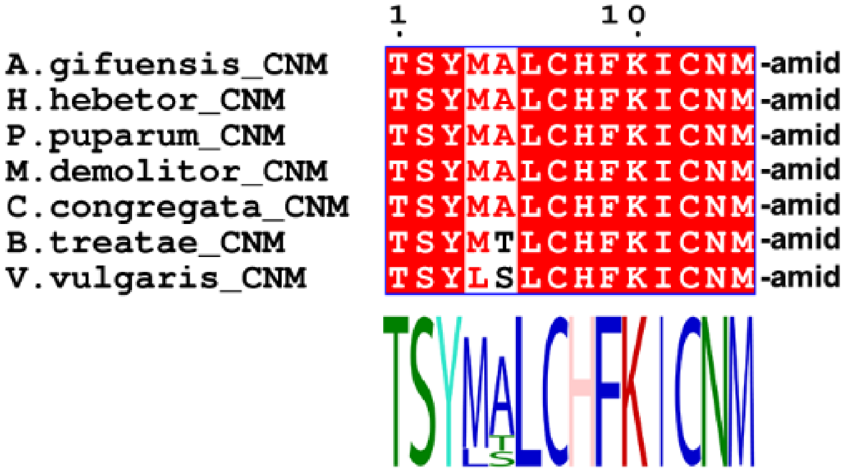

>KAF7991797.1 hypothetical protein HCN44_010598 [*Aphidius gifuensis*] Corazonin
MANVTSVSMILLVFIITVVSSQTFQYSRGWTNGKRSDIGTFGLGLPTEFKMNFPLQEIKRQ
TNDPSTIQCGLRQLKMLLQENSNDQIYSIPCDLLNILTKVIEEYQFATNTFQRSALNKDID

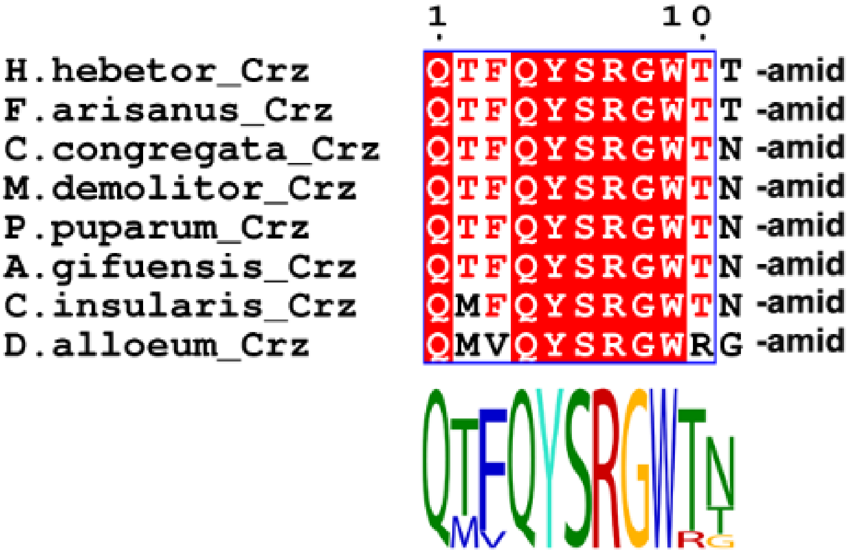

>KAF7996337.1 hypothetical protein HCN44_001969 [*Aphidius gifuensis*] Diuretic hormone 31

MLRKITLVWSLLIVFGVFGVFTTPVKANPFPEEREIYLDELEDPKYLIRMLTNLDGYLKQQENAKRGGSFNLDLGLGRGYSGAQAAKHMMGMAAANYANGPGRRRRSEQA

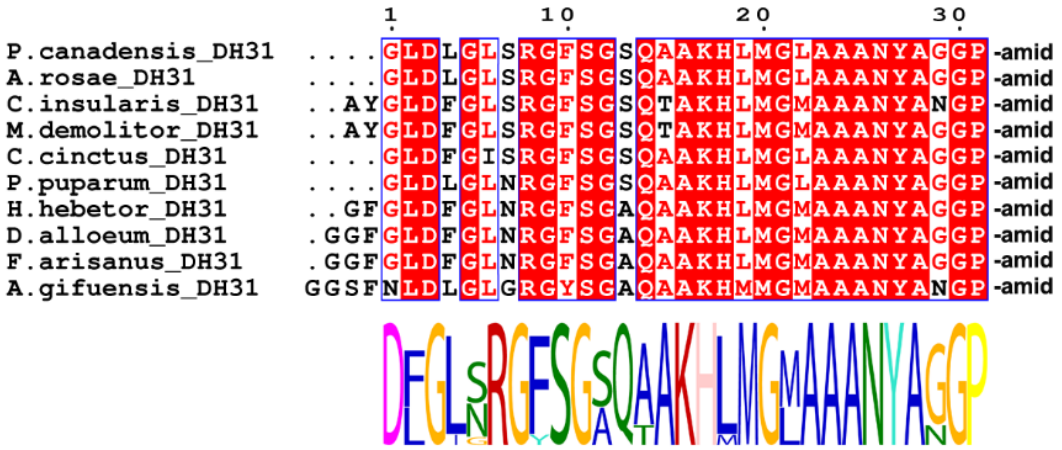

>KAF7994603.1 hypothetical protein HCN44_004075 [*Aphidius gifuensis*] Diuretic hormone 44

MMILISLLVASTLISMTKSSPLSYTAYHKRELNDDADQLYLPYLINDHILSTNKDNWPQISKESMANIKNIKTSLGRMKKRIGSLSVVNSLDVLRERMLLELARRKAMQDQKQIDANRRFLNSVGKRSVQDDMNYSKFELNNNDDIFDNQLMNNQAKNYNPTRNPLERTIDWYNVDDADEHNDDNEQSKMETNELGIL

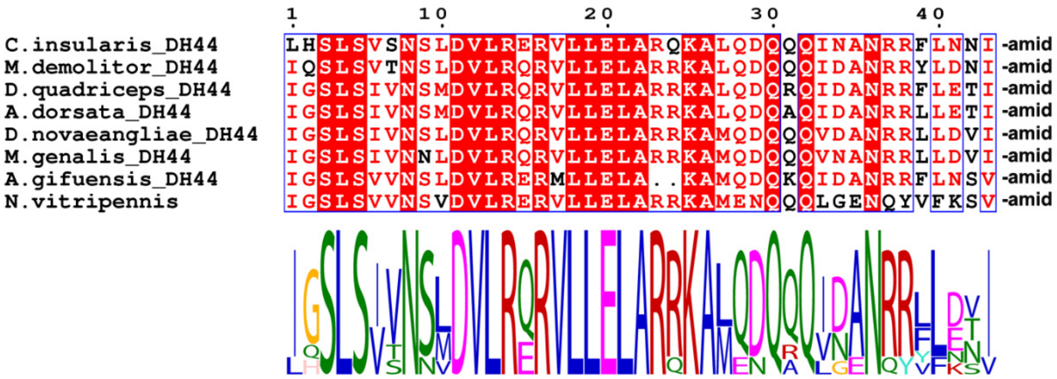

>KAF7992721.1 hypothetical protein HCN44_005065 [*Aphidius gifuensis*] Eclosion hormone
MRISTRFVLLMIAAFVIVILSTTTSASVPTVGICIRNCAQCKKMYGDYFSGPRCADDCVKF
KGKTIPDCEDTDSIASYIHALEEN

>KAF7993382.1 hypothetical protein HCN44_007885 [*Aphidius gifuensis*] ETH
MSKFKCLNFFSYHVSIATCMAVLILIIAGSYVAADEVPAFFLKIAKNMPRVGRSDDKLNEYF
LKTAKYPTRVSRRDTYSPTNKEAGPNGSRENYNEIHKRMINYKSPESSETSAWGNFPLAIE
GPPELWRTLASYTNDRYGSLPDDIDNEIWRRDKRMSMNSNQMAEP

>KAF7993326.1 hypothetical protein HCN44_006386 [*Aphidius gifuensis*] FMRFamide
MFNTQSGEYSRLVASDTERESYLAQWDFRYPANLKDSNGVTSIIATDYISYAIKTLVQPAQN
GKPGNGEYSRLPASDTEREPYLAQWDFRYPANLKESNGVTSILATDYITYAVKTLVQKAQ
NGKPGYELIVWILSREKTLSKSCEKTINNFFFGLVIMTINIGYLSAKNLSPLKVDSSAFHLYK
SRNELDYILKNLRLQNIDDTKDNQYINFGQNQDGENENLNYLPTSFISIDPKTKVTNDQSD
IIIRFARSGAFDIDSLENFRLLSLDIQQLLVLCGDFNNIDSTDIGLTQLCNYITAGLNE

>KAF7991605.1 hypothetical protein HCN44_008976 [*Aphidius gifuensis*] IDLSRF
MVRVISTSLAIGLALTACACFPRVASAIDLTRFYGHIHSKRSGDACHPYEPFRCPTDGTCISI
QYLCDGAPDCQDGYDEDARLCTAAKRPPVEETASFLQSLLASHGPNYLEKLFGSKARDA
LQPLGGINAVAIALSESQTIEDFGAALHLMRSDLEHLRSVFMAVENGDLGMLKSLGIKDSE
LGDVKFFLEKLVNTGFLD

>KAF7990199.1 hypothetical protein HCN44_000004 [*Aphidius gifuensis*] ILP1
MTKIKKLLFLIITYFVLQNVKNSESSPTTGLSFIFKVGMMVMKNNNYFGKDKIPLGSSILEV
SKKIDNLERTIKATSKSSIREINRLIQYNQIMNQFDKNLNYVDNMYEQFQKYFSGFMYEKN
TLLDFARSCTSHANNQLLSILDSIHRAVVPNNLATVGLFDALINNRQTKVIIAEKNNALICNI
TASLEQEIYGVYGMVSLAQVKGYTMARFSYEILKTMEQGQFVYEVNDLIDKFINVVTTIG
VKATNIMSIASRDIRPCDPDNHKQGETYEQLNKFIQGFVTNEVSLNSQHTCDKTCESYKRT
SLYPYETVPSKGIHNCQGSLHDCFEELGTTMCPPNNDANNDRRYGGFKGVTILLKKDEPD
KGDVTKCSKHGNTLHEIFGNVQSGYLNVCSVCMCLCDDYNNIETARTFSLRKVETNIVEN
LVITGIKLVKKKRVFYIQTLQGKLEADGKILNTSLQWVSVADVDYKNPNVSKSDYHTLNY
DERSVNLDTLASPDGYAMTGIRFLKIKGHLSLGIKVTPFNFTSGLLQGDENSEWITNSVND
RSEIVISNAGIPTLSRDENIIHMEKNTFVKFGWSDYLKDIQQTPIPFLDIQPVVADPPAPLSG
AGIYYKGQPGFGGFVGLKLVAYSYIDLVTTKVGE

>KAF7994890.1 hypothetical protein HCN44_004362 [*Aphidius gifuensis*] Ele
MKMSRGRLIMILSLFAWCAYVEAGRQTRPVDCEKYVYHPHCRGIQAKKRVYPAEIKIDNT
EQPCVCSPSSSSSSSSPGTKDSNNRRTTFSNSKLLDAMLNGLDMNTIYEAYAASSDRHRNY
NDITRERNSQRRRQPIDNTIDINDLDLLDY

>KAF7991194.1 hypothetical protein HCN44_002756 [*Aphidius gifuensis*] Inotocin
MMKEIIVMSLLVSASLACLIVNCPRGGKRGESPFLSMQGLVKECQSCGPGHQGQCFGPNIC
CGTNIGCFIGTPETYKCRMESLYSRPCIAGFAMCRDNNGRCAANGICCSQENCSIDATCKT
NEDYDKKIGQEYPRISSDNDQ

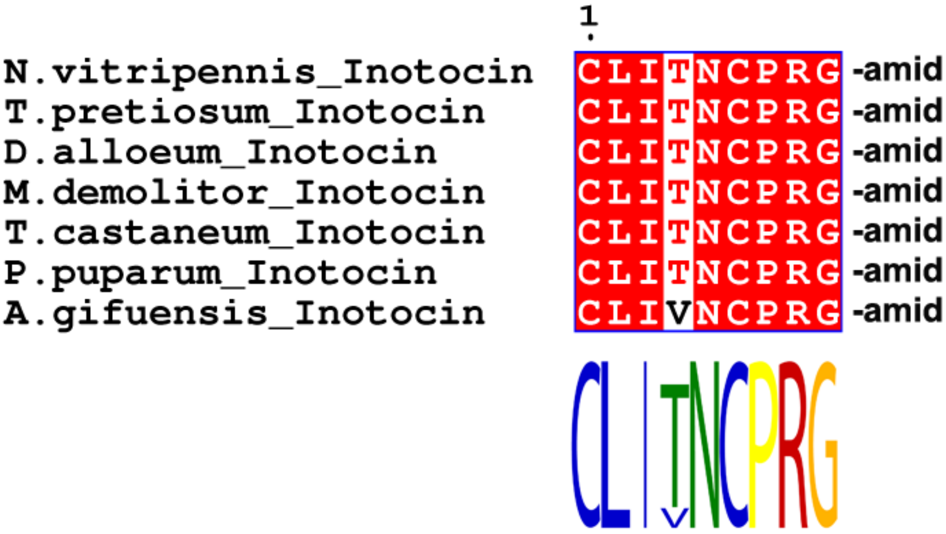

>KAF7998553.1 hypothetical protein HCN44_010961 [*Aphidius gifuensis*] ITG
MKTDKTHGSTAFYTIVVLMSIFEIQGGVYAWGGLFNRFSPEMLSNLGYGGHGNAYRPSYL
ERPLTGNYANNFGEGLEISNADEPCSGKRCIANEHCCPGQVCIDVDGASTGTCFFAYGLKQ
GELCRRDNDCETGLMCADVVGADTRSCQPPVTSNKQYSEECTMSGECDITRGLCCQIQR
RHRQTTRKVCSYFKDPLVCIGPVAIDQVKNIVQYTSGEKRITNQGINRLLLKRGLFI

>KAF7996763.1 hypothetical protein HCN44_002409 [*Aphidius gifuensis*] ITP
MMHQRQLKKSPTNYGSNLCHTKIDNIEKSNIRSRVLSNRTTSTTLPSTSPSSSSRSSSRSTLF
ISSLSLNRLSSSTLLLSVLAWSVTLLLISSCIGFADAAVLYGHPLGKRSFSTLQCKGVYDKSI
FARLDRICEDCYNLFREPQLHTLCRKNCFTSDYFKGCLDVLLLHDELEQIQTWIKQLHGAE
PSV

>KAF7987704.1 hypothetical protein HCN44_003567 [*Aphidius gifuensis*] Leucokinin
MWQLMGITIILVTSKSLSIECGLTKNTASIQNDVNKELLNKNVNYLPKQLKKRSIVKLEFH
DDGMNYEVPKLPSSQGNIYSALSVLPGTQTDKERNNQLLTNWGGKRDSNAYKIDPKIRRP
ARVPFNSWGGKRDGEKKFPKNQENNYRTILNPYCRPLMKSWHSKFVDDDNNLVDKRIVI
NRMSGNDNQPPPFNPWGGRKRRDINYGKYYDKVKSINK

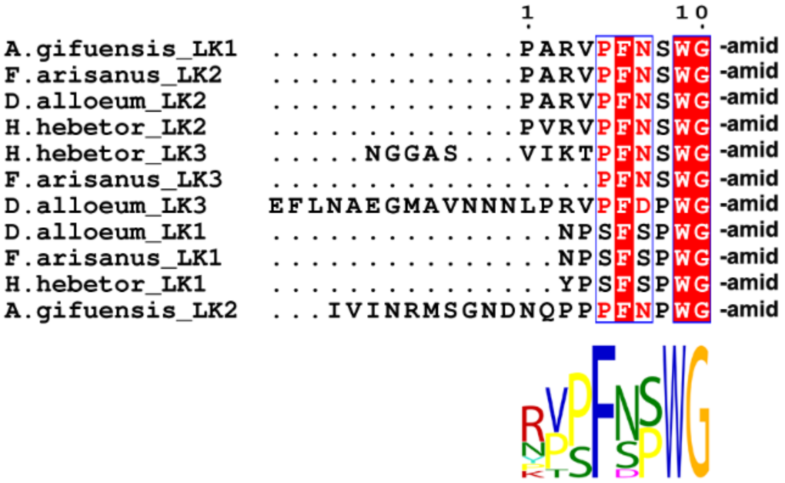

>KAF7994099.1 hypothetical protein HCN44_011368 [*Aphidius gifuensis*] Myosupressin
MKCNVSLIMSVVIFTVLTIQPIKIFGMPPAQCNPNLLDEVPPRIRKVCVALSTIYELGSAMEN
YIDDKVPVLHENIPLPDSGVKRQDAEHVFLRFGRRR

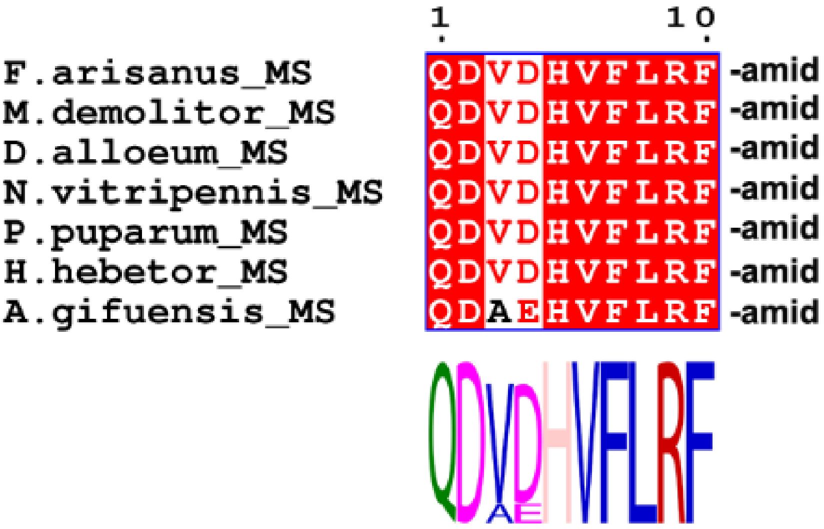

>KAF7987703.1 hypothetical protein HCN44_003566 [*Aphidius gifuensis*] Natalisin
MELILVIFLFALQGGSSIPAVRFLFDAKNTGGDTSPVLTRQYLWDLYKEHLSETPRMSQYDT
YIPSPSPIFSQLKNFRSSSSLSSSSLRNDNDERSYTKMSQCENIYKTKKPGYLLTMGENSDD
TKCRRTQNGWTIDESLFVDEPSWILVEENINDDKYPTKTEDPFYITRGKKSNEKNKKLYNS
TIVKRETLMTNDNNDSLNNENKKNDNIYKLNSRDNKNGYVLPRDKHADILDILQDEPFFI
SRGKKHNLKNNYNRNRRGDVENLLKNQDPFYVARGRRTSNRIWMPIMNLQK

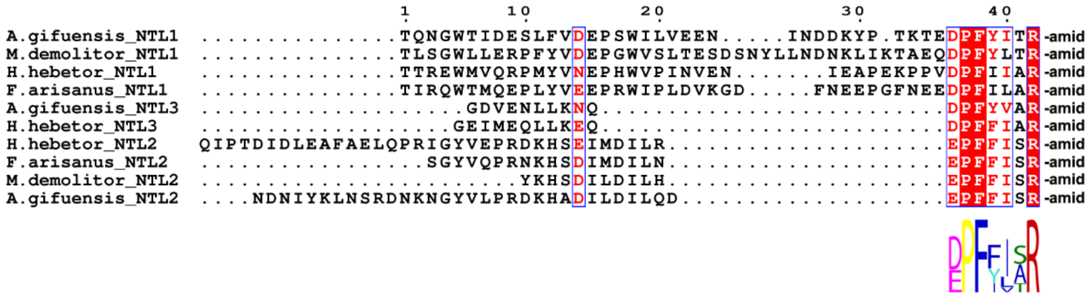

>KAF7991177.1 hypothetical protein HCN44_002739 [*Aphidius gifuensis*] Neuroparsin
MMTIKTIFIAAFILIIPSYTLSIPSIRFEPKERDDDVCQGCGVNCDNCKYGVTISSCRVLECRK
GPGDDCGGVKGTCGEGMICICDKCIGCSINTLECASSDIIRDCLPHRNRNSRYLNLERDNH
FTLVK

>KAF7997106.1 hypothetical protein HCN44_005383 [*Aphidius gifuensis*] NPF2
MLKILMIIVIMTIVLETNGEPDAMARPTRPKVFTNLNELQKYLELVKDYYSISGKARYGKR
AGDESMIPDDYRQILVRNNNIELSDNEYKVQNINKLIRQVMTQLKSTGNLQNRFTPKNDIY
I

>KAF7992678.1 hypothetical protein HCN44_005022 [*Aphidius gifuensis*] NVP
MLEETESPMKNEILDKVANSSAIFKRQKRDEPELTIEEQVANSIAIIKRQKKDEPELTIEEKY

KIANDLLDKNHVLFLAKFGKYLNVKLLDFFTGTNNYELTFYIEKLKRYFNNETRYIDIKNR
RYEALKVLIEQEDYFSEVEMMRRNPLLYDHLIGKYLTEDDIKTRDNLDTKNITFVNLLME
KIDRDGIQNLKKKQQDDEDEVFEEFDSDSDESDDEEKNNIKWGEMDNCDGKKLKDHNK
NSNNNSKKNIQCFDINYEEQQMLKKEFITHMYQSFLDGKDKDFDYSTVDDNEMYDNVDI
REQDEEEKYFDSETPDESTQLENQKIDDNESDDELEIYMKTLQ
MFFIGLRVFLVGIILVVVDGLAIGLLEDVTKADQVMRPKVKRAQELLMFGNQQNRRPESP
SAIATDDLTPSAEKRTLGTSGLEDVKAALADGETFNHQNPKQQITWGHLGESIPQREQNYE
YGKVMMNEVNDELPRVWDLQPYPRYFGLDDERRKRSEKTSVPSSVSPTQSTTTTTTKSP
SVTLSPSQKISTSSTQRPSTRLQQSKRSLPLVYQEPRYKRALIDREDLLTLLLLLENQQPRNR
NWRNWGNDEYENIEDDGSYPVIEDEDSRGNAQVDIPVYAPRHFGIGSDITPSEIGIPRTHPI
NSYEQYNNQYTPYENTQYGNAQYGSLYPHSNYDQQQPDKRFMISRKRSQGYDNYSGRN
LNDVIGYSQLMNTQQQGYGNYPQGMLY

>KAF7992639.1 hypothetical protein HCN44_004983 [*Aphidius gifuensis*] NPLP
MAKAPQKLAGLFIIFFTIAVDQLRLPMVNCQEDIDTTQCLPKKTFIAFLRLPEVSSNLAAYS
RTARIIQSARNDFINLKILGKNNDDDNGKICIPPSIYYEIFSDPKIRAHFDAIERAQKMLEIRD
VDSFIDTEKKSIATLAKNDDLPITTVQEREQKNQDDEEKRSQAGMTPEELVKVYDGDTQE
DKTSADVIDHYTLPNGDLDVEAMSRDYHNGKRNIATLARDYALPERRNIGSVARQYGLSY
GKRNIATLARDKMIPNSGGKRTVPLTYGRYMWLKGKRNVGSLARDFALPSGKRNISYLA
KNGWLNIGYQKRNVGTLARDWNLPQTRTGRSSIDDDFNYDDKINKQYDNDLQNIINQSQ
ARRQSDYSDEYPTPVMQNKNLLDYEDFIEALADVRYPVAEKRFMDATGDSWQDDDDDD
ETLGYRPSKRHIGALARLGWLPTFRAARFSRSPRWSDQEDSTDGTSSNNTPNSSSRLGINQ
SDLKTRYVQTLKGDCRHGFKRYLLIPTNHHYLDGKIRFASMNI

>KAF7990442.1 hypothetical protein HCN44_000247 [*Aphidius gifuensis*] Orcokinin-A
MSIARVICLTALFVLLITVYVNAVPVQIRGENDVVYADPAVVEALARSYAALAEARDINFQ
DGPFARQSRRGLDSLSGATFGQNKRFDSRNSKWLGNVNHVELLDGTAKRNMDEIDRSGF
DNYMKRNFDEIDRAGWDSFVKRSMGNSYPQTRQH

>KAF7995290.1 hypothetical protein HCN44_005950 [*Aphidius gifuensis*] PBAN
MASSIFESENGICLGSNCLEETGSDVSGAMWFGPRLGRRRRFEEKISLDDDKMNTITDGIN
LNGWPIQFYPGAGFKRQAPQFTPRLGRELANGSSKKYISYDDVGSRSFLLDNNEDLFDIQ
QRVRSLSIPLTSDFHFHHHLPVLLTRLGRQMMFQRKI

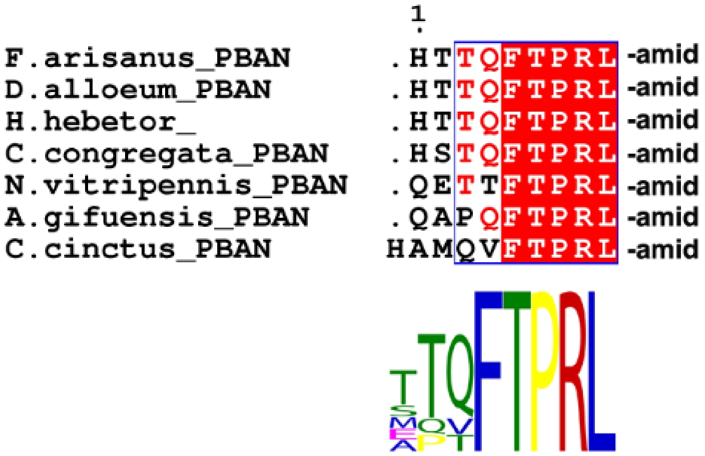

>KAF7994542.1 hypothetical protein HCN44_004014 [*Aphidius gifuensis*] Pigment dispersing factor
MIILIQIRNGYLVLEKINNTSTQTGTVGTSVDERELDSSNYQYAREVDNELQLARILMLPQR
ICHTKRNSELINSILGLPKNMNNAGK

>KAF7992386.1 hypothetical protein HCN44_001711 [*Aphidius gifuensis*] Pro
MIVFFFVAMLAKIKNGLQTAKNYLDTAKDIADLVSTSLGHNKHHEHGDRDNKNDKLSKR
LGNGNITSAFFRLLGLDTPRIAAIAINSAIFLTQMISSLFNIQQKTDKSKEKRSMDSDQFDPL
KFLLNTRNEKIQHLLKQAKNPELPNQLMDNLKDPDTSCVRLLMCKASPIILAAQESMENK
TTNRKFDITSWLPTKDEFEENGDTCEKNFSDCQVFPDM

>KAF7990443.1 hypothetical protein HCN44_000248 [*Aphidius gifuensis*] PTTH
MIFVKQPNYYRLQWSCPCESHYEILDLGAHSYPRYLSTATCASKPCHNKFNQCKLIHYKV
YVLRHRDINDSNDTSEEQFIEQPMLPEPFRVKWHLKPIITTVACVSASEGRMN

>KAF7990818.1 hypothetical protein HCN44_000623 [*Aphidius gifuensis*] sNPF
MNKEMKNSTGLIVWLFIIGFAVARQNYLSDDDINNIQDWKSNCEICKLFSIWNKAHNELNF
EYSQPLQEHLMTRKSHRSPSLRLRFGRRDSPKPN

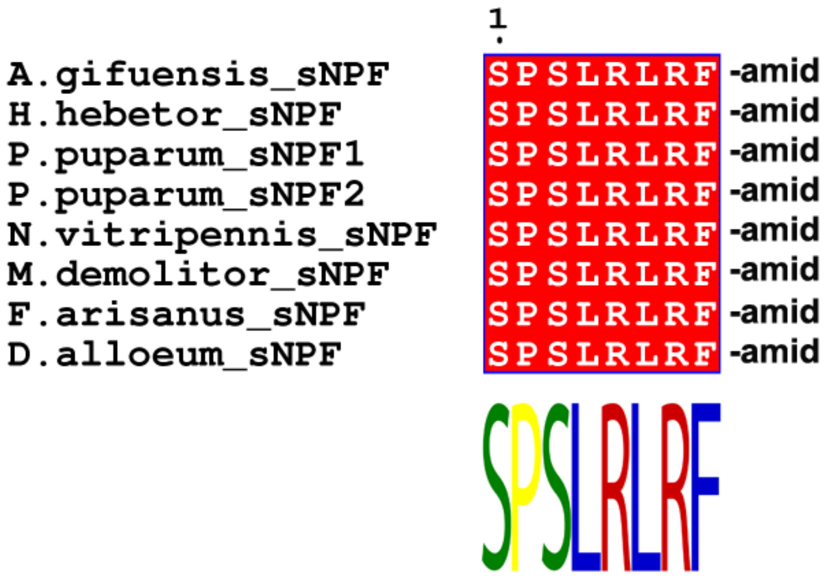

>KAF7993381.1 hypothetical protein HCN44_007884 [*Aphidius gifuensis*] SIFamide
MPVDEIVINNEIPVDETERENVPVEKKNDMANTARKYPTAAELFAITQLEIQENEPVDEEL
AKFFADLEQSALEENGLKEITYSEIKKPAITSVKILPPTKRIT
MNSKKQKEHSKDDNTEKEDPVLPIRSGKHGKKIITVDDEVKKTKEYSKFR
MSFVRVGLAIFIIALVACLSVEGGYRKPPFNGSIFGKRSGTMTDSEVMNRAMSSMCEMASE
TCNAWLLHQDSN

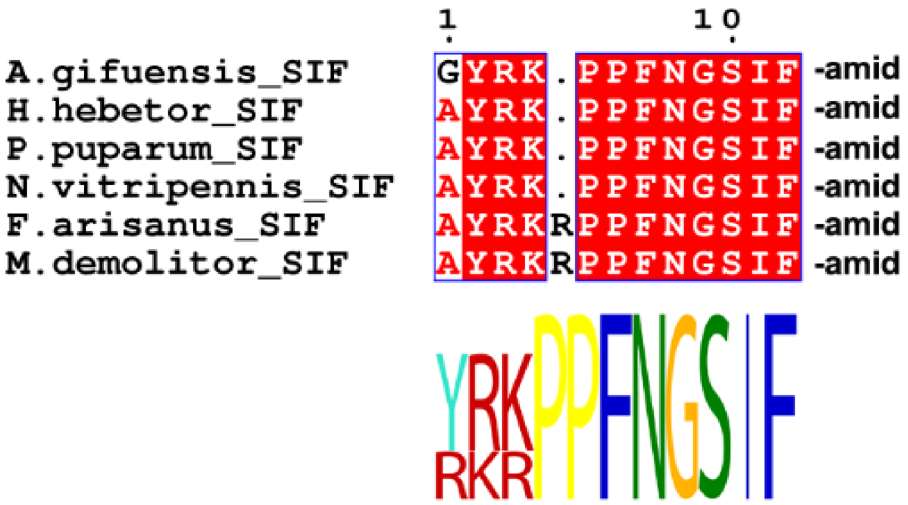

>KAF7988021.1 hypothetical protein HCN44_007515 [*Aphidius gifuensis*] Trissin

MKLFCIVFLIALFAIWTYGESLTCDQCGRECVTSCGTRQFRACCFNNLKKRVPNLGLKVW
LSPMRDNGHLKLVYDV
