# Supplementary figures and images for: Genome-Wide Identification of Neuropeptides and Their Receptors in an Aphid Endoparasitoid Wasp, Aphidius gifuensi
Source: Insects. 2021 Aug 18;12(8):745. doi: 10.3390/insects12080745 (PMC8397052; doi:10.3390/insects12080745)

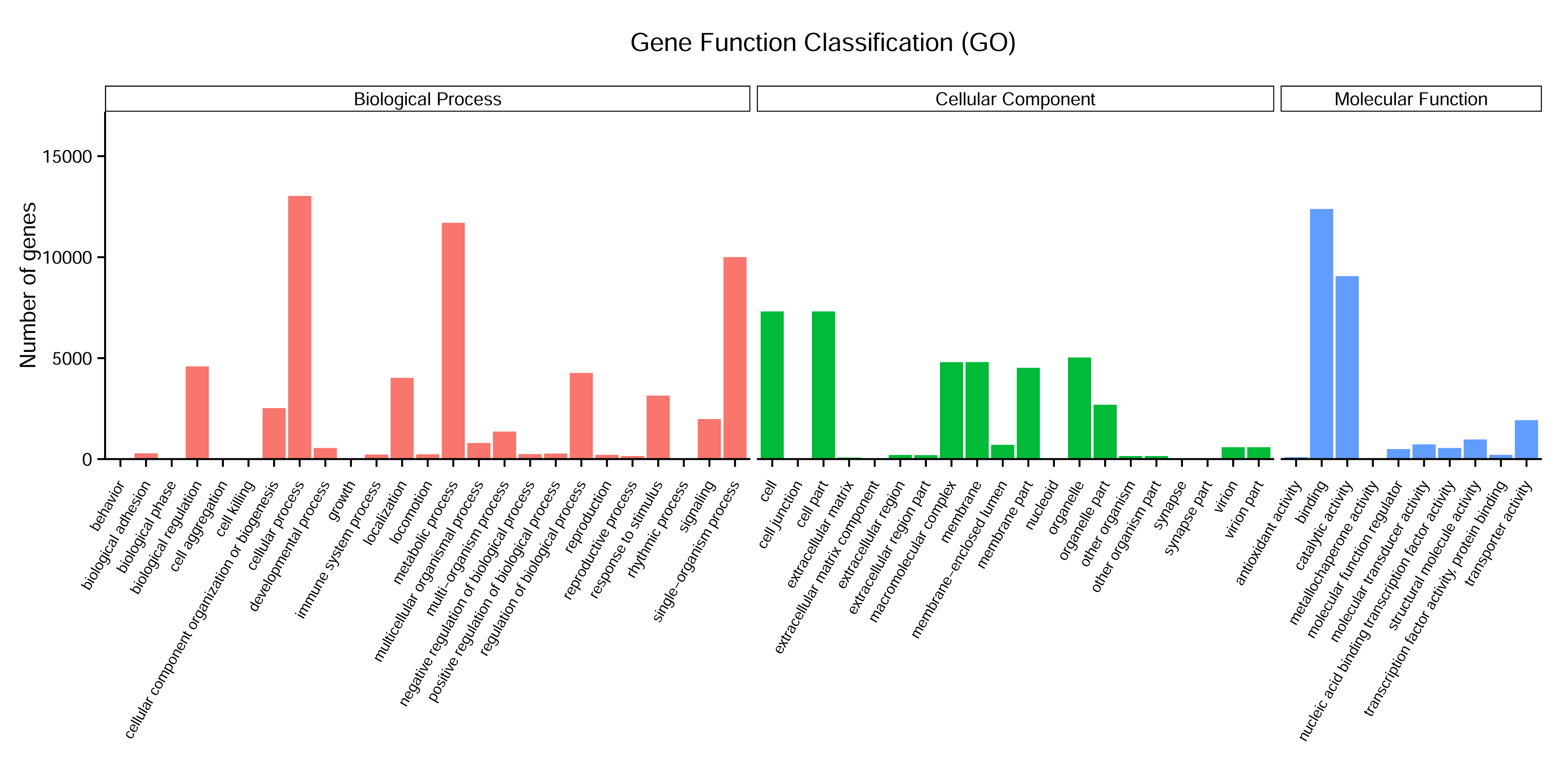

Supplement: Supplementary file 1 [file insects-12-00745-s001.zip › Supplementary Files/Figure S1.tif]

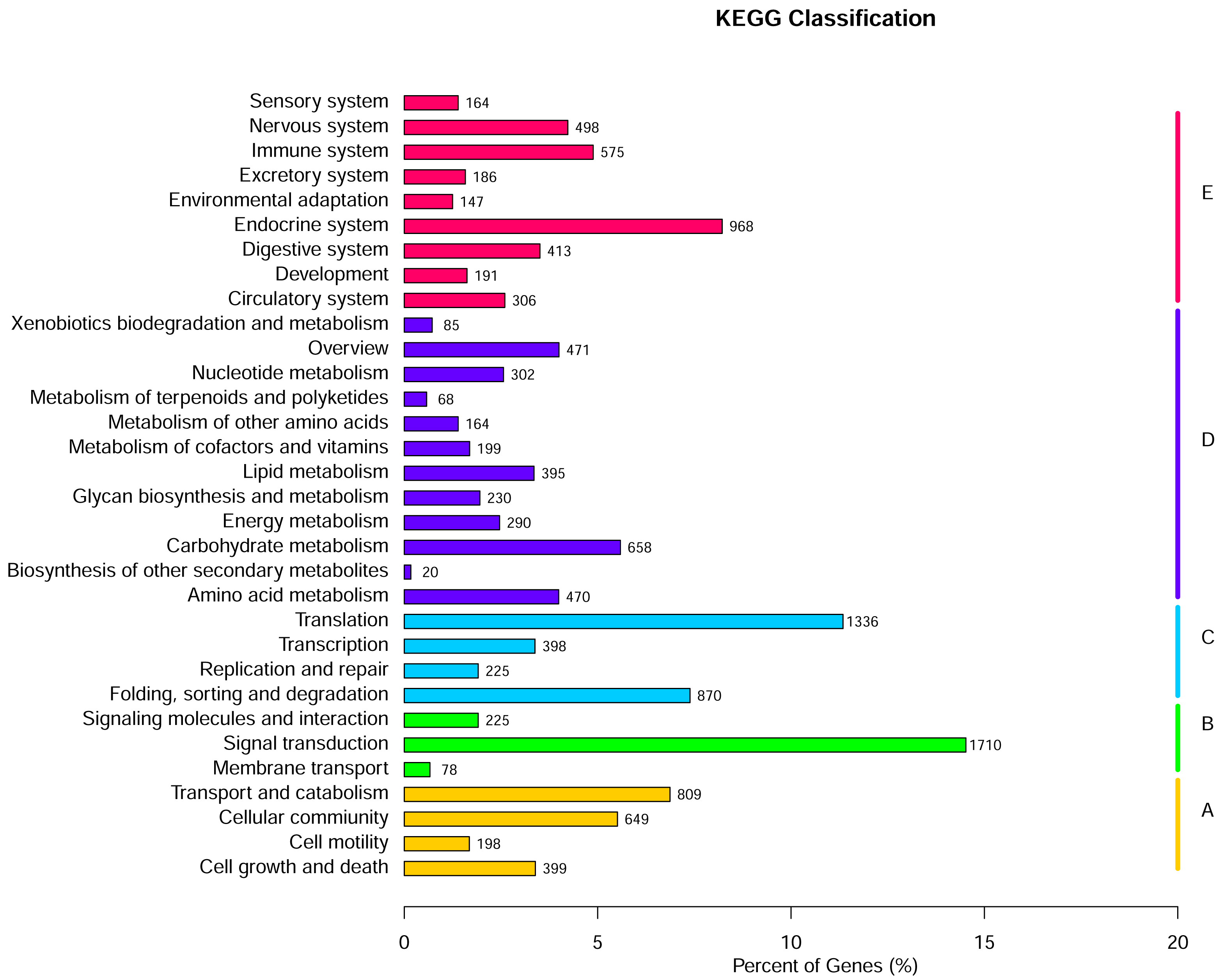

Supplement: Supplementary file 1 [file insects-12-00745-s001.zip › Supplementary Files/Figure S2.tif]
